# Luteolin Isolated from *Polygonum cuspidatum* Is a Potential Compound against Nasopharyngeal Carcinoma

**DOI:** 10.1155/2022/9740066

**Published:** 2022-12-23

**Authors:** Yu Xiong, Wenliang Zhong, Jie Liu, Bo Cheng, Jingying Fan, Fangliang Zhou, Lan He, Daofa Tian, Yingchun He

**Affiliations:** ^1^Hunan University of Chinese Medicine, Changsha, Hunan, China; ^2^The First Hospital of Hunan University of Chinese Medicine, Changsha, Hunan, China; ^3^Hunan Provincial Ophthalmology and Otolaryngology Diseases Prevention and Treatment with Traditional Chinese Medicine and Visual Function Protection Engineering and Technological Research Center, Hunan University of Chinese Medicine, Changsha, Hunan, China; ^4^Hunan Provincial Key Laboratory for the Prevention and Treatment of Ophthalmology and Otolaryngology Diseases with Traditional Chinese Medicine, Hunan University of Chinese Medicine, Changsha, Hunan, China

## Abstract

**Introduction:**

To reveal the mechanisms by which luteolin, the major bioactive component of the Traditional Chinese Medicine (TCM) *Polygonum cuspidatum*, inhibits proliferation and promotes apoptosis in nasopharyngeal carcinoma (NPC) CNE2 cells.

**Methods:**

Based on the Traditional Chinese Medicine Systems Pharmacology Database and Analysis Platform (TCMSP), bioactive compounds of *P. cuspidatum*, potential target genes and NPC disease targets of TCMSP were screened, relationship networks were constructed using these potential targets of NPC, and Gene Ontology (GO) analysis and Kyoto Encyclopedia of Genes and Genomes (KEGG) pathway analyses were performed. The predicted compounds, targets and pathways were corroborated using *in vitro* experiments, such as MTT, Cytation™ 5 real-time cell monitoring, cell cycle detection, Annexin V-FITC/PI double staining, Hoechst 33342 staining, and mitochondrial membrane potential (ΔΨ*m*) detection.

**Results:**

The results showed that 10 bioactive compounds (OB ≥30% and DL ≥0.18), 157 potential target genes from *P. cuspidatum*, and 56 common targets related to NPC were found. These included important bioactive compounds such as luteolin, quercetin, and beta-sitosterol. Key common targets included EGFR, MYC, AKT1, CASP3, CCND1, ERBB2, and common targets were enriched for the PI3K-AKT, JAK/STAT, MAPK, and C-type lectin receptor signaling pathways. The binding energy of luteolin for six common targets was less than -5.0 kcal·mol^−1^. After luteolin (20 *μ*M, and 40 *μ*M) treatment to CNE2 cells for 36 h, cell survival rates decreased, accompanied by cell morphology changes, inhibition of the cell cycle at G2/M phase, and an induction of apoptosis. The expression of the cell proliferation related protein PCNA, the antiapoptosis protein XIAP, and the PI3K-AKT pathway diagram related proteins p-ERK1/2, ERK1/2, AKT, and PI3K, all decreased.

**Conclusion:**

Luteolin derived from *P. cuspidatum* inhibited the proliferation of NPC CNE2 cells and promoted cell apoptosis through the PI3K-AKT signal pathway.

## 1. Introduction

Nasopharyngeal carcinoma (NPC) is an epithelial cancer originating from the endometrium of the nasopharyngeal mucosa, with obvious geographical distribution characteristics, and over 70% of new cases being found in East Asia and Southeast Asia [1]. With the advancement of imaging technology and effective population screening methods, the incidence and mortality rates associated with NPC have decreased over the past decades [2]. New therapies such as intensity modulated radiotherapy (IMRT) [3], concurrent chemoradiotherapy [4], and immunotherapy [5] have emerged. Nonetheless, early diagnosis of NPC, clinical management and prevention and treatment of recurrent and metastatic disease remains to be addressed. Traditional Chinese Medicine (TCM), also known as Chinese Herbal Medicine, has received increasing attention, promoting use on a global scale and leading to the development and application of many herbs as medicine [6]. The herb *Polygonum cuspidatum* has been commonly used in TCM. It is the dried rhizome and root of *Polygonum cuspidatum Sieb.et Zucc*, also known as *Fallopia japonica* and *Hu Zhang* which is used, and it is mainly produced in the Guangxi, Guangdong, and Jiangsu provinces of China. The Leigong paozhilun written 1500 years ago is the earliest ancient book from China recording the use of *Polygonum cuspidatum* as a medicine. *Bencao gangmu* named it “Hu Zhang”, *Jiangsu zhiyaozhi* named “Yingyanglian”, *Diannan bencao* named “Banzhuang”, and *Bencao tujing* named “Kuzhang”. *P. cuspidatum* is used to promote blood circulation, relieve pain, relieve coughs, dissipate phlegm, and promote choleretic action [7]. The traditional Chinese usage of *P. cuspidatum* was mainly as an oral water decoction, also as a topical powder, and an ointment [8, 9].

Clinical observations by Chinese researchers in 1977 confirmed that *P. cuspidatum* could treat burns and scalds, control wound infection, and also control Pseudomonas aeruginosa infection [10, 11]. It also has significant effects on the treatment of acute and chronic viral hepatitis, and acute jaundice infectious hepatitis [12–14] and can be used as a laxative [15] and natural dye [16]. Studies have confirmed that the multiple bioactive compounds found in *P. cuspidatum* had positive effects on most diseases and could be used as anti-inflammatories and anti-infection agents [17], and could treat hyperlipidemia and AIDS [18]. In particular, it is most associated with its preventative effects on cardiovascular diseases and tumors [19–21]. A previous study showed that *P. cuspidatum* and its bioactive compounds can effectively inhibit NPC [22], lung cancer [23], colon cancer [24], and breast cancer [25]. The antitumor effect of TCM *P. cuspidatum* has the advantages of multiple links and multiple targets, but it does not conform to the development trend of precision medicine and individualized medicine in antitumor drug research. Numerous bioactive compounds are active ingredients extracted from TCM herbs. They have clear structure and excellent pharmacological properties, which are convenient for research and medical application. Furthermore, there have been reports that a variety of bioactive compounds found in TCM have anti-NPC effects [26–28]. But, how can bioactive compounds of *P. cuspidatum* treating NPC and the mechanism of action of *P. cuspidatum* as an anti-NPC remains nebulous.

There are thousands of known bioactive compounds, and the workload of screening and researching compounds that exert effective anti-NPC activity is huge. It is necessary to adopt an efficient and high-throughput searching method to simplify the drug screening process. Nevertheless, there are few investigations and research methods searching for effective bioactive compounds against NPC from the numerous compounds in *P. cuspidatum*, but these search results are not accurate or comprehensive. In our study, we have used of network pharmacology, based on network database analysis and systems biology knowledge, to carry out multitarget and multichannel system network analysis and prediction of the effective active components of *P. cuspidatum*, and then search bioactive compounds with better anti-NPC effect, and interact more with NPC disease targets.

In this study, a network pharmacology approach was used to explore potential targets of *P. cuspidatum* and signaling pathways responsible for its anti-NPC effect. Then a bioactive compounds-targets-pathways-NPC network was constructed and subsequent network pharmacological predictions were validated *in vivo* using MTT assays, Annexin V-FITC/PI double staining, Hoechst 33342 staining, mitochondrial membrane potential (ΔΨ*m*) detection, and western blot. We found that luteolin derived from *P. cuspidatum* inhibited the proliferation of NPC CNE2 cells and promoted cell apoptosis through the PI3K-AKT signal pathway. We have applied network pharmacology to systematically search the bioactive compounds in *P. cuspidatum*, and used molecular docking methods and *in vitro* experiments to explore the interaction mode and signaling pathways between the effective compounds in *P. cuspidatum* and their targets with the aim of developing novel anti-NPC drugs. Therefore, our preliminarily study has helped elucidate the mechanism of action of luteolin, the bioactive compound in the TCM *P. cuspidatum*, and its ability to inhibit the proliferation of NPC cells and promote apoptosis. This may provide key technical support for drug development, clinical diagnosis and personalized diagnosis, and treatment of NPC. This investigation may provide a new feasible research idea for the research of TCM and the development of effective anticancer bioactive compounds in natural herbs, and may answer to the antitumor mechanism of TCM at the molecular level.

## 2. Materials and Methods

### 2.1. Data Collection

By searching for all the bioactive compounds of *P. cuspidatum* in the TCM Systems Pharmacology Database and Analysis Platform (TCMSP) database (http://lsp.nwu.edu.cn/tcmsp.php), all bioactive compounds were filtered with the oral bioavailability (OB) ≥ 30% and drug − likeness (DL) ≥ 0.18 as filter conditions. The targets of these bioactive compounds were searched out form the TCMSP database, the names of these targets were standardized through the combination of Perl language and Uniports databases, and the standardized target name (Swiss-Prod ID) of the bioactive compounds were obtained. Finally, with “nasopharyngeal carcinoma” as the key word, the disease target information was searched in the GeneCards database (https://www.genecards.org/) to obtain NPC-related disease targets.

### 2.2. Bioinformatics Analysis

*R* software (https://www.r-project.org/) was used to compare bioactive compounds of *P. cuspidatum* with disease targets found in NPC, and found where they intersect to determine common targets to construct a Venn diagram. STRING (https://string-db.org/) website was used to draw a protein-protein interaction (PPI) network diagram. A bioactive compound-NPC-target network was constructed with Cytoscape 3.7.2 software. Through the DAVID website (https://david.ncifcrf.gov/), Gene Ontology (GO) analysis, and Kyoto Encyclopedia of Genes and Genomes (KEGG) pathway analysis was performed on the common targets in this network, where *P* < 0.05 was used as the effective pathway filtering condition. Subsequently, computers were used to simulate a bioactive compound as a ligand docked to protein, to explore possible modes of binding interaction of bioactive compound with proteins. The top six protein structures with the highest frequency of common targets were downloaded from the RCSB PDB database (http://www.rcsb.org), imported into AutoDock Tools 1.5.6 software and subjected to Delete Water, and Add Hydrogens, for the target proteins in pdbqt format output. Use AutoDock Vina and *R* software to perform molecular docking and calculate binding energy, the default settings are energy_range = 5 and num_modes = 20. The smaller the binding energy, the stronger the binding of the ligand to the protein, PyMol software (http://www.pymol.org) was used to visualized the docking mode with the lowest binding energy.

### 2.3. Drug Dissolution and Dilution

Luteolin (Cat#B20888, HPLC≥98%) was purchased from Shanghai Yuanye Bio-Technology Co., Ltd (Shanghai, China) dissolved in DMSO (Solarbio, Beijing, China), and a 200 mM solution was prepared for storage. It was also diluted to a low concentration with RPMI-1640 medium (HyClone, Logan, UT, USA) as needed. Also cis-Diammineplatinum (II) dichloride (cisplatin, CIS; Sigma-Aldrich, St. Louis, MO, USA) was dissolved in physiological saline at a concentration of 3.33 mM(1 g/L) and stored until use.

### 2.4. Cell Culture

The human NPC cell line CNE2 and normal nasopharyngeal epithelial cells NP69 were purchase from Beijing Beina Chuanglian Biotechnology Research, and subcultured in our laboratory. CNE2 cells were cultured in RPMI-1640 medium containing 10% fetal bovine serum (Gibco, Waltham, MA, USA) and 100 kU/L penicillin-0.1 g/L streptomycin (Procell, Wuhan, HuBei, China). NP69 were cultured in K-SFM medium containing 2% fetal bovine serum and 10 kU/L penicillin-0.01 g/L streptomycin. Cells were incubated at 37°C, 5% CO_2_, and subcultured once every 2 to 3 days.

### 2.5. MTT Cell Proliferation Assay

At the logarithmic growth phase, CNE2 and NP69 cells were harvested and digested with 0.25% EDTA trypsin solution (Solarbio) to prepare a single cell suspension and 100 *μ*L per well was added to a 96-well plate at a cell density of 4 × 10^3^ cells/well. After the CNE2 cells became adherent, different concentrations of luteolin (5 *μ*M, 10 *μ*M, 20 *μ*M, 40 *μ*M, and 80 *μ*M) diluted in RPMI-1640 medium was added, including a control group (DMSO solvent control group, luteolin, and 0 *μ*M), and four repeat wells were used for each experimental group. Correspondingly, different concentrations of luteolin (5 *μ*M, 10 *μ*M, 20 *μ*M, 40 *μ*M, and 80 *μ*M) diluted in K-SFM medium was added, including a control group (DMSO solvent control group, luteolin, and 0 *μ*M), and four repeat wells were used for each experimental group. After incubating at 37°C with a 5% CO_2_ atmosphere for 24, 36, and 48 h, the supernatant was discarded and 100 *μ*L of MTT (500 *μ*g/mL) (Solarbio) was added to each well. The cells were then incubated for 4 h and absorbances (OD) were measure at a wavelength of 490 nm and growth curves were constructed, enabling the calculation of cell proliferation rate and the median 50% inhibition concentration (IC_50_).

### 2.6. Cytation™ 5 Real-Time Monitoring of Cell Proliferation and Morphology

CNE2 cells were added in a 96-well plate at a cell density of 4 × 10^3^ cells/well. After the cells became adherent, different concentrations of luteolin (2.5 *μ*M, 5 *μ*M, 10 *μ*M, 20 *μ*M, 40 *μ*M, and 80 *μ*M) were added to each well, including a control group and positive control group (cisplatin, CIS, and 10 *μ*M) using five replicate wells for each experimental group. The cells were then placed into a Cytation™ 5 multifunctional cell imaging microplate detection system (Perkin-Elmer, Billerica, MA, USA) at 37°C and 5% CO_2_ for 48 h and cell morphology images were taken every 6 h to monitor cell growth and morphology.

### 2.7. Cell Cycle Detection Using a Ceilometer K2 Dual Fluorescence Cell Analyzer

CNE2 cells with different concentrations of luteolin (0 *μ*M, 20 *μ*M, and 40 *μ*M) and CIS (10 *μ*M) were incubated for 36 h, they were collected and washed twice with precooled PBS. Next, 1 mL of 75% alcohol was added to each tube and the cells were resuspended. They were then placed at -20°C for 15 min and wash twice with precooled PBS, and 100 *μ*L of staining solution (PBS : PI : RNase = 95 : 4 : 1) was added to each tube, and stained for 30 min. After staining, 20 *μ*L of sample was aspirated and added to the Cellometer SD100 Cell Counting Chamber, and placed in the Cellometer K2 Dual Fluorescence Cell Analyzer (Nexcelom, Lawrence, MA, USA) for counting. Each experimental group was triplicate repeated.

### 2.8. Apoptosis Detection by Annexin V-FITC/PI Double Staining

CNE2 cells with different concentrations of luteolin (0 *μ*M, 20 *μ*M, and 40 *μ*M) and CIS (10 *μ*M) were incubated for 36 h, the cells were collected and washed twice with precooled PBS. Then 50 *μ*L of 1 × Binding Buffer was added to each tube to resuspend the cells, along with 5 *μ*L Annexin V-FITC and 5 *μ*L Propidium Iodide (PI; BD Biosciences, San Jose, CA, USA) to each tube. After staining at room temperature for 15 min, 50 *μ*L of 1 × Binding Buffer was added to each tube to stop the reaction, and placed in the Cellometer K2 Dual Fluorescence Cell Analyzer for counting. The experiment was performed in triplicate.

### 2.9. Apoptosis Detection Using Hoechst 33342 Staining

CNE2 cells with different concentrations of luteolin (0 *μ*M, 20 *μ*M, and 40 *μ*M) and CIS (10 *μ*M) for 36 h, the cells were collected and washed twice with precooled PBS. Then 300 *μ*L of Hoechst 33342 (10 *μ*g/mL) (Solarbio) was added to each well, and stained at 37°C for 20 min, then wash twice with PBS, and place in the Cytation™ 5 to capture cell morphology and staining. Image J software was used to analyze the fluorescence intensity, the experiment were performed in triplicate.

### 2.10. Detection of Mitochondrial Membrane Potential (ΔΨ*m*)

CNE2 cells with different concentrations of luteolin (0 *μ*M, 20 *μ*M, and 40 *μ*M) and CIS (10 *μ*M) were incubated for 36 h, the cells were collected and washed twice with precooled PBS and 300 *μ*L JC-1 staining solution (Solarbio) was added to each well, and left to stain at 37°C for 20 min, washed twice with PBS, and place in the Cytation™ 5 to capture cell morphology and staining. Image J software was used to analyze the fluorescence intensity, and each experiment was triplicate repeated.

### 2.11. Western Blot

CNE2 cells with different concentrations of luteolin (0 *μ*M, 20 *μ*M, and 40 *μ*M) and CIS (10 *μ*M) were incubated for 36 h, total protein was extracted and quantified, and 50 *μ*g of protein was used for western blot analysis. The appropriate concentration of separating gel and stacking gel was chosen depending upon the molecular weight of the target protein. The samples were then loaded, onto the SDS-PAGE gel, electrophoresed and the resolved proteins were transferred to a transfer membrane (PVDF), and blocked. Diluted *β*-actin (Cell Signaling Technology, CST, MA, USA), p-ERK1/2 (CST), ERK1/2 (CST), AKT (CST), PI3K (CST), PCNA (Bioss, Woburn, MA, USA), and XIAP (CST) at a dilution ratio of 1 : 1,000 were incubated with the PVDF membrane overnight at 4°C, then wash three times with TBST, for 10 min each. Then, the corresponding diluted goat anti-mouse or goat anti-rabbit secondary antibody was added, and incubated with the PVDF membrane at room temperature for 2 h, washed with TBST three times for 10 min and then scanned on an ODYSSEY CLx Infrared Imager (LICOR, Lincoln, NE, USA) to detect the signal intensities of the immunoreactive bands. Using *β*-actin as an internal reference, the comparative protein expression levels could be calculated, the experiment was performed in triplicate.

### 2.12. Statistical Analysis

All experimental data used SPSS 23.0 statistical software for statistical analysis, and GraphPad Prism 8 software was used for statistical graph drawing, and the experimental data were all expressed by $\bar{x}\pm s$ . The data comparisons between the two groups used an independent sample *t*-test method. Comparisons of measured data between groups used a one-way analysis of variance (one-way ANOVA), and an LSD test was used for multiple comparisons. For uniform variance, and Dunnett's T3 multiple test was used for uneven variances and a value of *P* < 0.05 was considered to be statistically significant.

## 3. Results

### 3.1. Selection of the Bioactive Compounds in *P. cuspidatum* and Prediction of their Possible Molecular Mechanism of Action

#### 3.1.1. Target Filtrate and Construction of Interaction Network

**Figure 1:**
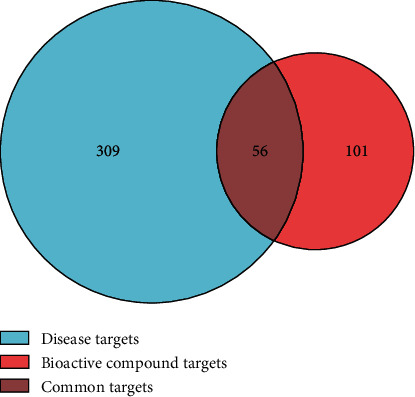
Venn diagram of the bioactive compounds from *P. cuspidatum* and the targets of NPC disease. A total of 365 disease targets (the blue circle) with a correlation score of more than 10 for NPC were obtained from the GeneCards database, and 157 bioactive compound of *P. cuspidatum* targets (the vermilion circle) were retrieved from the TCMSP database, with a total of 56 common targets (the ruby area in the middle) at the intersection of the two.

**Figure 2:**
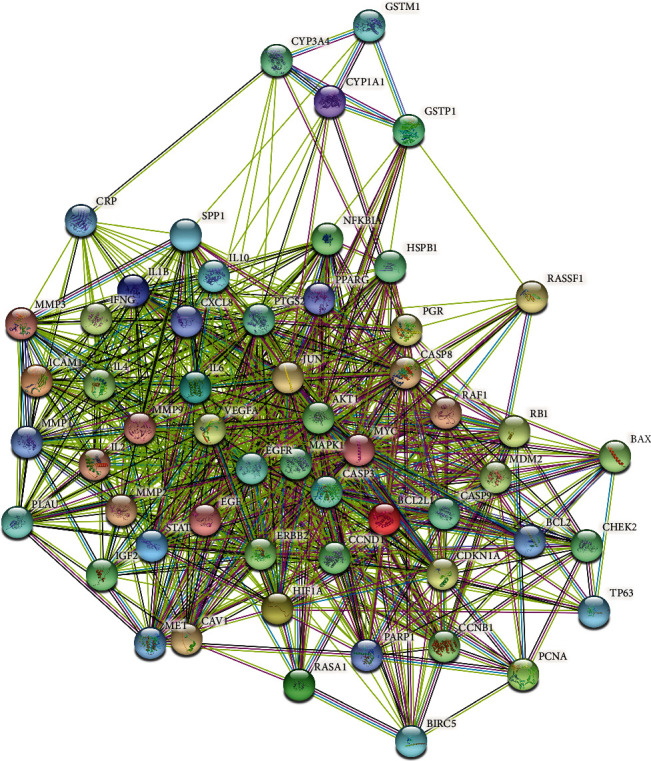
Protein-protein interaction (PPI) diagram.

**Figure 3:**
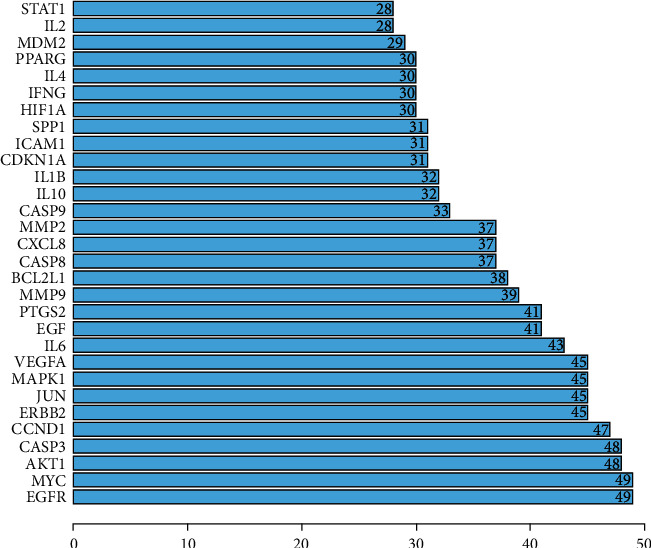
The top 30 common targets with the highest frequency of protein-protein interactions.

**Figure 4:**
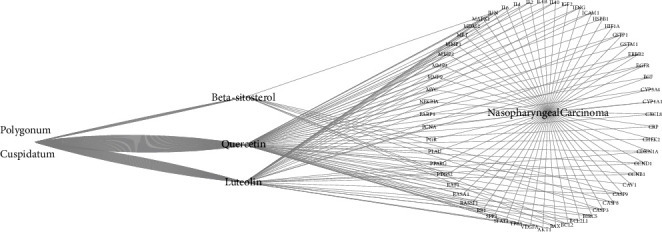
The bioactive compounds from the *P. cuspidatum*-NPC-target network, which contained 56 targets and three compounds, luteolin, quercetin, and beta-sitosterol.

The bioactive compounds derived from *P. cuspidatum* were searched for in the TCMSP database and screened with OB ≥ 30% and DL ≥ 0.18. A total of 10 bioactive compounds were obtained, which were sorted according to the OB value and results are shown in Table 1. We went on to further search targets for the bioactive compounds of *P. cuspidatum* in the TCMSP database and to standardize the names of the identified targets and found a total of 213 targets (including repetitions) for the bioactive compounds. Furthermore, 1,857 disease-related targets for NPC were found in the GeneCards database. Among them, 365 disease targets with a correlation score of more than 10 for NPC and 157 bioactive compounds (without repetition) from *P. cuspidatum* were used to screen out 56 common targets using *R* software, and a Venn diagram was produced (Figure 1). Next, using the STRING platform to input these 56 common targets, we constructed a Protein-protein interaction (PPI) diagram (Figure 2). The top 30 common targets with the highest frequency of protein-protein interactions were EGFR, MYC, AKT1, CASP3, CCND1, and ERBB2, suggesting that these targets are major regulatory proteins for the treatment of NPC (Figure 3). Cytoscape 3.7.2 software was used to construct bioactive compounds from the *P. cuspidatum*-NPC-target network, which contained 56 targets and three compounds, as can be seen in Figure 4. Based on the screening results, we selected luteolin to be used in our *in vitro* experiments. See Tables S1–S5 in the Supplementary Material for comprehensive information of the bioactive compounds of *P. cuspidatum*.

#### 3.1.2. GO Analysis and KEGG Pathway for P. Cuspidatum in the Treatment of NPC

**Figure 5:**
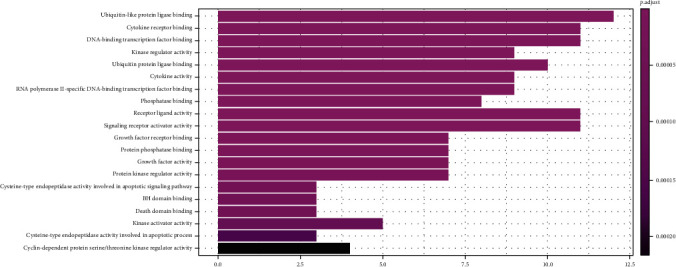
Barplot of GO analysis.

**Figure 6:**
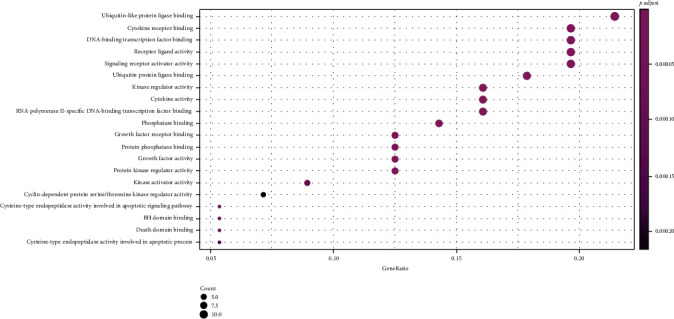
Dotplot of GO analysis.

**Figure 7:**
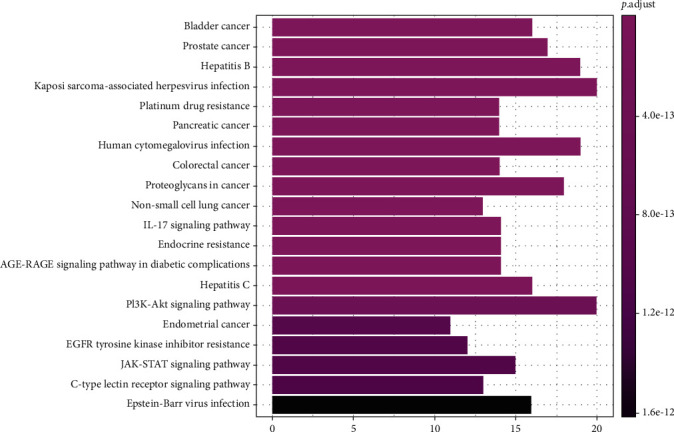
Barplot of the top 20 pathways of KEGG pathway analysis.

**Figure 8:**
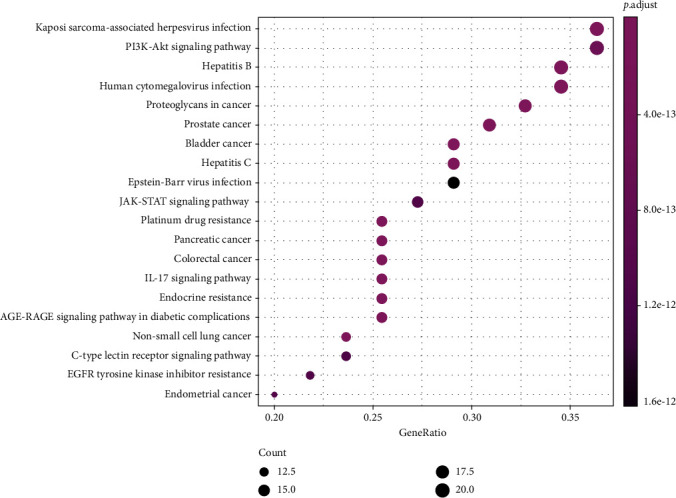
Dotplot of the top 20 pathways of KEGG pathway analysis.

**Figure 9:**
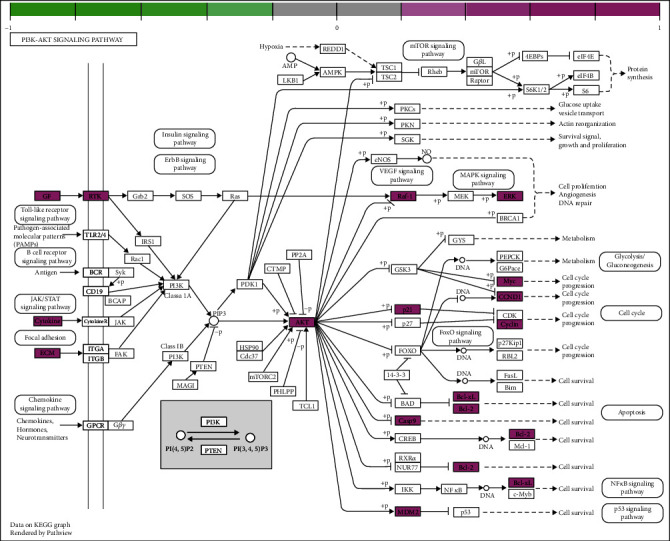
PI3K-AKT signaling pathway as an example and shows the potential targets of *P. cuspidatum* in the treatment of NPC.

The results from the GO analysis showed that the biological processes involved in the drug targets for the treatment of NPC included ubiquitin-like protein ligase binding, cytokine receptor binding, DNA-binding, transcription factor binding, and receptor ligand activity (Figures 5 and 6), the KEGG pathway analysis results are shown in Figures 7 and 8, that shows the KEGG bubble chart, the larger the node and the closer the color is to Magenta, and the higher the significance of the signaling pathway, indicating that the signaling pathway is more important. A total of 136 signaling pathways were obtained through this KEGG analysis.  Table 2 lists the signaling pathways with enriched targets ≥ 15. It also suggested that *P. cuspidatum* may be suitable for the treatment of NPC through multiple regulatory pathways such as the PI3K-AKT, JAK/STAT, MAPK, and C-type lectin receptor signaling pathways.  Figure 9 takes the PI3K-AKT signaling pathway as an example and shows the potential targets of *P. cuspidatum* in the treatment of NPC. Therefore, we selected the PI3K-AKT signaling pathway for subsequent *in vitro* experiments. See Tables S6 and S7 in the Supplementary Material for comprehensive information of common targets of drug compounds and diseases.

#### 3.1.3. Molecular Docking Analysis Predicts the Binding Ability of Luteolin for Its Targets

**Figure 10:**
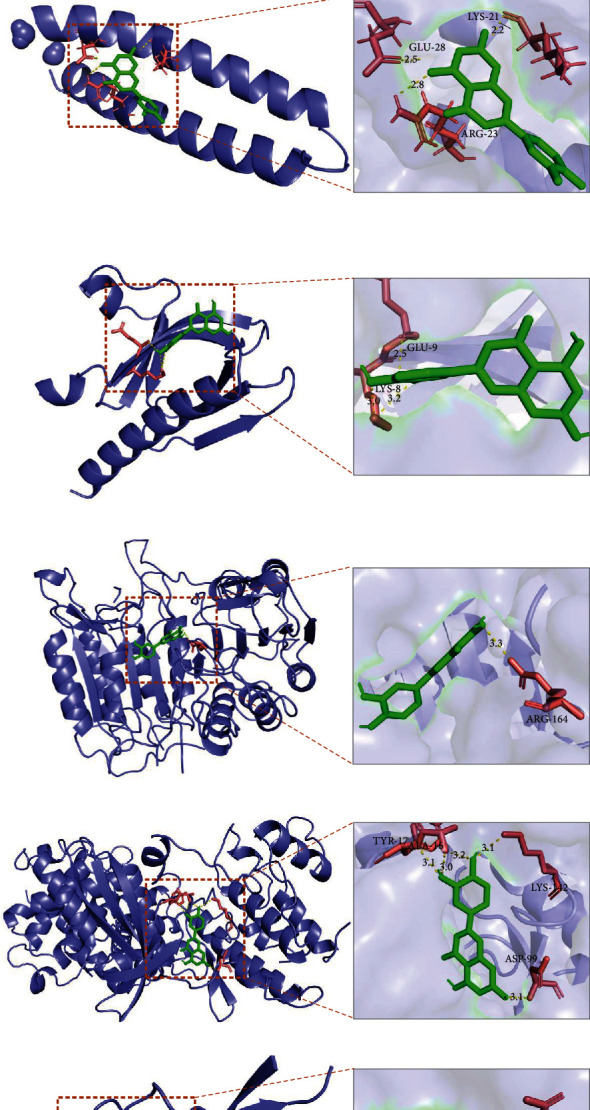
The lowest binding energy interaction mode diagram of luteolin (red frame) with EGFR (a), MYC (b), AKT1 (c), CASP3 (d), CCND1 (e), and ERBB2 (f).

Molecular docking method confirmed that there was an interaction between luteolin and its major targets and using Autodock Vina, we calculated the binding energies for the following major targets: EGFR, MYC, AKT1, CASP3, CCND1, and ERBB2 (Table 3). We then found that their main chemical interactions were represented by hydrogen bonding and *π*-*π* stacking and an interaction mode diagram between the lowest binding energy molecule and its ligand is shown in Figures 10(a)–10(f).

### 3.2. In Vitro Experiments to Verify the Possible Molecular Mechanisms for Luteolin from P. Cuspidatum

#### 3.2.1. Luteolin Inhibits the Proliferation of Human NPC CNE2 Cells

**Figure 11:**
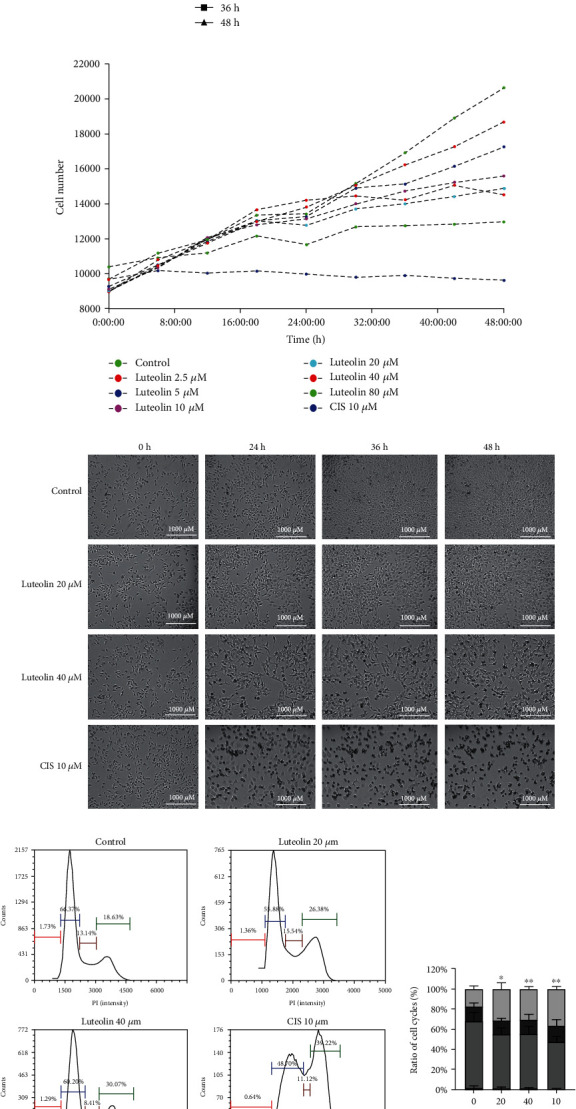
Luteolin inhibits the proliferation of human NPC CNE2 cells. (a) The results from our MTT assay showed that CNE2 cells treated with different concentrations of luteolin (0 *μ*M, 5 *μ*M, 10 *μ*M, 20 *μ*M, 40 *μ*M, and 80 *μ*M) for 24 h, 36 h, and 48 h resulted in a dose-dependent inhibition of growth and survival rates (mean ± SD, *n* = 5). (b) Real-time monitoring of Cytation™ 5 experiments showed that with a 48 h luteolin treatment, CNE2 cell growth was inhibited (*P* < 0.05). (c) Real-time monitoring of Cytation™ 5 experiments showed that with a 48 h luteolin treatment, CNE2 cell morphology changed significantly after drug treatment (bar = 1000 *μ*m, 40×). (d) The cell cycle results showed that when compared to the control group, the cell cycle at the G2/M phase was significantly prolonged after 36 h luteolin treatment (mean ± SD, *n* = 3, comparisons of measured data between groups used one-way ANOVA) vs. control group: ^∗^*P* < 0.05, ^∗∗^*P* < 0.01.

**Figure 12:**
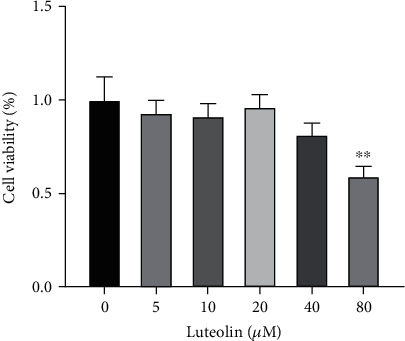
Luteolin inhibits the proliferation of human normal nasopharyngeal epithelial cells NP69 vs. control group: ^∗∗^*P* < 0.01.

The results from our MTT assay showed (Figure 11(a)) that CNE2 cells treated with luteolin, the major bioactive compound derived from *P. cuspidatum*, for 24 h, 36 h, and 48 h resulted in a dose-dependent inhibition of growth and survival rates and this inhibition was also increased with an increased drug action time. After luteolin treatment for 24 h, 36 h and 48 h, the IC_50_ was 77.19 ± 3.36 *μ*M (*F* = 33.65, *P* < 0.0001), 57.10 ± 3.29 *μ*M (*F* = 20.69, *P* < 0.0001), and 39.74 ± 6.77 *μ*M (*F* = 28.86, *P* < 0.0001), respectively. Results from the Cytation™ 5 experiments showed that with a 48 h luteolin treatment, CNE2 cell growth was inhibited (Figure 11(b)) and their cell morphology changed significantly after drug treatment (Figure 11(c)). Cell membrane swelling and rupture began to appear after 24 h of luteolin (20 *μ*M) treatment, and the cell proliferation rate was significantly reduced when compared to the control group. The morphological changes seen in CNE2 cells after luteolin (40 *μ*M) treatment were even more pronounced showing many damaged and detached cells, and cell number and growth were also further suppressed. The cell cycle results showed that when compared to the control group, the cell cycle at the G2/M phase was significantly prolonged after 36 h luteolin treatment (*F* = 12.10, *P* = 0.0024, Figure 11(d)). On the other hand, there was no significant difference in the cell viability rate of normal nasopharyngeal epithelial cells NP69 after different concentrations of luteolin (0 *μ*M, 5 *μ*M, 10 *μ*M, 20 *μ*M, and 40 *μ*M) treatment for 36 h (Figure 12).

#### 3.2.2. Luteolin Promotes Apoptosis in Human NPC CNE2 Cells

**Figure 13:**
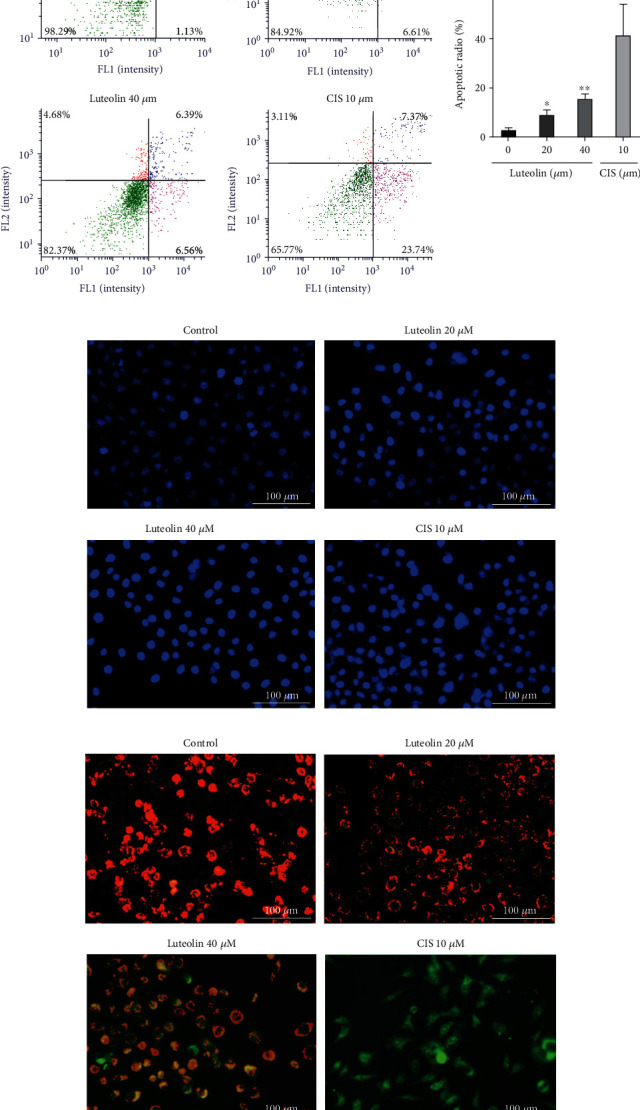
Luteolin promotes apoptosis in human NPC CNE2 cells. (a) Flow cytometry quantitative detection of Annexin V-FITC(+)/PI(-) for early apoptotic cells (lower right) and Annexin V-FITC(+)/PI(+) for apoptotic cells (upper right). Compared to the control group, the cell apoptosis rate was significantly higher after a 36 h luteolin treatment (mean ± SD, *n* = 3, comparisons of measured data between groups used one-way ANOVA). FL1: Annexin V-FITC, FL2: PI. (b) Hoechst 33342 staining showed that cell morphology and fluorescence intensity were changed, when compared with the control group, the blue fluorescence seen in the luteolin group gradually increased (bar = 1000 *μ*m, 200×). (c) JC-1 staining showed that the cells with both green and orange fluorescence increased after luteolin treatment for 36 hours (bar = 1000 *μ*m, 200×). vs. control group: ^∗^*P* < 0.05, ^∗∗^*P* < 0.01.

Quantification of cell fluorescence staining using flow cytometer, the results from Annexin V-FITC/PI fluorescence double staining (Figure 13(a)) revealed that when compared to the control group, the cell apoptosis rate was significantly higher after a 36 h luteolin treatment (*F* = 19.92, *P* = 0.0005). Using Hoechst 33342 staining, we found that cell morphology and fluorescence intensity were changed (Figure 13(b)), when compared with the control group, the blue fluorescence seen in the luteolin group gradually increased, along with the uniform fine mesh, sand-like light blue nuclei seen in the control group, indicating that the level of cell apoptosis was low. After luteolin treatment, however, the blue fluorescence in the nuclei became stronger and brighter, and was accompanied by pyknosis of the nucleus, with blurred edges. Furthermore, the number of cells with the uniform fine mesh sand-like light blue nuclei was significantly reduced, and the overall fluorescence intensity increased along with the gray value of the fluorescent images. The degree value represents the increase in apoptosis rate. The results of mitochondrial membrane potential assay (ΔΨ*m*) showed (Figure 13(c)) that the mitochondrial membrane potential of cells in the control group was higher, and JC-1 dye existed as a polymer on the cell membrane with red fluorescence. The luteolin promoted CNE2 cells apoptosis, the mitochondrial membrane potential decreased, and JC-1 entered the cells as a monomer and showed green fluorescence, meanwhile the red fluorescence gradually weakened, and the cells with both green and orange fluorescence increased.

#### 3.2.3. Luteolin Induces Changes in the Expression of Related Proteins in Human NPC CNE2 Cells

**Figure 14:**
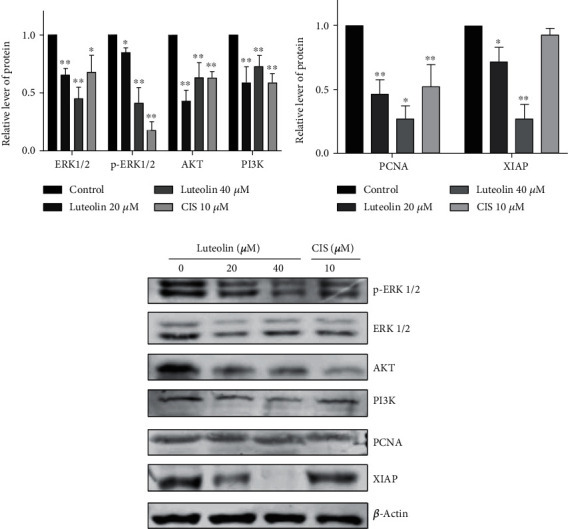
Luteolin induces changes in the expression of related proteins in human NPC CNE2 cells. (a‑c) Western blot results showed when compared to the control group, the expression of p-ERK1/2, ERK1/2, AKT, and PI3K, the related proteins in the PI3K-AKT signaling pathway diagram, and the expression of the proliferation and apoptosis-related proteins PCNA and XIAP expression levels all decreased to varying degrees after luteolin treatment for 36 h (average relative level of protein ± SD, *n* = 3, comparisons of representative data between groups used one-way ANOVA) vs. control group: ^∗^*P* < 0.05, ^∗∗^*P* < 0.01.

According to the results from the KEGG pathway enrichment analysis, we chose to detect the expression levels of proteins related to the PI3K-AKT signaling pathway (Figure 9), and the proliferation and apoptosis-related proteins PCNA and XIAP. According to our western blot results (Figures 14(a)–14(c)), we found that luteolin could alter the expression of p-ERK1/2, ERK1/2, AKT, and PI3K, the related proteins in the PI3K-AKT signaling pathway diagram, and the expression of the proliferation and apoptosis-related proteins PCNA and XIAP. When compared to the control group, p-ERK1/2 (*F* = 71.35, *P* < 0.0001), ERK1/2 (*F* = 18.54, *P* = 0.0006), AKT (*F* = 24.70, *P* = 0.0002), PI3K (*F* = 13.79, *P* = 0.0016), PCNA (*F* = 21.79, *P* = 0.0003), and XIAP (*F* = 47.56, *P* < 0.0001) protein expression levels all decreased to varying degrees after luteolin treatment for 36 h.

## 4. Discussion

Tumorigenesis and the development of NPC is a multistep and multifactorial process, with a complicated pathogenesis. Modern medicine has suggested a variety of mechanisms, by which proliferation, migration, and invasion of NPC can be inhibited, while apoptosis can be promoted. In this study, we conducted a network pharmacology analysis of the Chinese herbal medicine, *P. cuspidatum*. With these biological systems biological network as the goal, we analyzed the bioactive compounds of drugs, drug targets, NPC-related targets, common drug-disease targets, and pathways in these networks. Through *in vitro* experiments, we were verify the anti-NPC effect of the relate targets of PI3K-AKT pathway and the major bioactive compounds found in *P. cuspidatum*.

Network pharmacology can reflect the complex interactional interactions between drug target and biological function and molecular structure [29]. A single-target drug may be effective against a single-molecule, but is likely to be compensated for by other pathways in the body. Network pharmacology observes the intervention and impact of compounds on disease networks through network analysis, and analyzes the effects of bioactive compounds on different nodes of this network, so as to understand the effectiveness of compounds from a systematic perspective. Therefore, we used network pharmacology as the entry point for a preliminarily exploration of the targets and mechanisms of action of compounds derived from the Chinese herbal medicine *P. cuspidatum* in the treatment of NPC. Tumor occurrence and development are related to the interaction of multiple pathways, multiple gene expression profiles, and multiple functional proteins. The distinctive feature of TCM is that it adopts a systematic, holistic view, and dialectical approach to the prevention and treatment of diseases corresponding to the basic characteristics of network pharmacology, such as integrity and systemicity. Network pharmacological analysis can provide reference for the multichannel and multitargeted prevention and treatment of NPC by TCM [30], which is helpful for in-depth discussion of the material basis, mechanism of action, and compatibility principles of *P. cuspidatum*.

We have constructed a network diagram of the bioactive compound of *P. cuspidatum*-NPC-target, which contained 56 common targets and three major compounds. The more bioactive compounds or targets connected, the greater the importance was given to the bioactive compound or target, which contained several key disease targets that appeared more frequently of protein-protein interactions: EGFR, MYC, AKT1, CASP3, CCND1, and ERBB2 (Figure 3) and the major bioactive compounds of *P. cuspidatum* including luteolin, quercetin, and beta-sitosterol. Luteolin may inhibit fat formation and proliferation of nasopharyngeal epithelial cells, and promote cell apoptosis and necrosis, leading to inhibition of tumor growth [31] by the inhibition of the reactivation of Epstein-Barr virus (EBV). This causes a reduced tendency for tumor deterioration by genome instability and cell proliferation, migration, and invasion caused by viral reactivation [32]. |Through enhance protein phosphorylation and proteasome degradation, it leads to downregulation of cyclin D1, and thus inhibits the cell cycle progression of NPC cells in G1 phase and prevents them from entering S phase in a dose-dependent and time-dependent manner. It also eliminates the insulin effect on the Akt/glycogen synthase kinase-3*β*/Cyclin D1 pathway, thereby inhibiting insulin-induced cell proliferation [33], and other ways to inhibit the occurrence and development of nasopharyngeal carcinoma. Quercetin has also been proven to exert its anti-NPC effect by a variety of mechanisms. It can be added to reduce the dose of cisplatin required for the treatment of NPC, to below toxic levels and reduce toxicity-related risk while maintaining or improving efficacy [34]. By inhibiting the expression of vascular endothelial growth factor (VEGF) in NPC cells, it can antagonize the formation of new blood vessels and metastasis of NPC [35]. A recent study has reported that using network pharmacology to analyze the mechanism of TCM *Lei Gong Teng* against NPC, it was shown that bioactive compounds such as beta-sitosterol also existed in *Lei Gong Teng* [36], but other ways of inhibiting NPC have not been reported.

Our study used molecular docking to elucidate the interaction between the ligand luteolin and the key target receptor. Generally, the lower the binding energy of the ligand and receptor, the greater the stability of the binding conformation. If the binding energy is less than -5.0 kcal·mol^−1^, it indicates that the ligand has a good binding ability with the receptor, and if the binding energy is less than -7.0 kcal·mol^−1^, it indicates that the ligand has a strong binding ability with the receptor. Luteolin was docked with six major target proteins, and it was found that it had a strong binding capacity for EGFR, MYC, AKT1, CASP3, CCND1, and ERBB2. Some studies have shown that HPLC-TOF/MS (high performance liquid chromatography time-of-flight mass spectrometry) has been used to clarify the analysis of other bioactive compounds in *P. cuspidatum* herbal, and it has been identified that *P. cuspidatum* does contain luteolin (predicted m/z 463.123 9, measured m/z 463.124 0) [37]. Sun et al. used HSCCC (high-speed countercurrent chromatography) method to successfully separate 4.9 mg of luteolin from 600 g of *P. cuspidatum*(HPLC = 98%). The extracted and purified luteolin from *P. Cuspidatum* was identified by 1H and 13C NMR spectroscopy, and the chemical structure was consistent with that of the purified luteolin standard (HPLC≥98%) [38]. These references confirmed the existence of the compound luteolin in *P. cuspidatum*, confirming that the predicted results of network pharmacology in this study are reliable. Although the content of luteolin in *P. cuspidatum* is very small, compared with the other two bioactive compounds, it has stronger ability to bind to the target and the ability to significantly inhibit the proliferation of nasopharyngeal carcinoma CNE2 cells (Supplementary Material. Figure S1), so luteolin was selected as the *in vitro* cell experiment validated compounds.

We analyzed the common targets through GO analysis and KEGG pathway enrichment analysis, and the results showed that the bioactive compounds of *P. cuspidatum* may involve ubiquitin-like protein ligase binding, cytokine receptor binding, DNA-binding, transcription factor binding, and receptor ligand activity. Moreover, the major bioactive compounds derived from *P. cuspidatum* may inhibit tumor development and destruction through the PI3K-AKT, JAK/STAT, MAPK, C-type lectin receptor signal pathways, and consistent with existing clinical reports [39–42], suggested that the KEGG pathway analysis results can be trusted. However, research looking at treatment methods for NPC remains insufficient. Therefore, we further carried out *in vitro* experiments to verify a role for luteolin on the inhibition of NPC CNE2 cells line via the PI3K-AKT pathway, and furthermore, study its biological mechanism of the inhibition.

*In vitro* cell experiments, our data confirmed that treatment with luteolin at concentrations of 20 *μ*M and 40 *μ*M for 36 h significantly inhibited the proliferation of NPC CNE2 cells and our MTT assay showed that the cell survival rate decreased after treatment. MTT also verified that the concentration of luteolin (0 *μ*M, 5 *μ*M, 10 *μ*M, 20 *μ*M, and 40 *μ*M) used in the *in vitro* experiment had no effect on the cell viability rate of human normal nasopharyngeal NP69 cells. Cytation™ 5 real-time monitoring of cell proliferation confirmed that treatment with luteolin decreased both growth rate and cell number, which were accompanied by changes in cell morphology. Moreover, our study of the cell cycle suggested that luteolin associated with NPC CNE2 cell proliferation by blocking their G2/M phase. Using Annexin V-FITC/PI double staining, Hoechst 33342 staining, and mitochondrial membrane potential measurements (ΔΨ*m*), we were able to infer that luteolin may also inhibit the occurrence and development of NPC by inducing cell apoptosis [26]. Furthermore, western blot results showed that after treatment with luteolin (20 *μ*M and 40 *μ*M) for 36 h, expression of the cell proliferation and apoptosis-related proteins PCNA and XIAP were downregulated, and when compared to the control group, key proteins found in the PI3K-AKT signaling pathway were marked in red, such as p-ERK1/2, ERK1/2, AKT, PI3K, and others. This demonstrated that the PI3K-AKT signaling pathway may play an important role in the inhibitory effect of luteolin on NPC, which is consistent with the predicted results from our network pharmacology analysis. Therefore, our results showed that luteolin may inhibit the proliferation of NPC CNE2 cells and promote their apoptosis through the PI3K-AKT signal pathway.

Although there have been previous studies on the anticancer activity of luteolin or *P. cuspidatum* [43–45], our study is the first to use network pharmacology and experimental verification to preliminarily analyze the material basis and molecular mechanism of *P. cuspidatum* against NPC. Our research ideas and analysis methods are different from other studies. Our study not only confirmed that luteolin inhibits the proliferation of NPC CNE2 cells *in vitro* and promotes apoptosis of CNE2 cells through the PI3K-AKT signaling pathway but also showed that the common drug-disease targets. The enrichment analysis showed that the bioactive compounds of *P. cuspidatum* in the anti-NPC treatment may involve biological processes and signaling pathways, which is a systematic and comprehensive network prediction. These findings may provide key information for the development of herbal medicines, clinical diagnosis and personalized diagnosis, and treatment for NPC. However, our study is only a preliminary exploratory study, and there are still some limitations. Network pharmacology is mainly for prediction and screening, and our in vitro experimental verification part is not comprehensive enough. We have not been able to conduct studies on the NPC CNE2 cell line with the whole plant or luteolin-enriched plants of *P. cuspidatum*, and we have not been able to isolate the luteolin compound in *P. cuspidatum* and compare its activity with the luteolin standard. Moreover, our experimental verification is *in vitro* cell model verification, lacking animal experiments or randomized controlled clinical trials, and more in-depth and systematic research is needed on the anti-nasopharyngeal cancer research of *P. cuspidatum*.

## 5. Conclusions

Here, we have combined network pharmacology methodology with *in vitro* experimental verification to clarify the possible mechanism of action of luteolin, the major bioactive compound found in *P. cuspidatum*, used in the treatment of NPC. The results show that 157 bioactive compounds from *P. cuspidatum* regulated 56 common drug-disease targets, and downregulated PI3K-AKT and other signaling pathways found in NPC. Our data also revealed possible pathways and mechanisms of action of *P. cuspidatum* as being associated with the proliferation of NPC cells and inducing their apoptosis. However, research on the pharmacological mechanism and clinical application of *P. cuspidatum* and luteolin remains unclear; therefore, more in-depth and innovative studies on *P. cuspidatum* and luteolin are needed to confirm their efficacy and ultimately drug safety.

## Figures and Tables

**Table 1:** Basic parameters of the major bioactive compounds of *P. cuspidatum*.

Number	Compound	OB (%)	DL
MOL013287	Physovenine	106.21	0.19
MOL013288	Picralinal	58.01	0.75
MOL000492	(+)-catechin	54.83	0.24
MOL002268	Rhein	47.07	0.28
MOL000098	Quercetin	46.43	0.28
MOL002280	Torachrysone-8-O-beta-D-(6′-oxayl)-glucoside	43.02	0.74
MOL002259	Physciondiglucoside	41.65	0.63
MOL000358	Beta-sitosterol	36.91	0.75
MOL000006	Luteolin	36.16	0.25
MOL013281	6,8-Dihydroxy-7-methoxyxanthone	35.83	0.21

**Table 2:** The signaling pathways with enriched targets ≥ 15 from KEGG analysis.

ID	Description	geneID	Count
hsa05167	Kaposi sarcoma-associated herpesvirus infection	PTGS2/BAX/CASP9/JUN/CASP3/CASP8/AKT1/VEGFA/CCND1/CDKN1A/MAPK1/RB1/IL6/NFKBIA/ICAM1/RAF1/HIF1A/STAT1/MYC/CXCL8	20
hsa04151	PI3K-Akt signaling pathway	BCL2/CASP9/EGFR/AKT1/VEGFA/CCND1/BCL2L1/CDKN1A/MAPK1/IL6/MDM2/ERBB2/IL2/IL4/MET/EGF/RAF1/MYC/SPP1/IGF2	20
hsa05161	Hepatitis B	BCL2/BAX/CASP9/JUN/CASP3/CASP8/AKT1/CDKN1A/MMP9/MAPK1/RB1/IL6/NFKBIA/PCNA/BIRC5/RAF1/STAT1/MYC/CXCL8	19
hsa05163	Human cytomegalovirus infection	PTGS2/BAX/CASP9/CASP3/CASP8/EGFR/AKT1/VEGFA/CCND1/CDKN1A/MAPK1/RB1/IL6/NFKBIA/MDM2/RAF1/MYC/IL1B/CXCL8	19
hsa05205	Proteoglycans in cancer	CASP3/EGFR/AKT1/VEGFA/CCND1/CDKN1A/MMP2/MMP9/MAPK1/MDM2/ERBB2/MET/PLAU/RAF1/HIF1A/CAV1/MYC/IGF2	18
hsa05215	Prostate cancer	BCL2/CASP9/EGFR/AKT1/CCND1/CDKN1A/MMP9/MAPK1/RB1/NFKBIA/MDM2/ERBB2/GSTP1/MMP3/PLAU/EGF/RAF1	17
hsa05206	MicroRNAs in cancer	PTGS2/BCL2/CASP3/EGFR/VEGFA/CCND1/CDKN1A/MMP9/MAPK1/TP63/MDM2/ERBB2/MET/PLAU/RAF1/MYC/RASSF1	17
hsa05219	Bladder cancer	EGFR/VEGFA/CCND1/CDKN1A/MMP2/MMP9/MAPK1/RB1/MDM2/MMP1/ERBB2/EGF/RAF1/MYC/CXCL8/RASSF1	16
hsa05160	Hepatitis C	BAX/CASP9/CASP3/CASP8/EGFR/AKT1/CCND1/CDKN1A/MAPK1/RB1/NFKBIA/IFNG/EGF/RAF1/STAT1/MYC	16
hsa05169	Epstein-Barr virus infection	BCL2/BAX/CASP9/JUN/CASP3/CASP8/AKT1/CCND1/CDKN1A/RB1/IL6/NFKBIA/MDM2/ICAM1/STAT1/MYC	16
hsa05165	Human papillomavirus infection	PTGS2/BAX/CASP3/CASP8/EGFR/AKT1/VEGFA/CCND1/CDKN1A/MAPK1/RB1/MDM2/EGF/RAF1/STAT1/SPP1	16
hsa04630	JAK-STAT signaling pathway	BCL2/EGFR/AKT1/CCND1/BCL2L1/CDKN1A/IL10/IL6/IL2/IFNG/IL4/EGF/RAF1/STAT1/MYC	15
hsa04010	MAPK signaling pathway	JUN/CASP3/EGFR/AKT1/VEGFA/MAPK1/ERBB2/MET/EGF/RAF1/MYC/IL1B/HSPB1/IGF2/RASA1	15

**Table 3:** Molecular docking information of luteolin with major targets.

Target	Uniport ID	PDBID	Affinity(kcal/mol)
EGFR	P00533	1IVO	-8.3
MYC	P01106	1A93	-8.4
AKT1	P31749	1H10	-6.3
CASP3	P42574	1CP3	-7.7
CCND1	P24385	2 W96	-8.2
ERBB2	P04626	1MFG	-8.4

## Data Availability

The datasets used and analyzed during the current study are available from the corresponding author on reasonable request.

## Supplementary Materials

Supplementary Materials Table S1: Basic information of the bioactive compounds of P. cuspidatum. Table S2: The targets for the bioactive compounds of P. cuspidatum in the TCMSP database. Table S3: The standard names of targets for the bioactive compounds of P. cuspidatum. Table S4: Basic information of the disease related targets for NPC. Table S5: The common targets of disease targets for NPC and bioactive compounds from P. cuspidatum. Table S6: GO analysis of common targets of drug compounds and diseases through the DAVID website. Table S7: KEGG pathway analysis of common targets of drug compounds and diseases through the DAVID website. Figure S1: Effect of different bioactive compounds on the survival rate of CNE2 cells in NPC.
