# Supplementary material for: Luteolin Isolated from Polygonum cuspidatum Is a Potential Compound against Nasopharyngeal Carcinoma
Source: Biomed Res Int. 2022 Dec 23;2022:9740066. doi: 10.1155/2022/9740066 (PMC9803567; doi:10.1155/2022/9740066)
Supplement: Supplementary Materials — Table S1: Basic information of the bioactive compounds of P. cuspidatum. Table S2: The targets for the bioactive compounds of P. cuspidatum in the TCMSP database. Table S3: The standard names of targets for the bioactive compounds of P. cuspidatum. Table S4: Basic information of the disease related targets for NPC. Table S5: The common targets of disease targets for NPC and bioactive compounds from P. cuspidatum. Table S6: GO analysis of common targets of drug compounds and diseases through the DAVID website. Table S7: KEGG pathway analysis of common targets of drug compounds and diseases through the DAVID website. Figure S1: Effect of different bioactive compounds on the survival rate of CNE2 cells in NPC. [file 9740066.f1.zip › Table 1S Basic information of the bioactive compounds of P. cuspidatum.docx]

| MW | AlogP | Hdon | Hacc | OB(%) | Caco-2 | BBB | DL | [FASA-](https://tcmsp-e.com/portal/search?st=hn&sst=hi&qw=Coptidis%20Rhizoma) | HL |
| --- | --- | --- | --- | --- | --- | --- | --- | --- | --- |
| 272.27 | 2.3 | 3 | 5 | 42.36 | 0.38 | -0.48 | 0.21 | 0.41 | 16.83 |
| 302.3 | 2.28 | 3 | 6 | 36.63 | 0.43 | -0.32 | 0.27 | 0.28 | 16.12 |
| 302.3 | 2.28 | 3 | 6 | 74.24 | 0.37 | -0.43 | 0.26 | 0.31 | 16.85 |
| 328.34 | 2.82 | 1 | 6 | 65.82 | 0.85 | 0.07 | 0.33 | 0.22 | 16.41 |
| 298.31 | 2.84 | 1 | 5 | 43.72 | 0.96 | 0.22 | 0.25 | 0.27 | 16.77 |
| 268.28 | 2.85 | 1 | 4 | 37.27 | 0.91 | 0.16 | 0.2 | 0.35 | 17.24 |
| 426.8 | 7.55 | 1 | 1 | 38.69 | 1.45 | 1.16 | 0.78 | 0.23 | 5.41 |
| 446.39 | 0.64 | 6 | 11 | 40.12 | -0.85 | -1.74 | 0.75 | 0.36 | 17.36 |
| 400.76 | 7.63 | 1 | 1 | 37.58 | 1.34 | 0.98 | 0.71 | 0.21 | 4.63 |
| 386.73 | 7.38 | 1 | 1 | 37.87 | 1.43 | 1.13 | 0.68 | 0.2 | 4.52 |
| 414.79 | 8.08 | 1 | 1 | 36.91 | 1.32 | 0.99 | 0.75 | 0.23 | 5.36 |
| 344.34 | 2.55 | 2 | 7 | 37.94 | 0.65 | -0.13 | 0.37 | 0.21 | 16.25 |
| 456.83 | 8.46 | 0 | 2 | 40.39 | 1.39 | 1.11 | 0.85 | 0.22 | 6.34 |
| 454.81 | 8.02 | 0 | 2 | 46.44 | 1.41 | 1.06 | 0.86 | 0.21 | 6.77 |
| 394.75 | 8.43 | 0 | 0 | 45.03 | 1.9 | 1.81 | 0.71 | 0.24 | 6.21 |
| 412.77 | 7.64 | 1 | 1 | 43.83 | 1.44 | 1 | 0.76 | 0.22 | 5.57 |
| 284.28 | 2.59 | 2 | 5 | 30.68 | 0.79 | 0.04 | 0.23 | 0.32 | 17.75 |
| 300.28 | 2.32 | 3 | 6 | 30.97 | 0.48 | -0.49 | 0.27 | 0.28 | 16.44 |
| 412.77 | 8.18 | 0 | 1 | 36.08 | 1.46 | 1.22 | 0.76 | 0.25 | 5.49 |
| 270.25 | 2.33 | 3 | 5 | 33.52 | 0.63 | -0.05 | 0.21 | 0.36 | 16.25 |
| 288.27 | 2.03 | 4 | 6 | 33.23 | 0.27 | -0.27 | 0.24 | 0.34 | 15.67 |
| 328.34 | 2.82 | 1 | 6 | 49.07 | 0.86 | -0.03 | 0.33 | 0.21 | 15.87 |
| 330.31 | 2.01 | 3 | 7 | 47.14 | 0.53 | -0.32 | 0.34 | 0.25 | 13.54 |
| 414.79 | 8.08 | 1 | 1 | 36.91 | 1.32 | 0.87 | 0.75 | 0.22 | 5.37 |
| 288.27 | 2.03 | 4 | 6 | 71.79 | 0.17 | -0.54 | 0.24 | 0.38 | 15.81 |
| 414.79 | 8.08 | 1 | 1 | 36.91 | 1.32 | 0.87 | 0.75 | 0 | 5.08 |
| 286.25 | 2.07 | 4 | 6 | 36.16 | 0.19 | -0.84 | 0.25 | 0.39 | 15.94 |
| 298.31 | 2.84 | 1 | 5 | 44.09 | 1.01 | 0.54 | 0.25 | 0.26 | 17.02 |
| 302.25 | 1.5 | 5 | 7 | 46.43 | 0.05 | -0.77 | 0.28 | 0.38 | 14.4 |
