# Supplementary material for: Luteolin Isolated from Polygonum cuspidatum Is a Potential Compound against Nasopharyngeal Carcinoma
Source: Biomed Res Int. 2022 Dec 23;2022:9740066. doi: 10.1155/2022/9740066 (PMC9803567; doi:10.1155/2022/9740066)
Supplement: Supplementary Materials — Table S1: Basic information of the bioactive compounds of P. cuspidatum. Table S2: The targets for the bioactive compounds of P. cuspidatum in the TCMSP database. Table S3: The standard names of targets for the bioactive compounds of P. cuspidatum. Table S4: Basic information of the disease related targets for NPC. Table S5: The common targets of disease targets for NPC and bioactive compounds from P. cuspidatum. Table S6: GO analysis of common targets of drug compounds and diseases through the DAVID website. Table S7: KEGG pathway analysis of common targets of drug compounds and diseases through the DAVID website. Figure S1: Effect of different bioactive compounds on the survival rate of CNE2 cells in NPC. [file 9740066.f1.zip › Table 2S The targets for the bioactive compounds of P. cuspidatum in the TCMSP database.docx]

| Mol ID | Molecule Name | Target Name | Source | status |
| --- | --- | --- | --- | --- |
| MOL000105 | protocatechuic acid | Prostaglandin G/H synthase 1 | DrugBank |  |
| MOL000105 | protocatechuic acid | Arachidonate 5-lipoxygenase | DrugBank |  |
| MOL000105 | protocatechuic acid | Prostaglandin G/H synthase 2 | DrugBank |  |
| MOL000105 | protocatechuic acid | Amine oxidase [flavin-containing] B | DrugBank |  |
| MOL000105 | protocatechuic acid | Lysozyme | DrugBank |  |
| MOL000105 | protocatechuic acid | Nicotinate-nucleotide--dimethylbenzimidazole phosphoribosyltransferase | DrugBank |  |
| MOL000105 | protocatechuic acid | Alcohol dehydrogenase 1C | DrugBank |  |
| MOL000105 | protocatechuic acid | Trypsin-3 | DrugBank |  |
| MOL000105 | protocatechuic acid | Protein kinase C alpha type | N/A | validated |
| MOL000105 | protocatechuic acid | Protein kinase C beta type | N/A | validated |
| MOL000105 | protocatechuic acid | Protein kinase C gamma type | N/A | validated |
| MOL000105 | protocatechuic acid | Maltase-glucoamylase, intestinal | DrugBank | validated |
| MOL000105 | protocatechuic acid | Protein kinase C zeta type | N/A | validated |
| MOL011783 | Vananote | Sodium-dependent noradrenaline transporter | DrugBank |  |
| MOL011783 | Vananote | Sodium-dependent dopamine transporter | DrugBank |  |
| MOL011783 | Vananote | Amine oxidase [flavin-containing] B | DrugBank |  |
| MOL011783 | Vananote | Amine oxidase [flavin-containing] A | DrugBank |  |
| MOL011783 | Vananote | Lysozyme | DrugBank |  |
| MOL001229 | cis-resveratrol | Prostaglandin G/H synthase 1 | DrugBank |  |
| MOL001229 | cis-resveratrol | Prostaglandin G/H synthase 2 | DrugBank |  |
| MOL001229 | cis-resveratrol | Heat shock protein HSP 90 | DrugBank |  |
| MOL001229 | cis-resveratrol | Amine oxidase [flavin-containing] B | DrugBank |  |
| MOL001229 | cis-resveratrol | mRNA of PKA Catalytic Subunit C-alpha | DrugBank |  |
| MOL001229 | cis-resveratrol | Nuclear receptor coactivator 2 | DrugBank |  |
| MOL001229 | cis-resveratrol | cAMP-dependent protein kinase inhibitor alpha | DrugBank |  |
| MOL001229 | cis-resveratrol | NRH dehydrogenase [quinone] 2 | DrugBank |  |
| MOL001229 | cis-resveratrol | Alcohol dehydrogenase 1B | DrugBank |  |
| MOL001229 | cis-resveratrol | Alcohol dehydrogenase 1C | DrugBank |  |
| MOL001229 | cis-resveratrol | Cytochrome P450-cam | DrugBank |  |
| MOL001229 | cis-resveratrol | Lysozyme | DrugBank |  |
| MOL012443 | Ambrettolid | Gamma-aminobutyric acid receptor subunit alpha-1 | DrugBank |  |
| MOL012744 | resveratrol | Prostaglandin G/H synthase 1 | DrugBank | validated |
| MOL012744 | resveratrol | Prostaglandin G/H synthase 2 | DrugBank | validated |
| MOL012744 | resveratrol | Heat shock protein HSP 90 | DrugBank |  |
| MOL012744 | resveratrol | Amine oxidase [flavin-containing] B | DrugBank |  |
| MOL012744 | resveratrol | mRNA of PKA Catalytic Subunit C-alpha | DrugBank |  |
| MOL012744 | resveratrol | Nuclear receptor coactivator 2 | DrugBank |  |
| MOL012744 | resveratrol | Carbonic anhydrase II | DrugBank |  |
| MOL012744 | resveratrol | Transcription factor p65 | N/A | validated |
| MOL012744 | resveratrol | Signal transducer and activator of transcription 3 | N/A | validated |
| MOL012744 | resveratrol | RAC-alpha serine/threonine-protein kinase | N/A | validated |
| MOL012744 | resveratrol | Vascular endothelial growth factor A | DrugBank | validated |
| MOL012744 | resveratrol | G1/S-specific cyclin-D1 | N/A | validated |
| MOL012744 | resveratrol | Apoptosis regulator Bcl-2 | DrugBank | validated |
| MOL012744 | resveratrol | Bcl-2-like protein 1 | N/A | validated |
| MOL012744 | resveratrol | Proto-oncogene c-Fos | N/A | validated |
| MOL012744 | resveratrol | Cyclin-dependent kinase inhibitor 1 | N/A | validated |
| MOL012744 | resveratrol | Eukaryotic translation initiation factor 6 | N/A | validated |
| MOL012744 | resveratrol | Apoptosis regulator BAX | N/A | validated |
| MOL012744 | resveratrol | Caspase-9 | N/A | validated |
| MOL012744 | resveratrol | Urokinase-type plasminogen activator | DrugBank | validated |
| MOL012744 | resveratrol | 72 kDa type IV collagenase | DrugBank | validated |
| MOL012744 | resveratrol | Matrix metalloproteinase-9 | N/A | validated |
| MOL012744 | resveratrol | Mitogen-activated protein kinase 3 | DrugBank | validated |
| MOL012744 | resveratrol | Mitogen-activated protein kinase 1 | DrugBank | validated |
| MOL012744 | resveratrol | Interleukin-10 | N/A | validated |
| MOL012744 | resveratrol | Cell division protein kinase 4 | DrugBank | validated |
| MOL012744 | resveratrol | Tumor necrosis factor | DrugBank | validated |
| MOL012744 | resveratrol | Transcription factor AP-1 | DrugBank | validated |
| MOL012744 | resveratrol | Interleukin-6 | DrugBank | validated |
| MOL012744 | resveratrol | Cell division protein kinase 6 | DrugBank | validated |
| MOL012744 | resveratrol | Activator of 90 kDa heat shock protein ATPase homolog 1 | N/A | validated |
| MOL012744 | resveratrol | Caspase-3 | N/A | validated |
| MOL012744 | resveratrol | Cellular tumor antigen p53 | DrugBank | validated |
| MOL012744 | resveratrol | Mitogen-activated protein kinase 8 | DrugBank | validated |
| MOL012744 | resveratrol | NF-kappa-B inhibitor alpha | N/A | validated |
| MOL012744 | resveratrol | Ornithine decarboxylase | DrugBank | validated |
| MOL012744 | resveratrol | Xanthine dehydrogenase/oxidase | DrugBank | validated |
| MOL012744 | resveratrol | Bcl-2 homologous antagonist/killer | N/A | validated |
| MOL012744 | resveratrol | Bcl-2-binding component 3 | N/A | validated |
| MOL012744 | resveratrol | Superoxide dismutase [Cu-Zn] | DrugBank | validated |
| MOL012744 | resveratrol | Catalase | DrugBank | validated |
| MOL012744 | resveratrol | Protein kinase C alpha type | N/A | validated |
| MOL012744 | resveratrol | Telomerase protein component 1 | N/A | validated |
| MOL012744 | resveratrol | Hypoxia-inducible factor 1-alpha | N/A | validated |
| MOL012744 | resveratrol | Insulin-like growth factor 1 receptor | N/A | validated |
| MOL012744 | resveratrol | Signal transducer and activator of transcription 1-alpha/beta | N/A | validated |
| MOL012744 | resveratrol | Protein CBFA2T1 | N/A | validated |
| MOL012744 | resveratrol | Probable E3 ubiquitin-protein ligase HERC5 | N/A | validated |
| MOL012744 | resveratrol | Cell division control protein 2 homolog | DrugBank | validated |
| MOL012744 | resveratrol | Endothelin-1 | N/A | validated |
| MOL012744 | resveratrol | 78 kDa glucose-regulated protein | DrugBank | validated |
| MOL012744 | resveratrol | Forkhead box protein O1 | N/A | validated |
| MOL012744 | resveratrol | Heparin-binding growth factor 2 | DrugBank | validated |
| MOL012744 | resveratrol | Peroxisome proliferator-activated receptor gamma | N/A | validated |
| MOL012744 | resveratrol | Catenin beta-1 | N/A | validated |
| MOL012744 | resveratrol | Myc proto-oncogene protein | N/A | validated |
| MOL012744 | resveratrol | Tissue factor | DrugBank | validated |
| MOL012744 | resveratrol | Gap junction alpha-1 protein | DrugBank | validated |
| MOL012744 | resveratrol | Cytochrome P450 1A1 | N/A | validated |
| MOL012744 | resveratrol | Intercellular adhesion molecule 1 | N/A | validated |
| MOL012744 | resveratrol | Interleukin-1 beta | DrugBank | validated |
| MOL012744 | resveratrol | C-C motif chemokine 2 | DrugBank | validated |
| MOL012744 | resveratrol | E-selectin | DrugBank | validated |
| MOL012744 | resveratrol | Vascular cell adhesion protein 1 | DrugBank | validated |
| MOL012744 | resveratrol | Protein kinase C delta type | N/A | validated |
| MOL012744 | resveratrol | Interleukin-8 | N/A | validated |
| MOL012744 | resveratrol | Induced myeloid leukemia cell differentiation protein Mcl-1 | N/A | validated |
| MOL012744 | resveratrol | Breast cancer type 1 susceptibility protein | N/A | validated |
| MOL012744 | resveratrol | X-ray repair cross-complementing protein 6 | N/A | validated |
| MOL012744 | resveratrol | Superoxide dismutase [Mn], mitochondrial | N/A | validated |
| MOL012744 | resveratrol | Protein kinase C beta type | N/A | validated |
| MOL012744 | resveratrol | Platelet endothelial cell adhesion molecule | N/A | validated |
| MOL012744 | resveratrol | Baculoviral IAP repeat-containing protein 5 | N/A | validated |
| MOL012744 | resveratrol | Dual oxidase 2 | N/A | validated |
| MOL012744 | resveratrol | Nitric oxide synthase, endothelial | N/A | validated |
| MOL012744 | resveratrol | Tyrosine-protein phosphatase non-receptor type 1 | N/A | validated |
| MOL012744 | resveratrol | G1/S-specific cyclin-D2 | N/A | validated |
| MOL012744 | resveratrol | Bcl-2-related protein A1 | N/A | validated |
| MOL012744 | resveratrol | Multidrug resistance protein 1 | DrugBank | validated |
| MOL012744 | resveratrol | Hepatocyte growth factor | DrugBank | validated |
| MOL012744 | resveratrol | Transforming growth factor beta-1 | N/A | validated |
| MOL012744 | resveratrol | Estrogen sulfotransferase | DrugBank | validated |
| MOL012744 | resveratrol | Maltase-glucoamylase, intestinal | DrugBank | validated |
| MOL012744 | resveratrol | Cytochrome P450 1B1 | N/A | validated |
| MOL012744 | resveratrol | G2/mitotic-specific cyclin-B1 | N/A | validated |
| MOL012744 | resveratrol | DNA damage-inducible transcript 3 protein | N/A | validated |
| MOL012744 | resveratrol | Tissue-type plasminogen activator | DrugBank | validated |
| MOL012744 | resveratrol | Phosphatidylinositol-3,4,5-trisphosphate 3-phosphatase and dual-specificity protein phosphatase PTEN | N/A | validated |
| MOL012744 | resveratrol | Cell division protein kinase 7 | DrugBank | validated |
| MOL012744 | resveratrol | Interleukin-1 alpha | N/A | validated |
| MOL012744 | resveratrol | Myeloperoxidase | DrugBank | validated |
| MOL012744 | resveratrol | Tumor necrosis factor receptor superfamily member 10B | N/A | validated |
| MOL012744 | resveratrol | Cytochrome P450 19A1 | N/A | validated |
| MOL012744 | resveratrol | ATP-binding cassette sub-family G member 2 | N/A | validated |
| MOL012744 | resveratrol | Nuclear factor erythroid 2-related factor 2 | N/A | validated |
| MOL012744 | resveratrol | NAD(P)H dehydrogenase [quinone] 1 | DrugBank | validated |
| MOL012744 | resveratrol | Baculoviral IAP repeat-containing protein 4 | N/A | validated |
| MOL012744 | resveratrol | Aryl hydrocarbon receptor | DrugBank | validated |
| MOL012744 | resveratrol | 5'-AMP-activated protein kinase subunit gamma-2 | N/A | validated |
| MOL012744 | resveratrol | Solute carrier family 2, facilitated glucose transporter member 4 | N/A | validated |
| MOL012744 | resveratrol | Peroxisome proliferator-activated receptor alpha | N/A | validated |
| MOL012744 | resveratrol | C-reactive protein | N/A | validated |
| MOL012744 | resveratrol | Serum paraoxonase/arylesterase 1 | DrugBank | validated |
| MOL012744 | resveratrol | T-lymphocyte activation antigen CD80 | DrugBank | validated |
| MOL012744 | resveratrol | G1/S-specific cyclin-E1 | N/A | validated |
| MOL012744 | resveratrol | G1/S-specific cyclin-E2 | N/A | validated |
| MOL012744 | resveratrol | Basal cell adhesion molecule | N/A | validated |
| MOL012744 | resveratrol | NAD-dependent deacetylase sirtuin-1 | N/A | validated |
| MOL012744 | resveratrol | CREB/ATF bZIP transcription factor | N/A | validated |
| MOL012744 | resveratrol | High affinity nerve growth factor receptor | DrugBank | validated |
| MOL012744 | resveratrol | Krueppel-like factor 10 | N/A | validated |
| MOL012744 | resveratrol | Apoptotic protease-activating factor 1 | DrugBank | validated |
| MOL012744 | resveratrol | Canalicular multispecific organic anion transporter 2 | N/A | validated |
| MOL012744 | resveratrol | Sterol regulatory element-binding protein 1 | N/A | validated |
| MOL012744 | resveratrol | Adiponectin receptor protein 1 | N/A | validated |
| MOL012744 | resveratrol | Adiponectin receptor protein 2 | N/A | validated |
| MOL012744 | resveratrol | Tyrosine-protein kinase JAK1 | N/A | validated |
| MOL012744 | resveratrol | Type-1 angiotensin II receptor | DrugBank | validated |
| MOL012744 | resveratrol | Insulin receptor substrate 1 | N/A | validated |
| MOL012744 | resveratrol | CASP8 and FADD-like apoptosis regulator | N/A | validated |
| MOL012744 | resveratrol | Lengsin | N/A | validated |
| MOL012744 | resveratrol | Pygopus homolog 1 | N/A | validated |
| MOL012744 | resveratrol | Interleukin-17B | N/A | validated |
| MOL012744 | resveratrol | NAD-dependent deacetylase sirtuin-2 | N/A | validated |
| MOL012744 | resveratrol | C5a anaphylatoxin chemotactic receptor | N/A | validated |
| MOL012744 | resveratrol | Serine/threonine-protein kinase mTOR | DrugBank | validated |
| MOL012744 | resveratrol | C-C chemokine receptor type 2 | N/A | validated |
| MOL012744 | resveratrol | Eukaryotic translation initiation factor 2 subunit 1 | N/A | validated |
| MOL012744 | resveratrol | Prostaglandin E synthase | N/A | validated |
| MOL012744 | resveratrol | Phorbol-12-myristate-13-acetate-induced protein 1 | N/A | validated |
| MOL012744 | resveratrol | Bcl-2-like protein 11 | N/A | validated |
| MOL012744 | resveratrol | Tumor necrosis factor ligand superfamily member 10 | N/A | validated |
| MOL012744 | resveratrol | Tumor necrosis factor receptor superfamily member 10A | N/A | validated |
| MOL012744 | resveratrol | 40S ribosomal protein S6 | N/A | validated |
| MOL012744 | resveratrol | GTP cyclohydrolase 1 | N/A | validated |
| MOL012744 | resveratrol | Baculoviral IAP repeat-containing protein 3 | N/A | validated |
| MOL012744 | resveratrol | TNF receptor-associated factor 2 | N/A | validated |
| MOL012744 | resveratrol | Cell division control protein 42 homolog | N/A | validated |
| MOL012744 | resveratrol | Basigin | N/A | validated |
| MOL012744 | resveratrol | Collagen alpha-1(II) chain | DrugBank | validated |
| MOL012744 | resveratrol | Integrin beta-1 | DrugBank | validated |
| MOL012744 | resveratrol | T-cell-specific surface glycoprotein CD28 | N/A | validated |
| MOL012744 | resveratrol | Oxysterols receptor LXR-alpha | DrugBank | validated |
| MOL012744 | resveratrol | Pappalysin-1 | N/A | validated |
| MOL012744 | resveratrol | SPARC | N/A | validated |
| MOL012744 | resveratrol | Breast cancer type 2 susceptibility protein | N/A | validated |
| MOL012744 | resveratrol | Alpha- and gamma-adaptin-binding protein p34 | N/A | validated |
| MOL012744 | resveratrol | Serine/threonine-protein kinase D1 | N/A | validated |
| MOL012744 | resveratrol | Protransforming growth factor alpha | N/A | validated |
| MOL012744 | resveratrol | CD320 antigen | N/A | validated |
| MOL012744 | resveratrol | Transforming growth factor beta-2 | N/A | validated |
| MOL013280 | (-)-3-Hydroxy-4-methoxy-8-9-methylenedioxy pterocarpan | Prostaglandin G/H synthase 1 | DrugBank |  |
| MOL013280 | (-)-3-Hydroxy-4-methoxy-8-9-methylenedioxy pterocarpan | Sodium channel protein type 5 subunit alpha | DrugBank |  |
| MOL013280 | (-)-3-Hydroxy-4-methoxy-8-9-methylenedioxy pterocarpan | Prostaglandin G/H synthase 2 | DrugBank |  |
| MOL013280 | (-)-3-Hydroxy-4-methoxy-8-9-methylenedioxy pterocarpan | Carbonic anhydrase II | DrugBank |  |
| MOL013280 | (-)-3-Hydroxy-4-methoxy-8-9-methylenedioxy pterocarpan | Retinoic acid receptor RXR-alpha | DrugBank |  |
| MOL013280 | (-)-3-Hydroxy-4-methoxy-8-9-methylenedioxy pterocarpan | Acetylcholinesterase | DrugBank |  |
| MOL013280 | (-)-3-Hydroxy-4-methoxy-8-9-methylenedioxy pterocarpan | Alpha-1B adrenergic receptor | DrugBank |  |
| MOL013280 | (-)-3-Hydroxy-4-methoxy-8-9-methylenedioxy pterocarpan | Alpha-1D adrenergic receptor | DrugBank |  |
| MOL013280 | (-)-3-Hydroxy-4-methoxy-8-9-methylenedioxy pterocarpan | Heat shock protein HSP 90 | DrugBank |  |
| MOL013280 | (-)-3-Hydroxy-4-methoxy-8-9-methylenedioxy pterocarpan | Phosphatidylinositol-4,5-bisphosphate 3-kinase catalytic subunit, gamma isoform | DrugBank |  |
| MOL013280 | (-)-3-Hydroxy-4-methoxy-8-9-methylenedioxy pterocarpan | mRNA of PKA Catalytic Subunit C-alpha | DrugBank |  |
| MOL013280 | (-)-3-Hydroxy-4-methoxy-8-9-methylenedioxy pterocarpan | Trypsin-1 | DrugBank |  |
| MOL013280 | (-)-3-Hydroxy-4-methoxy-8-9-methylenedioxy pterocarpan | Calcium-activated potassium channel subunit alpha 1 | DrugBank |  |
| MOL013280 | (-)-3-Hydroxy-4-methoxy-8-9-methylenedioxy pterocarpan | Calmodulin | DrugBank |  |
| MOL013281 | 6,8-Dihydroxy-7-methoxyxanthone | Prostaglandin G/H synthase 1 | DrugBank |  |
| MOL013281 | 6,8-Dihydroxy-7-methoxyxanthone | Prostaglandin G/H synthase 2 | DrugBank |  |
| MOL013281 | 6,8-Dihydroxy-7-methoxyxanthone | Carbonic anhydrase II | DrugBank |  |
| MOL013281 | 6,8-Dihydroxy-7-methoxyxanthone | Beta-2 adrenergic receptor | DrugBank |  |
| MOL013281 | 6,8-Dihydroxy-7-methoxyxanthone | Dipeptidyl peptidase IV | DrugBank |  |
| MOL013281 | 6,8-Dihydroxy-7-methoxyxanthone | Mitogen-activated protein kinase 14 | DrugBank |  |
| MOL013281 | 6,8-Dihydroxy-7-methoxyxanthone | Glycogen synthase kinase-3 beta | DrugBank |  |
| MOL013281 | 6,8-Dihydroxy-7-methoxyxanthone | Heat shock protein HSP 90 | DrugBank |  |
| MOL013281 | 6,8-Dihydroxy-7-methoxyxanthone | Cell division protein kinase 2 | DrugBank |  |
| MOL013281 | 6,8-Dihydroxy-7-methoxyxanthone | Phosphatidylinositol-4,5-bisphosphate 3-kinase catalytic subunit, gamma isoform | DrugBank |  |
| MOL013281 | 6,8-Dihydroxy-7-methoxyxanthone | Serine/threonine-protein kinase Chk1 | DrugBank |  |
| MOL013281 | 6,8-Dihydroxy-7-methoxyxanthone | mRNA of PKA Catalytic Subunit C-alpha | DrugBank |  |
| MOL013281 | 6,8-Dihydroxy-7-methoxyxanthone | cAMP-dependent protein kinase inhibitor alpha | DrugBank |  |
| MOL013283 | 3,4,3',5'-Tetrahydroxystilbene-3-glucoside | Prostaglandin G/H synthase 2 | DrugBank |  |
| MOL013283 | 3,4,3',5'-Tetrahydroxystilbene-3-glucoside | Carbonic anhydrase II | DrugBank |  |
| MOL013283 | 3,4,3',5'-Tetrahydroxystilbene-3-glucoside | Heat shock protein HSP 90 | DrugBank |  |
| MOL006468 | Anthraglycoside A | Prostaglandin G/H synthase 2 | DrugBank |  |
| MOL006468 | Anthraglycoside A | DNA topoisomerase II | DrugBank |  |
| MOL013286 | Fallacinol | Prostaglandin G/H synthase 1 | DrugBank |  |
| MOL013286 | Fallacinol | Sodium channel protein type 5 subunit alpha | DrugBank |  |
| MOL013286 | Fallacinol | Prostaglandin G/H synthase 2 | DrugBank |  |
| MOL013286 | Fallacinol | Carbonic anhydrase II | DrugBank |  |
| MOL013286 | Fallacinol | Coagulation factor VII | DrugBank |  |
| MOL013286 | Fallacinol | DNA topoisomerase II | DrugBank |  |
| MOL013286 | Fallacinol | Heat shock protein HSP 90 | DrugBank |  |
| MOL013286 | Fallacinol | Phosphatidylinositol-4,5-bisphosphate 3-kinase catalytic subunit, gamma isoform | DrugBank |  |
| MOL013286 | Fallacinol | Ig gamma-1 chain C region | DrugBank |  |
| MOL013286 | Fallacinol | Nuclear receptor coactivator 2 | DrugBank |  |
| MOL013286 | Fallacinol | Nuclear receptor coactivator 1 | DrugBank |  |
| MOL013287 | Physovenine | Nitric oxide synthase, inducible | DrugBank |  |
| MOL013287 | Physovenine | Prostaglandin G/H synthase 1 | DrugBank |  |
| MOL013287 | Physovenine | Dopamine D1 receptor | DrugBank |  |
| MOL013287 | Physovenine | Muscarinic acetylcholine receptor M3 | DrugBank |  |
| MOL013287 | Physovenine | Thrombin | DrugBank |  |
| MOL013287 | Physovenine | Muscarinic acetylcholine receptor M1 | DrugBank |  |
| MOL013287 | Physovenine | Estrogen receptor | DrugBank |  |
| MOL013287 | Physovenine | Androgen receptor | DrugBank |  |
| MOL013287 | Physovenine | Sodium channel protein type 5 subunit alpha | DrugBank |  |
| MOL013287 | Physovenine | Prostaglandin G/H synthase 2 | DrugBank |  |
| MOL013287 | Physovenine | Nitric-oxide synthase, endothelial | DrugBank |  |
| MOL013287 | Physovenine | Carbonic anhydrase II | DrugBank |  |
| MOL013287 | Physovenine | Retinoic acid receptor RXR-alpha | DrugBank |  |
| MOL013287 | Physovenine | Delta-type opioid receptor | DrugBank |  |
| MOL013287 | Physovenine | Acetylcholinesterase | DrugBank |  |
| MOL013287 | Physovenine | CGMP-inhibited 3',5'-cyclic phosphodiesterase A | DrugBank |  |
| MOL013287 | Physovenine | Sodium-dependent noradrenaline transporter | DrugBank |  |
| MOL013287 | Physovenine | Alpha-1A adrenergic receptor | DrugBank |  |
| MOL013287 | Physovenine | Muscarinic acetylcholine receptor M2 | DrugBank |  |
| MOL013287 | Physovenine | Alpha-2B adrenergic receptor | DrugBank |  |
| MOL013287 | Physovenine | Alpha-1B adrenergic receptor | DrugBank |  |
| MOL013287 | Physovenine | Sodium-dependent dopamine transporter | DrugBank |  |
| MOL013287 | Physovenine | Beta-2 adrenergic receptor | DrugBank |  |
| MOL013287 | Physovenine | Neuronal acetylcholine receptor subunit alpha-2 | DrugBank |  |
| MOL013287 | Physovenine | Sodium-dependent serotonin transporter | DrugBank |  |
| MOL013287 | Physovenine | Mu-type opioid receptor | DrugBank |  |
| MOL013287 | Physovenine | Estrogen receptor beta | DrugBank |  |
| MOL013287 | Physovenine | Gamma-aminobutyric acid receptor subunit alpha-1 | DrugBank |  |
| MOL013287 | Physovenine | Glycogen synthase kinase-3 beta | DrugBank |  |
| MOL013287 | Physovenine | Heat shock protein HSP 90 | DrugBank |  |
| MOL013287 | Physovenine | Cell division protein kinase 2 | DrugBank |  |
| MOL013287 | Physovenine | Beta-lactamase | DrugBank |  |
| MOL013287 | Physovenine | Neuronal acetylcholine receptor protein, alpha-7 chain | DrugBank |  |
| MOL013287 | Physovenine | mRNA of PKA Catalytic Subunit C-alpha | DrugBank |  |
| MOL013287 | Physovenine | Trypsin-1 | DrugBank |  |
| MOL013287 | Physovenine | Proto-oncogene serine/threonine-protein kinase Pim-1 | DrugBank |  |
| MOL013287 | Physovenine | Cyclin-A2 | DrugBank |  |
| MOL013287 | Physovenine | Glutamate receptor 2 | DrugBank |  |
| MOL013288 | Picralinal | Androgen receptor | DrugBank |  |
| MOL013288 | Picralinal | Sodium channel protein type 5 subunit alpha | DrugBank |  |
| MOL013288 | Picralinal | Delta-type opioid receptor | DrugBank |  |
| MOL013288 | Picralinal | Mu-type opioid receptor | DrugBank |  |
| MOL013289 | polydatin | Prostaglandin G/H synthase 2 | DrugBank |  |
| MOL013289 | polydatin | Carbonic anhydrase II | DrugBank |  |
| MOL013289 | polydatin | Calmodulin | DrugBank |  |
| MOL013289 | polydatin | Phospholipase B1, membrane-associated | N/A | validated |
| MOL013293 | Questinol | Prostaglandin G/H synthase 1 | DrugBank |  |
| MOL013293 | Questinol | Sodium channel protein type 5 subunit alpha | DrugBank |  |
| MOL013293 | Questinol | Prostaglandin G/H synthase 2 | DrugBank |  |
| MOL013293 | Questinol | Nitric-oxide synthase, endothelial | DrugBank |  |
| MOL013293 | Questinol | Carbonic anhydrase II | DrugBank |  |
| MOL013293 | Questinol | Coagulation factor VII | DrugBank |  |
| MOL013293 | Questinol | DNA topoisomerase II | DrugBank |  |
| MOL013293 | Questinol | Heat shock protein HSP 90 | DrugBank |  |
| MOL013293 | Questinol | Phosphatidylinositol-4,5-bisphosphate 3-kinase catalytic subunit, gamma isoform | DrugBank |  |
| MOL013293 | Questinol | Nuclear receptor coactivator 2 | DrugBank |  |
| MOL013293 | Questinol | Calmodulin | DrugBank |  |
| MOL013295 | Torachrysone-8-O-beta-D-glucoside | Coagulation factor Xa | DrugBank |  |
| MOL013295 | Torachrysone-8-O-beta-D-glucoside | Prostaglandin G/H synthase 2 | DrugBank |  |
| MOL013295 | Torachrysone-8-O-beta-D-glucoside | Carbonic anhydrase II | DrugBank |  |
| MOL013295 | Torachrysone-8-O-beta-D-glucoside | DNA topoisomerase II | DrugBank |  |
| MOL013295 | Torachrysone-8-O-beta-D-glucoside | Nuclear receptor coactivator 2 | DrugBank |  |
| MOL001456 | citric acid | Coagulation factor Xa | DrugBank |  |
| MOL001456 | citric acid | Prostaglandin G/H synthase 2 | DrugBank |  |
| MOL001456 | citric acid | Aldose reductase | DrugBank |  |
| MOL001456 | citric acid | Proto-oncogene tyrosine-protein kinase SRC | DrugBank |  |
| MOL001456 | citric acid | Cathepsin D | DrugBank |  |
| MOL001456 | citric acid | Ig gamma-1 chain C region | DrugBank |  |
| MOL001456 | citric acid | Nitric-oxide synthase, brain | DrugBank |  |
| MOL001456 | citric acid | Glutamate [NMDA] receptor subunit epsilon 1 | DrugBank |  |
| MOL001456 | citric acid | Gamma-aminobutyric acid receptor subunit alpha-1 | DrugBank |  |
| MOL001456 | citric acid | Ferrichrome-iron receptor | DrugBank |  |
| MOL001456 | citric acid | Glutamate receptor 2 | DrugBank |  |
| MOL001456 | citric acid | mRNA of Protein-tyrosine phosphatase, non-receptor type 1 | DrugBank |  |
| MOL001456 | citric acid | Bacillolysin | DrugBank |  |
| MOL001456 | citric acid | Carboxypeptidase B | DrugBank |  |
| MOL001456 | citric acid | Triosephosphate isomerase | DrugBank |  |
| MOL001456 | citric acid | Glutamate receptor, ionotropic kainate 2 | DrugBank |  |
| MOL001456 | citric acid | Histidine decarboxylase | DrugBank | validated |
| MOL001468 | MLT | 4-aminobutyrate aminotransferase, mitochondrial | DrugBank |  |
| MOL001468 | MLT | Aspartate aminotransferase, cytoplasmic | DrugBank |  |
| MOL001468 | MLT | mRNA of Protein-tyrosine phosphatase, non-receptor type 1 | DrugBank |  |
| MOL001468 | MLT | Aldose reductase | DrugBank |  |
| MOL001468 | MLT | Gamma-aminobutyric acid receptor subunit alpha-1 | DrugBank |  |
| MOL001468 | MLT | Cathepsin D | DrugBank |  |
| MOL001468 | MLT | Cholinesterase | DrugBank |  |
| MOL001468 | MLT | Alcohol dehydrogenase 1B | DrugBank |  |
| MOL001468 | MLT | Alcohol dehydrogenase 1C | DrugBank |  |
| MOL001468 | MLT | Cytochrome P450-cam | DrugBank |  |
| MOL001468 | MLT | Aspartate aminotransferase | DrugBank |  |
| MOL001468 | MLT | Ferrichrome-iron receptor | DrugBank |  |
| MOL001468 | MLT | Formate acetyltransferase 1 | DrugBank |  |
| MOL001468 | MLT | Adenylosuccinate synthetase | DrugBank |  |
| MOL001468 | MLT | Bacillolysin | DrugBank |  |
| MOL001468 | MLT | 1-aminocyclopropane-1-carboxylate deaminase | DrugBank |  |
| MOL001468 | MLT | Nicotinate-nucleotide--dimethylbenzimidazole phosphoribosyltransferase | DrugBank |  |
| MOL001468 | MLT | Growth-inhibiting protein 18 | DrugBank |  |
| MOL001468 | MLT | Aspartate aminotransferase, mitochondrial | DrugBank |  |
| MOL001468 | MLT | S-adenosylmethionine synthetase isoform type-2 | DrugBank |  |
| MOL001468 | MLT | Prolyl 3-hydroxylase 3 | DrugBank |  |
| MOL001468 | MLT | S-adenosylmethionine synthetase isoform type-1 | DrugBank |  |
| MOL001468 | MLT | Adenylosuccinate synthetase isozyme 1 | DrugBank |  |
| MOL001468 | MLT | Triosephosphate isomerase | DrugBank |  |
| MOL001468 | MLT | NAD-dependent malic enzyme, mitochondrial | DrugBank |  |
| MOL001468 | MLT | Proto-oncogene tyrosine-protein kinase SRC | DrugBank |  |
| MOL001468 | MLT | Glutamate dehydrogenase 1, mitochondrial | DrugBank |  |
| MOL001468 | MLT | Glycine receptor alpha-1 chain | DrugBank |  |
| MOL001468 | MLT | Dihydroorotase | DrugBank |  |
| MOL001468 | MLT | mRNA of PKA Catalytic Subunit C-alpha | DrugBank |  |
| MOL001468 | MLT | Arginase-1 | DrugBank |  |
| MOL001468 | MLT | Serine--pyruvate aminotransferase | DrugBank |  |
| MOL001468 | MLT | Thioredoxin reductase, cytoplasmic | DrugBank |  |
| MOL001468 | MLT | Malonamidase E2 | DrugBank |  |
| MOL001468 | MLT | NADP-dependent malic enzyme, mitochondrial | DrugBank |  |
| MOL001468 | MLT | Nitric-oxide synthase, brain | DrugBank |  |
| MOL001468 | MLT | Alanine aminotransferase 1 | DrugBank |  |
| MOL001729 | Crysophanol | Prostaglandin G/H synthase 1 | DrugBank |  |
| MOL001729 | Crysophanol | Prostaglandin G/H synthase 2 | DrugBank |  |
| MOL001729 | Crysophanol | Heat shock protein HSP 90 | DrugBank |  |
| MOL001729 | Crysophanol | Nuclear receptor coactivator 2 | DrugBank |  |
| MOL001729 | Crysophanol | Calmodulin | DrugBank |  |
| MOL001729 | Crysophanol | Sodium channel protein type 5 subunit alpha | DrugBank |  |
| MOL001729 | Crysophanol | CGMP-inhibited 3',5'-cyclic phosphodiesterase A | DrugBank |  |
| MOL001729 | Crysophanol | Gamma-aminobutyric acid receptor subunit alpha-1 | DrugBank |  |
| MOL001729 | Crysophanol | Phosphatidylinositol-4,5-bisphosphate 3-kinase catalytic subunit, gamma isoform | DrugBank |  |
| MOL001729 | Crysophanol | mRNA of PKA Catalytic Subunit C-alpha | DrugBank |  |
| MOL001729 | Crysophanol | Ig gamma-1 chain C region | DrugBank |  |
| MOL001729 | Crysophanol | cAMP-dependent protein kinase inhibitor alpha | DrugBank |  |
| MOL001829 | Glucofrangulin | Prostaglandin G/H synthase 2 | DrugBank |  |
| MOL001829 | Glucofrangulin | DNA topoisomerase II | DrugBank |  |
| MOL001880 | OXL | Prostaglandin G/H synthase 1 | DrugBank |  |
| MOL001880 | OXL | Nitric-oxide synthase, brain | DrugBank |  |
| MOL001880 | OXL | Procollagen-lysine,2-oxoglutarate 5-dioxygenase 1 | DrugBank |  |
| MOL001880 | OXL | Aldehyde dehydrogenase, mitochondrial | DrugBank |  |
| MOL001880 | OXL | Succinate semialdehyde dehydrogenase, mitochondrial | DrugBank |  |
| MOL001880 | OXL | 4-aminobutyrate aminotransferase, mitochondrial | DrugBank |  |
| MOL001880 | OXL | Prostaglandin G/H synthase 2 | DrugBank |  |
| MOL001880 | OXL | Serine hydroxymethyltransferase, mitochondrial | DrugBank |  |
| MOL001880 | OXL | DNA polymerase (HSV) | DrugBank |  |
| MOL001880 | OXL | Glutamate [NMDA] receptor subunit zeta-1 | DrugBank |  |
| MOL001880 | OXL | Kynureninase | DrugBank |  |
| MOL001880 | OXL | Ornithine aminotransferase, mitochondrial | DrugBank |  |
| MOL001880 | OXL | Glycine receptor alpha-1 chain | DrugBank |  |
| MOL001880 | OXL | Aspartate aminotransferase, cytoplasmic | DrugBank |  |
| MOL001880 | OXL | Glutamate [NMDA] receptor subunit epsilon 1 | DrugBank |  |
| MOL001880 | OXL | Gamma-aminobutyric acid receptor subunit alpha-1 | DrugBank |  |
| MOL001880 | OXL | Proto-oncogene tyrosine-protein kinase SRC | DrugBank |  |
| MOL001880 | OXL | Cathepsin D | DrugBank |  |
| MOL001880 | OXL | Lactotransferrin | DrugBank |  |
| MOL001880 | OXL | Macrophage metalloelastase | DrugBank |  |
| MOL001880 | OXL | Leukotriene A-4 hydrolase | DrugBank |  |
| MOL001880 | OXL | M-phase inducer phosphatase 2 | DrugBank |  |
| MOL001880 | OXL | Thioredoxin reductase, cytoplasmic | DrugBank |  |
| MOL001880 | OXL | Cholinesterase | DrugBank |  |
| MOL001880 | OXL | Xanthine dehydrogenase/oxidase | DrugBank |  |
| MOL001880 | OXL | Alcohol dehydrogenase 1B | DrugBank |  |
| MOL001880 | OXL | Alcohol dehydrogenase 1C | DrugBank |  |
| MOL001880 | OXL | Alcohol dehydrogenase 1A | DrugBank |  |
| MOL001880 | OXL | Catalase | DrugBank |  |
| MOL001880 | OXL | Glycine dehydrogenase [decarboxylating], mitochondrial | DrugBank |  |
| MOL001880 | OXL | Ribonucleoside-diphosphate reductase large subunit | DrugBank |  |
| MOL001880 | OXL | Pyruvate kinase isozymes R/L | DrugBank |  |
| MOL001880 | OXL | Dihydroorotate dehydrogenase, mitochondrial | DrugBank |  |
| MOL001880 | OXL | Monocarboxylate transporter 2 | DrugBank |  |
| MOL001880 | OXL | Cytochrome P450-cam | DrugBank |  |
| MOL001880 | OXL | Aspartate aminotransferase | DrugBank |  |
| MOL001880 | OXL | Beta-galactosidase | DrugBank |  |
| MOL001880 | OXL | Ferrichrome-iron receptor | DrugBank |  |
| MOL001880 | OXL | Histidinol dehydrogenase | DrugBank |  |
| MOL001880 | OXL | Pyruvate dehydrogenase [cytochrome] | DrugBank |  |
| MOL001880 | OXL | Acetyl-CoA acetyltransferase | DrugBank |  |
| MOL001880 | OXL | Formate dehydrogenase H | DrugBank |  |
| MOL001880 | OXL | Formate acetyltransferase 1 | DrugBank |  |
| MOL001880 | OXL | Adenylosuccinate synthetase | DrugBank |  |
| MOL001880 | OXL | Succinate dehydrogenase flavoprotein subunit | DrugBank |  |
| MOL001880 | OXL | Alanine racemase | DrugBank |  |
| MOL001880 | OXL | Gag-Pol polyprotein | DrugBank |  |
| MOL001880 | OXL | Methionine synthase | DrugBank |  |
| MOL001880 | OXL | C4-dicarboxylate transport transcriptional regulatory protein dctD | DrugBank |  |
| MOL001880 | OXL | D-alanyl-D-alanine carboxypeptidase | DrugBank |  |
| MOL001880 | OXL | Isocitrate dehydrogenase [NADP] | DrugBank |  |
| MOL001880 | OXL | Phosphoenolpyruvate carboxykinase [ATP] | DrugBank |  |
| MOL001880 | OXL | Siroheme synthase | DrugBank |  |
| MOL001880 | OXL | Aconitate hydratase 2 | DrugBank |  |
| MOL001880 | OXL | Monomeric sarcosine oxidase | DrugBank |  |
| MOL001880 | OXL | Bacillolysin | DrugBank |  |
| MOL001880 | OXL | Dihydroxyacetone kinase | DrugBank |  |
| MOL001880 | OXL | Haloalkane dehalogenase | DrugBank |  |
| MOL001880 | OXL | Gamma-aminobutyraldehyde dehydrogenase | DrugBank |  |
| MOL001880 | OXL | Fumarate reductase flavoprotein subunit | DrugBank |  |
| MOL001880 | OXL | 2-isopropylmalate synthase | DrugBank |  |
| MOL001880 | OXL | 1-aminocyclopropane-1-carboxylate deaminase | DrugBank |  |
| MOL001880 | OXL | Hydroxylamine reductase | DrugBank |  |
| MOL001880 | OXL | Nicotinate-nucleotide--dimethylbenzimidazole phosphoribosyltransferase | DrugBank |  |
| MOL001880 | OXL | Glucose--fructose oxidoreductase | DrugBank |  |
| MOL001880 | OXL | Growth-inhibiting protein 18 | DrugBank |  |
| MOL001880 | OXL | Serine hydroxymethyltransferase 2 | DrugBank |  |
| MOL001880 | OXL | Serine hydroxymethyltransferase 1 | DrugBank |  |
| MOL001880 | OXL | Chondroitinase AC | DrugBank |  |
| MOL001880 | OXL | Methylmalonyl-CoA carboxyltransferase 5S subunit | DrugBank |  |
| MOL001880 | OXL | L-cysteine/cystine lyase C-DES | DrugBank |  |
| MOL001880 | OXL | Malonamidase E2 | DrugBank |  |
| MOL001880 | OXL | Serotransferrin | DrugBank |  |
| MOL001880 | OXL | Glutamate dehydrogenase 1, mitochondrial | DrugBank |  |
| MOL001880 | OXL | Aspartate aminotransferase, mitochondrial | DrugBank |  |
| MOL001880 | OXL | Pyruvate kinase isozymes M1/M2 | DrugBank |  |
| MOL001880 | OXL | Serine--pyruvate aminotransferase | DrugBank |  |
| MOL001880 | OXL | Monocarboxylate transporter 7 | DrugBank |  |
| MOL001880 | OXL | Alanine aminotransferase 1 | DrugBank |  |
| MOL001880 | OXL | Aldehyde dehydrogenase X, mitochondrial | DrugBank |  |
| MOL001880 | OXL | Succinate dehydrogenase [ubiquinone] flavoprotein subunit, mitochondrial | DrugBank |  |
| MOL001880 | OXL | Cystathionine gamma-lyase | DrugBank |  |
| MOL001880 | OXL | Serine hydroxymethyltransferase, cytosolic | DrugBank |  |
| MOL001880 | OXL | Betaine--homocysteine S-methyltransferase 1 | DrugBank |  |
| MOL001880 | OXL | Succinyl-CoA:3-ketoacid-coenzyme A transferase 2, mitochondrial | DrugBank |  |
| MOL001880 | OXL | Alanine--glyoxylate aminotransferase 2, mitochondrial | DrugBank |  |
| MOL001880 | OXL | NADP-dependent malic enzyme | DrugBank |  |
| MOL001880 | OXL | Glutamate dehydrogenase 2, mitochondrial | DrugBank |  |
| MOL001880 | OXL | Alanyl-tRNA synthetase, cytoplasmic | DrugBank |  |
| MOL001880 | OXL | Glycine amidinotransferase, mitochondrial | DrugBank |  |
| MOL001880 | OXL | Calcium-transporting ATPase type 2C member 1 | DrugBank |  |
| MOL001880 | OXL | S-adenosylmethionine synthetase isoform type-1 | DrugBank |  |
| MOL001880 | OXL | Cysteine desulfurase, mitochondrial | DrugBank |  |
| MOL001880 | OXL | Alanine--glyoxylate aminotransferase 2-like 2 | DrugBank |  |
| MOL001880 | OXL | Proton-coupled amino acid transporter 1 | DrugBank |  |
| MOL001880 | OXL | Adenylosuccinate synthetase isozyme 1 | DrugBank |  |
| MOL001880 | OXL | L-lactate dehydrogenase A chain | DrugBank |  |
| MOL001880 | OXL | Glycine receptor subunit alpha-2 | DrugBank |  |
| MOL001880 | OXL | 2-amino-3-ketobutyrate coenzyme A ligase, mitochondrial | DrugBank |  |
| MOL001880 | OXL | Pyruvate dehydrogenase E1 component subunit beta, mitochondrial | DrugBank |  |
| MOL001880 | OXL | Triosephosphate isomerase | DrugBank |  |
| MOL001880 | OXL | Delta-1-pyrroline-5-carboxylate dehydrogenase, mitochondrial | DrugBank |  |
| MOL001880 | OXL | NAD-dependent malic enzyme, mitochondrial | DrugBank |  |
| MOL001880 | OXL | Glycine receptor subunit alpha-3 | DrugBank |  |
| MOL001880 | OXL | SHMT2 protein | DrugBank |  |
| MOL001880 | OXL | NADP-dependent malic enzyme, mitochondrial | DrugBank |  |
| MOL001880 | OXL | L-lactate dehydrogenase B chain | DrugBank |  |
| MOL001880 | OXL | Trypsin-3 | DrugBank |  |
| MOL001880 | OXL | Sigma factor sigB regulation protein rsbQ | DrugBank |  |
| MOL001880 | OXL | Aromatic-amino-acid aminotransferase | DrugBank |  |
| MOL001880 | OXL | Aldose reductase | DrugBank |  |
| MOL001880 | OXL | Glycogen phosphorylase, muscle form | DrugBank |  |
| MOL001880 | OXL | Fumarate hydratase class II | DrugBank |  |
| MOL001880 | OXL | N-acetylneuraminate lyase | DrugBank |  |
| MOL001880 | OXL | Phosphate-binding protein pstS precursor | DrugBank |  |
| MOL001880 | OXL | Beta-amylase | DrugBank |  |
| MOL001880 | OXL | Tyrosine-protein kinase transforming protein Src | DrugBank |  |
| MOL001880 | OXL | 2-hydroxy-6-oxo-7-methylocta-2,4-dienoate hydrolase | DrugBank |  |
| MOL001880 | OXL | Phosphotriesterase | DrugBank |  |
| MOL001880 | OXL | NADH-ubiquinone oxidoreductase 75 kDa subunit, mitochondrial | DrugBank |  |
| MOL001880 | OXL | Gephyrin | DrugBank |  |
| MOL001880 | OXL | Succinyl-CoA:3-ketoacid-coenzyme A transferase 1, mitochondrial | DrugBank |  |
| MOL001880 | OXL | Prolyl 4-hydroxylase subunit alpha-2 | DrugBank |  |
| MOL001880 | OXL | Calcium-binding mitochondrial carrier protein Aralar2 | DrugBank |  |
| MOL001880 | OXL | Succinyl-CoA ligase [ADP-forming] beta-chain, mitochondrial | DrugBank |  |
| MOL001886 | Tar | Prostaglandin G/H synthase 1 | DrugBank |  |
| MOL001886 | Tar | Nitric-oxide synthase, brain | DrugBank |  |
| MOL001886 | Tar | 4-aminobutyrate aminotransferase, mitochondrial | DrugBank |  |
| MOL001886 | Tar | Prostaglandin G/H synthase 2 | DrugBank |  |
| MOL001886 | Tar | Glycine receptor alpha-1 chain | DrugBank |  |
| MOL001886 | Tar | Aspartate aminotransferase, cytoplasmic | DrugBank |  |
| MOL001886 | Tar | mRNA of Protein-tyrosine phosphatase, non-receptor type 1 | DrugBank |  |
| MOL001886 | Tar | Glutamate receptor 1 | DrugBank |  |
| MOL001886 | Tar | Aldose reductase | DrugBank |  |
| MOL001886 | Tar | Glutamate [NMDA] receptor subunit epsilon 1 | DrugBank |  |
| MOL001886 | Tar | Gamma-aminobutyric acid receptor subunit alpha-1 | DrugBank |  |
| MOL001886 | Tar | Glycogen phosphorylase, muscle form | DrugBank |  |
| MOL001886 | Tar | Cathepsin D | DrugBank |  |
| MOL001886 | Tar | Lactotransferrin | DrugBank |  |
| MOL001886 | Tar | Cholinesterase | DrugBank |  |
| MOL001886 | Tar | Alcohol dehydrogenase 1B | DrugBank |  |
| MOL001886 | Tar | Alcohol dehydrogenase 1C | DrugBank |  |
| MOL001886 | Tar | Cytochrome P450-cam | DrugBank |  |
| MOL001886 | Tar | Aspartate aminotransferase | DrugBank |  |
| MOL001886 | Tar | Ferrichrome-iron receptor | DrugBank |  |
| MOL001886 | Tar | Formate acetyltransferase 1 | DrugBank |  |
| MOL001886 | Tar | 3-phosphoshikimate 1-carboxyvinyltransferase | DrugBank |  |
| MOL001886 | Tar | Adenylosuccinate synthetase | DrugBank |  |
| MOL001886 | Tar | Glucarate dehydratase | DrugBank |  |
| MOL001886 | Tar | Bacillolysin | DrugBank |  |
| MOL001886 | Tar | Growth-inhibiting protein 18 | DrugBank |  |
| MOL001886 | Tar | Methylmalonyl-CoA carboxyltransferase 5S subunit | DrugBank |  |
| MOL001886 | Tar | Aspartate aminotransferase, mitochondrial | DrugBank |  |
| MOL001886 | Tar | Glutamate receptor 2 | DrugBank |  |
| MOL001886 | Tar | Calcium-transporting ATPase type 2C member 1 | DrugBank |  |
| MOL001886 | Tar | Glutamate receptor, ionotropic kainate 2 | DrugBank |  |
| MOL001886 | Tar | Triosephosphate isomerase | DrugBank |  |
| MOL001886 | Tar | NAD-dependent malic enzyme, mitochondrial | DrugBank |  |
| MOL001886 | Tar | Arachidonate 5-lipoxygenase | DrugBank |  |
| MOL001886 | Tar | Dihydroorotase | DrugBank |  |
| MOL001886 | Tar | mRNA of PKA Catalytic Subunit C-alpha | DrugBank |  |
| MOL001886 | Tar | Dihydroorotate dehydrogenase, mitochondrial | DrugBank |  |
| MOL001886 | Tar | Glycogen phosphorylase, liver form | DrugBank |  |
| MOL001886 | Tar | S-adenosylmethionine synthetase isoform type-1 | DrugBank |  |
| MOL001886 | Tar | Serine--pyruvate aminotransferase | DrugBank |  |
| MOL001886 | Tar | S-adenosylmethionine synthetase | DrugBank |  |
| MOL001886 | Tar | Glutamate dehydrogenase 1, mitochondrial | DrugBank |  |
| MOL001886 | Tar | Prolyl 3-hydroxylase 3 | DrugBank |  |
| MOL001886 | Tar | Galactosylgalactosylxylosylprotein 3-beta-glucuronosyltransferase 1 | DrugBank |  |
| MOL001886 | Tar | Endoplasmic reticulum mannosyl-oligosaccharide 1,2-alpha-mannosidase | DrugBank |  |
| MOL002055 | Torachrysone | Prostaglandin G/H synthase 1 | DrugBank |  |
| MOL002055 | Torachrysone | Sodium channel protein type 5 subunit alpha | DrugBank |  |
| MOL002055 | Torachrysone | Prostaglandin G/H synthase 2 | DrugBank |  |
| MOL002055 | Torachrysone | Nitric-oxide synthase, endothelial | DrugBank |  |
| MOL002055 | Torachrysone | Retinoic acid receptor RXR-alpha | DrugBank |  |
| MOL002055 | Torachrysone | CGMP-inhibited 3',5'-cyclic phosphodiesterase A | DrugBank |  |
| MOL002055 | Torachrysone | Alpha-1A adrenergic receptor | DrugBank |  |
| MOL002055 | Torachrysone | Alpha-1B adrenergic receptor | DrugBank |  |
| MOL002055 | Torachrysone | Beta-2 adrenergic receptor | DrugBank |  |
| MOL002055 | Torachrysone | Estrogen receptor beta | DrugBank |  |
| MOL002055 | Torachrysone | Gamma-aminobutyric acid receptor subunit alpha-1 | DrugBank |  |
| MOL002055 | Torachrysone | Heat shock protein HSP 90 | DrugBank |  |
| MOL002055 | Torachrysone | Beta-lactamase | DrugBank |  |
| MOL002055 | Torachrysone | Leukotriene A-4 hydrolase | DrugBank |  |
| MOL002055 | Torachrysone | Amine oxidase [flavin-containing] B | DrugBank |  |
| MOL002055 | Torachrysone | Neuronal acetylcholine receptor protein, alpha-7 chain | DrugBank |  |
| MOL002055 | Torachrysone | mRNA of PKA Catalytic Subunit C-alpha | DrugBank |  |
| MOL002055 | Torachrysone | Nuclear receptor coactivator 2 | DrugBank |  |
| MOL002055 | Torachrysone | cAMP-dependent protein kinase inhibitor alpha | DrugBank |  |
| MOL002055 | Torachrysone | Beta-1 adrenergic receptor | DrugBank |  |
| MOL002055 | Torachrysone | Alpha-2A adrenergic receptor | DrugBank |  |
| MOL002055 | Torachrysone | Alpha-2C adrenergic receptor | DrugBank |  |
| MOL002055 | Torachrysone | Sodium-dependent serotonin transporter | DrugBank |  |
| MOL002055 | Torachrysone | Ig gamma-1 chain C region | DrugBank |  |
| MOL002055 | Torachrysone | Glutamate receptor 2 | DrugBank |  |
| MOL002055 | Torachrysone | Nuclear receptor coactivator 1 | DrugBank |  |
| MOL002243 | Anthraglycoside B | Coagulation factor Xa | DrugBank |  |
| MOL002243 | Anthraglycoside B | DNA topoisomerase II | DrugBank |  |
| MOL002247 | Emodin-6-glucoside | Coagulation factor Xa | DrugBank |  |
| MOL002259 | Physciondiglucoside | DNA topoisomerase II | DrugBank |  |
| MOL002267 | Rhein diglucoside | DNA topoisomerase II | DrugBank |  |
| MOL002268 | rhein | Prostaglandin G/H synthase 1 | DrugBank |  |
| MOL002268 | rhein | Prostaglandin G/H synthase 2 | DrugBank |  |
| MOL002268 | rhein | Heat shock protein HSP 90 | DrugBank |  |
| MOL002268 | rhein | Phosphatidylinositol-4,5-bisphosphate 3-kinase catalytic subunit, gamma isoform | DrugBank |  |
| MOL002268 | rhein | Nuclear receptor coactivator 2 | DrugBank |  |
| MOL002268 | rhein | Aldose reductase | DrugBank |  |
| MOL002268 | rhein | Transcription factor AP-1 | DrugBank | validated |
| MOL002280 | Torachrysone-8-O-beta-D-(6'-oxayl)-glucoside | DNA topoisomerase II | DrugBank |  |
| MOL000263 | oleanolic acid | Caspase-9 | N/A | validated |
| MOL000263 | oleanolic acid | Caspase-3 | N/A | validated |
| MOL000263 | oleanolic acid | Heme oxygenase 1 | DrugBank | validated |
| MOL000263 | oleanolic acid | Intercellular adhesion molecule 1 | N/A | validated |
| MOL000263 | oleanolic acid | NAD(P)H dehydrogenase [quinone] 1 | DrugBank | validated |
| MOL000263 | oleanolic acid | Pancreatic alpha-amylase | DrugBank | validated |
| MOL000357 | Sitogluside | Progesterone receptor | DrugBank |  |
| MOL000357 | Sitogluside | Prostaglandin G/H synthase 1 | DrugBank |  |
| MOL000357 | Sitogluside | Muscarinic acetylcholine receptor M3 | DrugBank |  |
| MOL000357 | Sitogluside | Potassium voltage-gated channel subfamily H member 2 | DrugBank |  |
| MOL000357 | Sitogluside | Muscarinic acetylcholine receptor M1 | DrugBank |  |
| MOL000357 | Sitogluside | Sodium channel protein type 5 subunit alpha | DrugBank |  |
| MOL000357 | Sitogluside | Coagulation factor Xa | DrugBank |  |
| MOL000357 | Sitogluside | Prostaglandin G/H synthase 2 | DrugBank |  |
| MOL000357 | Sitogluside | 5-hydroxytryptamine receptor 3A | DrugBank |  |
| MOL000357 | Sitogluside | Retinoic acid receptor RXR-alpha | DrugBank |  |
| MOL000357 | Sitogluside | CGMP-inhibited 3',5'-cyclic phosphodiesterase A | DrugBank |  |
| MOL000357 | Sitogluside | Alpha-1B adrenergic receptor | DrugBank |  |
| MOL000357 | Sitogluside | Beta-2 adrenergic receptor | DrugBank |  |
| MOL000357 | Sitogluside | Alpha-1D adrenergic receptor | DrugBank |  |
| MOL000357 | Sitogluside | Heat shock protein HSP 90 | DrugBank |  |
| MOL000357 | Sitogluside | Nuclear receptor coactivator 2 | DrugBank |  |
| MOL000357 | Sitogluside | Calmodulin | DrugBank |  |
| MOL000358 | beta-sitosterol | Progesterone receptor | DrugBank |  |
| MOL000358 | beta-sitosterol | Nuclear receptor coactivator 2 | DrugBank |  |
| MOL000358 | beta-sitosterol | Prostaglandin G/H synthase 1 | DrugBank |  |
| MOL000358 | beta-sitosterol | Prostaglandin G/H synthase 2 | DrugBank |  |
| MOL000358 | beta-sitosterol | Heat shock protein HSP 90 | DrugBank |  |
| MOL000358 | beta-sitosterol | Phosphatidylinositol-4,5-bisphosphate 3-kinase catalytic subunit, gamma isoform | DrugBank |  |
| MOL000358 | beta-sitosterol | Potassium voltage-gated channel subfamily H member 2 | DrugBank |  |
| MOL000358 | beta-sitosterol | mRNA of PKA Catalytic Subunit C-alpha | DrugBank |  |
| MOL000358 | beta-sitosterol | Dopamine D1 receptor | DrugBank |  |
| MOL000358 | beta-sitosterol | Muscarinic acetylcholine receptor M3 | DrugBank |  |
| MOL000358 | beta-sitosterol | Muscarinic acetylcholine receptor M1 | DrugBank |  |
| MOL000358 | beta-sitosterol | Sodium channel protein type 5 subunit alpha | DrugBank |  |
| MOL000358 | beta-sitosterol | Gamma-aminobutyric-acid receptor alpha-2 subunit | DrugBank |  |
| MOL000358 | beta-sitosterol | Muscarinic acetylcholine receptor M4 | DrugBank |  |
| MOL000358 | beta-sitosterol | CGMP-inhibited 3',5'-cyclic phosphodiesterase A | DrugBank |  |
| MOL000358 | beta-sitosterol | 5-hydroxytryptamine 2A receptor | DrugBank |  |
| MOL000358 | beta-sitosterol | Gamma-aminobutyric-acid receptor alpha-5 subunit | DrugBank |  |
| MOL000358 | beta-sitosterol | Alpha-1A adrenergic receptor | DrugBank |  |
| MOL000358 | beta-sitosterol | Gamma-aminobutyric-acid receptor alpha-3 subunit | DrugBank |  |
| MOL000358 | beta-sitosterol | Muscarinic acetylcholine receptor M2 | DrugBank |  |
| MOL000358 | beta-sitosterol | Alpha-1B adrenergic receptor | DrugBank |  |
| MOL000358 | beta-sitosterol | Beta-2 adrenergic receptor | DrugBank |  |
| MOL000358 | beta-sitosterol | Neuronal acetylcholine receptor subunit alpha-2 | DrugBank |  |
| MOL000358 | beta-sitosterol | Sodium-dependent serotonin transporter | DrugBank |  |
| MOL000358 | beta-sitosterol | Mu-type opioid receptor | DrugBank |  |
| MOL000358 | beta-sitosterol | Gamma-aminobutyric acid receptor subunit alpha-1 | DrugBank |  |
| MOL000358 | beta-sitosterol | Neuronal acetylcholine receptor protein, alpha-7 chain | DrugBank |  |
| MOL000358 | beta-sitosterol | Cytochrome P450-cam | DrugBank |  |
| MOL000358 | beta-sitosterol | Apoptosis regulator Bcl-2 | DrugBank | validated |
| MOL000358 | beta-sitosterol | Apoptosis regulator BAX | N/A | validated |
| MOL000358 | beta-sitosterol | Caspase-9 | N/A | validated |
| MOL000358 | beta-sitosterol | Transcription factor AP-1 | DrugBank | validated |
| MOL000358 | beta-sitosterol | Caspase-3 | N/A | validated |
| MOL000358 | beta-sitosterol | Caspase-8 | N/A | validated |
| MOL000358 | beta-sitosterol | Protein kinase C alpha type | N/A | validated |
| MOL000358 | beta-sitosterol | Transforming growth factor beta-1 | N/A | validated |
| MOL000358 | beta-sitosterol | Serum paraoxonase/arylesterase 1 | DrugBank | validated |
| MOL000358 | beta-sitosterol | Microtubule-associated protein 2 | DrugBank | validated |
| MOL000431 | coumarin | Thrombin | DrugBank |  |
| MOL000431 | coumarin | Prostaglandin G/H synthase 2 | DrugBank |  |
| MOL000431 | coumarin | Acetylcholinesterase | DrugBank |  |
| MOL000431 | coumarin | Amine oxidase [flavin-containing] B | DrugBank |  |
| MOL000431 | coumarin | Amine oxidase [flavin-containing] A | DrugBank |  |
| MOL000431 | coumarin | Apoptosis regulator BAX | N/A | validated |
| MOL000431 | coumarin | Xanthine dehydrogenase/oxidase | DrugBank | validated |
| MOL000431 | coumarin | Cytochrome P450 3A4 | DrugBank | validated |
| MOL000431 | coumarin | Gamma-glutamyltransferase 5 | N/A | validated |
| MOL000431 | coumarin | Arachidonate 5-lipoxygenase | DrugBank | validated |
| MOL000431 | coumarin | Glutamate--cysteine ligase catalytic subunit | DrugBank | validated |
| MOL000431 | coumarin | Glutathione S-transferase P | DrugBank | validated |
| MOL000431 | coumarin | Glutathione S-transferase A2 | DrugBank | validated |
| MOL000431 | coumarin | Aldo-keto reductase family 1 member C1 | DrugBank | validated |
| MOL000431 | coumarin | Antizyme inhibitor 1 | N/A | validated |
| MOL000431 | coumarin | Cytochrome P450 2A6 | DrugBank | validated |
| MOL004368 | Hyperin | DNA topoisomerase II | DrugBank |  |
| MOL004368 | Hyperin | mRNA of Protein-tyrosine phosphatase, non-receptor type 1 | DrugBank |  |
| MOL004368 | Hyperin | Trypsin-1 | DrugBank |  |
| MOL004368 | Hyperin | Nitric oxide synthase, inducible | DrugBank |  |
| MOL004368 | Hyperin | Prostaglandin G/H synthase 1 | DrugBank |  |
| MOL004368 | Hyperin | Prostaglandin G/H synthase 2 | DrugBank |  |
| MOL004368 | Hyperin | Heat shock protein HSP 90 | DrugBank |  |
| MOL004368 | Hyperin | Phosphatidylinositol-4,5-bisphosphate 3-kinase catalytic subunit, gamma isoform | DrugBank |  |
| MOL004368 | Hyperin | Nuclear receptor coactivator 2 | DrugBank |  |
| MOL000437 | Hirsutrin | DNA topoisomerase II | DrugBank |  |
| MOL000437 | Hirsutrin | mRNA of Protein-tyrosine phosphatase, non-receptor type 1 | DrugBank |  |
| MOL000437 | Hirsutrin | Coagulation factor Xa | DrugBank |  |
| MOL004570 | 5-[(Z)-2-(3,4-dihydroxyphenyl)vinyl]resorcinol | Prostaglandin G/H synthase 1 | DrugBank |  |
| MOL004570 | 5-[(Z)-2-(3,4-dihydroxyphenyl)vinyl]resorcinol | Prostaglandin G/H synthase 2 | DrugBank |  |
| MOL004570 | 5-[(Z)-2-(3,4-dihydroxyphenyl)vinyl]resorcinol | Heat shock protein HSP 90 | DrugBank |  |
| MOL004570 | 5-[(Z)-2-(3,4-dihydroxyphenyl)vinyl]resorcinol | Amine oxidase [flavin-containing] B | DrugBank |  |
| MOL004570 | 5-[(Z)-2-(3,4-dihydroxyphenyl)vinyl]resorcinol | mRNA of PKA Catalytic Subunit C-alpha | DrugBank |  |
| MOL004570 | 5-[(Z)-2-(3,4-dihydroxyphenyl)vinyl]resorcinol | Nuclear receptor coactivator 2 | DrugBank |  |
| MOL000472 | emodin | Prostaglandin G/H synthase 1 | DrugBank |  |
| MOL000472 | emodin | Prostaglandin G/H synthase 2 | DrugBank |  |
| MOL000472 | emodin | Coagulation factor VII | DrugBank |  |
| MOL000472 | emodin | Heat shock protein HSP 90 | DrugBank |  |
| MOL000472 | emodin | Phosphatidylinositol-4,5-bisphosphate 3-kinase catalytic subunit, gamma isoform | DrugBank |  |
| MOL000472 | emodin | mRNA of PKA Catalytic Subunit C-alpha | DrugBank |  |
| MOL000472 | emodin | Ig gamma-1 chain C region | DrugBank |  |
| MOL000472 | emodin | Coagulation factor Xa | DrugBank |  |
| MOL000472 | emodin | Vascular endothelial growth factor receptor 2 | DrugBank | validated |
| MOL000472 | emodin | DNA topoisomerase II | DrugBank |  |
| MOL000472 | emodin | Nuclear receptor coactivator 2 | DrugBank |  |
| MOL000472 | emodin | Nuclear receptor coactivator 1 | DrugBank |  |
| MOL000472 | emodin | Calmodulin | DrugBank |  |
| MOL000472 | emodin | Cyclin-dependent kinase inhibitor 1 | N/A | validated |
| MOL000472 | emodin | Vascular endothelial growth factor receptor 1 | DrugBank | validated |
| MOL000472 | emodin | Matrix metalloproteinase-9 | N/A | validated |
| MOL000472 | emodin | Pro-epidermal growth factor | DrugBank | validated |
| MOL000472 | emodin | Tumor necrosis factor | DrugBank | validated |
| MOL000472 | emodin | Caspase-3 | N/A | validated |
| MOL000472 | emodin | Cellular tumor antigen p53 | DrugBank | validated |
| MOL000472 | emodin | Protein kinase C epsilon type | N/A | validated |
| MOL000472 | emodin | Interstitial collagenase | DrugBank | validated |
| MOL000472 | emodin | Peroxisome proliferator-activated receptor gamma | N/A | validated |
| MOL000472 | emodin | Myc proto-oncogene protein | N/A | validated |
| MOL000472 | emodin | Cytochrome P450 1A1 | N/A | validated |
| MOL000472 | emodin | Interleukin-1 beta | DrugBank | validated |
| MOL000472 | emodin | Protein kinase C delta type | N/A | validated |
| MOL000472 | emodin | Granulocyte-macrophage colony-stimulating factor | N/A | validated |
| MOL000472 | emodin | Transforming growth factor beta-1 | N/A | validated |
| MOL000472 | emodin | Actin, aortic smooth muscle | N/A | validated |
| MOL000472 | emodin | Amine oxidase [flavin-containing] B | DrugBank | validated |
| MOL000472 | emodin | Tyrosine-protein kinase BTK | DrugBank | validated |
| MOL000472 | emodin | Solute carrier family 2, facilitated glucose transporter member 4 | N/A | validated |
| MOL000472 | emodin | Vascular endothelial growth factor receptor 3 | DrugBank | validated |
| MOL000472 | emodin | Solute carrier family 2, facilitated glucose transporter member 1 | N/A | validated |
| MOL000476 | Physcion | Prostaglandin G/H synthase 1 | DrugBank |  |
| MOL000476 | Physcion | Sodium channel protein type 5 subunit alpha | DrugBank |  |
| MOL000476 | Physcion | Prostaglandin G/H synthase 2 | DrugBank |  |
| MOL000476 | Physcion | Nitric-oxide synthase, endothelial | DrugBank |  |
| MOL000476 | Physcion | Coagulation factor VII | DrugBank |  |
| MOL000476 | Physcion | DNA topoisomerase II | DrugBank |  |
| MOL000476 | Physcion | Heat shock protein HSP 90 | DrugBank |  |
| MOL000476 | Physcion | Phosphatidylinositol-4,5-bisphosphate 3-kinase catalytic subunit, gamma isoform | DrugBank |  |
| MOL000476 | Physcion | Beta-lactamase | DrugBank |  |
| MOL000476 | Physcion | Ig gamma-1 chain C region | DrugBank |  |
| MOL000476 | Physcion | Nuclear receptor coactivator 2 | DrugBank |  |
| MOL000476 | Physcion | Nuclear receptor coactivator 1 | DrugBank |  |
| MOL000476 | Physcion | cAMP-dependent protein kinase inhibitor alpha | DrugBank |  |
| MOL000476 | Physcion | Calmodulin | DrugBank |  |
| MOL000476 | Physcion | mRNA of PKA Catalytic Subunit C-alpha | DrugBank |  |
| MOL000476 | Physcion | Coagulation factor Xa | DrugBank |  |
| MOL000476 | Physcion | Retinoic acid receptor RXR-alpha | DrugBank |  |
| MOL000492 | (+)-catechin | Prostaglandin G/H synthase 1 | DrugBank |  |
| MOL000492 | (+)-catechin | Estrogen receptor | DrugBank |  |
| MOL000492 | (+)-catechin | Prostaglandin G/H synthase 2 | DrugBank |  |
| MOL000492 | (+)-catechin | Heat shock protein HSP 90 | DrugBank |  |
| MOL000492 | (+)-catechin | Beta-lactamase | DrugBank |  |
| MOL000492 | (+)-catechin | mRNA of PKA Catalytic Subunit C-alpha | DrugBank |  |
| MOL000492 | (+)-catechin | Nuclear receptor coactivator 2 | DrugBank |  |
| MOL000492 | (+)-catechin | Calmodulin | DrugBank |  |
| MOL000492 | (+)-catechin | Retinoic acid receptor RXR-alpha | DrugBank |  |
| MOL000492 | (+)-catechin | Catalase | DrugBank | validated |
| MOL000492 | (+)-catechin | Hyaluronan synthase 2 | N/A | validated |
| MOL000513 | 3,4,5-trihydroxybenzoic acid | Prostaglandin G/H synthase 1 | DrugBank |  |
| MOL000513 | 3,4,5-trihydroxybenzoic acid | Prostaglandin G/H synthase 2 | DrugBank |  |
| MOL000513 | 3,4,5-trihydroxybenzoic acid | Amine oxidase [flavin-containing] B | DrugBank |  |
| MOL000513 | 3,4,5-trihydroxybenzoic acid | Progesterone receptor | DrugBank |  |
| MOL000513 | 3,4,5-trihydroxybenzoic acid | mRNA of Protein-tyrosine phosphatase, non-receptor type 1 | DrugBank |  |
| MOL000513 | 3,4,5-trihydroxybenzoic acid | DNA topoisomerase II | DrugBank |  |
| MOL000513 | 3,4,5-trihydroxybenzoic acid | Heat shock protein HSP 90 | DrugBank |  |
| MOL000513 | 3,4,5-trihydroxybenzoic acid | Phosphatidylinositol-4,5-bisphosphate 3-kinase catalytic subunit, gamma isoform | DrugBank |  |
| MOL000513 | 3,4,5-trihydroxybenzoic acid | Caspase-9 | N/A | validated |
| MOL000513 | 3,4,5-trihydroxybenzoic acid | Caspase-3 | N/A | validated |
| MOL000513 | 3,4,5-trihydroxybenzoic acid | Cellular tumor antigen p53 | DrugBank | validated |
| MOL000513 | 3,4,5-trihydroxybenzoic acid | Fatty acid synthase | DrugBank | validated |
| MOL000513 | 3,4,5-trihydroxybenzoic acid | Tumor necrosis factor ligand superfamily member 6 | N/A | validated |
| MOL000513 | 3,4,5-trihydroxybenzoic acid | Microsomal glutathione S-transferase 1 | DrugBank | validated |
| MOL000513 | 3,4,5-trihydroxybenzoic acid | Cytochrome P450 3A43 | N/A | validated |
| MOL005290 | 3,5-Dimethyl-p-anisic acid | Prostaglandin G/H synthase 1 | DrugBank |  |
| MOL005290 | 3,5-Dimethyl-p-anisic acid | Muscarinic acetylcholine receptor M1 | DrugBank |  |
| MOL005290 | 3,5-Dimethyl-p-anisic acid | Beta-1 adrenergic receptor | DrugBank |  |
| MOL005290 | 3,5-Dimethyl-p-anisic acid | Prostaglandin G/H synthase 2 | DrugBank |  |
| MOL005290 | 3,5-Dimethyl-p-anisic acid | Alpha-2A adrenergic receptor | DrugBank |  |
| MOL005290 | 3,5-Dimethyl-p-anisic acid | Alpha-2C adrenergic receptor | DrugBank |  |
| MOL005290 | 3,5-Dimethyl-p-anisic acid | Sodium-dependent noradrenaline transporter | DrugBank |  |
| MOL005290 | 3,5-Dimethyl-p-anisic acid | Alpha-1A adrenergic receptor | DrugBank |  |
| MOL005290 | 3,5-Dimethyl-p-anisic acid | Muscarinic acetylcholine receptor M2 | DrugBank |  |
| MOL005290 | 3,5-Dimethyl-p-anisic acid | Alpha-1B adrenergic receptor | DrugBank |  |
| MOL005290 | 3,5-Dimethyl-p-anisic acid | Sodium-dependent dopamine transporter | DrugBank |  |
| MOL005290 | 3,5-Dimethyl-p-anisic acid | Beta-2 adrenergic receptor | DrugBank |  |
| MOL005290 | 3,5-Dimethyl-p-anisic acid | Amine oxidase [flavin-containing] B | DrugBank |  |
| MOL005290 | 3,5-Dimethyl-p-anisic acid | Amine oxidase [flavin-containing] A | DrugBank |  |
| MOL000558 | quercetin-3-L-arabinon-7-D-glucoside | DNA topoisomerase II | DrugBank |  |
| MOL000006 | luteolin | Prostaglandin G/H synthase 1 | DrugBank |  |
| MOL000006 | luteolin | Androgen receptor | DrugBank | validated |
| MOL000006 | luteolin | Prostaglandin G/H synthase 2 | DrugBank | validated |
| MOL000006 | luteolin | Heat shock protein HSP 90 | DrugBank |  |
| MOL000006 | luteolin | Trypsin-1 | DrugBank |  |
| MOL000006 | luteolin | Nuclear receptor coactivator 2 | DrugBank |  |
| MOL000006 | luteolin | mRNA of PKA Catalytic Subunit C-alpha | DrugBank |  |
| MOL000006 | luteolin | Dipeptidyl peptidase IV | DrugBank |  |
| MOL000006 | luteolin | Phosphatidylinositol-4,5-bisphosphate 3-kinase catalytic subunit, gamma isoform | DrugBank |  |
| MOL000006 | luteolin | Transcription factor p65 | N/A | validated |
| MOL000006 | luteolin | Epidermal growth factor receptor | DrugBank | validated |
| MOL000006 | luteolin | RAC-alpha serine/threonine-protein kinase | N/A | validated |
| MOL000006 | luteolin | Vascular endothelial growth factor A | DrugBank | validated |
| MOL000006 | luteolin | G1/S-specific cyclin-D1 | N/A | validated |
| MOL000006 | luteolin | Bcl-2-like protein 1 | N/A | validated |
| MOL000006 | luteolin | Cyclin-dependent kinase inhibitor 1 | N/A | validated |
| MOL000006 | luteolin | Caspase-9 | N/A | validated |
| MOL000006 | luteolin | 72 kDa type IV collagenase | DrugBank | validated |
| MOL000006 | luteolin | Matrix metalloproteinase-9 | N/A | validated |
| MOL000006 | luteolin | Mitogen-activated protein kinase 1 | DrugBank | validated |
| MOL000006 | luteolin | Interleukin-10 | N/A | validated |
| MOL000006 | luteolin | Retinoblastoma-associated protein | DrugBank | validated |
| MOL000006 | luteolin | Cell division protein kinase 4 | DrugBank | validated |
| MOL000006 | luteolin | Tumor necrosis factor | DrugBank | validated |
| MOL000006 | luteolin | Transcription factor AP-1 | DrugBank | validated |
| MOL000006 | luteolin | Interleukin-6 | DrugBank | validated |
| MOL000006 | luteolin | Caspase-3 | N/A | validated |
| MOL000006 | luteolin | Cellular tumor antigen p53 | DrugBank | validated |
| MOL000006 | luteolin | NF-kappa-B inhibitor alpha | N/A | validated |
| MOL000006 | luteolin | Xanthine dehydrogenase/oxidase | DrugBank | validated |
| MOL000006 | luteolin | DNA topoisomerase 1 | DrugBank | validated |
| MOL000006 | luteolin | E3 ubiquitin-protein ligase Mdm2 | N/A | validated |
| MOL000006 | luteolin | Amyloid beta A4 protein | DrugBank | validated |
| MOL000006 | luteolin | Interstitial collagenase | DrugBank | validated |
| MOL000006 | luteolin | Proliferating cell nuclear antigen | N/A | validated |
| MOL000006 | luteolin | Receptor tyrosine-protein kinase erbB-2 | N/A | validated |
| MOL000006 | luteolin | Peroxisome proliferator-activated receptor gamma | N/A | validated |
| MOL000006 | luteolin | Heme oxygenase 1 | DrugBank | validated |
| MOL000006 | luteolin | Caspase-7 | DrugBank | validated |
| MOL000006 | luteolin | Intercellular adhesion molecule 1 | N/A | validated |
| MOL000006 | luteolin | Induced myeloid leukemia cell differentiation protein Mcl-1 | N/A | validated |
| MOL000006 | luteolin | Baculoviral IAP repeat-containing protein 5 | N/A | validated |
| MOL000006 | luteolin | Interleukin-2 | DrugBank | validated |
| MOL000006 | luteolin | G2/mitotic-specific cyclin-B1 | N/A | validated |
| MOL000006 | luteolin | Tyrosinase | N/A | validated |
| MOL000006 | luteolin | Interferon gamma | DrugBank | validated |
| MOL000006 | luteolin | Interleukin-4 | N/A | validated |
| MOL000006 | luteolin | DNA topoisomerase 2-alpha | N/A | validated |
| MOL000006 | luteolin | Glutathione S-transferase P | DrugBank | validated |
| MOL000006 | luteolin | Baculoviral IAP repeat-containing protein 4 | N/A | validated |
| MOL000006 | luteolin | Solute carrier family 2, facilitated glucose transporter member 4 | N/A | validated |
| MOL000006 | luteolin | Insulin receptor | DrugBank | validated |
| MOL000006 | luteolin | CD40 ligand | N/A | validated |
| MOL000006 | luteolin | Prostaglandin E synthase | N/A | validated |
| MOL000006 | luteolin | Kinetochore protein Nuf2 | N/A | validated |
| MOL000006 | luteolin | Adenylate cyclase type 2 | N/A | validated |
| MOL000006 | luteolin | Hepatocyte growth factor receptor | DrugBank | validated |
| MOL006469 | Morkit | Prostaglandin G/H synthase 1 | DrugBank |  |
| MOL006469 | Morkit | Sodium channel protein type 5 subunit alpha | DrugBank |  |
| MOL006469 | Morkit | Prostaglandin G/H synthase 2 | DrugBank |  |
| MOL006469 | Morkit | Amine oxidase [flavin-containing] B | DrugBank |  |
| MOL006488 | Questin | Prostaglandin G/H synthase 1 | DrugBank |  |
| MOL006488 | Questin | Sodium channel protein type 5 subunit alpha | DrugBank |  |
| MOL006488 | Questin | Prostaglandin G/H synthase 2 | DrugBank |  |
| MOL006488 | Questin | Nitric-oxide synthase, endothelial | DrugBank |  |
| MOL006488 | Questin | Retinoic acid receptor RXR-alpha | DrugBank |  |
| MOL006488 | Questin | Alpha-1B adrenergic receptor | DrugBank |  |
| MOL006488 | Questin | DNA topoisomerase II | DrugBank |  |
| MOL006488 | Questin | Heat shock protein HSP 90 | DrugBank |  |
| MOL006488 | Questin | Phosphatidylinositol-4,5-bisphosphate 3-kinase catalytic subunit, gamma isoform | DrugBank |  |
| MOL006488 | Questin | Ig gamma-1 chain C region | DrugBank |  |
| MOL006488 | Questin | Nuclear receptor coactivator 2 | DrugBank |  |
| MOL006488 | Questin | Nuclear receptor coactivator 1 | DrugBank |  |
| MOL006488 | Questin | cAMP-dependent protein kinase inhibitor alpha | DrugBank |  |
| MOL007387 | 2-MOHYDROP | Alcohol dehydrogenase 1C | DrugBank |  |
| MOL007387 | 2-MOHYDROP | Lysozyme | DrugBank |  |
| MOL007387 | 2-MOHYDROP | Nicotinate-nucleotide--dimethylbenzimidazole phosphoribosyltransferase | DrugBank |  |
| MOL007387 | 2-MOHYDROP | Amine oxidase [flavin-containing] B | DrugBank |  |
| MOL007954 | GRE | Prostaglandin G/H synthase 1 | DrugBank |  |
| MOL007954 | GRE | Arachidonate 5-lipoxygenase | DrugBank |  |
| MOL007954 | GRE | Prostaglandin G/H synthase 2 | DrugBank |  |
| MOL007954 | GRE | Amine oxidase [flavin-containing] B | DrugBank |  |
| MOL007954 | GRE | Lysozyme | DrugBank |  |
| MOL007954 | GRE | Chymotrypsinogen B | DrugBank |  |
| MOL007954 | GRE | Nicotinate-nucleotide--dimethylbenzimidazole phosphoribosyltransferase | DrugBank |  |
| MOL007954 | GRE | Alcohol dehydrogenase 1C | DrugBank |  |
| MOL000008 | apigenin | Prostaglandin G/H synthase 1 | DrugBank |  |
| MOL000008 | apigenin | Androgen receptor | DrugBank | validated |
| MOL000008 | apigenin | Prostaglandin G/H synthase 2 | DrugBank | validated |
| MOL000008 | apigenin | Heat shock protein HSP 90 | DrugBank |  |
| MOL000008 | apigenin | Trypsin-1 | DrugBank |  |
| MOL000008 | apigenin | Nuclear receptor coactivator 2 | DrugBank |  |
| MOL000008 | apigenin | Phosphatidylinositol-4,5-bisphosphate 3-kinase catalytic subunit, gamma isoform | DrugBank |  |
| MOL000008 | apigenin | mRNA of PKA Catalytic Subunit C-alpha | DrugBank |  |
| MOL000008 | apigenin | Sodium channel protein type 5 subunit alpha | DrugBank |  |
| MOL000008 | apigenin | Coagulation factor Xa | DrugBank |  |
| MOL000008 | apigenin | Coagulation factor VII | DrugBank |  |
| MOL000008 | apigenin | DNA topoisomerase II | DrugBank |  |
| MOL000008 | apigenin | Dipeptidyl peptidase IV | DrugBank |  |
| MOL000008 | apigenin | Calmodulin | DrugBank |  |
| MOL000008 | apigenin | Transcription factor p65 | N/A | validated |
| MOL000008 | apigenin | RAC-alpha serine/threonine-protein kinase | N/A | validated |
| MOL000008 | apigenin | Vascular endothelial growth factor A | DrugBank | validated |
| MOL000008 | apigenin | G1/S-specific cyclin-D1 | N/A | validated |
| MOL000008 | apigenin | Apoptosis regulator Bcl-2 | DrugBank | validated |
| MOL000008 | apigenin | Bcl-2-like protein 1 | N/A | validated |
| MOL000008 | apigenin | Proto-oncogene c-Fos | N/A | validated |
| MOL000008 | apigenin | Cyclin-dependent kinase inhibitor 1 | N/A | validated |
| MOL000008 | apigenin | Eukaryotic translation initiation factor 6 | N/A | validated |
| MOL000008 | apigenin | Apoptosis regulator BAX | N/A | validated |
| MOL000008 | apigenin | Caspase-9 | N/A | validated |
| MOL000008 | apigenin | Urokinase-type plasminogen activator | DrugBank | validated |
| MOL000008 | apigenin | Matrix metalloproteinase-9 | N/A | validated |
| MOL000008 | apigenin | Retinoblastoma-associated protein | DrugBank | validated |
| MOL000008 | apigenin | Cell division protein kinase 4 | DrugBank | validated |
| MOL000008 | apigenin | Tumor necrosis factor | DrugBank | validated |
| MOL000008 | apigenin | Transcription factor AP-1 | DrugBank | validated |
| MOL000008 | apigenin | Cell division protein kinase 6 | DrugBank | validated |
| MOL000008 | apigenin | Cyclin-dependent kinase inhibitor 2A, isoforms 1/2/3 | N/A | validated |
| MOL000008 | apigenin | Eukaryotic translation elongation factor 1 epsilon-1 | N/A | validated |
| MOL000008 | apigenin | Activator of 90 kDa heat shock protein ATPase homolog 1 | N/A | validated |
| MOL000008 | apigenin | Caspase-3 | N/A | validated |
| MOL000008 | apigenin | Cellular tumor antigen p53 | DrugBank | validated |
| MOL000008 | apigenin | NF-kappa-B inhibitor alpha | N/A | validated |
| MOL000008 | apigenin | Ornithine decarboxylase | DrugBank | validated |
| MOL000008 | apigenin | E3 ubiquitin-protein ligase Mdm2 | N/A | validated |
| MOL000008 | apigenin | Bcl2 antagonist of cell death | N/A | validated |
| MOL000008 | apigenin | Interstitial collagenase | DrugBank | validated |
| MOL000008 | apigenin | Hypoxia-inducible factor 1-alpha | N/A | validated |
| MOL000008 | apigenin | Insulin-like growth factor 1 receptor | N/A | validated |
| MOL000008 | apigenin | Protein CBFA2T1 | N/A | validated |
| MOL000008 | apigenin | Probable E3 ubiquitin-protein ligase HERC5 | N/A | validated |
| MOL000008 | apigenin | Cell division control protein 2 homolog | DrugBank | validated |
| MOL000008 | apigenin | Acetyl-CoA carboxylase 1 | DrugBank | validated |
| MOL000008 | apigenin | Heme oxygenase 1 | DrugBank | validated |
| MOL000008 | apigenin | Intercellular adhesion molecule 1 | N/A | validated |
| MOL000008 | apigenin | Induced myeloid leukemia cell differentiation protein Mcl-1 | N/A | validated |
| MOL000008 | apigenin | G1/S-specific cyclin-D2 | N/A | validated |
| MOL000008 | apigenin | Interleukin-2 | DrugBank | validated |
| MOL000008 | apigenin | G2/mitotic-specific cyclin-B1 | N/A | validated |
| MOL000008 | apigenin | Plasminogen activator inhibitor 1 | N/A | validated |
| MOL000008 | apigenin | Interferon gamma | DrugBank | validated |
| MOL000008 | apigenin | Interleukin-4 | N/A | validated |
| MOL000008 | apigenin | NF-kappa-B essential modulator | N/A | validated |
| MOL000008 | apigenin | Cytochrome P450 19A1 | N/A | validated |
| MOL000008 | apigenin | Baculoviral IAP repeat-containing protein 4 | N/A | validated |
| MOL000008 | apigenin | 26S proteasome non-ATPase regulatory subunit 3 | N/A | validated |
| MOL000008 | apigenin | Solute carrier family 2, facilitated glucose transporter member 4 | N/A | validated |
| MOL000008 | apigenin | Insulin receptor | DrugBank | validated |
| MOL000008 | apigenin | CD40 ligand | N/A | validated |
| MOL000008 | apigenin | Cytochrome c | DrugBank | validated |
| MOL000008 | apigenin | CASP8 and FADD-like apoptosis regulator | N/A | validated |
| MOL000008 | apigenin | Alpha- and gamma-adaptin-binding protein p34 | N/A | validated |
| MOL000008 | apigenin | Insulin | DrugBank | validated |
| MOL000008 | apigenin | Low affinity immunoglobulin epsilon Fc receptor | N/A | validated |
| MOL000008 | apigenin | Interleukin-13 | N/A | validated |
| MOL000008 | apigenin | High affinity immunoglobulin epsilon receptor subunit beta | DrugBank | validated |
| MOL000008 | apigenin | Intestinal-type alkaline phosphatase | N/A | validated |
| MOL000008 | apigenin | Proteasome activator complex subunit 3 | N/A | validated |
| MOL000008 | apigenin | Glucose-6-phosphatase | N/A | validated |
| MOL000008 | apigenin | Adenomatous polyposis coli protein | N/A | validated |
| MOL000008 | apigenin | Transient receptor potential cation channel subfamily M member 2 | N/A | validated |
| MOL000008 | apigenin | Aldo-keto reductase family 1 member C3 | DrugBank | validated |
| MOL000008 | apigenin | Sodium/iodide cotransporter | N/A | validated |
| MOL000008 | apigenin | Sodium/potassium-transporting ATPase subunit gamma | N/A | validated |
| MOL000008 | apigenin | Dolichyl-phosphate beta-glucosyltransferase | N/A | validated |
| MOL000009 | luteolin-7-o-glucoside | Trypsin-1 | DrugBank |  |
| MOL000009 | luteolin-7-o-glucoside | Nitric oxide synthase, inducible | DrugBank | validated |
| MOL000009 | luteolin-7-o-glucoside | Prostaglandin G/H synthase 2 | DrugBank | validated |
| MOL009070 | Luteolin 7-O-glucuronide | Coagulation factor Xa | DrugBank |  |
| MOL009070 | Luteolin 7-O-glucuronide | mRNA of Protein-tyrosine phosphatase, non-receptor type 1 | DrugBank |  |
| MOL009070 | Luteolin 7-O-glucuronide | DNA topoisomerase II | DrugBank |  |
| MOL000098 | quercetin | Prostaglandin G/H synthase 1 | DrugBank | validated |
| MOL000098 | quercetin | Androgen receptor | DrugBank | validated |
| MOL000098 | quercetin | Peroxisome proliferator activated receptor gamma | DrugBank |  |
| MOL000098 | quercetin | Prostaglandin G/H synthase 2 | DrugBank | validated |
| MOL000098 | quercetin | Heat shock protein HSP 90 | DrugBank |  |
| MOL000098 | quercetin | Phosphatidylinositol-4,5-bisphosphate 3-kinase catalytic subunit, gamma isoform | DrugBank |  |
| MOL000098 | quercetin | Nuclear receptor coactivator 2 | DrugBank |  |
| MOL000098 | quercetin | Dipeptidyl peptidase IV | DrugBank |  |
| MOL000098 | quercetin | Aldose reductase | DrugBank |  |
| MOL000098 | quercetin | Trypsin-1 | DrugBank |  |
| MOL000098 | quercetin | DNA topoisomerase II | DrugBank |  |
| MOL000098 | quercetin | Thrombin | DrugBank |  |
| MOL000098 | quercetin | Potassium voltage-gated channel subfamily H member 2 | DrugBank |  |
| MOL000098 | quercetin | Sodium channel protein type 5 subunit alpha | DrugBank |  |
| MOL000098 | quercetin | Coagulation factor Xa | DrugBank |  |
| MOL000098 | quercetin | Beta-2 adrenergic receptor | DrugBank |  |
| MOL000098 | quercetin | Stromelysin-1 | DrugBank |  |
| MOL000098 | quercetin | mRNA of PKA Catalytic Subunit C-alpha | DrugBank |  |
| MOL000098 | quercetin | Coagulation factor VII | DrugBank |  |
| MOL000098 | quercetin | Nitric-oxide synthase, endothelial | DrugBank |  |
| MOL000098 | quercetin | Retinoic acid receptor RXR-alpha | DrugBank |  |
| MOL000098 | quercetin | Acetylcholinesterase | DrugBank |  |
| MOL000098 | quercetin | Gamma-aminobutyric acid receptor subunit alpha-1 | DrugBank |  |
| MOL000098 | quercetin | Amine oxidase [flavin-containing] B | DrugBank |  |
| MOL000098 | quercetin | Transcription factor p65 | N/A | validated |
| MOL000098 | quercetin | Epidermal growth factor receptor | DrugBank | validated |
| MOL000098 | quercetin | RAC-alpha serine/threonine-protein kinase | N/A | validated |
| MOL000098 | quercetin | Vascular endothelial growth factor A | DrugBank | validated |
| MOL000098 | quercetin | G1/S-specific cyclin-D1 | N/A | validated |
| MOL000098 | quercetin | Apoptosis regulator Bcl-2 | DrugBank | validated |
| MOL000098 | quercetin | Bcl-2-like protein 1 | N/A | validated |
| MOL000098 | quercetin | Proto-oncogene c-Fos | N/A | validated |
| MOL000098 | quercetin | Cyclin-dependent kinase inhibitor 1 | N/A | validated |
| MOL000098 | quercetin | Eukaryotic translation initiation factor 6 | N/A | validated |
| MOL000098 | quercetin | Apoptosis regulator BAX | N/A | validated |
| MOL000098 | quercetin | Caspase-9 | N/A | validated |
| MOL000098 | quercetin | Urokinase-type plasminogen activator | DrugBank | validated |
| MOL000098 | quercetin | 72 kDa type IV collagenase | DrugBank | validated |
| MOL000098 | quercetin | Matrix metalloproteinase-9 | N/A | validated |
| MOL000098 | quercetin | Mitogen-activated protein kinase 1 | DrugBank | validated |
| MOL000098 | quercetin | Interleukin-10 | N/A | validated |
| MOL000098 | quercetin | Pro-epidermal growth factor | DrugBank | validated |
| MOL000098 | quercetin | Retinoblastoma-associated protein | DrugBank | validated |
| MOL000098 | quercetin | Tumor necrosis factor | DrugBank | validated |
| MOL000098 | quercetin | Transcription factor AP-1 | DrugBank | validated |
| MOL000098 | quercetin | Interleukin-6 | DrugBank | validated |
| MOL000098 | quercetin | Cyclin-dependent kinase inhibitor 2A, isoforms 1/2/3 | N/A | validated |
| MOL000098 | quercetin | Activator of 90 kDa heat shock protein ATPase homolog 1 | N/A | validated |
| MOL000098 | quercetin | Caspase-3 | N/A | validated |
| MOL000098 | quercetin | Cellular tumor antigen p53 | DrugBank | validated |
| MOL000098 | quercetin | ETS domain-containing protein Elk-1 | N/A | validated |
| MOL000098 | quercetin | NF-kappa-B inhibitor alpha | N/A | validated |
| MOL000098 | quercetin | NADPH--cytochrome P450 reductase | DrugBank | validated |
| MOL000098 | quercetin | Ornithine decarboxylase | DrugBank | validated |
| MOL000098 | quercetin | Xanthine dehydrogenase/oxidase | DrugBank | validated |
| MOL000098 | quercetin | Caspase-8 | N/A | validated |
| MOL000098 | quercetin | DNA topoisomerase 1 | DrugBank | validated |
| MOL000098 | quercetin | RAF proto-oncogene serine/threonine-protein kinase | N/A | validated |
| MOL000098 | quercetin | Superoxide dismutase [Cu-Zn] | DrugBank | validated |
| MOL000098 | quercetin | Protein kinase C alpha type | N/A | validated |
| MOL000098 | quercetin | Interstitial collagenase | DrugBank | validated |
| MOL000098 | quercetin | Hypoxia-inducible factor 1-alpha | N/A | validated |
| MOL000098 | quercetin | Signal transducer and activator of transcription 1-alpha/beta | N/A | validated |
| MOL000098 | quercetin | Protein CBFA2T1 | N/A | validated |
| MOL000098 | quercetin | Probable E3 ubiquitin-protein ligase HERC5 | N/A | validated |
| MOL000098 | quercetin | Cell division control protein 2 homolog | DrugBank | validated |
| MOL000098 | quercetin | 78 kDa glucose-regulated protein | DrugBank | validated |
| MOL000098 | quercetin | Receptor tyrosine-protein kinase erbB-2 | N/A | validated |
| MOL000098 | quercetin | Peroxisome proliferator-activated receptor gamma | N/A | validated |
| MOL000098 | quercetin | Acetyl-CoA carboxylase 1 | DrugBank | validated |
| MOL000098 | quercetin | Heme oxygenase 1 | DrugBank | validated |
| MOL000098 | quercetin | Cytochrome P450 3A4 | DrugBank | validated |
| MOL000098 | quercetin | Cytochrome P450 1A2 | DrugBank | validated |
| MOL000098 | quercetin | Caveolin-1 | N/A | validated |
| MOL000098 | quercetin | Myc proto-oncogene protein | N/A | validated |
| MOL000098 | quercetin | Tissue factor | DrugBank | validated |
| MOL000098 | quercetin | Gap junction alpha-1 protein | DrugBank | validated |
| MOL000098 | quercetin | Cytochrome P450 1A1 | N/A | validated |
| MOL000098 | quercetin | Intercellular adhesion molecule 1 | N/A | validated |
| MOL000098 | quercetin | Interleukin-1 beta | DrugBank | validated |
| MOL000098 | quercetin | C-C motif chemokine 2 | DrugBank | validated |
| MOL000098 | quercetin | E-selectin | DrugBank | validated |
| MOL000098 | quercetin | Vascular cell adhesion protein 1 | DrugBank | validated |
| MOL000098 | quercetin | Prostaglandin E2 receptor EP3 subtype | DrugBank | validated |
| MOL000098 | quercetin | Interleukin-8 | N/A | validated |
| MOL000098 | quercetin | Protein kinase C beta type | N/A | validated |
| MOL000098 | quercetin | Baculoviral IAP repeat-containing protein 5 | N/A | validated |
| MOL000098 | quercetin | Dual oxidase 2 | N/A | validated |
| MOL000098 | quercetin | Nitric oxide synthase, endothelial | N/A | validated |
| MOL000098 | quercetin | Heat shock protein beta-1 | N/A | validated |
| MOL000098 | quercetin | Transforming growth factor beta-1 | N/A | validated |
| MOL000098 | quercetin | Estrogen sulfotransferase | DrugBank | validated |
| MOL000098 | quercetin | Maltase-glucoamylase, intestinal | DrugBank | validated |
| MOL000098 | quercetin | Interleukin-2 | DrugBank | validated |
| MOL000098 | quercetin | Nuclear receptor subfamily 1 group I member 2 | N/A | validated |
| MOL000098 | quercetin | Cytochrome P450 1B1 | N/A | validated |
| MOL000098 | quercetin | G2/mitotic-specific cyclin-B1 | N/A | validated |
| MOL000098 | quercetin | Tissue-type plasminogen activator | DrugBank | validated |
| MOL000098 | quercetin | Thrombomodulin | DrugBank | validated |
| MOL000098 | quercetin | Plasminogen activator inhibitor 1 | N/A | validated |
| MOL000098 | quercetin | Collagen alpha-1(I) chain | DrugBank | validated |
| MOL000098 | quercetin | Interferon gamma | DrugBank | validated |
| MOL000098 | quercetin | Arachidonate 5-lipoxygenase | DrugBank | validated |
| MOL000098 | quercetin | Phosphatidylinositol-3,4,5-trisphosphate 3-phosphatase and dual-specificity protein phosphatase PTEN | N/A | validated |
| MOL000098 | quercetin | Interleukin-1 alpha | N/A | validated |
| MOL000098 | quercetin | Myeloperoxidase | DrugBank | validated |
| MOL000098 | quercetin | DNA topoisomerase 2-alpha | N/A | validated |
| MOL000098 | quercetin | Neutrophil cytosol factor 1 | N/A | validated |
| MOL000098 | quercetin | ATP-binding cassette sub-family G member 2 | N/A | validated |
| MOL000098 | quercetin | Hyaluronan synthase 2 | N/A | validated |
| MOL000098 | quercetin | Glutathione S-transferase P | DrugBank | validated |
| MOL000098 | quercetin | Nuclear factor erythroid 2-related factor 2 | N/A | validated |
| MOL000098 | quercetin | NAD(P)H dehydrogenase [quinone] 1 | DrugBank | validated |
| MOL000098 | quercetin | Poly [ADP-ribose] polymerase 1 | N/A | validated |
| MOL000098 | quercetin | Aryl hydrocarbon receptor | DrugBank | validated |
| MOL000098 | quercetin | 26S proteasome non-ATPase regulatory subunit 3 | N/A | validated |
| MOL000098 | quercetin | Solute carrier family 2, facilitated glucose transporter member 4 | N/A | validated |
| MOL000098 | quercetin | Collagen alpha-1(III) chain | DrugBank | validated |
| MOL000098 | quercetin | DNA gyrase subunit B | DrugBank | validated |
| MOL000098 | quercetin | C-X-C motif chemokine 11 | N/A | validated |
| MOL000098 | quercetin | C-X-C motif chemokine 2 | N/A | validated |
| MOL000098 | quercetin | DDB1- and CUL4-associated factor 5 | N/A | validated |
| MOL000098 | quercetin | Nuclear receptor subfamily 1 group I member 3 | N/A | validated |
| MOL000098 | quercetin | Serine/threonine-protein kinase Chk2 | N/A | validated |
| MOL000098 | quercetin | Insulin receptor | DrugBank | validated |
| MOL000098 | quercetin | Claudin-4 | N/A | validated |
| MOL000098 | quercetin | Peroxisome proliferator-activated receptor alpha | N/A | validated |
| MOL000098 | quercetin | Peroxisome proliferator-activated receptor delta | N/A | validated |
| MOL000098 | quercetin | Heat shock factor protein 1 | N/A | validated |
| MOL000098 | quercetin | C-reactive protein | N/A | validated |
| MOL000098 | quercetin | C-X-C motif chemokine 10 | N/A | validated |
| MOL000098 | quercetin | Inhibitor of nuclear factor kappa-B kinase subunit alpha | N/A | validated |
| MOL000098 | quercetin | Osteopontin | N/A | validated |
| MOL000098 | quercetin | Runt-related transcription factor 2 | N/A | validated |
| MOL000098 | quercetin | Ras association domain-containing protein 1 | N/A | validated |
| MOL000098 | quercetin | Transcription factor E2F1 | N/A | validated |
| MOL000098 | quercetin | Transcription factor E2F2 | N/A | validated |
| MOL000098 | quercetin | Prostatic acid phosphatase | DrugBank | validated |
| MOL000098 | quercetin | Cathepsin D | DrugBank | validated |
| MOL000098 | quercetin | Insulin-like growth factor-binding protein 3 | N/A | validated |
| MOL000098 | quercetin | Insulin-like growth factor II | N/A | validated |
| MOL000098 | quercetin | CD40 ligand | N/A | validated |
| MOL000098 | quercetin | Interferon regulatory factor 1 | N/A | validated |
| MOL000098 | quercetin | Receptor tyrosine-protein kinase erbB-3 | N/A | validated |
| MOL000098 | quercetin | Serum paraoxonase/arylesterase 1 | DrugBank | validated |
| MOL000098 | quercetin | Type I iodothyronine deiodinase | N/A | validated |
| MOL000098 | quercetin | Procollagen C-endopeptidase enhancer 1 | N/A | validated |
| MOL000098 | quercetin | Puromycin-sensitive aminopeptidase | N/A | validated |
| MOL000098 | quercetin | Hexokinase-2 | N/A | validated |
| MOL000098 | quercetin | Homeobox protein Nkx-3.1 | N/A | validated |
| MOL000098 | quercetin | Ras GTPase-activating protein 1 | N/A | validated |
| MOL000098 | quercetin | Peroxidase C1A | N/A | validated |
| MOL000098 | quercetin | Glutathione S-transferase Mu 1 | DrugBank | validated |
| MOL000098 | quercetin | Glutathione S-transferase Mu 2 | DrugBank | validated |
