# Supplementary material for: Luteolin Isolated from Polygonum cuspidatum Is a Potential Compound against Nasopharyngeal Carcinoma
Source: Biomed Res Int. 2022 Dec 23;2022:9740066. doi: 10.1155/2022/9740066 (PMC9803567; doi:10.1155/2022/9740066)
Supplement: Supplementary Materials — Table S1: Basic information of the bioactive compounds of P. cuspidatum. Table S2: The targets for the bioactive compounds of P. cuspidatum in the TCMSP database. Table S3: The standard names of targets for the bioactive compounds of P. cuspidatum. Table S4: Basic information of the disease related targets for NPC. Table S5: The common targets of disease targets for NPC and bioactive compounds from P. cuspidatum. Table S6: GO analysis of common targets of drug compounds and diseases through the DAVID website. Table S7: KEGG pathway analysis of common targets of drug compounds and diseases through the DAVID website. Figure S1: Effect of different bioactive compounds on the survival rate of CNE2 cells in NPC. [file 9740066.f1.zip › Table 3S The standard names of targets for the bioactive compounds of P. cuspidatum.docx]

| MolId | MolName | Target | Gene Symbol |
| --- | --- | --- | --- |
| MOL000358 | beta-sitosterol | Progesterone receptor | PGR |
| MOL000358 | beta-sitosterol | Nuclear receptor coactivator 2 | NCOA2 |
| MOL000358 | beta-sitosterol | Prostaglandin G/H synthase 1 | PTGS1 |
| MOL000358 | beta-sitosterol | Prostaglandin G/H synthase 2 | PTGS2 |
| MOL000358 | beta-sitosterol | Potassium voltage-gated channel subfamily H member 2 | KCNH2 |
| MOL000358 | beta-sitosterol | Muscarinic acetylcholine receptor M3 | CHRM3 |
| MOL000358 | beta-sitosterol | Muscarinic acetylcholine receptor M1 | CHRM1 |
| MOL000358 | beta-sitosterol | Sodium channel protein type 5 subunit alpha | SCN5A |
| MOL000358 | beta-sitosterol | Muscarinic acetylcholine receptor M4 | CHRM4 |
| MOL000358 | beta-sitosterol | Alpha-1A adrenergic receptor | ADRA1A |
| MOL000358 | beta-sitosterol | Muscarinic acetylcholine receptor M2 | CHRM2 |
| MOL000358 | beta-sitosterol | Alpha-1B adrenergic receptor | ADRA1B |
| MOL000358 | beta-sitosterol | Beta-2 adrenergic receptor | ADRB2 |
| MOL000358 | beta-sitosterol | Neuronal acetylcholine receptor subunit alpha-2 | CHRNA2 |
| MOL000358 | beta-sitosterol | Sodium-dependent serotonin transporter | SLC6A4 |
| MOL000358 | beta-sitosterol | Mu-type opioid receptor | OPRM1 |
| MOL000358 | beta-sitosterol | Gamma-aminobutyric acid receptor subunit alpha-1 | GABRA1 |
| MOL000358 | beta-sitosterol | Apoptosis regulator Bcl-2 | BCL2 |
| MOL000358 | beta-sitosterol | Apoptosis regulator BAX | BAX |
| MOL000358 | beta-sitosterol | Caspase-9 | CASP9 |
| MOL000358 | beta-sitosterol | Transcription factor AP-1 | JUN |
| MOL000358 | beta-sitosterol | Caspase-3 | CASP3 |
| MOL000358 | beta-sitosterol | Caspase-8 | CASP8 |
| MOL000358 | beta-sitosterol | Protein kinase C alpha type | PRKCA |
| MOL000358 | beta-sitosterol | Serum paraoxonase/arylesterase 1 | PON1 |
| MOL000358 | beta-sitosterol | Microtubule-associated protein 2 | MAP2 |
| MOL000006 | luteolin | Prostaglandin G/H synthase 1 | PTGS1 |
| MOL000006 | luteolin | Androgen receptor | AR |
| MOL000006 | luteolin | Prostaglandin G/H synthase 2 | PTGS2 |
| MOL000006 | luteolin | Trypsin-1 | PRSS1 |
| MOL000006 | luteolin | Nuclear receptor coactivator 2 | NCOA2 |
| MOL000006 | luteolin | Transcription factor p65 | RELA |
| MOL000006 | luteolin | Epidermal growth factor receptor | EGFR |
| MOL000006 | luteolin | RAC-alpha serine/threonine-protein kinase | AKT1 |
| MOL000006 | luteolin | Vascular endothelial growth factor A | VEGFA |
| MOL000006 | luteolin | G1/S-specific cyclin-D1 | CCND1 |
| MOL000006 | luteolin | Bcl-2-like protein 1 | BCL2L1 |
| MOL000006 | luteolin | Cyclin-dependent kinase inhibitor 1 | CDKN1A |
| MOL000006 | luteolin | Caspase-9 | CASP9 |
| MOL000006 | luteolin | 72 kDa type IV collagenase | MMP2 |
| MOL000006 | luteolin | Matrix metalloproteinase-9 | MMP9 |
| MOL000006 | luteolin | Mitogen-activated protein kinase 1 | MAPK1 |
| MOL000006 | luteolin | Interleukin-10 | IL10 |
| MOL000006 | luteolin | Retinoblastoma-associated protein | RB1 |
| MOL000006 | luteolin | Tumor necrosis factor | TNFSF15 |
| MOL000006 | luteolin | Transcription factor AP-1 | JUN |
| MOL000006 | luteolin | Interleukin-6 | IL6 |
| MOL000006 | luteolin | Caspase-3 | CASP3 |
| MOL000006 | luteolin | Cellular tumor antigen p53 | TP63 |
| MOL000006 | luteolin | NF-kappa-B inhibitor alpha | NFKBIA |
| MOL000006 | luteolin | DNA topoisomerase 1 | TOP1 |
| MOL000006 | luteolin | E3 ubiquitin-protein ligase Mdm2 | MDM2 |
| MOL000006 | luteolin | Amyloid beta A4 protein | APP |
| MOL000006 | luteolin | Interstitial collagenase | MMP1 |
| MOL000006 | luteolin | Proliferating cell nuclear antigen | PCNA |
| MOL000006 | luteolin | Receptor tyrosine-protein kinase erbB-2 | ERBB2 |
| MOL000006 | luteolin | Peroxisome proliferator-activated receptor gamma | PPARG |
| MOL000006 | luteolin | Heme oxygenase 1 | HMOX1 |
| MOL000006 | luteolin | Caspase-7 | CASP7 |
| MOL000006 | luteolin | Intercellular adhesion molecule 1 | ICAM1 |
| MOL000006 | luteolin | Induced myeloid leukemia cell differentiation protein Mcl-1 | MCL1 |
| MOL000006 | luteolin | Baculoviral IAP repeat-containing protein 5 | BIRC5 |
| MOL000006 | luteolin | Interleukin-2 | IL2 |
| MOL000006 | luteolin | G2/mitotic-specific cyclin-B1 | CCNB1 |
| MOL000006 | luteolin | Tyrosinase | TYR |
| MOL000006 | luteolin | Interferon gamma | IFNG |
| MOL000006 | luteolin | Interleukin-4 | IL4 |
| MOL000006 | luteolin | DNA topoisomerase 2-alpha | TOP2A |
| MOL000006 | luteolin | Glutathione S-transferase P | GSTP1 |
| MOL000006 | luteolin | Solute carrier family 2, facilitated glucose transporter member 4 | SLC2A4 |
| MOL000006 | luteolin | Insulin receptor | INSR |
| MOL000006 | luteolin | CD40 ligand | CD40LG |
| MOL000006 | luteolin | Prostaglandin E synthase | PTGES |
| MOL000006 | luteolin | Kinetochore protein Nuf2 | NUF2 |
| MOL000006 | luteolin | Adenylate cyclase type 2 | ADCY2 |
| MOL000006 | luteolin | Hepatocyte growth factor receptor | MET |
| MOL000098 | quercetin | Prostaglandin G/H synthase 1 | PTGS1 |
| MOL000098 | quercetin | Androgen receptor | AR |
| MOL000098 | quercetin | Peroxisome proliferator activated receptor gamma | PPARG |
| MOL000098 | quercetin | Prostaglandin G/H synthase 2 | PTGS2 |
| MOL000098 | quercetin | Nuclear receptor coactivator 2 | NCOA2 |
| MOL000098 | quercetin | Aldose reductase | AKR1B1 |
| MOL000098 | quercetin | Trypsin-1 | PRSS1 |
| MOL000098 | quercetin | Potassium voltage-gated channel subfamily H member 2 | KCNH2 |
| MOL000098 | quercetin | Sodium channel protein type 5 subunit alpha | SCN5A |
| MOL000098 | quercetin | Beta-2 adrenergic receptor | ADRB2 |
| MOL000098 | quercetin | Stromelysin-1 | MMP3 |
| MOL000098 | quercetin | Coagulation factor VII | F7 |
| MOL000098 | quercetin | Retinoic acid receptor RXR-alpha | RXRA |
| MOL000098 | quercetin | Acetylcholinesterase | ACHE |
| MOL000098 | quercetin | Gamma-aminobutyric acid receptor subunit alpha-1 | GABRA1 |
| MOL000098 | quercetin | Amine oxidase [flavin-containing] B | MAOB |
| MOL000098 | quercetin | Transcription factor p65 | RELA |
| MOL000098 | quercetin | Epidermal growth factor receptor | EGFR |
| MOL000098 | quercetin | RAC-alpha serine/threonine-protein kinase | AKT1 |
| MOL000098 | quercetin | Vascular endothelial growth factor A | VEGFA |
| MOL000098 | quercetin | G1/S-specific cyclin-D1 | CCND1 |
| MOL000098 | quercetin | Apoptosis regulator Bcl-2 | BCL2 |
| MOL000098 | quercetin | Bcl-2-like protein 1 | BCL2L1 |
| MOL000098 | quercetin | Proto-oncogene c-Fos | FOS |
| MOL000098 | quercetin | Cyclin-dependent kinase inhibitor 1 | CDKN1A |
| MOL000098 | quercetin | Eukaryotic translation initiation factor 6 | EIF6 |
| MOL000098 | quercetin | Apoptosis regulator BAX | BAX |
| MOL000098 | quercetin | Caspase-9 | CASP9 |
| MOL000098 | quercetin | Urokinase-type plasminogen activator | PLAU |
| MOL000098 | quercetin | 72 kDa type IV collagenase | MMP2 |
| MOL000098 | quercetin | Matrix metalloproteinase-9 | MMP9 |
| MOL000098 | quercetin | Mitogen-activated protein kinase 1 | MAPK1 |
| MOL000098 | quercetin | Interleukin-10 | IL10 |
| MOL000098 | quercetin | Pro-epidermal growth factor | EGF |
| MOL000098 | quercetin | Retinoblastoma-associated protein | RB1 |
| MOL000098 | quercetin | Tumor necrosis factor | TNFSF15 |
| MOL000098 | quercetin | Transcription factor AP-1 | JUN |
| MOL000098 | quercetin | Interleukin-6 | IL6 |
| MOL000098 | quercetin | Activator of 90 kDa heat shock protein ATPase homolog 1 | AHSA1 |
| MOL000098 | quercetin | Caspase-3 | CASP3 |
| MOL000098 | quercetin | Cellular tumor antigen p53 | TP63 |
| MOL000098 | quercetin | ETS domain-containing protein Elk-1 | ELK1 |
| MOL000098 | quercetin | NF-kappa-B inhibitor alpha | NFKBIA |
| MOL000098 | quercetin | NADPH--cytochrome P450 reductase | POR |
| MOL000098 | quercetin | Ornithine decarboxylase | ODC1 |
| MOL000098 | quercetin | Caspase-8 | CASP8 |
| MOL000098 | quercetin | DNA topoisomerase 1 | TOP1 |
| MOL000098 | quercetin | RAF proto-oncogene serine/threonine-protein kinase | RAF1 |
| MOL000098 | quercetin | Superoxide dismutase [Cu-Zn] | SOD1 |
| MOL000098 | quercetin | Protein kinase C alpha type | PRKCA |
| MOL000098 | quercetin | Interstitial collagenase | MMP1 |
| MOL000098 | quercetin | Hypoxia-inducible factor 1-alpha | HIF1A |
| MOL000098 | quercetin | Signal transducer and activator of transcription 1-alpha/beta | STAT1 |
| MOL000098 | quercetin | Protein CBFA2T1 | RUNX1T1 |
| MOL000098 | quercetin | Receptor tyrosine-protein kinase erbB-2 | ERBB2 |
| MOL000098 | quercetin | Peroxisome proliferator-activated receptor gamma | PPARG |
| MOL000098 | quercetin | Acetyl-CoA carboxylase 1 | ACACA |
| MOL000098 | quercetin | Heme oxygenase 1 | HMOX1 |
| MOL000098 | quercetin | Cytochrome P450 3A4 | CYP3A4 |
| MOL000098 | quercetin | Cytochrome P450 1A2 | CYP1A2 |
| MOL000098 | quercetin | Caveolin-1 | CAV1 |
| MOL000098 | quercetin | Myc proto-oncogene protein | MYC |
| MOL000098 | quercetin | Tissue factor | F3 |
| MOL000098 | quercetin | Gap junction alpha-1 protein | GJA1 |
| MOL000098 | quercetin | Cytochrome P450 1A1 | CYP1A1 |
| MOL000098 | quercetin | Intercellular adhesion molecule 1 | ICAM1 |
| MOL000098 | quercetin | Interleukin-1 beta | IL1B |
| MOL000098 | quercetin | C-C motif chemokine 2 | CCL2 |
| MOL000098 | quercetin | E-selectin | SELE |
| MOL000098 | quercetin | Vascular cell adhesion protein 1 | VCAM1 |
| MOL000098 | quercetin | Prostaglandin E2 receptor EP3 subtype | PTGER3 |
| MOL000098 | quercetin | Interleukin-8 | CXCL8 |
| MOL000098 | quercetin | Protein kinase C beta type | PRKCB |
| MOL000098 | quercetin | Baculoviral IAP repeat-containing protein 5 | BIRC5 |
| MOL000098 | quercetin | Dual oxidase 2 | DUOX2 |
| MOL000098 | quercetin | Nitric oxide synthase, endothelial | NOS3 |
| MOL000098 | quercetin | Heat shock protein beta-1 | HSPB1 |
| MOL000098 | quercetin | Estrogen sulfotransferase | SULT1E1 |
| MOL000098 | quercetin | Maltase-glucoamylase, intestinal | MGAM |
| MOL000098 | quercetin | Interleukin-2 | IL2 |
| MOL000098 | quercetin | Nuclear receptor subfamily 1 group I member 2 | NR1I2 |
| MOL000098 | quercetin | Cytochrome P450 1B1 | CYP1B1 |
| MOL000098 | quercetin | G2/mitotic-specific cyclin-B1 | CCNB1 |
| MOL000098 | quercetin | Tissue-type plasminogen activator | PLAT |
| MOL000098 | quercetin | Thrombomodulin | THBD |
| MOL000098 | quercetin | Plasminogen activator inhibitor 1 | SERPINE1 |
| MOL000098 | quercetin | Collagen alpha-1(I) chain | COL1A1 |
| MOL000098 | quercetin | Interferon gamma | IFNG |
| MOL000098 | quercetin | Arachidonate 5-lipoxygenase | ALOX5 |
| MOL000098 | quercetin | Interleukin-1 alpha | IL1A |
| MOL000098 | quercetin | Myeloperoxidase | MPO |
| MOL000098 | quercetin | DNA topoisomerase 2-alpha | TOP2A |
| MOL000098 | quercetin | Neutrophil cytosol factor 1 | NCF1 |
| MOL000098 | quercetin | ATP-binding cassette sub-family G member 2 | ABCG2 |
| MOL000098 | quercetin | Hyaluronan synthase 2 | HAS2 |
| MOL000098 | quercetin | Glutathione S-transferase P | GSTP1 |
| MOL000098 | quercetin | Nuclear factor erythroid 2-related factor 2 | NFE2L2 |
| MOL000098 | quercetin | NAD(P)H dehydrogenase [quinone] 1 | NQO1 |
| MOL000098 | quercetin | Poly [ADP-ribose] polymerase 1 | PARP1 |
| MOL000098 | quercetin | Aryl hydrocarbon receptor | AHR |
| MOL000098 | quercetin | 26S proteasome non-ATPase regulatory subunit 3 | PSMD3 |
| MOL000098 | quercetin | Solute carrier family 2, facilitated glucose transporter member 4 | SLC2A4 |
| MOL000098 | quercetin | Collagen alpha-1(III) chain | COL3A1 |
| MOL000098 | quercetin | C-X-C motif chemokine 11 | CXCL11 |
| MOL000098 | quercetin | C-X-C motif chemokine 2 | CXCL2 |
| MOL000098 | quercetin | DDB1- and CUL4-associated factor 5 | DCAF5 |
| MOL000098 | quercetin | Nuclear receptor subfamily 1 group I member 3 | NR1I3 |
| MOL000098 | quercetin | Serine/threonine-protein kinase Chk2 | CHEK2 |
| MOL000098 | quercetin | Insulin receptor | INSR |
| MOL000098 | quercetin | Claudin-4 | CLDN4 |
| MOL000098 | quercetin | Peroxisome proliferator-activated receptor alpha | PPARA |
| MOL000098 | quercetin | Peroxisome proliferator-activated receptor delta | PPARD |
| MOL000098 | quercetin | Heat shock factor protein 1 | HSF1 |
| MOL000098 | quercetin | C-reactive protein | CRP |
| MOL000098 | quercetin | C-X-C motif chemokine 10 | CXCL10 |
| MOL000098 | quercetin | Inhibitor of nuclear factor kappa-B kinase subunit alpha | CHUK |
| MOL000098 | quercetin | Osteopontin | SPP1 |
| MOL000098 | quercetin | Runt-related transcription factor 2 | RUNX2 |
| MOL000098 | quercetin | Ras association domain-containing protein 1 | RASSF1 |
| MOL000098 | quercetin | Transcription factor E2F1 | E2F1 |
| MOL000098 | quercetin | Transcription factor E2F2 | E2F2 |
| MOL000098 | quercetin | Prostatic acid phosphatase | ACPP |
| MOL000098 | quercetin | Cathepsin D | CTSD |
| MOL000098 | quercetin | Insulin-like growth factor-binding protein 3 | IGFBP3 |
| MOL000098 | quercetin | Insulin-like growth factor II | IGF2 |
| MOL000098 | quercetin | CD40 ligand | CD40LG |
| MOL000098 | quercetin | Interferon regulatory factor 1 | IRF1 |
| MOL000098 | quercetin | Receptor tyrosine-protein kinase erbB-3 | ERBB3 |
| MOL000098 | quercetin | Serum paraoxonase/arylesterase 1 | PON1 |
| MOL000098 | quercetin | Type I iodothyronine deiodinase | DIO1 |
| MOL000098 | quercetin | Procollagen C-endopeptidase enhancer 1 | PCOLCE |
| MOL000098 | quercetin | Puromycin-sensitive aminopeptidase | NPEPPS |
| MOL000098 | quercetin | Hexokinase-2 | HK2 |
| MOL000098 | quercetin | Ras GTPase-activating protein 1 | RASA1 |
| MOL000098 | quercetin | Glutathione S-transferase Mu 1 | GSTM1 |
| MOL000098 | quercetin | Glutathione S-transferase Mu 2 | GSTM2 |
