# Supplementary material for: Luteolin Isolated from Polygonum cuspidatum Is a Potential Compound against Nasopharyngeal Carcinoma
Source: Biomed Res Int. 2022 Dec 23;2022:9740066. doi: 10.1155/2022/9740066 (PMC9803567; doi:10.1155/2022/9740066)
Supplement: Supplementary Materials — Table S1: Basic information of the bioactive compounds of P. cuspidatum. Table S2: The targets for the bioactive compounds of P. cuspidatum in the TCMSP database. Table S3: The standard names of targets for the bioactive compounds of P. cuspidatum. Table S4: Basic information of the disease related targets for NPC. Table S5: The common targets of disease targets for NPC and bioactive compounds from P. cuspidatum. Table S6: GO analysis of common targets of drug compounds and diseases through the DAVID website. Table S7: KEGG pathway analysis of common targets of drug compounds and diseases through the DAVID website. Figure S1: Effect of different bioactive compounds on the survival rate of CNE2 cells in NPC. [file 9740066.f1.zip › Table 4S Basic information of the disease related targets for NPC.docx]

| Gene Symbol | Description | Category | Gifts | GC Id | Relevance score | GeneCards Link |
| --- | --- | --- | --- | --- | --- | --- |
| TP53 | Tumor Protein P53 | Protein Coding | 54 | GC17M007661 | 150.59 | https://www.genecards.org/cgi-bin/carddisp.pl?gene=TP53 |
| PIK3CA | Phosphatidylinositol-4,5-Bisphosphate 3-Kinase Catalytic Subunit Alpha | Protein Coding | 52 | GC03P179148 | 83.71 | https://www.genecards.org/cgi-bin/carddisp.pl?gene=PIK3CA |
| CDKN2A | Cyclin Dependent Kinase Inhibitor 2A | Protein Coding | 51 | GC09M021967 | 73.84 | https://www.genecards.org/cgi-bin/carddisp.pl?gene=CDKN2A |
| EGFR | Epidermal Growth Factor Receptor | Protein Coding | 54 | GC07P055019 | 71.97 | https://www.genecards.org/cgi-bin/carddisp.pl?gene=EGFR |
| PTEN | Phosphatase And Tensin Homolog | Protein Coding | 52 | GC10P087863 | 70.54 | https://www.genecards.org/cgi-bin/carddisp.pl?gene=PTEN |
| HRAS | HRas Proto-Oncogene, GTPase | Protein Coding | 52 | GC11M000635 | 69.79 | https://www.genecards.org/cgi-bin/carddisp.pl?gene=HRAS |
| CDH1 | Cadherin 1 | Protein Coding | 50 | GC16P068737 | 69.14 | https://www.genecards.org/cgi-bin/carddisp.pl?gene=CDH1 |
| CTNNB1 | Catenin Beta 1 | Protein Coding | 53 | GC03P041236 | 68.14 | https://www.genecards.org/cgi-bin/carddisp.pl?gene=CTNNB1 |
| ERBB2 | Erb-B2 Receptor Tyrosine Kinase 2 | Protein Coding | 54 | GC17P039687 | 65.74 | https://www.genecards.org/cgi-bin/carddisp.pl?gene=ERBB2 |
| MET | MET Proto-Oncogene, Receptor Tyrosine Kinase | Protein Coding | 54 | GC07P116672 | 65.36 | https://www.genecards.org/cgi-bin/carddisp.pl?gene=MET |
| AKT1 | AKT Serine/Threonine Kinase 1 | Protein Coding | 54 | GC14M104769 | 61.63 | https://www.genecards.org/cgi-bin/carddisp.pl?gene=AKT1 |
| CCND1 | Cyclin D1 | Protein Coding | 52 | GC11P069641 | 60.29 | https://www.genecards.org/cgi-bin/carddisp.pl?gene=CCND1 |
| TERT | Telomerase Reverse Transcriptase | Protein Coding | 51 | GC05M001253 | 52.13 | https://www.genecards.org/cgi-bin/carddisp.pl?gene=TERT |
| MST1R | Macrophage Stimulating 1 Receptor | Protein Coding | 48 | GC03M049958 | 51.85 | https://www.genecards.org/cgi-bin/carddisp.pl?gene=MST1R |
| NRAS | NRAS Proto-Oncogene, GTPase | Protein Coding | 50 | GC01M114704 | 49.39 | https://www.genecards.org/cgi-bin/carddisp.pl?gene=NRAS |
| CDKN1A | Cyclin Dependent Kinase Inhibitor 1A | Protein Coding | 50 | GC06P047460 | 48.85 | https://www.genecards.org/cgi-bin/carddisp.pl?gene=CDKN1A |
| MTOR | Mechanistic Target Of Rapamycin Kinase | Protein Coding | 54 | GC01M011106 | 48.66 | https://www.genecards.org/cgi-bin/carddisp.pl?gene=MTOR |
| SMAD4 | SMAD Family Member 4 | Protein Coding | 50 | GC18P051028 | 48.57 | https://www.genecards.org/cgi-bin/carddisp.pl?gene=SMAD4 |
| TGFBR2 | Transforming Growth Factor Beta Receptor 2 | Protein Coding | 51 | GC03P030623 | 45.45 | https://www.genecards.org/cgi-bin/carddisp.pl?gene=TGFBR2 |
| EGF | Epidermal Growth Factor | Protein Coding | 51 | GC04P109912 | 45.3 | https://www.genecards.org/cgi-bin/carddisp.pl?gene=EGF |
| BAX | BCL2 Associated X, Apoptosis Regulator | Protein Coding | 48 | GC19P048954 | 45.21 | https://www.genecards.org/cgi-bin/carddisp.pl?gene=BAX |
| MIR21 | MicroRNA 21 | RNA Gene | 24 | GC17P059841 | 44.01 | https://www.genecards.org/cgi-bin/carddisp.pl?gene=MIR21 |
| MDM2 | MDM2 Proto-Oncogene | Protein Coding | 52 | GC12P068808 | 43.89 | https://www.genecards.org/cgi-bin/carddisp.pl?gene=MDM2 |
| H19 | H19 Imprinted Maternally Expressed Transcript | RNA Gene | 28 | GC11M001995 | 42.6 | https://www.genecards.org/cgi-bin/carddisp.pl?gene=H19 |
| KIT | KIT Proto-Oncogene, Receptor Tyrosine Kinase | Protein Coding | 53 | GC04P054657 | 42.34 | https://www.genecards.org/cgi-bin/carddisp.pl?gene=KIT |
| MIR31 | MicroRNA 31 | RNA Gene | 20 | GC09M021513 | 40.56 | https://www.genecards.org/cgi-bin/carddisp.pl?gene=MIR31 |
| ESR1 | Estrogen Receptor 1 | Protein Coding | 53 | GC06P151656 | 40.35 | https://www.genecards.org/cgi-bin/carddisp.pl?gene=ESR1 |
| MMP1 | Matrix Metallopeptidase 1 | Protein Coding | 51 | GC11M102810 | 38.98 | https://www.genecards.org/cgi-bin/carddisp.pl?gene=MMP1 |
| MIR143 | MicroRNA 143 | RNA Gene | 22 | GC05P149410 | 38.65 | https://www.genecards.org/cgi-bin/carddisp.pl?gene=MIR143 |
| MIR17 | MicroRNA 17 | RNA Gene | 21 | GC13P091350 | 38.6 | https://www.genecards.org/cgi-bin/carddisp.pl?gene=MIR17 |
| VEGFA | Vascular Endothelial Growth Factor A | Protein Coding | 48 | GC06P043770 | 38.4 | https://www.genecards.org/cgi-bin/carddisp.pl?gene=VEGFA |
| FGFR1 | Fibroblast Growth Factor Receptor 1 | Protein Coding | 55 | GC08M038400 | 38.03 | https://www.genecards.org/cgi-bin/carddisp.pl?gene=FGFR1 |
| MIR141 | MicroRNA 141 | RNA Gene | 21 | GC12P008231 | 37.93 | https://www.genecards.org/cgi-bin/carddisp.pl?gene=MIR141 |
| RAF1 | Raf-1 Proto-Oncogene, Serine/Threonine Kinase | Protein Coding | 54 | GC03M012583 | 37.88 | https://www.genecards.org/cgi-bin/carddisp.pl?gene=RAF1 |
| MIR125A | MicroRNA 125a | RNA Gene | 21 | GC19P051720 | 35.94 | https://www.genecards.org/cgi-bin/carddisp.pl?gene=MIR125A |
| MIR29C | MicroRNA 29c | RNA Gene | 18 | GC01M207802 | 35.41 | https://www.genecards.org/cgi-bin/carddisp.pl?gene=MIR29C |
| MYC | MYC Proto-Oncogene, BHLH Transcription Factor | Protein Coding | 51 | GC08P127735 | 35.22 | https://www.genecards.org/cgi-bin/carddisp.pl?gene=MYC |
| MIR205 | MicroRNA 205 | RNA Gene | 20 | GC01P209432 | 35.03 | https://www.genecards.org/cgi-bin/carddisp.pl?gene=MIR205 |
| MIR185 | MicroRNA 185 | RNA Gene | 21 | GC22P020034 | 34.83 | https://www.genecards.org/cgi-bin/carddisp.pl?gene=MIR185 |
| ERCC1 | ERCC Excision Repair 1, Endonuclease Non-Catalytic Subunit | Protein Coding | 45 | GC19M045409 | 34.77 | https://www.genecards.org/cgi-bin/carddisp.pl?gene=ERCC1 |
| TWIST1 | Twist Family BHLH Transcription Factor 1 | Protein Coding | 45 | GC07M019020 | 34.12 | https://www.genecards.org/cgi-bin/carddisp.pl?gene=TWIST1 |
| MIR30E | MicroRNA 30e | RNA Gene | 21 | GC01P040754 | 33.5 | https://www.genecards.org/cgi-bin/carddisp.pl?gene=MIR30E |
| MIR93 | MicroRNA 93 | RNA Gene | 20 | GC07M100282 | 33.11 | https://www.genecards.org/cgi-bin/carddisp.pl?gene=MIR93 |
| KRT5 | Keratin 5 | Protein Coding | 47 | GC12M052514 | 32.81 | https://www.genecards.org/cgi-bin/carddisp.pl?gene=KRT5 |
| MIR10B | MicroRNA 10b | RNA Gene | 21 | GC02P176150 | 32.62 | https://www.genecards.org/cgi-bin/carddisp.pl?gene=MIR10B |
| MMP9 | Matrix Metallopeptidase 9 | Protein Coding | 52 | GC20P046008 | 32.44 | https://www.genecards.org/cgi-bin/carddisp.pl?gene=MMP9 |
| MIR15A | MicroRNA 15a | RNA Gene | 16 | GC13M050049 | 32.19 | https://www.genecards.org/cgi-bin/carddisp.pl?gene=MIR15A |
| HIF1A | Hypoxia Inducible Factor 1 Subunit Alpha | Protein Coding | 47 | GC14P061695 | 31.93 | https://www.genecards.org/cgi-bin/carddisp.pl?gene=HIF1A |
| PTGS2 | Prostaglandin-Endoperoxide Synthase 2 | Protein Coding | 48 | GC01M186640 | 31.05 | https://www.genecards.org/cgi-bin/carddisp.pl?gene=PTGS2 |
| STAT3 | Signal Transducer And Activator Of Transcription 3 | Protein Coding | 52 | GC17M042313 | 30.86 | https://www.genecards.org/cgi-bin/carddisp.pl?gene=STAT3 |
| BCL2 | BCL2 Apoptosis Regulator | Protein Coding | 51 | GC18M063123 | 30.78 | https://www.genecards.org/cgi-bin/carddisp.pl?gene=BCL2 |
| NFKBIA | NFKB Inhibitor Alpha | Protein Coding | 50 | GC14M035401 | 30.56 | https://www.genecards.org/cgi-bin/carddisp.pl?gene=NFKBIA |
| MIR335 | MicroRNA 335 | RNA Gene | 18 | GC07P130496 | 30.3 | https://www.genecards.org/cgi-bin/carddisp.pl?gene=MIR335 |
| MIR34C | MicroRNA 34c | RNA Gene | 21 | GC11P111582 | 30.23 | https://www.genecards.org/cgi-bin/carddisp.pl?gene=MIR34C |
| IL1B | Interleukin 1 Beta | Protein Coding | 48 | GC02M112829 | 30.15 | https://www.genecards.org/cgi-bin/carddisp.pl?gene=IL1B |
| KRAS | KRAS Proto-Oncogene, GTPase | Protein Coding | 51 | GC12M025204 | 29.92 | https://www.genecards.org/cgi-bin/carddisp.pl?gene=KRAS |
| MMP2 | Matrix Metallopeptidase 2 | Protein Coding | 53 | GC16P055390 | 29.63 | https://www.genecards.org/cgi-bin/carddisp.pl?gene=MMP2 |
| KRT7 | Keratin 7 | Protein Coding | 41 | GC12P052232 | 29.53 | https://www.genecards.org/cgi-bin/carddisp.pl?gene=KRT7 |
| CD44 | CD44 Molecule (Indian Blood Group) | Protein Coding | 47 | GC11P035139 | 29.45 | https://www.genecards.org/cgi-bin/carddisp.pl?gene=CD44 |
| APC | APC Regulator Of WNT Signaling Pathway | Protein Coding | 48 | GC05P112707 | 29.45 | https://www.genecards.org/cgi-bin/carddisp.pl?gene=APC |
| MUC1 | Mucin 1, Cell Surface Associated | Protein Coding | 47 | GC01M155185 | 29.04 | https://www.genecards.org/cgi-bin/carddisp.pl?gene=MUC1 |
| BRAF | B-Raf Proto-Oncogene, Serine/Threonine Kinase | Protein Coding | 54 | GC07M140719 | 28.88 | https://www.genecards.org/cgi-bin/carddisp.pl?gene=BRAF |
| MIR100 | MicroRNA 100 | RNA Gene | 21 | GC11M122152 | 28.86 | https://www.genecards.org/cgi-bin/carddisp.pl?gene=MIR100 |
| CD274 | CD274 Molecule | Protein Coding | 44 | GC09P005450 | 28.64 | https://www.genecards.org/cgi-bin/carddisp.pl?gene=CD274 |
| IL6 | Interleukin 6 | Protein Coding | 50 | GC07P022765 | 28.11 | https://www.genecards.org/cgi-bin/carddisp.pl?gene=IL6 |
| MIR192 | MicroRNA 192 | RNA Gene | 21 | GC11M064891 | 27.92 | https://www.genecards.org/cgi-bin/carddisp.pl?gene=MIR192 |
| MIR324 | MicroRNA 324 | RNA Gene | 18 | GC17M007223 | 27.81 | https://www.genecards.org/cgi-bin/carddisp.pl?gene=MIR324 |
| MIR27A | MicroRNA 27a | RNA Gene | 22 | GC19M014010 | 27.54 | https://www.genecards.org/cgi-bin/carddisp.pl?gene=MIR27A |
| CXCL8 | C-X-C Motif Chemokine Ligand 8 | Protein Coding | 41 | GC04P073740 | 27.32 | https://www.genecards.org/cgi-bin/carddisp.pl?gene=CXCL8 |
| CASP3 | Caspase 3 | Protein Coding | 50 | GC04M184627 | 27.1 | https://www.genecards.org/cgi-bin/carddisp.pl?gene=CASP3 |
| SMARCB1 | SWI/SNF Related, Matrix Associated, Actin Dependent Regulator Of Chromatin, Subfamily B, Member 1 | Protein Coding | 45 | GC22P023786 | 26.46 | https://www.genecards.org/cgi-bin/carddisp.pl?gene=SMARCB1 |
| TNF | Tumor Necrosis Factor | Protein Coding | 51 | GC06P047305 | 26.44 | https://www.genecards.org/cgi-bin/carddisp.pl?gene=TNF |
| NME1 | NME/NM23 Nucleoside Diphosphate Kinase 1 | Protein Coding | 47 | GC17P051154 | 26.16 | https://www.genecards.org/cgi-bin/carddisp.pl?gene=NME1 |
| RASSF1 | Ras Association Domain Family Member 1 | Protein Coding | 44 | GC03M050329 | 26.15 | https://www.genecards.org/cgi-bin/carddisp.pl?gene=RASSF1 |
| NOTCH1 | Notch Receptor 1 | Protein Coding | 51 | GC09M136602 | 25.8 | https://www.genecards.org/cgi-bin/carddisp.pl?gene=NOTCH1 |
| MIR34B | MicroRNA 34b | RNA Gene | 21 | GC11P111546 | 25.74 | https://www.genecards.org/cgi-bin/carddisp.pl?gene=MIR34B |
| MIR342 | MicroRNA 342 | RNA Gene | 19 | GC14P100109 | 25.72 | https://www.genecards.org/cgi-bin/carddisp.pl?gene=MIR342 |
| MIR140 | MicroRNA 140 | RNA Gene | 22 | GC16P069934 | 25.62 | https://www.genecards.org/cgi-bin/carddisp.pl?gene=MIR140 |
| MIR193A | MicroRNA 193a | RNA Gene | 18 | GC17P031559 | 25.34 | https://www.genecards.org/cgi-bin/carddisp.pl?gene=MIR193A |
| STK11 | Serine/Threonine Kinase 11 | Protein Coding | 49 | GC19P001177 | 25.15 | https://www.genecards.org/cgi-bin/carddisp.pl?gene=STK11 |
| MIR224 | MicroRNA 224 | RNA Gene | 17 | GC0XM151958 | 25.12 | https://www.genecards.org/cgi-bin/carddisp.pl?gene=MIR224 |
| KRT19 | Keratin 19 | Protein Coding | 45 | GC17M041523 | 25.07 | https://www.genecards.org/cgi-bin/carddisp.pl?gene=KRT19 |
| VEGFC | Vascular Endothelial Growth Factor C | Protein Coding | 47 | GC04M176683 | 24.95 | https://www.genecards.org/cgi-bin/carddisp.pl?gene=VEGFC |
| FGFR2 | Fibroblast Growth Factor Receptor 2 | Protein Coding | 54 | GC10M121478 | 24.83 | https://www.genecards.org/cgi-bin/carddisp.pl?gene=FGFR2 |
| MLH1 | MutL Homolog 1 | Protein Coding | 48 | GC03P036993 | 24.76 | https://www.genecards.org/cgi-bin/carddisp.pl?gene=MLH1 |
| CDK4 | Cyclin Dependent Kinase 4 | Protein Coding | 54 | GC12M057743 | 24.76 | https://www.genecards.org/cgi-bin/carddisp.pl?gene=CDK4 |
| GSTM1 | Glutathione S-Transferase Mu 1 | Protein Coding | 41 | GC01P109687 | 24.48 | https://www.genecards.org/cgi-bin/carddisp.pl?gene=GSTM1 |
| JUN | Jun Proto-Oncogene, AP-1 Transcription Factor Subunit | Protein Coding | 49 | GC01M058780 | 24.47 | https://www.genecards.org/cgi-bin/carddisp.pl?gene=JUN |
| CA9 | Carbonic Anhydrase 9 | Protein Coding | 44 | GC09P035673 | 24.42 | https://www.genecards.org/cgi-bin/carddisp.pl?gene=CA9 |
| MIR148A | MicroRNA 148a | RNA Gene | 18 | GC07M025993 | 24.31 | https://www.genecards.org/cgi-bin/carddisp.pl?gene=MIR148A |
| XRCC1 | X-Ray Repair Cross Complementing 1 | Protein Coding | 43 | GC19M043543 | 24.31 | https://www.genecards.org/cgi-bin/carddisp.pl?gene=XRCC1 |
| MIR483 | MicroRNA 483 | RNA Gene | 18 | GC11M002188 | 24.29 | https://www.genecards.org/cgi-bin/carddisp.pl?gene=MIR483 |
| BCL2L1 | BCL2 Like 1 | Protein Coding | 47 | GC20M031664 | 24.15 | https://www.genecards.org/cgi-bin/carddisp.pl?gene=BCL2L1 |
| EZH2 | Enhancer Of Zeste 2 Polycomb Repressive Complex 2 Subunit | Protein Coding | 54 | GC07M148807 | 23.92 | https://www.genecards.org/cgi-bin/carddisp.pl?gene=EZH2 |
| CXCR4 | C-X-C Motif Chemokine Receptor 4 | Protein Coding | 52 | GC02M136114 | 23.86 | https://www.genecards.org/cgi-bin/carddisp.pl?gene=CXCR4 |
| PGR | Progesterone Receptor | Protein Coding | 50 | GC11M100943 | 23.78 | https://www.genecards.org/cgi-bin/carddisp.pl?gene=PGR |
| BRCA1 | BRCA1 DNA Repair Associated | Protein Coding | 50 | GC17M043044 | 23.78 | https://www.genecards.org/cgi-bin/carddisp.pl?gene=BRCA1 |
| NPCA2 | Nasopharyngeal Carcinoma, Susceptibility To, 2 | Genetic Locus | 2 | GC06U901697 | 23.71 | https://www.genecards.org/cgi-bin/carddisp.pl?gene=NPCA2 |
| VHL | Von Hippel-Lindau Tumor Suppressor | Protein Coding | 47 | GC03P010211 | 23.62 | https://www.genecards.org/cgi-bin/carddisp.pl?gene=VHL |
| IL10 | Interleukin 10 | Protein Coding | 47 | GC01M206767 | 23.39 | https://www.genecards.org/cgi-bin/carddisp.pl?gene=IL10 |
| CDKN1B | Cyclin Dependent Kinase Inhibitor 1B | Protein Coding | 48 | GC12P012716 | 23.18 | https://www.genecards.org/cgi-bin/carddisp.pl?gene=CDKN1B |
| FGFR3 | Fibroblast Growth Factor Receptor 3 | Protein Coding | 55 | GC04P001795 | 22.94 | https://www.genecards.org/cgi-bin/carddisp.pl?gene=FGFR3 |
| MIR486-1 | MicroRNA 486-1 | RNA Gene | 16 | GC08M041660 | 22.87 | https://www.genecards.org/cgi-bin/carddisp.pl?gene=MIR486-1 |
| CASP9 | Caspase 9 | Protein Coding | 48 | GC01M015491 | 22.43 | https://www.genecards.org/cgi-bin/carddisp.pl?gene=CASP9 |
| KRT20 | Keratin 20 | Protein Coding | 41 | GC17M040875 | 22.29 | https://www.genecards.org/cgi-bin/carddisp.pl?gene=KRT20 |
| VIM | Vimentin | Protein Coding | 50 | GC10P017227 | 22.14 | https://www.genecards.org/cgi-bin/carddisp.pl?gene=VIM |
| MALAT1 | Metastasis Associated Lung Adenocarcinoma Transcript 1 | RNA Gene | 24 | GC11P065806 | 22.09 | https://www.genecards.org/cgi-bin/carddisp.pl?gene=MALAT1 |
| FHIT | Fragile Histidine Triad Diadenosine Triphosphatase | Protein Coding | 44 | GC03M059747 | 21.82 | https://www.genecards.org/cgi-bin/carddisp.pl?gene=FHIT |
| HOTAIR | HOX Transcript Antisense RNA | RNA Gene | 25 | GC12M053962 | 21.81 | https://www.genecards.org/cgi-bin/carddisp.pl?gene=HOTAIR |
| FGF2 | Fibroblast Growth Factor 2 | Protein Coding | 47 | GC04P122826 | 21.77 | https://www.genecards.org/cgi-bin/carddisp.pl?gene=FGF2 |
| CCNB1 | Cyclin B1 | Protein Coding | 47 | GC05P069167 | 21.49 | https://www.genecards.org/cgi-bin/carddisp.pl?gene=CCNB1 |
| NKX2-1 | NK2 Homeobox 1 | Protein Coding | 46 | GC14M036516 | 21.46 | https://www.genecards.org/cgi-bin/carddisp.pl?gene=NKX2-1 |
| PARP1 | Poly(ADP-Ribose) Polymerase 1 | Protein Coding | 49 | GC01M226360 | 21.42 | https://www.genecards.org/cgi-bin/carddisp.pl?gene=PARP1 |
| CDH2 | Cadherin 2 | Protein Coding | 50 | GC18M027950 | 21.42 | https://www.genecards.org/cgi-bin/carddisp.pl?gene=CDH2 |
| DNMT1 | DNA Methyltransferase 1 | Protein Coding | 50 | GC19M010133 | 21.39 | https://www.genecards.org/cgi-bin/carddisp.pl?gene=DNMT1 |
| RB1 | RB Transcriptional Corepressor 1 | Protein Coding | 49 | GC13P048303 | 21.24 | https://www.genecards.org/cgi-bin/carddisp.pl?gene=RB1 |
| MAPK8 | Mitogen-Activated Protein Kinase 8 | Protein Coding | 50 | GC10P048306 | 21.23 | https://www.genecards.org/cgi-bin/carddisp.pl?gene=MAPK8 |
| SOX2 | SRY-Box Transcription Factor 2 | Protein Coding | 47 | GC03P181711 | 21.16 | https://www.genecards.org/cgi-bin/carddisp.pl?gene=SOX2 |
| PCNA | Proliferating Cell Nuclear Antigen | Protein Coding | 51 | GC20M005114 | 21.01 | https://www.genecards.org/cgi-bin/carddisp.pl?gene=PCNA |
| MIR15B | MicroRNA 15b | RNA Gene | 18 | GC03P160404 | 21 | https://www.genecards.org/cgi-bin/carddisp.pl?gene=MIR15B |
| CXCL12 | C-X-C Motif Chemokine Ligand 12 | Protein Coding | 45 | GC10M044294 | 20.98 | https://www.genecards.org/cgi-bin/carddisp.pl?gene=CXCL12 |
| MIR30B | MicroRNA 30b | RNA Gene | 20 | GC08M134800 | 20.89 | https://www.genecards.org/cgi-bin/carddisp.pl?gene=MIR30B |
| CDKN2B-AS1 | CDKN2B Antisense RNA 1 | RNA Gene | 21 | GC09P021994 | 20.72 | https://www.genecards.org/cgi-bin/carddisp.pl?gene=CDKN2B-AS1 |
| EPCAM | Epithelial Cell Adhesion Molecule | Protein Coding | 47 | GC02P047345 | 20.7 | https://www.genecards.org/cgi-bin/carddisp.pl?gene=EPCAM |
| FAS | Fas Cell Surface Death Receptor | Protein Coding | 50 | GC10P088969 | 20.61 | https://www.genecards.org/cgi-bin/carddisp.pl?gene=FAS |
| MIR30A | MicroRNA 30a | RNA Gene | 20 | GC06M071403 | 20.58 | https://www.genecards.org/cgi-bin/carddisp.pl?gene=MIR30A |
| KRT18 | Keratin 18 | Protein Coding | 48 | GC12P052948 | 20.38 | https://www.genecards.org/cgi-bin/carddisp.pl?gene=KRT18 |
| BMI1 | BMI1 Proto-Oncogene, Polycomb Ring Finger | Protein Coding | 43 | GC10P022326 | 20.3 | https://www.genecards.org/cgi-bin/carddisp.pl?gene=BMI1 |
| ABCC1 | ATP Binding Cassette Subfamily C Member 1 | Protein Coding | 47 | GC16P015949 | 20.15 | https://www.genecards.org/cgi-bin/carddisp.pl?gene=ABCC1 |
| SNAI1 | Snail Family Transcriptional Repressor 1 | Protein Coding | 44 | GC20P049982 | 19.95 | https://www.genecards.org/cgi-bin/carddisp.pl?gene=SNAI1 |
| CYCS | Cytochrome C, Somatic | Protein Coding | 48 | GC07M025118 | 19.86 | https://www.genecards.org/cgi-bin/carddisp.pl?gene=CYCS |
| DNMT3B | DNA Methyltransferase 3 Beta | Protein Coding | 50 | GC20P032762 | 19.83 | https://www.genecards.org/cgi-bin/carddisp.pl?gene=DNMT3B |
| PTCH1 | Patched 1 | Protein Coding | 50 | GC09M095442 | 19.74 | https://www.genecards.org/cgi-bin/carddisp.pl?gene=PTCH1 |
| HSPB1 | Heat Shock Protein Family B (Small) Member 1 | Protein Coding | 51 | GC07P076302 | 19.68 | https://www.genecards.org/cgi-bin/carddisp.pl?gene=HSPB1 |
| KRT8 | Keratin 8 | Protein Coding | 47 | GC12M052897 | 19.65 | https://www.genecards.org/cgi-bin/carddisp.pl?gene=KRT8 |
| SNAI2 | Snail Family Transcriptional Repressor 2 | Protein Coding | 44 | GC08M048854 | 19.65 | https://www.genecards.org/cgi-bin/carddisp.pl?gene=SNAI2 |
| MIR9-1 | MicroRNA 9-1 | RNA Gene | 20 | GC01M156420 | 19.47 | https://www.genecards.org/cgi-bin/carddisp.pl?gene=MIR9-1 |
| PVT1 | Pvt1 Oncogene | RNA Gene | 25 | GC08P127804 | 19.36 | https://www.genecards.org/cgi-bin/carddisp.pl?gene=PVT1 |
| ANXA5 | Annexin A5 | Protein Coding | 46 | GC04M121667 | 19.35 | https://www.genecards.org/cgi-bin/carddisp.pl?gene=ANXA5 |
| MEG3 | Maternally Expressed 3 | RNA Gene | 29 | GC14P104771 | 19.34 | https://www.genecards.org/cgi-bin/carddisp.pl?gene=MEG3 |
| MMP3 | Matrix Metallopeptidase 3 | Protein Coding | 51 | GC11M102835 | 19.24 | https://www.genecards.org/cgi-bin/carddisp.pl?gene=MMP3 |
| MIR139 | MicroRNA 139 | RNA Gene | 19 | GC11M072615 | 19.23 | https://www.genecards.org/cgi-bin/carddisp.pl?gene=MIR139 |
| TMEM8B | Transmembrane Protein 8B | Protein Coding | 33 | GC09P035785 | 19.21 | https://www.genecards.org/cgi-bin/carddisp.pl?gene=TMEM8B |
| MIR144 | MicroRNA 144 | RNA Gene | 16 | GC17M029965 | 19.13 | https://www.genecards.org/cgi-bin/carddisp.pl?gene=MIR144 |
| CASP8 | Caspase 8 | Protein Coding | 52 | GC02P201233 | 19.09 | https://www.genecards.org/cgi-bin/carddisp.pl?gene=CASP8 |
| DAPK1 | Death Associated Protein Kinase 1 | Protein Coding | 48 | GC09P087497 | 18.77 | https://www.genecards.org/cgi-bin/carddisp.pl?gene=DAPK1 |
| ATM | ATM Serine/Threonine Kinase | Protein Coding | 54 | GC11P108222 | 18.7 | https://www.genecards.org/cgi-bin/carddisp.pl?gene=ATM |
| BPIFA1 | BPI Fold Containing Family A Member 1 | Protein Coding | 36 | GC20P033235 | 18.7 | https://www.genecards.org/cgi-bin/carddisp.pl?gene=BPIFA1 |
| OGG1 | 8-Oxoguanine DNA Glycosylase | Protein Coding | 47 | GC03P009751 | 18.57 | https://www.genecards.org/cgi-bin/carddisp.pl?gene=OGG1 |
| ZEB1 | Zinc Finger E-Box Binding Homeobox 1 | Protein Coding | 48 | GC10P031318 | 18.55 | https://www.genecards.org/cgi-bin/carddisp.pl?gene=ZEB1 |
| EZR | Ezrin | Protein Coding | 45 | GC06M158765 | 18.44 | https://www.genecards.org/cgi-bin/carddisp.pl?gene=EZR |
| GAS5 | Growth Arrest Specific 5 | RNA Gene | 23 | GC01M173947 | 18.43 | https://www.genecards.org/cgi-bin/carddisp.pl?gene=GAS5 |
| MIR142 | MicroRNA 142 | RNA Gene | 20 | GC17M058331 | 18.38 | https://www.genecards.org/cgi-bin/carddisp.pl?gene=MIR142 |
| CHEK2 | Checkpoint Kinase 2 | Protein Coding | 53 | GC22M028687 | 18.3 | https://www.genecards.org/cgi-bin/carddisp.pl?gene=CHEK2 |
| ENO2 | Enolase 2 | Protein Coding | 47 | GC12P006913 | 18.29 | https://www.genecards.org/cgi-bin/carddisp.pl?gene=ENO2 |
| TLR2 | Toll Like Receptor 2 | Protein Coding | 51 | GC04P153684 | 18.19 | https://www.genecards.org/cgi-bin/carddisp.pl?gene=TLR2 |
| CDKN2B | Cyclin Dependent Kinase Inhibitor 2B | Protein Coding | 47 | GC09M022002 | 18.07 | https://www.genecards.org/cgi-bin/carddisp.pl?gene=CDKN2B |
| CYP2E1 | Cytochrome P450 Family 2 Subfamily E Member 1 | Protein Coding | 45 | GC10P133520 | 17.94 | https://www.genecards.org/cgi-bin/carddisp.pl?gene=CYP2E1 |
| HLA-A | Major Histocompatibility Complex, Class I, A | Protein Coding | 46 | GC06P047265 | 17.91 | https://www.genecards.org/cgi-bin/carddisp.pl?gene=HLA-A |
| MIR423 | MicroRNA 423 | RNA Gene | 18 | GC17P030117 | 17.86 | https://www.genecards.org/cgi-bin/carddisp.pl?gene=MIR423 |
| NEAT1 | Nuclear Paraspeckle Assembly Transcript 1 | RNA Gene | 23 | GC11P065794 | 17.68 | https://www.genecards.org/cgi-bin/carddisp.pl?gene=NEAT1 |
| LINC00312 | Long Intergenic Non-Protein Coding RNA 312 | RNA Gene | 20 | GC03P008571 | 17.68 | https://www.genecards.org/cgi-bin/carddisp.pl?gene=LINC00312 |
| XIST | X Inactive Specific Transcript | RNA Gene | 24 | GC0XM073820 | 17.66 | https://www.genecards.org/cgi-bin/carddisp.pl?gene=XIST |
| MIR296 | MicroRNA 296 | RNA Gene | 17 | GC20M058817 | 17.64 | https://www.genecards.org/cgi-bin/carddisp.pl?gene=MIR296 |
| CCAT1 | Colon Cancer Associated Transcript 1 | RNA Gene | 14 | GC08M127207 | 17.5 | https://www.genecards.org/cgi-bin/carddisp.pl?gene=CCAT1 |
| HULC | Hepatocellular Carcinoma Up-Regulated Long Non-Coding RNA | RNA Gene | 21 | GC06P008438 | 17.45 | https://www.genecards.org/cgi-bin/carddisp.pl?gene=HULC |
| FASLG | Fas Ligand | Protein Coding | 47 | GC01P172628 | 17.41 | https://www.genecards.org/cgi-bin/carddisp.pl?gene=FASLG |
| BIRC5 | Baculoviral IAP Repeat Containing 5 | Protein Coding | 47 | GC17P078214 | 17.33 | https://www.genecards.org/cgi-bin/carddisp.pl?gene=BIRC5 |
| LINC-ROR | Long Intergenic Non-Protein Coding RNA, Regulator Of Reprogramming | RNA Gene | 16 | GC18M057054 | 17.25 | https://www.genecards.org/cgi-bin/carddisp.pl?gene=LINC-ROR |
| ETS1 | ETS Proto-Oncogene 1, Transcription Factor | Protein Coding | 49 | GC11M128458 | 17.12 | https://www.genecards.org/cgi-bin/carddisp.pl?gene=ETS1 |
| MIR338 | MicroRNA 338 | RNA Gene | 17 | GC17M081126 | 17.12 | https://www.genecards.org/cgi-bin/carddisp.pl?gene=MIR338 |
| PDCD1 | Programmed Cell Death 1 | Protein Coding | 48 | GC02M241849 | 17.11 | https://www.genecards.org/cgi-bin/carddisp.pl?gene=PDCD1 |
| ALB | Albumin | Protein Coding | 50 | GC04P073397 | 16.84 | https://www.genecards.org/cgi-bin/carddisp.pl?gene=ALB |
| MIR29B1 | MicroRNA 29b-1 | RNA Gene | 21 | GC07M130877 | 16.67 | https://www.genecards.org/cgi-bin/carddisp.pl?gene=MIR29B1 |
| PSMB9 | Proteasome 20S Subunit Beta 9 | Protein Coding | 46 | GC06P047342 | 16.54 | https://www.genecards.org/cgi-bin/carddisp.pl?gene=PSMB9 |
| LRRC56 | Leucine Rich Repeat Containing 56 | Protein Coding | 36 | GC11P000518 | 16.54 | https://www.genecards.org/cgi-bin/carddisp.pl?gene=LRRC56 |
| SETD2 | SET Domain Containing 2, Histone Lysine Methyltransferase | Protein Coding | 47 | GC03M047033 | 16.4 | https://www.genecards.org/cgi-bin/carddisp.pl?gene=SETD2 |
| NPC1 | NPC Intracellular Cholesterol Transporter 1 | Protein Coding | 47 | GC18M023506 | 16.33 | https://www.genecards.org/cgi-bin/carddisp.pl?gene=NPC1 |
| MUC5AC | Mucin 5AC, Oligomeric Mucus/Gel-Forming | Protein Coding | 39 | GC11P001151 | 16.32 | https://www.genecards.org/cgi-bin/carddisp.pl?gene=MUC5AC |
| HSPA4 | Heat Shock Protein Family A (Hsp70) Member 4 | Protein Coding | 41 | GC05P133051 | 16.22 | https://www.genecards.org/cgi-bin/carddisp.pl?gene=HSPA4 |
| CDH13 | Cadherin 13 | Protein Coding | 42 | GC16P082626 | 16.2 | https://www.genecards.org/cgi-bin/carddisp.pl?gene=CDH13 |
| NTRK1 | Neurotrophic Receptor Tyrosine Kinase 1 | Protein Coding | 48 | GC01P156786 | 16.2 | https://www.genecards.org/cgi-bin/carddisp.pl?gene=NTRK1 |
| MIR193B | MicroRNA 193b | RNA Gene | 20 | GC16P014307 | 16.18 | https://www.genecards.org/cgi-bin/carddisp.pl?gene=MIR193B |
| CCR6 | C-C Motif Chemokine Receptor 6 | Protein Coding | 44 | GC06P167111 | 16.17 | https://www.genecards.org/cgi-bin/carddisp.pl?gene=CCR6 |
| TP63 | Tumor Protein P63 | Protein Coding | 48 | GC03P189598 | 16.14 | https://www.genecards.org/cgi-bin/carddisp.pl?gene=TP63 |
| IFNG | Interferon Gamma | Protein Coding | 48 | GC12M068064 | 16.14 | https://www.genecards.org/cgi-bin/carddisp.pl?gene=IFNG |
| AFAP1-AS1 | AFAP1 Antisense RNA 1 | RNA Gene | 17 | GC04P007756 | 16.07 | https://www.genecards.org/cgi-bin/carddisp.pl?gene=AFAP1-AS1 |
| ACTC1 | Actin Alpha Cardiac Muscle 1 | Protein Coding | 42 | GC15M034788 | 16.04 | https://www.genecards.org/cgi-bin/carddisp.pl?gene=ACTC1 |
| MKI67 | Marker Of Proliferation Ki-67 | Protein Coding | 44 | GC10M128096 | 16.03 | https://www.genecards.org/cgi-bin/carddisp.pl?gene=MKI67 |
| KRT13 | Keratin 13 | Protein Coding | 43 | GC17M041500 | 16.02 | https://www.genecards.org/cgi-bin/carddisp.pl?gene=KRT13 |
| MIR199A1 | MicroRNA 199a-1 | RNA Gene | 18 | GC19M010792 | 15.86 | https://www.genecards.org/cgi-bin/carddisp.pl?gene=MIR199A1 |
| IGF2 | Insulin Like Growth Factor 2 | Protein Coding | 48 | GC11M002130 | 15.81 | https://www.genecards.org/cgi-bin/carddisp.pl?gene=IGF2 |
| MIR24-1 | MicroRNA 24-1 | RNA Gene | 18 | GC09P095086 | 15.81 | https://www.genecards.org/cgi-bin/carddisp.pl?gene=MIR24-1 |
| HLA-B | Major Histocompatibility Complex, Class I, B | Protein Coding | 45 | GC06M031315 | 15.76 | https://www.genecards.org/cgi-bin/carddisp.pl?gene=HLA-B |
| ENG | Endoglin | Protein Coding | 46 | GC09M127815 | 15.73 | https://www.genecards.org/cgi-bin/carddisp.pl?gene=ENG |
| TGFB1 | Transforming Growth Factor Beta 1 | Protein Coding | 52 | GC19M041301 | 15.7 | https://www.genecards.org/cgi-bin/carddisp.pl?gene=TGFB1 |
| CD80 | CD80 Molecule | Protein Coding | 41 | GC03M119524 | 15.68 | https://www.genecards.org/cgi-bin/carddisp.pl?gene=CD80 |
| ABCC2 | ATP Binding Cassette Subfamily C Member 2 | Protein Coding | 47 | GC10P099782 | 15.67 | https://www.genecards.org/cgi-bin/carddisp.pl?gene=ABCC2 |
| CSF2 | Colony Stimulating Factor 2 | Protein Coding | 44 | GC05P132073 | 15.65 | https://www.genecards.org/cgi-bin/carddisp.pl?gene=CSF2 |
| MIR145 | MicroRNA 145 | RNA Gene | 21 | GC05P149430 | 15.54 | https://www.genecards.org/cgi-bin/carddisp.pl?gene=MIR145 |
| SERPINA3 | Serpin Family A Member 3 | Protein Coding | 43 | GC14P094612 | 15.41 | https://www.genecards.org/cgi-bin/carddisp.pl?gene=SERPINA3 |
| LGALS3 | Galectin 3 | Protein Coding | 44 | GC14P055124 | 15.29 | https://www.genecards.org/cgi-bin/carddisp.pl?gene=LGALS3 |
| MIR212 | MicroRNA 212 | RNA Gene | 19 | GC17M002050 | 15.26 | https://www.genecards.org/cgi-bin/carddisp.pl?gene=MIR212 |
| CYP19A1 | Cytochrome P450 Family 19 Subfamily A Member 1 | Protein Coding | 48 | GC15M051208 | 15.22 | https://www.genecards.org/cgi-bin/carddisp.pl?gene=CYP19A1 |
| TKT | Transketolase | Protein Coding | 46 | GC03M053224 | 15.12 | https://www.genecards.org/cgi-bin/carddisp.pl?gene=TKT |
| THY1 | Thy-1 Cell Surface Antigen | Protein Coding | 44 | GC11M119417 | 15.07 | https://www.genecards.org/cgi-bin/carddisp.pl?gene=THY1 |
| BMP6 | Bone Morphogenetic Protein 6 | Protein Coding | 43 | GC06P007726 | 14.91 | https://www.genecards.org/cgi-bin/carddisp.pl?gene=BMP6 |
| UBAP1 | Ubiquitin Associated Protein 1 | Protein Coding | 38 | GC09P034179 | 14.85 | https://www.genecards.org/cgi-bin/carddisp.pl?gene=UBAP1 |
| IL4 | Interleukin 4 | Protein Coding | 46 | GC05P132673 | 14.85 | https://www.genecards.org/cgi-bin/carddisp.pl?gene=IL4 |
| PDGFRA | Platelet Derived Growth Factor Receptor Alpha | Protein Coding | 55 | GC04P054229 | 14.82 | https://www.genecards.org/cgi-bin/carddisp.pl?gene=PDGFRA |
| PECAM1 | Platelet And Endothelial Cell Adhesion Molecule 1 | Protein Coding | 40 | GC17M064319 | 14.77 | https://www.genecards.org/cgi-bin/carddisp.pl?gene=PECAM1 |
| SRC | SRC Proto-Oncogene, Non-Receptor Tyrosine Kinase | Protein Coding | 51 | GC20P037344 | 14.74 | https://www.genecards.org/cgi-bin/carddisp.pl?gene=SRC |
| KDR | Kinase Insert Domain Receptor | Protein Coding | 53 | GC04M055078 | 14.7 | https://www.genecards.org/cgi-bin/carddisp.pl?gene=KDR |
| PPARG | Peroxisome Proliferator Activated Receptor Gamma | Protein Coding | 52 | GC03P012287 | 14.62 | https://www.genecards.org/cgi-bin/carddisp.pl?gene=PPARG |
| CRP | C-Reactive Protein | Protein Coding | 46 | GC01M159716 | 14.62 | https://www.genecards.org/cgi-bin/carddisp.pl?gene=CRP |
| ANKRD11 | Ankyrin Repeat Domain 11 | Protein Coding | 38 | GC16M089267 | 14.61 | https://www.genecards.org/cgi-bin/carddisp.pl?gene=ANKRD11 |
| IL17A | Interleukin 17A | Protein Coding | 42 | GC06P052186 | 14.5 | https://www.genecards.org/cgi-bin/carddisp.pl?gene=IL17A |
| PMS2 | PMS1 Homolog 2, Mismatch Repair System Component | Protein Coding | 48 | GC07M005973 | 14.48 | https://www.genecards.org/cgi-bin/carddisp.pl?gene=PMS2 |
| ICAM1 | Intercellular Adhesion Molecule 1 | Protein Coding | 50 | GC19P010270 | 14.48 | https://www.genecards.org/cgi-bin/carddisp.pl?gene=ICAM1 |
| STAT1 | Signal Transducer And Activator Of Transcription 1 | Protein Coding | 53 | GC02M190908 | 14.48 | https://www.genecards.org/cgi-bin/carddisp.pl?gene=STAT1 |
| CASC9 | Cancer Susceptibility 9 | RNA Gene | 14 | GC08M075132 | 14.47 | https://www.genecards.org/cgi-bin/carddisp.pl?gene=CASC9 |
| MIR26B | MicroRNA 26b | RNA Gene | 21 | GC02P218402 | 14.44 | https://www.genecards.org/cgi-bin/carddisp.pl?gene=MIR26B |
| S100A1 | S100 Calcium Binding Protein A1 | Protein Coding | 41 | GC01P153627 | 14.38 | https://www.genecards.org/cgi-bin/carddisp.pl?gene=S100A1 |
| MIR129-1 | MicroRNA 129-1 | RNA Gene | 17 | GC07P128207 | 14.37 | https://www.genecards.org/cgi-bin/carddisp.pl?gene=MIR129-1 |
| ERCC2 | ERCC Excision Repair 2, TFIIH Core Complex Helicase Subunit | Protein Coding | 47 | GC19M045349 | 14.33 | https://www.genecards.org/cgi-bin/carddisp.pl?gene=ERCC2 |
| MIR452 | MicroRNA 452 | RNA Gene | 15 | GC0XM151959 | 14.32 | https://www.genecards.org/cgi-bin/carddisp.pl?gene=MIR452 |
| NPTN-IT1 | NPTN Intronic Transcript 1 | RNA Gene | 12 | GC15M073566 | 14.29 | https://www.genecards.org/cgi-bin/carddisp.pl?gene=NPTN-IT1 |
| TNFRSF10B | TNF Receptor Superfamily Member 10b | Protein Coding | 50 | GC08M023020 | 14.28 | https://www.genecards.org/cgi-bin/carddisp.pl?gene=TNFRSF10B |
| CCR7 | C-C Motif Chemokine Receptor 7 | Protein Coding | 45 | GC17M040556 | 14.28 | https://www.genecards.org/cgi-bin/carddisp.pl?gene=CCR7 |
| TP73 | Tumor Protein P73 | Protein Coding | 45 | GC01P003652 | 14.23 | https://www.genecards.org/cgi-bin/carddisp.pl?gene=TP73 |
| TG | Thyroglobulin | Protein Coding | 42 | GC08P132866 | 14.22 | https://www.genecards.org/cgi-bin/carddisp.pl?gene=TG |
| GSTP1 | Glutathione S-Transferase Pi 1 | Protein Coding | 50 | GC11P067583 | 14.11 | https://www.genecards.org/cgi-bin/carddisp.pl?gene=GSTP1 |
| MIR34A | MicroRNA 34a | RNA Gene | 22 | GC01M009151 | 14.07 | https://www.genecards.org/cgi-bin/carddisp.pl?gene=MIR34A |
| TGFB3 | Transforming Growth Factor Beta 3 | Protein Coding | 47 | GC14M075958 | 14.05 | https://www.genecards.org/cgi-bin/carddisp.pl?gene=TGFB3 |
| H2AC18 | H2A Clustered Histone 18 | Protein Coding | 26 | GC01M149961 | 14 | https://www.genecards.org/cgi-bin/carddisp.pl?gene=H2AC18 |
| EP300 | E1A Binding Protein P300 | Protein Coding | 50 | GC22P041091 | 14 | https://www.genecards.org/cgi-bin/carddisp.pl?gene=EP300 |
| SDHB | Succinate Dehydrogenase Complex Iron Sulfur Subunit B | Protein Coding | 47 | GC01M017020 | 13.97 | https://www.genecards.org/cgi-bin/carddisp.pl?gene=SDHB |
| TLR4 | Toll Like Receptor 4 | Protein Coding | 51 | GC09P117704 | 13.96 | https://www.genecards.org/cgi-bin/carddisp.pl?gene=TLR4 |
| CDKN3 | Cyclin Dependent Kinase Inhibitor 3 | Protein Coding | 42 | GC14P054398 | 13.96 | https://www.genecards.org/cgi-bin/carddisp.pl?gene=CDKN3 |
| MIR155 | MicroRNA 155 | RNA Gene | 18 | GC21P025573 | 13.89 | https://www.genecards.org/cgi-bin/carddisp.pl?gene=MIR155 |
| MAPK1 | Mitogen-Activated Protein Kinase 1 | Protein Coding | 51 | GC22M021754 | 13.83 | https://www.genecards.org/cgi-bin/carddisp.pl?gene=MAPK1 |
| MIR363 | MicroRNA 363 | RNA Gene | 15 | GC0XM134205 | 13.81 | https://www.genecards.org/cgi-bin/carddisp.pl?gene=MIR363 |
| MAD2L1 | Mitotic Arrest Deficient 2 Like 1 | Protein Coding | 45 | GC04M120055 | 13.79 | https://www.genecards.org/cgi-bin/carddisp.pl?gene=MAD2L1 |
| PIGR | Polymeric Immunoglobulin Receptor | Protein Coding | 41 | GC01M206928 | 13.78 | https://www.genecards.org/cgi-bin/carddisp.pl?gene=PIGR |
| NKILA | NF-KappaB Interacting LncRNA | RNA Gene | 11 | GC20P057711 | 13.78 | https://www.genecards.org/cgi-bin/carddisp.pl?gene=NKILA |
| MIR214 | MicroRNA 214 | RNA Gene | 20 | GC01M172234 | 13.7 | https://www.genecards.org/cgi-bin/carddisp.pl?gene=MIR214 |
| MIR151A | MicroRNA 151a | RNA Gene | 17 | GC08M140733 | 13.69 | https://www.genecards.org/cgi-bin/carddisp.pl?gene=MIR151A |
| MIR200A | MicroRNA 200a | RNA Gene | 21 | GC01P001463 | 13.69 | https://www.genecards.org/cgi-bin/carddisp.pl?gene=MIR200A |
| HNF1A-AS1 | HNF1A Antisense RNA 1 | RNA Gene | 18 | GC12M121052 | 13.68 | https://www.genecards.org/cgi-bin/carddisp.pl?gene=HNF1A-AS1 |
| MPP1 | Membrane Palmitoylated Protein 1 | Protein Coding | 40 | GC0XM154779 | 13.68 | https://www.genecards.org/cgi-bin/carddisp.pl?gene=MPP1 |
| CEACAM3 | CEA Cell Adhesion Molecule 3 | Protein Coding | 40 | GC19P041796 | 13.63 | https://www.genecards.org/cgi-bin/carddisp.pl?gene=CEACAM3 |
| POSTN | Periostin | Protein Coding | 43 | GC13M037562 | 13.61 | https://www.genecards.org/cgi-bin/carddisp.pl?gene=POSTN |
| IL1RN | Interleukin 1 Receptor Antagonist | Protein Coding | 48 | GC02P115307 | 13.54 | https://www.genecards.org/cgi-bin/carddisp.pl?gene=IL1RN |
| CADM1 | Cell Adhesion Molecule 1 | Protein Coding | 43 | GC11M115169 | 13.53 | https://www.genecards.org/cgi-bin/carddisp.pl?gene=CADM1 |
| TLR3 | Toll Like Receptor 3 | Protein Coding | 52 | GC04P186059 | 13.46 | https://www.genecards.org/cgi-bin/carddisp.pl?gene=TLR3 |
| PCAT7 | Prostate Cancer Associated Transcript 7 | RNA Gene | 11 | GC09P094555 | 13.45 | https://www.genecards.org/cgi-bin/carddisp.pl?gene=PCAT7 |
| FLT4 | Fms Related Receptor Tyrosine Kinase 4 | Protein Coding | 52 | GC05M180607 | 13.41 | https://www.genecards.org/cgi-bin/carddisp.pl?gene=FLT4 |
| MIR744 | MicroRNA 744 | RNA Gene | 15 | GC17P012081 | 13.39 | https://www.genecards.org/cgi-bin/carddisp.pl?gene=MIR744 |
| MIR146A | MicroRNA 146a | RNA Gene | 22 | GC05P160485 | 13.35 | https://www.genecards.org/cgi-bin/carddisp.pl?gene=MIR146A |
| IFNA2 | Interferon Alpha 2 | Protein Coding | 41 | GC09M021384 | 13.3 | https://www.genecards.org/cgi-bin/carddisp.pl?gene=IFNA2 |
| BRD7 | Bromodomain Containing 7 | Protein Coding | 41 | GC16M050313 | 13.3 | https://www.genecards.org/cgi-bin/carddisp.pl?gene=BRD7 |
| ZMYND10 | Zinc Finger MYND-Type Containing 10 | Protein Coding | 39 | GC03M050389 | 13.22 | https://www.genecards.org/cgi-bin/carddisp.pl?gene=ZMYND10 |
| CSF3 | Colony Stimulating Factor 3 | Protein Coding | 40 | GC17P040015 | 13.2 | https://www.genecards.org/cgi-bin/carddisp.pl?gene=CSF3 |
| MIR200C | MicroRNA 200c | RNA Gene | 21 | GC12P008229 | 13.14 | https://www.genecards.org/cgi-bin/carddisp.pl?gene=MIR200C |
| PDGFRB | Platelet Derived Growth Factor Receptor Beta | Protein Coding | 55 | GC05M150113 | 13.13 | https://www.genecards.org/cgi-bin/carddisp.pl?gene=PDGFRB |
| NPCA1 | Nasopharyngeal Carcinoma 1 | Genetic Locus | 2 | GC04U901553 | 13.13 | https://www.genecards.org/cgi-bin/carddisp.pl?gene=NPCA1 |
| CEACAM5 | CEA Cell Adhesion Molecule 5 | Protein Coding | 41 | GC19P041709 | 13.12 | https://www.genecards.org/cgi-bin/carddisp.pl?gene=CEACAM5 |
| FN1 | Fibronectin 1 | Protein Coding | 50 | GC02M215360 | 13.05 | https://www.genecards.org/cgi-bin/carddisp.pl?gene=FN1 |
| KLRK1 | Killer Cell Lectin Like Receptor K1 | Protein Coding | 40 | GC12M013865 | 13.03 | https://www.genecards.org/cgi-bin/carddisp.pl?gene=KLRK1 |
| FOXD2-AS1 | FOXD2 Adjacent Opposite Strand RNA 1 | RNA Gene | 16 | GC01M047432 | 12.99 | https://www.genecards.org/cgi-bin/carddisp.pl?gene=FOXD2-AS1 |
| CTNNA1 | Catenin Alpha 1 | Protein Coding | 47 | GC05P138613 | 12.91 | https://www.genecards.org/cgi-bin/carddisp.pl?gene=CTNNA1 |
| TERC | Telomerase RNA Component | RNA Gene | 28 | GC03M169765 | 12.91 | https://www.genecards.org/cgi-bin/carddisp.pl?gene=TERC |
| IGF1R | Insulin Like Growth Factor 1 Receptor | Protein Coding | 54 | GC15P098648 | 12.89 | https://www.genecards.org/cgi-bin/carddisp.pl?gene=IGF1R |
| AURKA | Aurora Kinase A | Protein Coding | 50 | GC20M056370 | 12.82 | https://www.genecards.org/cgi-bin/carddisp.pl?gene=AURKA |
| MIR150 | MicroRNA 150 | RNA Gene | 21 | GC19M049500 | 12.79 | https://www.genecards.org/cgi-bin/carddisp.pl?gene=MIR150 |
| CYP3A4 | Cytochrome P450 Family 3 Subfamily A Member 4 | Protein Coding | 48 | GC07M099759 | 12.79 | https://www.genecards.org/cgi-bin/carddisp.pl?gene=CYP3A4 |
| MIR196A1 | MicroRNA 196a-1 | RNA Gene | 19 | GC17M048632 | 12.79 | https://www.genecards.org/cgi-bin/carddisp.pl?gene=MIR196A1 |
| IL2 | Interleukin 2 | Protein Coding | 45 | GC04M122451 | 12.73 | https://www.genecards.org/cgi-bin/carddisp.pl?gene=IL2 |
| TGFBR1 | Transforming Growth Factor Beta Receptor 1 | Protein Coding | 52 | GC09P099104 | 12.72 | https://www.genecards.org/cgi-bin/carddisp.pl?gene=TGFBR1 |
| FBXW7 | F-Box And WD Repeat Domain Containing 7 | Protein Coding | 44 | GC04M152321 | 12.72 | https://www.genecards.org/cgi-bin/carddisp.pl?gene=FBXW7 |
| LINC00460 | Long Intergenic Non-Protein Coding RNA 460 | RNA Gene | 16 | GC13P106376 | 12.72 | https://www.genecards.org/cgi-bin/carddisp.pl?gene=LINC00460 |
| MIR99A | MicroRNA 99a | RNA Gene | 21 | GC21P016539 | 12.56 | https://www.genecards.org/cgi-bin/carddisp.pl?gene=MIR99A |
| FOXCUT | FOXC1 Upstream Transcript | RNA Gene | 12 | GC06P001604 | 12.52 | https://www.genecards.org/cgi-bin/carddisp.pl?gene=FOXCUT |
| FH | Fumarate Hydratase | Protein Coding | 45 | GC01M241499 | 12.48 | https://www.genecards.org/cgi-bin/carddisp.pl?gene=FH |
| SYP | Synaptophysin | Protein Coding | 43 | GC0XM049187 | 12.38 | https://www.genecards.org/cgi-bin/carddisp.pl?gene=SYP |
| RAD51 | RAD51 Recombinase | Protein Coding | 52 | GC15P040694 | 12.38 | https://www.genecards.org/cgi-bin/carddisp.pl?gene=RAD51 |
| ABCB1 | ATP Binding Cassette Subfamily B Member 1 | Protein Coding | 51 | GC07M087504 | 12.31 | https://www.genecards.org/cgi-bin/carddisp.pl?gene=ABCB1 |
| CHGA | Chromogranin A | Protein Coding | 42 | GC14P092923 | 12.3 | https://www.genecards.org/cgi-bin/carddisp.pl?gene=CHGA |
| MIR204 | MicroRNA 204 | RNA Gene | 21 | GC09M070809 | 12.24 | https://www.genecards.org/cgi-bin/carddisp.pl?gene=MIR204 |
| ICOSLG | Inducible T Cell Costimulator Ligand | Protein Coding | 39 | GC21M044222 | 12.18 | https://www.genecards.org/cgi-bin/carddisp.pl?gene=ICOSLG |
| HGF | Hepatocyte Growth Factor | Protein Coding | 52 | GC07M081699 | 12.17 | https://www.genecards.org/cgi-bin/carddisp.pl?gene=HGF |
| FOLR1 | Folate Receptor Alpha | Protein Coding | 47 | GC11P072190 | 12.14 | https://www.genecards.org/cgi-bin/carddisp.pl?gene=FOLR1 |
| MIR378A | MicroRNA 378a | RNA Gene | 18 | GC05P149732 | 12.09 | https://www.genecards.org/cgi-bin/carddisp.pl?gene=MIR378A |
| INS | Insulin | Protein Coding | 48 | GC11M002159 | 12.03 | https://www.genecards.org/cgi-bin/carddisp.pl?gene=INS |
| NCAM1 | Neural Cell Adhesion Molecule 1 | Protein Coding | 45 | GC11P112961 | 11.98 | https://www.genecards.org/cgi-bin/carddisp.pl?gene=NCAM1 |
| BAP1 | BRCA1 Associated Protein 1 | Protein Coding | 45 | GC03M052401 | 11.93 | https://www.genecards.org/cgi-bin/carddisp.pl?gene=BAP1 |
| MIR203A | MicroRNA 203a | RNA Gene | 19 | GC14P104655 | 11.92 | https://www.genecards.org/cgi-bin/carddisp.pl?gene=MIR203A |
| JUP | Junction Plakoglobin | Protein Coding | 47 | GC17M041754 | 11.9 | https://www.genecards.org/cgi-bin/carddisp.pl?gene=JUP |
| GPT | Glutamic--Pyruvic Transaminase | Protein Coding | 41 | GC08P144502 | 11.89 | https://www.genecards.org/cgi-bin/carddisp.pl?gene=GPT |
| FBXO30 | F-Box Protein 30 | Protein Coding | 32 | GC06M145795 | 11.85 | https://www.genecards.org/cgi-bin/carddisp.pl?gene=FBXO30 |
| SMO | Smoothened, Frizzled Class Receptor | Protein Coding | 48 | GC07P129463 | 11.85 | https://www.genecards.org/cgi-bin/carddisp.pl?gene=SMO |
| NFKB1 | Nuclear Factor Kappa B Subunit 1 | Protein Coding | 52 | GC04P102501 | 11.84 | https://www.genecards.org/cgi-bin/carddisp.pl?gene=NFKB1 |
| JAK1 | Janus Kinase 1 | Protein Coding | 50 | GC01M064833 | 11.81 | https://www.genecards.org/cgi-bin/carddisp.pl?gene=JAK1 |
| AFP | Alpha Fetoprotein | Protein Coding | 45 | GC04P073431 | 11.78 | https://www.genecards.org/cgi-bin/carddisp.pl?gene=AFP |
| TNFSF10 | TNF Superfamily Member 10 | Protein Coding | 46 | GC03M172505 | 11.76 | https://www.genecards.org/cgi-bin/carddisp.pl?gene=TNFSF10 |
| FLT1 | Fms Related Receptor Tyrosine Kinase 1 | Protein Coding | 51 | GC13M028300 | 11.74 | https://www.genecards.org/cgi-bin/carddisp.pl?gene=FLT1 |
| MIRLET7A1 | MicroRNA Let-7a-1 | RNA Gene | 21 | GC09P094175 | 11.74 | https://www.genecards.org/cgi-bin/carddisp.pl?gene=MIRLET7A1 |
| SLC2A1 | Solute Carrier Family 2 Member 1 | Protein Coding | 52 | GC01M042925 | 11.71 | https://www.genecards.org/cgi-bin/carddisp.pl?gene=SLC2A1 |
| CYP1A1 | Cytochrome P450 Family 1 Subfamily A Member 1 | Protein Coding | 47 | GC15M074719 | 11.71 | https://www.genecards.org/cgi-bin/carddisp.pl?gene=CYP1A1 |
| CD99 | CD99 Molecule (Xg Blood Group) | Protein Coding | 40 | GC0XP002691 | 11.7 | https://www.genecards.org/cgi-bin/carddisp.pl?gene=CD99 |
| MIR200B | MicroRNA 200b | RNA Gene | 20 | GC01P001167 | 11.65 | https://www.genecards.org/cgi-bin/carddisp.pl?gene=MIR200B |
| MAPK3 | Mitogen-Activated Protein Kinase 3 | Protein Coding | 49 | GC16M030117 | 11.59 | https://www.genecards.org/cgi-bin/carddisp.pl?gene=MAPK3 |
| XRCC3 | X-Ray Repair Cross Complementing 3 | Protein Coding | 41 | GC14M103697 | 11.58 | https://www.genecards.org/cgi-bin/carddisp.pl?gene=XRCC3 |
| MVK | Mevalonate Kinase | Protein Coding | 48 | GC12P109573 | 11.53 | https://www.genecards.org/cgi-bin/carddisp.pl?gene=MVK |
| JAK2 | Janus Kinase 2 | Protein Coding | 54 | GC09P004985 | 11.5 | https://www.genecards.org/cgi-bin/carddisp.pl?gene=JAK2 |
| IL13 | Interleukin 13 | Protein Coding | 44 | GC05P132656 | 11.49 | https://www.genecards.org/cgi-bin/carddisp.pl?gene=IL13 |
| MIR222 | MicroRNA 222 | RNA Gene | 21 | GC0XM045747 | 11.44 | https://www.genecards.org/cgi-bin/carddisp.pl?gene=MIR222 |
| BPIFB1 | BPI Fold Containing Family B Member 1 | Protein Coding | 33 | GC20P033273 | 11.42 | https://www.genecards.org/cgi-bin/carddisp.pl?gene=BPIFB1 |
| TYMP | Thymidine Phosphorylase | Protein Coding | 46 | GC22M050525 | 11.33 | https://www.genecards.org/cgi-bin/carddisp.pl?gene=TYMP |
| CCL5 | C-C Motif Chemokine Ligand 5 | Protein Coding | 43 | GC17M035871 | 11.31 | https://www.genecards.org/cgi-bin/carddisp.pl?gene=CCL5 |
| BCL3 | BCL3 Transcription Coactivator | Protein Coding | 41 | GC19P044747 | 11.3 | https://www.genecards.org/cgi-bin/carddisp.pl?gene=BCL3 |
| MSH3 | MutS Homolog 3 | Protein Coding | 43 | GC05P080654 | 11.27 | https://www.genecards.org/cgi-bin/carddisp.pl?gene=MSH3 |
| MIR23A | MicroRNA 23a | RNA Gene | 20 | GC19M014011 | 11.24 | https://www.genecards.org/cgi-bin/carddisp.pl?gene=MIR23A |
| SPP1 | Secreted Phosphoprotein 1 | Protein Coding | 46 | GC04P087975 | 11.22 | https://www.genecards.org/cgi-bin/carddisp.pl?gene=SPP1 |
| HLA-DRB1 | Major Histocompatibility Complex, Class II, DR Beta 1 | Protein Coding | 46 | GC06M032578 | 11.21 | https://www.genecards.org/cgi-bin/carddisp.pl?gene=HLA-DRB1 |
| MIR216A | MicroRNA 216a | RNA Gene | 19 | GC02M055988 | 11.19 | https://www.genecards.org/cgi-bin/carddisp.pl?gene=MIR216A |
| MIR210 | MicroRNA 210 | RNA Gene | 21 | GC11M000674 | 11.16 | https://www.genecards.org/cgi-bin/carddisp.pl?gene=MIR210 |
| MIR122 | MicroRNA 122 | RNA Gene | 21 | GC18P058451 | 11.15 | https://www.genecards.org/cgi-bin/carddisp.pl?gene=MIR122 |
| WWOX | WW Domain Containing Oxidoreductase | Protein Coding | 47 | GC16P078099 | 11.14 | https://www.genecards.org/cgi-bin/carddisp.pl?gene=WWOX |
| CD4 | CD4 Molecule | Protein Coding | 49 | GC12P006786 | 11.03 | https://www.genecards.org/cgi-bin/carddisp.pl?gene=CD4 |
| MIR490 | MicroRNA 490 | RNA Gene | 16 | GC07P136903 | 10.98 | https://www.genecards.org/cgi-bin/carddisp.pl?gene=MIR490 |
| NBN | Nibrin | Protein Coding | 47 | GC08M089933 | 10.96 | https://www.genecards.org/cgi-bin/carddisp.pl?gene=NBN |
| TLR9 | Toll Like Receptor 9 | Protein Coding | 45 | GC03M052222 | 10.93 | https://www.genecards.org/cgi-bin/carddisp.pl?gene=TLR9 |
| DICER1 | Dicer 1, Ribonuclease III | Protein Coding | 47 | GC14M095086 | 10.9 | https://www.genecards.org/cgi-bin/carddisp.pl?gene=DICER1 |
| IFNB1 | Interferon Beta 1 | Protein Coding | 41 | GC09M021077 | 10.9 | https://www.genecards.org/cgi-bin/carddisp.pl?gene=IFNB1 |
| MIR183 | MicroRNA 183 | RNA Gene | 18 | GC07M129789 | 10.85 | https://www.genecards.org/cgi-bin/carddisp.pl?gene=MIR183 |
| MIR223 | MicroRNA 223 | RNA Gene | 21 | GC0XP066018 | 10.82 | https://www.genecards.org/cgi-bin/carddisp.pl?gene=MIR223 |
| MIR18A | MicroRNA 18a | RNA Gene | 18 | GC13P091432 | 10.79 | https://www.genecards.org/cgi-bin/carddisp.pl?gene=MIR18A |
| PSMB8 | Proteasome 20S Subunit Beta 8 | Protein Coding | 49 | GC06M032840 | 10.76 | https://www.genecards.org/cgi-bin/carddisp.pl?gene=PSMB8 |
| OSCP1 | Organic Solute Carrier Partner 1 | Protein Coding | 35 | GC01M036415 | 10.74 | https://www.genecards.org/cgi-bin/carddisp.pl?gene=OSCP1 |
| CAV1 | Caveolin 1 | Protein Coding | 48 | GC07P116524 | 10.73 | https://www.genecards.org/cgi-bin/carddisp.pl?gene=CAV1 |
| MIR106B | MicroRNA 106b | RNA Gene | 21 | GC07M100284 | 10.64 | https://www.genecards.org/cgi-bin/carddisp.pl?gene=MIR106B |
| BMP2 | Bone Morphogenetic Protein 2 | Protein Coding | 47 | GC20P006696 | 10.64 | https://www.genecards.org/cgi-bin/carddisp.pl?gene=BMP2 |
| MIR29A | MicroRNA 29a | RNA Gene | 21 | GC07M130876 | 10.59 | https://www.genecards.org/cgi-bin/carddisp.pl?gene=MIR29A |
| NUTM1 | NUT Midline Carcinoma Family Member 1 | Protein Coding | 33 | GC15P034343 | 10.51 | https://www.genecards.org/cgi-bin/carddisp.pl?gene=NUTM1 |
| COLQ | Collagen Like Tail Subunit Of Asymmetric Acetylcholinesterase | Protein Coding | 39 | GC03M015815 | 10.5 | https://www.genecards.org/cgi-bin/carddisp.pl?gene=COLQ |
| IFI27 | Interferon Alpha Inducible Protein 27 | Protein Coding | 37 | GC14P094104 | 10.47 | https://www.genecards.org/cgi-bin/carddisp.pl?gene=IFI27 |
| TYMS | Thymidylate Synthetase | Protein Coding | 47 | GC18P000657 | 10.42 | https://www.genecards.org/cgi-bin/carddisp.pl?gene=TYMS |
| CR2 | Complement C3d Receptor 2 | Protein Coding | 44 | GC01P207454 | 10.41 | https://www.genecards.org/cgi-bin/carddisp.pl?gene=CR2 |
| MIR28 | MicroRNA 28 | RNA Gene | 19 | GC03P188688 | 10.37 | https://www.genecards.org/cgi-bin/carddisp.pl?gene=MIR28 |
| CHEK1 | Checkpoint Kinase 1 | Protein Coding | 50 | GC11P125625 | 10.36 | https://www.genecards.org/cgi-bin/carddisp.pl?gene=CHEK1 |
| CD8A | CD8a Molecule | Protein Coding | 46 | GC02M086784 | 10.36 | https://www.genecards.org/cgi-bin/carddisp.pl?gene=CD8A |
| PDLIM7 | PDZ And LIM Domain 7 | Protein Coding | 40 | GC05M177483 | 10.32 | https://www.genecards.org/cgi-bin/carddisp.pl?gene=PDLIM7 |
| KLF6 | Kruppel Like Factor 6 | Protein Coding | 44 | GC10M003779 | 10.31 | https://www.genecards.org/cgi-bin/carddisp.pl?gene=KLF6 |
| RASA1 | RAS P21 Protein Activator 1 | Protein Coding | 46 | GC05P087267 | 10.28 | https://www.genecards.org/cgi-bin/carddisp.pl?gene=RASA1 |
| TIMP2 | TIMP Metallopeptidase Inhibitor 2 | Protein Coding | 44 | GC17M078852 | 10.27 | https://www.genecards.org/cgi-bin/carddisp.pl?gene=TIMP2 |
| TBX1 | T-Box Transcription Factor 1 | Protein Coding | 42 | GC22P019747 | 10.25 | https://www.genecards.org/cgi-bin/carddisp.pl?gene=TBX1 |
| ITGB4 | Integrin Subunit Beta 4 | Protein Coding | 48 | GC17P075721 | 10.24 | https://www.genecards.org/cgi-bin/carddisp.pl?gene=ITGB4 |
| MMP7 | Matrix Metallopeptidase 7 | Protein Coding | 48 | GC11M102425 | 10.15 | https://www.genecards.org/cgi-bin/carddisp.pl?gene=MMP7 |
| S100B | S100 Calcium Binding Protein B | Protein Coding | 45 | GC21M047431 | 10.13 | https://www.genecards.org/cgi-bin/carddisp.pl?gene=S100B |
| NTRK3 | Neurotrophic Receptor Tyrosine Kinase 3 | Protein Coding | 51 | GC15M087859 | 10.13 | https://www.genecards.org/cgi-bin/carddisp.pl?gene=NTRK3 |
| PLAU | Plasminogen Activator, Urokinase | Protein Coding | 51 | GC10P073909 | 10.09 | https://www.genecards.org/cgi-bin/carddisp.pl?gene=PLAU |
| RARB | Retinoic Acid Receptor Beta | Protein Coding | 50 | GC03P024830 | 10.08 | https://www.genecards.org/cgi-bin/carddisp.pl?gene=RARB |
| RAC1 | Rac Family Small GTPase 1 | Protein Coding | 49 | GC07P006380 | 10.08 | https://www.genecards.org/cgi-bin/carddisp.pl?gene=RAC1 |
| MIR23B | MicroRNA 23b | RNA Gene | 20 | GC09P095085 | 10.02 | https://www.genecards.org/cgi-bin/carddisp.pl?gene=MIR23B |
| CDK2 | Cyclin Dependent Kinase 2 | Protein Coding | 52 | GC12P055966 | 9.94 | https://www.genecards.org/cgi-bin/carddisp.pl?gene=CDK2 |
| MIR181A1 | MicroRNA 181a-1 | RNA Gene | 18 | GC01M198860 | 9.94 | https://www.genecards.org/cgi-bin/carddisp.pl?gene=MIR181A1 |
| MIR330 | MicroRNA 330 | RNA Gene | 18 | GC19M045663 | 9.92 | https://www.genecards.org/cgi-bin/carddisp.pl?gene=MIR330 |
| MGMT | O-6-Methylguanine-DNA Methyltransferase | Protein Coding | 50 | GC10P129467 | 9.88 | https://www.genecards.org/cgi-bin/carddisp.pl?gene=MGMT |
| PLAUR | Plasminogen Activator, Urokinase Receptor | Protein Coding | 44 | GC19M043646 | 9.87 | https://www.genecards.org/cgi-bin/carddisp.pl?gene=PLAUR |
| ARL2BP | ADP Ribosylation Factor Like GTPase 2 Binding Protein | Protein Coding | 37 | GC16P057245 | 9.85 | https://www.genecards.org/cgi-bin/carddisp.pl?gene=ARL2BP |
| SP1 | Sp1 Transcription Factor | Protein Coding | 44 | GC12P053380 | 9.79 | https://www.genecards.org/cgi-bin/carddisp.pl?gene=SP1 |
| MMP14 | Matrix Metallopeptidase 14 | Protein Coding | 51 | GC14P025277 | 9.76 | https://www.genecards.org/cgi-bin/carddisp.pl?gene=MMP14 |
| RELA | RELA Proto-Oncogene, NF-KB Subunit | Protein Coding | 50 | GC11M065653 | 9.75 | https://www.genecards.org/cgi-bin/carddisp.pl?gene=RELA |
| ABCG2 | ATP Binding Cassette Subfamily G Member 2 (Junior Blood Group) | Protein Coding | 50 | GC04M088090 | 9.69 | https://www.genecards.org/cgi-bin/carddisp.pl?gene=ABCG2 |
| CDX2 | Caudal Type Homeobox 2 | Protein Coding | 43 | GC13M027962 | 9.68 | https://www.genecards.org/cgi-bin/carddisp.pl?gene=CDX2 |
| CYP11A1 | Cytochrome P450 Family 11 Subfamily A Member 1 | Protein Coding | 48 | GC15M074337 | 9.65 | https://www.genecards.org/cgi-bin/carddisp.pl?gene=CYP11A1 |
| MIR590 | MicroRNA 590 | RNA Gene | 18 | GC07P074191 | 9.63 | https://www.genecards.org/cgi-bin/carddisp.pl?gene=MIR590 |
| CDK6 | Cyclin Dependent Kinase 6 | Protein Coding | 54 | GC07M092604 | 9.63 | https://www.genecards.org/cgi-bin/carddisp.pl?gene=CDK6 |
| MIR20A | MicroRNA 20a | RNA Gene | 19 | GC13P091434 | 9.6 | https://www.genecards.org/cgi-bin/carddisp.pl?gene=MIR20A |
| DLEC1 | DLEC1 Cilia And Flagella Associated Protein | Protein Coding | 36 | GC03P038038 | 9.58 | https://www.genecards.org/cgi-bin/carddisp.pl?gene=DLEC1 |
| MIR96 | MicroRNA 96 | RNA Gene | 20 | GC07M129774 | 9.57 | https://www.genecards.org/cgi-bin/carddisp.pl?gene=MIR96 |
| DPP9 | Dipeptidyl Peptidase 9 | Protein Coding | 40 | GC19M004675 | 9.54 | https://www.genecards.org/cgi-bin/carddisp.pl?gene=DPP9 |
| CLDN7 | Claudin 7 | Protein Coding | 41 | GC17M007259 | 9.52 | https://www.genecards.org/cgi-bin/carddisp.pl?gene=CLDN7 |
| MIR132 | MicroRNA 132 | RNA Gene | 21 | GC17M002049 | 9.5 | https://www.genecards.org/cgi-bin/carddisp.pl?gene=MIR132 |
| TYR | Tyrosinase | Protein Coding | 47 | GC11P089177 | 9.48 | https://www.genecards.org/cgi-bin/carddisp.pl?gene=TYR |
| PTPRC | Protein Tyrosine Phosphatase Receptor Type C | Protein Coding | 51 | GC01P198607 | 9.47 | https://www.genecards.org/cgi-bin/carddisp.pl?gene=PTPRC |
| SOX9 | SRY-Box Transcription Factor 9 | Protein Coding | 47 | GC17P072121 | 9.47 | https://www.genecards.org/cgi-bin/carddisp.pl?gene=SOX9 |
| CREBBP | CREB Binding Protein | Protein Coding | 52 | GC16M003726 | 9.45 | https://www.genecards.org/cgi-bin/carddisp.pl?gene=CREBBP |
| IL7 | Interleukin 7 | Protein Coding | 42 | GC08M078689 | 9.44 | https://www.genecards.org/cgi-bin/carddisp.pl?gene=IL7 |
| MIR26A1 | MicroRNA 26a-1 | RNA Gene | 20 | GC03P037969 | 9.43 | https://www.genecards.org/cgi-bin/carddisp.pl?gene=MIR26A1 |
| TIMP1 | TIMP Metallopeptidase Inhibitor 1 | Protein Coding | 45 | GC0XP047583 | 9.4 | https://www.genecards.org/cgi-bin/carddisp.pl?gene=TIMP1 |
| MIR3936HG | MIR3936 Host Gene | RNA Gene | 11 | GC05M132312 | 9.38 | https://www.genecards.org/cgi-bin/carddisp.pl?gene=MIR3936HG |
| MIR574 | MicroRNA 574 | RNA Gene | 18 | GC04P038872 | 9.34 | https://www.genecards.org/cgi-bin/carddisp.pl?gene=MIR574 |
| CDK1 | Cyclin Dependent Kinase 1 | Protein Coding | 45 | GC10P060772 | 9.34 | https://www.genecards.org/cgi-bin/carddisp.pl?gene=CDK1 |
| HSP90AA1 | Heat Shock Protein 90 Alpha Family Class A Member 1 | Protein Coding | 48 | GC14M102080 | 9.32 | https://www.genecards.org/cgi-bin/carddisp.pl?gene=HSP90AA1 |
| CTTN | Cortactin | Protein Coding | 43 | GC11P070398 | 9.3 | https://www.genecards.org/cgi-bin/carddisp.pl?gene=CTTN |
| MIRLET7G | MicroRNA Let-7g | RNA Gene | 20 | GC03M052268 | 9.28 | https://www.genecards.org/cgi-bin/carddisp.pl?gene=MIRLET7G |
| TLR7 | Toll Like Receptor 7 | Protein Coding | 46 | GC0XP012867 | 9.26 | https://www.genecards.org/cgi-bin/carddisp.pl?gene=TLR7 |
| SMAD3 | SMAD Family Member 3 | Protein Coding | 49 | GC15P067063 | 9.25 | https://www.genecards.org/cgi-bin/carddisp.pl?gene=SMAD3 |
| ITGB1 | Integrin Subunit Beta 1 | Protein Coding | 50 | GC10M032900 | 9.21 | https://www.genecards.org/cgi-bin/carddisp.pl?gene=ITGB1 |
| BSG | Basigin (Ok Blood Group) | Protein Coding | 44 | GC19P000571 | 9.17 | https://www.genecards.org/cgi-bin/carddisp.pl?gene=BSG |
| XPA | XPA, DNA Damage Recognition And Repair Factor | Protein Coding | 45 | GC09M097635 | 9.16 | https://www.genecards.org/cgi-bin/carddisp.pl?gene=XPA |
| MCL1 | MCL1 Apoptosis Regulator, BCL2 Family Member | Protein Coding | 47 | GC01M150707 | 9.14 | https://www.genecards.org/cgi-bin/carddisp.pl?gene=MCL1 |
| SMIM10L2A | Small Integral Membrane Protein 10 Like 2A | Protein Coding | 18 | GC0XP135421 | 9.13 | https://www.genecards.org/cgi-bin/carddisp.pl?gene=SMIM10L2A |
| RPS24P1 | Ribosomal Protein S24 Pseudogene 1 | Pseudogene | 7 | GC0YP012244 | 9.13 | https://www.genecards.org/cgi-bin/carddisp.pl?gene=RPS24P1 |
| GSTT1 | Glutathione S-Transferase Theta 1 | Protein Coding | 32 | GC22Mi00270 | 9.11 | https://www.genecards.org/cgi-bin/carddisp.pl?gene=GSTT1 |
| DES | Desmin | Protein Coding | 48 | GC02P219418 | 9.08 | https://www.genecards.org/cgi-bin/carddisp.pl?gene=DES |
| DLC1 | DLC1 Rho GTPase Activating Protein | Protein Coding | 44 | GC08M013083 | 9.08 | https://www.genecards.org/cgi-bin/carddisp.pl?gene=DLC1 |
| CDKN1C | Cyclin Dependent Kinase Inhibitor 1C | Protein Coding | 47 | GC11M002887 | 9.06 | https://www.genecards.org/cgi-bin/carddisp.pl?gene=CDKN1C |
| CCNA2 | Cyclin A2 | Protein Coding | 44 | GC04M121816 | 9.02 | https://www.genecards.org/cgi-bin/carddisp.pl?gene=CCNA2 |
| MTAP | Methylthioadenosine Phosphorylase | Protein Coding | 47 | GC09P021792 | 9 | https://www.genecards.org/cgi-bin/carddisp.pl?gene=MTAP |
| TGFA | Transforming Growth Factor Alpha | Protein Coding | 46 | GC02M070447 | 8.98 | https://www.genecards.org/cgi-bin/carddisp.pl?gene=TGFA |
| XIAP | X-Linked Inhibitor Of Apoptosis | Protein Coding | 49 | GC0XP123859 | 8.94 | https://www.genecards.org/cgi-bin/carddisp.pl?gene=XIAP |
| MTA1 | Metastasis Associated 1 | Protein Coding | 42 | GC14P105419 | 8.91 | https://www.genecards.org/cgi-bin/carddisp.pl?gene=MTA1 |
| IGF1 | Insulin Like Growth Factor 1 | Protein Coding | 50 | GC12M102395 | 8.89 | https://www.genecards.org/cgi-bin/carddisp.pl?gene=IGF1 |
| E2F1 | E2F Transcription Factor 1 | Protein Coding | 43 | GC20M033675 | 8.86 | https://www.genecards.org/cgi-bin/carddisp.pl?gene=E2F1 |
| RSPRY1 | Ring Finger And SPRY Domain Containing 1 | Protein Coding | 37 | GC16P057187 | 8.85 | https://www.genecards.org/cgi-bin/carddisp.pl?gene=RSPRY1 |
| MIR25 | MicroRNA 25 | RNA Gene | 20 | GC07M100093 | 8.84 | https://www.genecards.org/cgi-bin/carddisp.pl?gene=MIR25 |
| DPP4 | Dipeptidyl Peptidase 4 | Protein Coding | 50 | GC02M161992 | 8.84 | https://www.genecards.org/cgi-bin/carddisp.pl?gene=DPP4 |
| NOS2 | Nitric Oxide Synthase 2 | Protein Coding | 49 | GC17M027756 | 8.82 | https://www.genecards.org/cgi-bin/carddisp.pl?gene=NOS2 |
| THBS1 | Thrombospondin 1 | Protein Coding | 44 | GC15P039581 | 8.8 | https://www.genecards.org/cgi-bin/carddisp.pl?gene=THBS1 |
| EIF4E | Eukaryotic Translation Initiation Factor 4E | Protein Coding | 50 | GC04M098871 | 8.75 | https://www.genecards.org/cgi-bin/carddisp.pl?gene=EIF4E |
| YAP1 | Yes1 Associated Transcriptional Regulator | Protein Coding | 47 | GC11P102110 | 8.72 | https://www.genecards.org/cgi-bin/carddisp.pl?gene=YAP1 |
| NQO1 | NAD(P)H Quinone Dehydrogenase 1 | Protein Coding | 49 | GC16M069706 | 8.71 | https://www.genecards.org/cgi-bin/carddisp.pl?gene=NQO1 |
| ANXA2 | Annexin A2 | Protein Coding | 48 | GC15M060347 | 8.7 | https://www.genecards.org/cgi-bin/carddisp.pl?gene=ANXA2 |
| LINC01315 | Long Intergenic Non-Protein Coding RNA 1315 | RNA Gene | 13 | GC22M045418 | 8.69 | https://www.genecards.org/cgi-bin/carddisp.pl?gene=LINC01315 |
| CFLAR | CASP8 And FADD Like Apoptosis Regulator | Protein Coding | 46 | GC02P201117 | 8.64 | https://www.genecards.org/cgi-bin/carddisp.pl?gene=CFLAR |
| CTSD | Cathepsin D | Protein Coding | 52 | GC11M001752 | 8.64 | https://www.genecards.org/cgi-bin/carddisp.pl?gene=CTSD |
| FOS | Fos Proto-Oncogene, AP-1 Transcription Factor Subunit | Protein Coding | 50 | GC14P075278 | 8.61 | https://www.genecards.org/cgi-bin/carddisp.pl?gene=FOS |
| EPHA2 | EPH Receptor A2 | Protein Coding | 52 | GC01M016124 | 8.61 | https://www.genecards.org/cgi-bin/carddisp.pl?gene=EPHA2 |
| MAPK14 | Mitogen-Activated Protein Kinase 14 | Protein Coding | 51 | GC06P047451 | 8.6 | https://www.genecards.org/cgi-bin/carddisp.pl?gene=MAPK14 |
| SOD2 | Superoxide Dismutase 2 | Protein Coding | 51 | GC06M159669 | 8.58 | https://www.genecards.org/cgi-bin/carddisp.pl?gene=SOD2 |
| MIR217 | MicroRNA 217 | RNA Gene | 18 | GC02M055982 | 8.58 | https://www.genecards.org/cgi-bin/carddisp.pl?gene=MIR217 |
| CDH3 | Cadherin 3 | Protein Coding | 47 | GC16P068637 | 8.57 | https://www.genecards.org/cgi-bin/carddisp.pl?gene=CDH3 |
| MIR31HG | MIR31 Host Gene | RNA Gene | 18 | GC09M021439 | 8.53 | https://www.genecards.org/cgi-bin/carddisp.pl?gene=MIR31HG |
| CCL26 | C-C Motif Chemokine Ligand 26 | Protein Coding | 37 | GC07M075769 | 8.52 | https://www.genecards.org/cgi-bin/carddisp.pl?gene=CCL26 |
| SKP2 | S-Phase Kinase Associated Protein 2 | Protein Coding | 44 | GC05P036103 | 8.51 | https://www.genecards.org/cgi-bin/carddisp.pl?gene=SKP2 |
| CFTR | CF Transmembrane Conductance Regulator | Protein Coding | 51 | GC07P117287 | 8.51 | https://www.genecards.org/cgi-bin/carddisp.pl?gene=CFTR |
| FOXO3 | Forkhead Box O3 | Protein Coding | 44 | GC06P108559 | 8.48 | https://www.genecards.org/cgi-bin/carddisp.pl?gene=FOXO3 |
| AXL | AXL Receptor Tyrosine Kinase | Protein Coding | 51 | GC19P041219 | 8.46 | https://www.genecards.org/cgi-bin/carddisp.pl?gene=AXL |
| PDPN | Podoplanin | Protein Coding | 40 | GC01P013583 | 8.46 | https://www.genecards.org/cgi-bin/carddisp.pl?gene=PDPN |
| GSK3B | Glycogen Synthase Kinase 3 Beta | Protein Coding | 50 | GC03M119821 | 8.46 | https://www.genecards.org/cgi-bin/carddisp.pl?gene=GSK3B |
| VEGFD | Vascular Endothelial Growth Factor D | Protein Coding | 34 | GC0XM015345 | 8.45 | https://www.genecards.org/cgi-bin/carddisp.pl?gene=VEGFD |
| CD40LG | CD40 Ligand | Protein Coding | 47 | GC0XP136649 | 8.45 | https://www.genecards.org/cgi-bin/carddisp.pl?gene=CD40LG |
| MIR16-1 | MicroRNA 16-1 | RNA Gene | 21 | GC13M050048 | 8.44 | https://www.genecards.org/cgi-bin/carddisp.pl?gene=MIR16-1 |
| ATR | ATR Serine/Threonine Kinase | Protein Coding | 51 | GC03M142449 | 8.43 | https://www.genecards.org/cgi-bin/carddisp.pl?gene=ATR |
| XPC | XPC Complex Subunit, DNA Damage Recognition And Repair Factor | Protein Coding | 45 | GC03M015787 | 8.41 | https://www.genecards.org/cgi-bin/carddisp.pl?gene=XPC |
| WNT5A | Wnt Family Member 5A | Protein Coding | 49 | GC03M055465 | 8.39 | https://www.genecards.org/cgi-bin/carddisp.pl?gene=WNT5A |
| MIR373 | MicroRNA 373 | RNA Gene | 20 | GC19P054634 | 8.33 | https://www.genecards.org/cgi-bin/carddisp.pl?gene=MIR373 |
| PHB | Prohibitin | Protein Coding | 47 | GC17M049404 | 8.32 | https://www.genecards.org/cgi-bin/carddisp.pl?gene=PHB |
| ODC1 | Ornithine Decarboxylase 1 | Protein Coding | 47 | GC02M010432 | 8.31 | https://www.genecards.org/cgi-bin/carddisp.pl?gene=ODC1 |
| BAK1 | BCL2 Antagonist/Killer 1 | Protein Coding | 44 | GC06M033572 | 8.3 | https://www.genecards.org/cgi-bin/carddisp.pl?gene=BAK1 |
| PTHLH | Parathyroid Hormone Like Hormone | Protein Coding | 45 | GC12M027959 | 8.29 | https://www.genecards.org/cgi-bin/carddisp.pl?gene=PTHLH |
| CCNE1 | Cyclin E1 | Protein Coding | 48 | GC19P029811 | 8.26 | https://www.genecards.org/cgi-bin/carddisp.pl?gene=CCNE1 |
| CD40 | CD40 Molecule | Protein Coding | 48 | GC20P046118 | 8.24 | https://www.genecards.org/cgi-bin/carddisp.pl?gene=CD40 |
| FOXM1 | Forkhead Box M1 | Protein Coding | 44 | GC12M002857 | 8.21 | https://www.genecards.org/cgi-bin/carddisp.pl?gene=FOXM1 |
| HMGA2 | High Mobility Group AT-Hook 2 | Protein Coding | 44 | GC12P065824 | 8.19 | https://www.genecards.org/cgi-bin/carddisp.pl?gene=HMGA2 |
| MIR451A | MicroRNA 451a | RNA Gene | 17 | GC17M028861 | 8.17 | https://www.genecards.org/cgi-bin/carddisp.pl?gene=MIR451A |
| SELP | Selectin P | Protein Coding | 45 | GC01M169558 | 8.15 | https://www.genecards.org/cgi-bin/carddisp.pl?gene=SELP |
| TNFRSF10A | TNF Receptor Superfamily Member 10a | Protein Coding | 45 | GC08M023190 | 8.13 | https://www.genecards.org/cgi-bin/carddisp.pl?gene=TNFRSF10A |
| TOP2A | DNA Topoisomerase II Alpha | Protein Coding | 50 | GC17M040388 | 8.12 | https://www.genecards.org/cgi-bin/carddisp.pl?gene=TOP2A |
| B2M | Beta-2-Microglobulin | Protein Coding | 48 | GC15P044711 | 8.09 | https://www.genecards.org/cgi-bin/carddisp.pl?gene=B2M |
| MIR24-2 | MicroRNA 24-2 | RNA Gene | 18 | GC19M014008 | 8.08 | https://www.genecards.org/cgi-bin/carddisp.pl?gene=MIR24-2 |
| AMACR | Alpha-Methylacyl-CoA Racemase | Protein Coding | 45 | GC05M033986 | 8.08 | https://www.genecards.org/cgi-bin/carddisp.pl?gene=AMACR |
| PROM1 | Prominin 1 | Protein Coding | 44 | GC04M015965 | 8.08 | https://www.genecards.org/cgi-bin/carddisp.pl?gene=PROM1 |
| PIK3CG | Phosphatidylinositol-4,5-Bisphosphate 3-Kinase Catalytic Subunit Gamma | Protein Coding | 48 | GC07P106865 | 8.06 | https://www.genecards.org/cgi-bin/carddisp.pl?gene=PIK3CG |
| SERPINB5 | Serpin Family B Member 5 | Protein Coding | 43 | GC18P063476 | 8.03 | https://www.genecards.org/cgi-bin/carddisp.pl?gene=SERPINB5 |
| CCL2 | C-C Motif Chemokine Ligand 2 | Protein Coding | 48 | GC17P034255 | 8.02 | https://www.genecards.org/cgi-bin/carddisp.pl?gene=CCL2 |
| PRKCA | Protein Kinase C Alpha | Protein Coding | 50 | GC17P066302 | 8.02 | https://www.genecards.org/cgi-bin/carddisp.pl?gene=PRKCA |
| MIR149 | MicroRNA 149 | RNA Gene | 21 | GC02P240456 | 8.02 | https://www.genecards.org/cgi-bin/carddisp.pl?gene=MIR149 |
| WRAP53 | WD Repeat Containing Antisense To TP53 | Protein Coding | 41 | GC17P008049 | 7.98 | https://www.genecards.org/cgi-bin/carddisp.pl?gene=WRAP53 |
| PPM1D | Protein Phosphatase, Mg2+/Mn2+ Dependent 1D | Protein Coding | 47 | GC17P060600 | 7.96 | https://www.genecards.org/cgi-bin/carddisp.pl?gene=PPM1D |
| HPSE | Heparanase | Protein Coding | 44 | GC04M083292 | 7.92 | https://www.genecards.org/cgi-bin/carddisp.pl?gene=HPSE |
| AP5M1 | Adaptor Related Protein Complex 5 Subunit Mu 1 | Protein Coding | 32 | GC14P057268 | 7.89 | https://www.genecards.org/cgi-bin/carddisp.pl?gene=AP5M1 |
| LINC01544 | Long Intergenic Non-Protein Coding RNA 1544 | RNA Gene | 12 | GC18P061748 | 7.89 | https://www.genecards.org/cgi-bin/carddisp.pl?gene=LINC01544 |
| LINC02577 | Long Intergenic Non-Protein Coding RNA 2577 | RNA Gene | 9 | GC07P106777 | 7.89 | https://www.genecards.org/cgi-bin/carddisp.pl?gene=LINC02577 |
| ENSG00000246731 |  | Uncategorized | 8 | GC17M074210 | 7.89 | https://www.genecards.org/cgi-bin/carddisp.pl?gene=ENSG00000246731 |
| ENSG00000203635 |  | RNA Gene | 7 | GC02M001624 | 7.89 | https://www.genecards.org/cgi-bin/carddisp.pl?gene=ENSG00000203635 |
| lnc-ZNF674-1 |  | RNA Gene | 4 | GC0XM046600 | 7.89 | https://www.genecards.org/cgi-bin/carddisp.pl?gene=lnc-ZNF674-1 |
| HMGB1 | High Mobility Group Box 1 | Protein Coding | 44 | GC13M030456 | 7.88 | https://www.genecards.org/cgi-bin/carddisp.pl?gene=HMGB1 |
| TOP1 | DNA Topoisomerase I | Protein Coding | 48 | GC20P041028 | 7.87 | https://www.genecards.org/cgi-bin/carddisp.pl?gene=TOP1 |
| TCF4 | Transcription Factor 4 | Protein Coding | 46 | GC18M055222 | 7.85 | https://www.genecards.org/cgi-bin/carddisp.pl?gene=TCF4 |
| MIR365A | MicroRNA 365a | RNA Gene | 17 | GC16P014309 | 7.82 | https://www.genecards.org/cgi-bin/carddisp.pl?gene=MIR365A |
| IRF1 | Interferon Regulatory Factor 1 | Protein Coding | 47 | GC05M132481 | 7.81 | https://www.genecards.org/cgi-bin/carddisp.pl?gene=IRF1 |
| STMN1 | Stathmin 1 | Protein Coding | 44 | GC01M025884 | 7.81 | https://www.genecards.org/cgi-bin/carddisp.pl?gene=STMN1 |
| MIR98 | MicroRNA 98 | RNA Gene | 17 | GC0XM053582 | 7.8 | https://www.genecards.org/cgi-bin/carddisp.pl?gene=MIR98 |
| PLK1 | Polo Like Kinase 1 | Protein Coding | 49 | GC16P023888 | 7.79 | https://www.genecards.org/cgi-bin/carddisp.pl?gene=PLK1 |
| ARID1A | AT-Rich Interaction Domain 1A | Protein Coding | 44 | GC01P026693 | 7.79 | https://www.genecards.org/cgi-bin/carddisp.pl?gene=ARID1A |
| PRKCD | Protein Kinase C Delta | Protein Coding | 53 | GC03P053156 | 7.77 | https://www.genecards.org/cgi-bin/carddisp.pl?gene=PRKCD |
| PRODH | Proline Dehydrogenase 1 | Protein Coding | 45 | GC22M018912 | 7.74 | https://www.genecards.org/cgi-bin/carddisp.pl?gene=PRODH |
| LAMB3 | Laminin Subunit Beta 3 | Protein Coding | 45 | GC01M209614 | 7.73 | https://www.genecards.org/cgi-bin/carddisp.pl?gene=LAMB3 |
| SPARC | Secreted Protein Acidic And Cysteine Rich | Protein Coding | 50 | GC05M151639 | 7.69 | https://www.genecards.org/cgi-bin/carddisp.pl?gene=SPARC |
| TRIM24 | Tripartite Motif Containing 24 | Protein Coding | 43 | GC07P138460 | 7.69 | https://www.genecards.org/cgi-bin/carddisp.pl?gene=TRIM24 |
| ZEB2 | Zinc Finger E-Box Binding Homeobox 2 | Protein Coding | 48 | GC02M144384 | 7.66 | https://www.genecards.org/cgi-bin/carddisp.pl?gene=ZEB2 |
| PRKN | Parkin RBR E3 Ubiquitin Protein Ligase | Protein Coding | 40 | GC06M161348 | 7.65 | https://www.genecards.org/cgi-bin/carddisp.pl?gene=PRKN |
| IGFBP3 | Insulin Like Growth Factor Binding Protein 3 | Protein Coding | 45 | GC07M045912 | 7.63 | https://www.genecards.org/cgi-bin/carddisp.pl?gene=IGFBP3 |
| MIR320A | MicroRNA 320a | RNA Gene | 20 | GC08M022246 | 7.63 | https://www.genecards.org/cgi-bin/carddisp.pl?gene=MIR320A |
| ETV6 | ETS Variant Transcription Factor 6 | Protein Coding | 46 | GC12P011649 | 7.62 | https://www.genecards.org/cgi-bin/carddisp.pl?gene=ETV6 |
| MMP13 | Matrix Metallopeptidase 13 | Protein Coding | 50 | GC11M102942 | 7.61 | https://www.genecards.org/cgi-bin/carddisp.pl?gene=MMP13 |
| DGCR5 | DiGeorge Syndrome Critical Region Gene 5 | RNA Gene | 20 | GC22P019556 | 7.61 | https://www.genecards.org/cgi-bin/carddisp.pl?gene=DGCR5 |
| PKM | Pyruvate Kinase M1/2 | Protein Coding | 45 | GC15M072199 | 7.6 | https://www.genecards.org/cgi-bin/carddisp.pl?gene=PKM |
| NAT2 | N-Acetyltransferase 2 | Protein Coding | 43 | GC08P018391 | 7.6 | https://www.genecards.org/cgi-bin/carddisp.pl?gene=NAT2 |
| HSPA5 | Heat Shock Protein Family A (Hsp70) Member 5 | Protein Coding | 47 | GC09M125234 | 7.58 | https://www.genecards.org/cgi-bin/carddisp.pl?gene=HSPA5 |
| RPS6KB1 | Ribosomal Protein S6 Kinase B1 | Protein Coding | 49 | GC17P059893 | 7.58 | https://www.genecards.org/cgi-bin/carddisp.pl?gene=RPS6KB1 |
| AAGAB | Alpha And Gamma Adaptin Binding Protein | Protein Coding | 40 | GC15M067200 | 7.57 | https://www.genecards.org/cgi-bin/carddisp.pl?gene=AAGAB |
| CTLA4 | Cytotoxic T-Lymphocyte Associated Protein 4 | Protein Coding | 45 | GC02P203867 | 7.56 | https://www.genecards.org/cgi-bin/carddisp.pl?gene=CTLA4 |
| MIRLET7C | MicroRNA Let-7c | RNA Gene | 21 | GC21P016551 | 7.56 | https://www.genecards.org/cgi-bin/carddisp.pl?gene=MIRLET7C |
| ANXA1 | Annexin A1 | Protein Coding | 49 | GC09P073151 | 7.54 | https://www.genecards.org/cgi-bin/carddisp.pl?gene=ANXA1 |
| MMP11 | Matrix Metallopeptidase 11 | Protein Coding | 45 | GC22P023768 | 7.54 | https://www.genecards.org/cgi-bin/carddisp.pl?gene=MMP11 |
| CD24 | CD24 Molecule | Protein Coding | 33 | GC06M106969 | 7.51 | https://www.genecards.org/cgi-bin/carddisp.pl?gene=CD24 |
| FSCN1 | Fascin Actin-Bundling Protein 1 | Protein Coding | 43 | GC07P005592 | 7.46 | https://www.genecards.org/cgi-bin/carddisp.pl?gene=FSCN1 |
| CDKN2C | Cyclin Dependent Kinase Inhibitor 2C | Protein Coding | 45 | GC01P050960 | 7.44 | https://www.genecards.org/cgi-bin/carddisp.pl?gene=CDKN2C |
| EIF4EBP1 | Eukaryotic Translation Initiation Factor 4E Binding Protein 1 | Protein Coding | 47 | GC08P038007 | 7.42 | https://www.genecards.org/cgi-bin/carddisp.pl?gene=EIF4EBP1 |
| MIR101-1 | MicroRNA 101-1 | RNA Gene | 18 | GC01M065058 | 7.42 | https://www.genecards.org/cgi-bin/carddisp.pl?gene=MIR101-1 |
| GCG | Glucagon | Protein Coding | 41 | GC02M162142 | 7.39 | https://www.genecards.org/cgi-bin/carddisp.pl?gene=GCG |
| KITLG | KIT Ligand | Protein Coding | 44 | GC12M088492 | 7.38 | https://www.genecards.org/cgi-bin/carddisp.pl?gene=KITLG |
| CSF1R | Colony Stimulating Factor 1 Receptor | Protein Coding | 52 | GC05M150053 | 7.38 | https://www.genecards.org/cgi-bin/carddisp.pl?gene=CSF1R |
| CD82 | CD82 Molecule | Protein Coding | 43 | GC11P044586 | 7.37 | https://www.genecards.org/cgi-bin/carddisp.pl?gene=CD82 |
| MIR130A | MicroRNA 130a | RNA Gene | 20 | GC11P057641 | 7.34 | https://www.genecards.org/cgi-bin/carddisp.pl?gene=MIR130A |
| TNFRSF8 | TNF Receptor Superfamily Member 8 | Protein Coding | 42 | GC01P012063 | 7.32 | https://www.genecards.org/cgi-bin/carddisp.pl?gene=TNFRSF8 |
| ITGA6 | Integrin Subunit Alpha 6 | Protein Coding | 49 | GC02P172427 | 7.32 | https://www.genecards.org/cgi-bin/carddisp.pl?gene=ITGA6 |
| GFAP | Glial Fibrillary Acidic Protein | Protein Coding | 47 | GC17M044905 | 7.3 | https://www.genecards.org/cgi-bin/carddisp.pl?gene=GFAP |
| MIR372 | MicroRNA 372 | RNA Gene | 16 | GC19P054631 | 7.27 | https://www.genecards.org/cgi-bin/carddisp.pl?gene=MIR372 |
| CTNND1 | Catenin Delta 1 | Protein Coding | 45 | GC11P057788 | 7.27 | https://www.genecards.org/cgi-bin/carddisp.pl?gene=CTNND1 |
| MIR152 | MicroRNA 152 | RNA Gene | 20 | GC17M048037 | 7.26 | https://www.genecards.org/cgi-bin/carddisp.pl?gene=MIR152 |
| HDAC1 | Histone Deacetylase 1 | Protein Coding | 49 | GC01P032292 | 7.25 | https://www.genecards.org/cgi-bin/carddisp.pl?gene=HDAC1 |
| SFRP1 | Secreted Frizzled Related Protein 1 | Protein Coding | 43 | GC08M041238 | 7.2 | https://www.genecards.org/cgi-bin/carddisp.pl?gene=SFRP1 |
| MIR196A2 | MicroRNA 196a-2 | RNA Gene | 21 | GC12P054200 | 7.18 | https://www.genecards.org/cgi-bin/carddisp.pl?gene=MIR196A2 |
| MIR425 | MicroRNA 425 | RNA Gene | 17 | GC03M049251 | 7.18 | https://www.genecards.org/cgi-bin/carddisp.pl?gene=MIR425 |
| IL6R | Interleukin 6 Receptor | Protein Coding | 48 | GC01P154405 | 7.13 | https://www.genecards.org/cgi-bin/carddisp.pl?gene=IL6R |
| CLPTM1L | CLPTM1 Like | Protein Coding | 36 | GC05M001317 | 7.12 | https://www.genecards.org/cgi-bin/carddisp.pl?gene=CLPTM1L |
| MIR429 | MicroRNA 429 | RNA Gene | 19 | GC01P001464 | 7.11 | https://www.genecards.org/cgi-bin/carddisp.pl?gene=MIR429 |
| LGALS1 | Galectin 1 | Protein Coding | 43 | GC22P037675 | 7.11 | https://www.genecards.org/cgi-bin/carddisp.pl?gene=LGALS1 |
| NTRK2 | Neurotrophic Receptor Tyrosine Kinase 2 | Protein Coding | 53 | GC09P084668 | 7.09 | https://www.genecards.org/cgi-bin/carddisp.pl?gene=NTRK2 |
| BRD4 | Bromodomain Containing 4 | Protein Coding | 44 | GC19M015236 | 7.07 | https://www.genecards.org/cgi-bin/carddisp.pl?gene=BRD4 |
| BAD | BCL2 Associated Agonist Of Cell Death | Protein Coding | 46 | GC11M064273 | 7.07 | https://www.genecards.org/cgi-bin/carddisp.pl?gene=BAD |
| CCND2 | Cyclin D2 | Protein Coding | 50 | GC12P008103 | 7.05 | https://www.genecards.org/cgi-bin/carddisp.pl?gene=CCND2 |
| MTHFR | Methylenetetrahydrofolate Reductase | Protein Coding | 47 | GC01M011785 | 7.04 | https://www.genecards.org/cgi-bin/carddisp.pl?gene=MTHFR |
| PDCD4 | Programmed Cell Death 4 | Protein Coding | 43 | GC10P110871 | 7.03 | https://www.genecards.org/cgi-bin/carddisp.pl?gene=PDCD4 |
| S100A4 | S100 Calcium Binding Protein A4 | Protein Coding | 44 | GC01M153543 | 7.02 | https://www.genecards.org/cgi-bin/carddisp.pl?gene=S100A4 |
| WNT1 | Wnt Family Member 1 | Protein Coding | 46 | GC12P049053 | 7.01 | https://www.genecards.org/cgi-bin/carddisp.pl?gene=WNT1 |
| SERPINE1 | Serpin Family E Member 1 | Protein Coding | 50 | GC07P101127 | 7 | https://www.genecards.org/cgi-bin/carddisp.pl?gene=SERPINE1 |
| HLA-G | Major Histocompatibility Complex, Class I, G | Protein Coding | 44 | GC06P047256 | 7 | https://www.genecards.org/cgi-bin/carddisp.pl?gene=HLA-G |
| CTSB | Cathepsin B | Protein Coding | 51 | GC08M011842 | 7 | https://www.genecards.org/cgi-bin/carddisp.pl?gene=CTSB |
| NANOG | Nanog Homeobox | Protein Coding | 39 | GC12P007787 | 6.99 | https://www.genecards.org/cgi-bin/carddisp.pl?gene=NANOG |
| EGR1 | Early Growth Response 1 | Protein Coding | 44 | GC05P138465 | 6.99 | https://www.genecards.org/cgi-bin/carddisp.pl?gene=EGR1 |
| CLU | Clusterin | Protein Coding | 46 | GC08M027596 | 6.97 | https://www.genecards.org/cgi-bin/carddisp.pl?gene=CLU |
| MAD1L1 | Mitotic Arrest Deficient 1 Like 1 | Protein Coding | 45 | GC07M001815 | 6.95 | https://www.genecards.org/cgi-bin/carddisp.pl?gene=MAD1L1 |
| BIRC3 | Baculoviral IAP Repeat Containing 3 | Protein Coding | 46 | GC11P102317 | 6.95 | https://www.genecards.org/cgi-bin/carddisp.pl?gene=BIRC3 |
| JAK3 | Janus Kinase 3 | Protein Coding | 51 | GC19M017824 | 6.95 | https://www.genecards.org/cgi-bin/carddisp.pl?gene=JAK3 |
| LDHA | Lactate Dehydrogenase A | Protein Coding | 51 | GC11P018394 | 6.95 | https://www.genecards.org/cgi-bin/carddisp.pl?gene=LDHA |
| LOX | Lysyl Oxidase | Protein Coding | 44 | GC05M122063 | 6.94 | https://www.genecards.org/cgi-bin/carddisp.pl?gene=LOX |
| NRP1 | Neuropilin 1 | Protein Coding | 47 | GC10M033177 | 6.92 | https://www.genecards.org/cgi-bin/carddisp.pl?gene=NRP1 |
| RECK | Reversion Inducing Cysteine Rich Protein With Kazal Motifs | Protein Coding | 40 | GC09P036036 | 6.91 | https://www.genecards.org/cgi-bin/carddisp.pl?gene=RECK |
| LEF1 | Lymphoid Enhancer Binding Factor 1 | Protein Coding | 47 | GC04M108047 | 6.91 | https://www.genecards.org/cgi-bin/carddisp.pl?gene=LEF1 |
| ZFAS1 | ZNFX1 Antisense RNA 1 | RNA Gene | 18 | GC20P049276 | 6.89 | https://www.genecards.org/cgi-bin/carddisp.pl?gene=ZFAS1 |
| ACP3 | Acid Phosphatase 3 | Protein Coding | 35 | GC03P132319 | 6.88 | https://www.genecards.org/cgi-bin/carddisp.pl?gene=ACP3 |
| AKT3 | AKT Serine/Threonine Kinase 3 | Protein Coding | 52 | GC01M243488 | 6.84 | https://www.genecards.org/cgi-bin/carddisp.pl?gene=AKT3 |
| MIR188 | MicroRNA 188 | RNA Gene | 16 | GC0XP050003 | 6.84 | https://www.genecards.org/cgi-bin/carddisp.pl?gene=MIR188 |
| GDNF | Glial Cell Derived Neurotrophic Factor | Protein Coding | 47 | GC05M037812 | 6.83 | https://www.genecards.org/cgi-bin/carddisp.pl?gene=GDNF |
| CD34 | CD34 Molecule | Protein Coding | 43 | GC01M207880 | 6.8 | https://www.genecards.org/cgi-bin/carddisp.pl?gene=CD34 |
| IL5 | Interleukin 5 | Protein Coding | 44 | GC05M132541 | 6.77 | https://www.genecards.org/cgi-bin/carddisp.pl?gene=IL5 |
| RXRA | Retinoid X Receptor Alpha | Protein Coding | 49 | GC09P134317 | 6.77 | https://www.genecards.org/cgi-bin/carddisp.pl?gene=RXRA |
| CASP7 | Caspase 7 | Protein Coding | 50 | GC10P113679 | 6.77 | https://www.genecards.org/cgi-bin/carddisp.pl?gene=CASP7 |
| KLF4 | Kruppel Like Factor 4 | Protein Coding | 45 | GC09M107484 | 6.76 | https://www.genecards.org/cgi-bin/carddisp.pl?gene=KLF4 |
| MYB | MYB Proto-Oncogene, Transcription Factor | Protein Coding | 50 | GC06P135180 | 6.75 | https://www.genecards.org/cgi-bin/carddisp.pl?gene=MYB |
| PEBP1 | Phosphatidylethanolamine Binding Protein 1 | Protein Coding | 44 | GC12P118135 | 6.71 | https://www.genecards.org/cgi-bin/carddisp.pl?gene=PEBP1 |
| GGT1 | Gamma-Glutamyltransferase 1 | Protein Coding | 46 | GC22P024927 | 6.69 | https://www.genecards.org/cgi-bin/carddisp.pl?gene=GGT1 |
| FASN | Fatty Acid Synthase | Protein Coding | 49 | GC17M082078 | 6.67 | https://www.genecards.org/cgi-bin/carddisp.pl?gene=FASN |
| GRB2 | Growth Factor Receptor Bound Protein 2 | Protein Coding | 49 | GC17M075318 | 6.67 | https://www.genecards.org/cgi-bin/carddisp.pl?gene=GRB2 |
| MIR615 | MicroRNA 615 | RNA Gene | 20 | GC12P054033 | 6.66 | https://www.genecards.org/cgi-bin/carddisp.pl?gene=MIR615 |
| ETV5 | ETS Variant Transcription Factor 5 | Protein Coding | 39 | GC03M186046 | 6.66 | https://www.genecards.org/cgi-bin/carddisp.pl?gene=ETV5 |
| SMAD7 | SMAD Family Member 7 | Protein Coding | 43 | GC18M048919 | 6.66 | https://www.genecards.org/cgi-bin/carddisp.pl?gene=SMAD7 |
| ENO1 | Enolase 1 | Protein Coding | 47 | GC01M008861 | 6.65 | https://www.genecards.org/cgi-bin/carddisp.pl?gene=ENO1 |
| CDC42 | Cell Division Cycle 42 | Protein Coding | 51 | GC01P022057 | 6.62 | https://www.genecards.org/cgi-bin/carddisp.pl?gene=CDC42 |
| NOTCH4 | Notch Receptor 4 | Protein Coding | 45 | GC06M032648 | 6.62 | https://www.genecards.org/cgi-bin/carddisp.pl?gene=NOTCH4 |
| CLDN4 | Claudin 4 | Protein Coding | 41 | GC07P073799 | 6.61 | https://www.genecards.org/cgi-bin/carddisp.pl?gene=CLDN4 |
| MAP2K4 | Mitogen-Activated Protein Kinase Kinase 4 | Protein Coding | 46 | GC17P012020 | 6.6 | https://www.genecards.org/cgi-bin/carddisp.pl?gene=MAP2K4 |
| CASC2 | Cancer Susceptibility 2 | RNA Gene | 25 | GC10P118046 | 6.59 | https://www.genecards.org/cgi-bin/carddisp.pl?gene=CASC2 |
| COMT | Catechol-O-Methyltransferase | Protein Coding | 51 | GC22P019941 | 6.58 | https://www.genecards.org/cgi-bin/carddisp.pl?gene=COMT |
| VDR | Vitamin D Receptor | Protein Coding | 51 | GC12M047841 | 6.57 | https://www.genecards.org/cgi-bin/carddisp.pl?gene=VDR |
| MIR138-1 | MicroRNA 138-1 | RNA Gene | 19 | GC03P044115 | 6.57 | https://www.genecards.org/cgi-bin/carddisp.pl?gene=MIR138-1 |
| SHH | Sonic Hedgehog Signaling Molecule | Protein Coding | 50 | GC07M155799 | 6.56 | https://www.genecards.org/cgi-bin/carddisp.pl?gene=SHH |
| MIR371A | MicroRNA 371a | RNA Gene | 16 | GC19P053787 | 6.55 | https://www.genecards.org/cgi-bin/carddisp.pl?gene=MIR371A |
| CCN2 | Cellular Communication Network Factor 2 | Protein Coding | 39 | GC06M131948 | 6.55 | https://www.genecards.org/cgi-bin/carddisp.pl?gene=CCN2 |
| MVP | Major Vault Protein | Protein Coding | 39 | GC16P030094 | 6.55 | https://www.genecards.org/cgi-bin/carddisp.pl?gene=MVP |
| HLA-DQB1 | Major Histocompatibility Complex, Class II, DQ Beta 1 | Protein Coding | 44 | GC06M032804 | 6.51 | https://www.genecards.org/cgi-bin/carddisp.pl?gene=HLA-DQB1 |
| MDC1 | Mediator Of DNA Damage Checkpoint 1 | Protein Coding | 40 | GC06M030896 | 6.5 | https://www.genecards.org/cgi-bin/carddisp.pl?gene=MDC1 |
| DIABLO | Diablo IAP-Binding Mitochondrial Protein | Protein Coding | 47 | GC12M122208 | 6.49 | https://www.genecards.org/cgi-bin/carddisp.pl?gene=DIABLO |
| HDAC9 | Histone Deacetylase 9 | Protein Coding | 46 | GC07P018086 | 6.49 | https://www.genecards.org/cgi-bin/carddisp.pl?gene=HDAC9 |
| CHUK | Component Of Inhibitor Of Nuclear Factor Kappa B Kinase Complex | Protein Coding | 52 | GC10M100188 | 6.48 | https://www.genecards.org/cgi-bin/carddisp.pl?gene=CHUK |
| BECN1 | Beclin 1 | Protein Coding | 46 | GC17M042810 | 6.48 | https://www.genecards.org/cgi-bin/carddisp.pl?gene=BECN1 |
| IL1R1 | Interleukin 1 Receptor Type 1 | Protein Coding | 45 | GC02P102136 | 6.47 | https://www.genecards.org/cgi-bin/carddisp.pl?gene=IL1R1 |
| ALDH1A1 | Aldehyde Dehydrogenase 1 Family Member A1 | Protein Coding | 47 | GC09M072900 | 6.47 | https://www.genecards.org/cgi-bin/carddisp.pl?gene=ALDH1A1 |
| DDR1 | Discoidin Domain Receptor Tyrosine Kinase 1 | Protein Coding | 47 | GC06P047288 | 6.45 | https://www.genecards.org/cgi-bin/carddisp.pl?gene=DDR1 |
| TCF7L2 | Transcription Factor 7 Like 2 | Protein Coding | 45 | GC10P112950 | 6.45 | https://www.genecards.org/cgi-bin/carddisp.pl?gene=TCF7L2 |
| PSCA | Prostate Stem Cell Antigen | Protein Coding | 39 | GC08P142670 | 6.43 | https://www.genecards.org/cgi-bin/carddisp.pl?gene=PSCA |
| SOCS1 | Suppressor Of Cytokine Signaling 1 | Protein Coding | 43 | GC16M011255 | 6.42 | https://www.genecards.org/cgi-bin/carddisp.pl?gene=SOCS1 |
| NOTCH3 | Notch Receptor 3 | Protein Coding | 49 | GC19M015159 | 6.42 | https://www.genecards.org/cgi-bin/carddisp.pl?gene=NOTCH3 |
| RNASE3 | Ribonuclease A Family Member 3 | Protein Coding | 40 | GC14P020891 | 6.41 | https://www.genecards.org/cgi-bin/carddisp.pl?gene=RNASE3 |
| MSMB | Microseminoprotein Beta | Protein Coding | 40 | GC10M046033 | 6.4 | https://www.genecards.org/cgi-bin/carddisp.pl?gene=MSMB |
| CYP2A6 | Cytochrome P450 Family 2 Subfamily A Member 6 | Protein Coding | 47 | GC19M040843 | 6.38 | https://www.genecards.org/cgi-bin/carddisp.pl?gene=CYP2A6 |
| MECOM | MDS1 And EVI1 Complex Locus | Protein Coding | 47 | GC03M169083 | 6.37 | https://www.genecards.org/cgi-bin/carddisp.pl?gene=MECOM |
| FOSL1 | FOS Like 1, AP-1 Transcription Factor Subunit | Protein Coding | 44 | GC11M065909 | 6.36 | https://www.genecards.org/cgi-bin/carddisp.pl?gene=FOSL1 |
| MIR608 | MicroRNA 608 | RNA Gene | 17 | GC10P100974 | 6.35 | https://www.genecards.org/cgi-bin/carddisp.pl?gene=MIR608 |
| GJA1 | Gap Junction Protein Alpha 1 | Protein Coding | 50 | GC06P121436 | 6.34 | https://www.genecards.org/cgi-bin/carddisp.pl?gene=GJA1 |
| TFAP2A | Transcription Factor AP-2 Alpha | Protein Coding | 47 | GC06M010393 | 6.33 | https://www.genecards.org/cgi-bin/carddisp.pl?gene=TFAP2A |
| HOTTIP | HOXA Distal Transcript Antisense RNA | RNA Gene | 22 | GC07P027198 | 6.33 | https://www.genecards.org/cgi-bin/carddisp.pl?gene=HOTTIP |
| BIRC7 | Baculoviral IAP Repeat Containing 7 | Protein Coding | 40 | GC20P063235 | 6.31 | https://www.genecards.org/cgi-bin/carddisp.pl?gene=BIRC7 |
| IL33 | Interleukin 33 | Protein Coding | 40 | GC09P006206 | 6.31 | https://www.genecards.org/cgi-bin/carddisp.pl?gene=IL33 |
| MIR124-1 | MicroRNA 124-1 | RNA Gene | 21 | GC08M009903 | 6.3 | https://www.genecards.org/cgi-bin/carddisp.pl?gene=MIR124-1 |
| DANCR | Differentiation Antagonizing Non-Protein Coding RNA | RNA Gene | 21 | GC04P052712 | 6.29 | https://www.genecards.org/cgi-bin/carddisp.pl?gene=DANCR |
| ID1 | Inhibitor Of DNA Binding 1, HLH Protein | Protein Coding | 43 | GC20P031605 | 6.26 | https://www.genecards.org/cgi-bin/carddisp.pl?gene=ID1 |
| PRKDC | Protein Kinase, DNA-Activated, Catalytic Subunit | Protein Coding | 49 | GC08M047773 | 6.23 | https://www.genecards.org/cgi-bin/carddisp.pl?gene=PRKDC |
| APEX1 | Apurinic/Apyrimidinic Endodeoxyribonuclease 1 | Protein Coding | 45 | GC14P020455 | 6.23 | https://www.genecards.org/cgi-bin/carddisp.pl?gene=APEX1 |
| SQSTM1 | Sequestosome 1 | Protein Coding | 48 | GC05P179806 | 6.22 | https://www.genecards.org/cgi-bin/carddisp.pl?gene=SQSTM1 |
| SFN | Stratifin | Protein Coding | 46 | GC01P026873 | 6.21 | https://www.genecards.org/cgi-bin/carddisp.pl?gene=SFN |
| CYP1B1 | Cytochrome P450 Family 1 Subfamily B Member 1 | Protein Coding | 48 | GC02M038034 | 6.21 | https://www.genecards.org/cgi-bin/carddisp.pl?gene=CYP1B1 |
| IL18 | Interleukin 18 | Protein Coding | 44 | GC11M112143 | 6.19 | https://www.genecards.org/cgi-bin/carddisp.pl?gene=IL18 |
| MIF | Macrophage Migration Inhibitory Factor | Protein Coding | 49 | GC22P023894 | 6.19 | https://www.genecards.org/cgi-bin/carddisp.pl?gene=MIF |
| FOXP3 | Forkhead Box P3 | Protein Coding | 46 | GC0XM049250 | 6.18 | https://www.genecards.org/cgi-bin/carddisp.pl?gene=FOXP3 |
| MIR375 | MicroRNA 375 | RNA Gene | 19 | GC02M219001 | 6.18 | https://www.genecards.org/cgi-bin/carddisp.pl?gene=MIR375 |
| POU5F1 | POU Class 5 Homeobox 1 | Protein Coding | 47 | GC06M031184 | 6.17 | https://www.genecards.org/cgi-bin/carddisp.pl?gene=POU5F1 |
| MIR125B1 | MicroRNA 125b-1 | RNA Gene | 21 | GC11M122100 | 6.15 | https://www.genecards.org/cgi-bin/carddisp.pl?gene=MIR125B1 |
| DHFR | Dihydrofolate Reductase | Protein Coding | 49 | GC05M080626 | 6.15 | https://www.genecards.org/cgi-bin/carddisp.pl?gene=DHFR |
| EDNRA | Endothelin Receptor Type A | Protein Coding | 49 | GC04P147480 | 6.13 | https://www.genecards.org/cgi-bin/carddisp.pl?gene=EDNRA |
| MIR206 | MicroRNA 206 | RNA Gene | 20 | GC06P052144 | 6.12 | https://www.genecards.org/cgi-bin/carddisp.pl?gene=MIR206 |
| TGFB2 | Transforming Growth Factor Beta 2 | Protein Coding | 50 | GC01P218345 | 6.12 | https://www.genecards.org/cgi-bin/carddisp.pl?gene=TGFB2 |
| SI | Sucrase-Isomaltase | Protein Coding | 43 | GC03M164978 | 6.1 | https://www.genecards.org/cgi-bin/carddisp.pl?gene=SI |
| NAPSA | Napsin A Aspartic Peptidase | Protein Coding | 41 | GC19M050385 | 6.09 | https://www.genecards.org/cgi-bin/carddisp.pl?gene=NAPSA |
| IVL | Involucrin | Protein Coding | 37 | GC01P152881 | 6.04 | https://www.genecards.org/cgi-bin/carddisp.pl?gene=IVL |
| UCHL1 | Ubiquitin C-Terminal Hydrolase L1 | Protein Coding | 51 | GC04P041256 | 6.01 | https://www.genecards.org/cgi-bin/carddisp.pl?gene=UCHL1 |
| MICA | MHC Class I Polypeptide-Related Sequence A | Protein Coding | 39 | GC06P031399 | 5.98 | https://www.genecards.org/cgi-bin/carddisp.pl?gene=MICA |
| IDO1 | Indoleamine 2,3-Dioxygenase 1 | Protein Coding | 45 | GC08P039891 | 5.96 | https://www.genecards.org/cgi-bin/carddisp.pl?gene=IDO1 |
| ITGAV | Integrin Subunit Alpha V | Protein Coding | 46 | GC02P186589 | 5.95 | https://www.genecards.org/cgi-bin/carddisp.pl?gene=ITGAV |
| PTK2B | Protein Tyrosine Kinase 2 Beta | Protein Coding | 49 | GC08P027311 | 5.93 | https://www.genecards.org/cgi-bin/carddisp.pl?gene=PTK2B |
| PTPRG | Protein Tyrosine Phosphatase Receptor Type G | Protein Coding | 41 | GC03P061522 | 5.9 | https://www.genecards.org/cgi-bin/carddisp.pl?gene=PTPRG |
| IRS1 | Insulin Receptor Substrate 1 | Protein Coding | 47 | GC02M226731 | 5.87 | https://www.genecards.org/cgi-bin/carddisp.pl?gene=IRS1 |
| XRCC2 | X-Ray Repair Cross Complementing 2 | Protein Coding | 42 | GC07M152644 | 5.86 | https://www.genecards.org/cgi-bin/carddisp.pl?gene=XRCC2 |
| IL2RA | Interleukin 2 Receptor Subunit Alpha | Protein Coding | 50 | GC10M006010 | 5.84 | https://www.genecards.org/cgi-bin/carddisp.pl?gene=IL2RA |
| AKR1B10 | Aldo-Keto Reductase Family 1 Member B10 | Protein Coding | 43 | GC07P134527 | 5.83 | https://www.genecards.org/cgi-bin/carddisp.pl?gene=AKR1B10 |
| TAP1 | Transporter 1, ATP Binding Cassette Subfamily B Member | Protein Coding | 47 | GC06M032865 | 5.81 | https://www.genecards.org/cgi-bin/carddisp.pl?gene=TAP1 |
| CLDN1 | Claudin 1 | Protein Coding | 47 | GC03M190305 | 5.8 | https://www.genecards.org/cgi-bin/carddisp.pl?gene=CLDN1 |
| CREB1 | CAMP Responsive Element Binding Protein 1 | Protein Coding | 48 | GC02P207529 | 5.78 | https://www.genecards.org/cgi-bin/carddisp.pl?gene=CREB1 |
| S100A7 | S100 Calcium Binding Protein A7 | Protein Coding | 40 | GC01M153457 | 5.77 | https://www.genecards.org/cgi-bin/carddisp.pl?gene=S100A7 |
| DOCK8 | Dedicator Of Cytokinesis 8 | Protein Coding | 43 | GC09P000214 | 5.77 | https://www.genecards.org/cgi-bin/carddisp.pl?gene=DOCK8 |
| FGF1 | Fibroblast Growth Factor 1 | Protein Coding | 48 | GC05M142555 | 5.76 | https://www.genecards.org/cgi-bin/carddisp.pl?gene=FGF1 |
| PRDM2 | PR/SET Domain 2 | Protein Coding | 40 | GC01P013702 | 5.75 | https://www.genecards.org/cgi-bin/carddisp.pl?gene=PRDM2 |
| TIAM1 | TIAM Rac1 Associated GEF 1 | Protein Coding | 44 | GC21M031118 | 5.75 | https://www.genecards.org/cgi-bin/carddisp.pl?gene=TIAM1 |
| EWSR1 | EWS RNA Binding Protein 1 | Protein Coding | 43 | GC22P029269 | 5.73 | https://www.genecards.org/cgi-bin/carddisp.pl?gene=EWSR1 |
| FOXO1 | Forkhead Box O1 | Protein Coding | 48 | GC13M040555 | 5.73 | https://www.genecards.org/cgi-bin/carddisp.pl?gene=FOXO1 |
| SOX4 | SRY-Box Transcription Factor 4 | Protein Coding | 43 | GC06P021593 | 5.72 | https://www.genecards.org/cgi-bin/carddisp.pl?gene=SOX4 |
| CSF1 | Colony Stimulating Factor 1 | Protein Coding | 43 | GC01P109911 | 5.71 | https://www.genecards.org/cgi-bin/carddisp.pl?gene=CSF1 |
| NNMT | Nicotinamide N-Methyltransferase | Protein Coding | 45 | GC11P114257 | 5.69 | https://www.genecards.org/cgi-bin/carddisp.pl?gene=NNMT |
| IL1A | Interleukin 1 Alpha | Protein Coding | 44 | GC02M112773 | 5.68 | https://www.genecards.org/cgi-bin/carddisp.pl?gene=IL1A |
| COPS5 | COP9 Signalosome Subunit 5 | Protein Coding | 42 | GC08M067043 | 5.68 | https://www.genecards.org/cgi-bin/carddisp.pl?gene=COPS5 |
| E2F3 | E2F Transcription Factor 3 | Protein Coding | 43 | GC06P020402 | 5.66 | https://www.genecards.org/cgi-bin/carddisp.pl?gene=E2F3 |
| AURKB | Aurora Kinase B | Protein Coding | 48 | GC17M008864 | 5.66 | https://www.genecards.org/cgi-bin/carddisp.pl?gene=AURKB |
| NDRG1 | N-Myc Downstream Regulated 1 | Protein Coding | 45 | GC08M133237 | 5.62 | https://www.genecards.org/cgi-bin/carddisp.pl?gene=NDRG1 |
| CHKA | Choline Kinase Alpha | Protein Coding | 41 | GC11M068052 | 5.62 | https://www.genecards.org/cgi-bin/carddisp.pl?gene=CHKA |
| GJB2 | Gap Junction Protein Beta 2 | Protein Coding | 47 | GC13M020187 | 5.56 | https://www.genecards.org/cgi-bin/carddisp.pl?gene=GJB2 |
| TNFRSF6B | TNF Receptor Superfamily Member 6b | Protein Coding | 43 | GC20P063696 | 5.56 | https://www.genecards.org/cgi-bin/carddisp.pl?gene=TNFRSF6B |
| MIR497 | MicroRNA 497 | RNA Gene | 17 | GC17M007022 | 5.54 | https://www.genecards.org/cgi-bin/carddisp.pl?gene=MIR497 |
| CXCR2 | C-X-C Motif Chemokine Receptor 2 | Protein Coding | 48 | GC02P218125 | 5.54 | https://www.genecards.org/cgi-bin/carddisp.pl?gene=CXCR2 |
| YBX1 | Y-Box Binding Protein 1 | Protein Coding | 39 | GC01P042682 | 5.53 | https://www.genecards.org/cgi-bin/carddisp.pl?gene=YBX1 |
| HSP90B1 | Heat Shock Protein 90 Beta Family Member 1 | Protein Coding | 45 | GC12P103930 | 5.53 | https://www.genecards.org/cgi-bin/carddisp.pl?gene=HSP90B1 |
| NCOA3 | Nuclear Receptor Coactivator 3 | Protein Coding | 45 | GC20P047501 | 5.53 | https://www.genecards.org/cgi-bin/carddisp.pl?gene=NCOA3 |
| FLNC | Filamin C | Protein Coding | 44 | GC07P128830 | 5.52 | https://www.genecards.org/cgi-bin/carddisp.pl?gene=FLNC |
| CXCR3 | C-X-C Motif Chemokine Receptor 3 | Protein Coding | 44 | GC0XM071615 | 5.51 | https://www.genecards.org/cgi-bin/carddisp.pl?gene=CXCR3 |
| HLA-C | Major Histocompatibility Complex, Class I, C | Protein Coding | 44 | GC06M031272 | 5.5 | https://www.genecards.org/cgi-bin/carddisp.pl?gene=HLA-C |
| MIR494 | MicroRNA 494 | RNA Gene | 17 | GC14P104814 | 5.49 | https://www.genecards.org/cgi-bin/carddisp.pl?gene=MIR494 |
| RACK1 | Receptor For Activated C Kinase 1 | Protein Coding | 37 | GC05M181310 | 5.49 | https://www.genecards.org/cgi-bin/carddisp.pl?gene=RACK1 |
| ANGPT1 | Angiopoietin 1 | Protein Coding | 45 | GC08M107246 | 5.49 | https://www.genecards.org/cgi-bin/carddisp.pl?gene=ANGPT1 |
| PIN1 | Peptidylprolyl Cis/Trans Isomerase, NIMA-Interacting 1 | Protein Coding | 47 | GC19P009835 | 5.47 | https://www.genecards.org/cgi-bin/carddisp.pl?gene=PIN1 |
| MIR550A1 | MicroRNA 550a-1 | RNA Gene | 16 | GC07P030329 | 5.46 | https://www.genecards.org/cgi-bin/carddisp.pl?gene=MIR550A1 |
| ITGA5 | Integrin Subunit Alpha 5 | Protein Coding | 48 | GC12M054396 | 5.45 | https://www.genecards.org/cgi-bin/carddisp.pl?gene=ITGA5 |
| RARS1 | Arginyl-TRNA Synthetase 1 | Protein Coding | 35 | GC05P168487 | 5.44 | https://www.genecards.org/cgi-bin/carddisp.pl?gene=RARS1 |
| MAPK10 | Mitogen-Activated Protein Kinase 10 | Protein Coding | 51 | GC04M085990 | 5.44 | https://www.genecards.org/cgi-bin/carddisp.pl?gene=MAPK10 |
| GIP | Gastric Inhibitory Polypeptide | Protein Coding | 39 | GC17M048958 | 5.41 | https://www.genecards.org/cgi-bin/carddisp.pl?gene=GIP |
| CLDN3 | Claudin 3 | Protein Coding | 40 | GC07M073768 | 5.41 | https://www.genecards.org/cgi-bin/carddisp.pl?gene=CLDN3 |
| PDGFB | Platelet Derived Growth Factor Subunit B | Protein Coding | 50 | GC22M045657 | 5.4 | https://www.genecards.org/cgi-bin/carddisp.pl?gene=PDGFB |
| XRCC6 | X-Ray Repair Cross Complementing 6 | Protein Coding | 45 | GC22P041622 | 5.4 | https://www.genecards.org/cgi-bin/carddisp.pl?gene=XRCC6 |
| WIF1 | WNT Inhibitory Factor 1 | Protein Coding | 45 | GC12M065050 | 5.39 | https://www.genecards.org/cgi-bin/carddisp.pl?gene=WIF1 |
| HK2 | Hexokinase 2 | Protein Coding | 47 | GC02P074833 | 5.38 | https://www.genecards.org/cgi-bin/carddisp.pl?gene=HK2 |
| CSNK2A1 | Casein Kinase 2 Alpha 1 | Protein Coding | 51 | GC20M000472 | 5.35 | https://www.genecards.org/cgi-bin/carddisp.pl?gene=CSNK2A1 |
| RSF1 | Remodeling And Spacing Factor 1 | Protein Coding | 35 | GC11M077659 | 5.34 | https://www.genecards.org/cgi-bin/carddisp.pl?gene=RSF1 |
| PML | PML Nuclear Body Scaffold | Protein Coding | 45 | GC15P073994 | 5.34 | https://www.genecards.org/cgi-bin/carddisp.pl?gene=PML |
| TSG101 | Tumor Susceptibility 101 | Protein Coding | 43 | GC11M018468 | 5.34 | https://www.genecards.org/cgi-bin/carddisp.pl?gene=TSG101 |
| FADD | Fas Associated Via Death Domain | Protein Coding | 47 | GC11P070203 | 5.29 | https://www.genecards.org/cgi-bin/carddisp.pl?gene=FADD |
| PTPN12 | Protein Tyrosine Phosphatase Non-Receptor Type 12 | Protein Coding | 45 | GC07P077537 | 5.29 | https://www.genecards.org/cgi-bin/carddisp.pl?gene=PTPN12 |
| FGF4 | Fibroblast Growth Factor 4 | Protein Coding | 44 | GC11M069762 | 5.28 | https://www.genecards.org/cgi-bin/carddisp.pl?gene=FGF4 |
| DNMT3A | DNA Methyltransferase 3 Alpha | Protein Coding | 51 | GC02M025228 | 5.27 | https://www.genecards.org/cgi-bin/carddisp.pl?gene=DNMT3A |
| MIR491 | MicroRNA 491 | RNA Gene | 18 | GC09P020716 | 5.27 | https://www.genecards.org/cgi-bin/carddisp.pl?gene=MIR491 |
| IL2RB | Interleukin 2 Receptor Subunit Beta | Protein Coding | 48 | GC22M037125 | 5.27 | https://www.genecards.org/cgi-bin/carddisp.pl?gene=IL2RB |
| TRAF3 | TNF Receptor Associated Factor 3 | Protein Coding | 47 | GC14P104639 | 5.27 | https://www.genecards.org/cgi-bin/carddisp.pl?gene=TRAF3 |
| S100A6 | S100 Calcium Binding Protein A6 | Protein Coding | 43 | GC01M153535 | 5.24 | https://www.genecards.org/cgi-bin/carddisp.pl?gene=S100A6 |
| CAT | Catalase | Protein Coding | 50 | GC11P034460 | 5.22 | https://www.genecards.org/cgi-bin/carddisp.pl?gene=CAT |
| MIR9-3 | MicroRNA 9-3 | RNA Gene | 19 | GC15P089363 | 5.22 | https://www.genecards.org/cgi-bin/carddisp.pl?gene=MIR9-3 |
| IKBKB | Inhibitor Of Nuclear Factor Kappa B Kinase Subunit Beta | Protein Coding | 52 | GC08P042271 | 5.22 | https://www.genecards.org/cgi-bin/carddisp.pl?gene=IKBKB |
| NOTCH2 | Notch Receptor 2 | Protein Coding | 50 | GC01M119911 | 5.2 | https://www.genecards.org/cgi-bin/carddisp.pl?gene=NOTCH2 |
| PCSK9 | Proprotein Convertase Subtilisin/Kexin Type 9 | Protein Coding | 51 | GC01P055039 | 5.2 | https://www.genecards.org/cgi-bin/carddisp.pl?gene=PCSK9 |
| PLCG1 | Phospholipase C Gamma 1 | Protein Coding | 47 | GC20P041136 | 5.18 | https://www.genecards.org/cgi-bin/carddisp.pl?gene=PLCG1 |
| GZMB | Granzyme B | Protein Coding | 45 | GC14M024630 | 5.18 | https://www.genecards.org/cgi-bin/carddisp.pl?gene=GZMB |
| TDGF1 | Teratocarcinoma-Derived Growth Factor 1 | Protein Coding | 41 | GC03P046576 | 5.18 | https://www.genecards.org/cgi-bin/carddisp.pl?gene=TDGF1 |
| SNHG1 | Small Nucleolar RNA Host Gene 1 | RNA Gene | 21 | GC11M063444 | 5.16 | https://www.genecards.org/cgi-bin/carddisp.pl?gene=SNHG1 |
| SOCS3 | Suppressor Of Cytokine Signaling 3 | Protein Coding | 44 | GC17M078356 | 5.15 | https://www.genecards.org/cgi-bin/carddisp.pl?gene=SOCS3 |
| MIR124-3 | MicroRNA 124-3 | RNA Gene | 17 | GC20P063180 | 5.15 | https://www.genecards.org/cgi-bin/carddisp.pl?gene=MIR124-3 |
| PRKAA1 | Protein Kinase AMP-Activated Catalytic Subunit Alpha 1 | Protein Coding | 47 | GC05M040759 | 5.14 | https://www.genecards.org/cgi-bin/carddisp.pl?gene=PRKAA1 |
| NES | Nestin | Protein Coding | 39 | GC01M156668 | 5.14 | https://www.genecards.org/cgi-bin/carddisp.pl?gene=NES |
| MAPK9 | Mitogen-Activated Protein Kinase 9 | Protein Coding | 48 | GC05M180234 | 5.14 | https://www.genecards.org/cgi-bin/carddisp.pl?gene=MAPK9 |
| PRL | Prolactin | Protein Coding | 44 | GC06M022230 | 5.13 | https://www.genecards.org/cgi-bin/carddisp.pl?gene=PRL |
| HLA-DQA1 | Major Histocompatibility Complex, Class II, DQ Alpha 1 | Protein Coding | 42 | GC06P047340 | 5.13 | https://www.genecards.org/cgi-bin/carddisp.pl?gene=HLA-DQA1 |
| RRM2 | Ribonucleotide Reductase Regulatory Subunit M2 | Protein Coding | 48 | GC02P010123 | 5.12 | https://www.genecards.org/cgi-bin/carddisp.pl?gene=RRM2 |
| MTDH | Metadherin | Protein Coding | 40 | GC08P097643 | 5.12 | https://www.genecards.org/cgi-bin/carddisp.pl?gene=MTDH |
| ITGA3 | Integrin Subunit Alpha 3 | Protein Coding | 46 | GC17P050055 | 5.09 | https://www.genecards.org/cgi-bin/carddisp.pl?gene=ITGA3 |
| CYP2D6 | Cytochrome P450 Family 2 Subfamily D Member 6 | Protein Coding | 48 | GC22M042126 | 5.07 | https://www.genecards.org/cgi-bin/carddisp.pl?gene=CYP2D6 |
| MIR135A1 | MicroRNA 135a-1 | RNA Gene | 20 | GC03M052296 | 5.06 | https://www.genecards.org/cgi-bin/carddisp.pl?gene=MIR135A1 |
| RPL22 | Ribosomal Protein L22 | Protein Coding | 43 | GC01M006179 | 5.06 | https://www.genecards.org/cgi-bin/carddisp.pl?gene=RPL22 |
| MCM2 | Minichromosome Maintenance Complex Component 2 | Protein Coding | 47 | GC03P127598 | 5.04 | https://www.genecards.org/cgi-bin/carddisp.pl?gene=MCM2 |
| ZFHX3 | Zinc Finger Homeobox 3 | Protein Coding | 41 | GC16M072782 | 5.03 | https://www.genecards.org/cgi-bin/carddisp.pl?gene=ZFHX3 |
| STC2 | Stanniocalcin 2 | Protein Coding | 40 | GC05M173314 | 5 | https://www.genecards.org/cgi-bin/carddisp.pl?gene=STC2 |
| TSLP | Thymic Stromal Lymphopoietin | Protein Coding | 38 | GC05P111070 | 4.99 | https://www.genecards.org/cgi-bin/carddisp.pl?gene=TSLP |
| MAD2L2 | Mitotic Arrest Deficient 2 Like 2 | Protein Coding | 44 | GC01M011674 | 4.99 | https://www.genecards.org/cgi-bin/carddisp.pl?gene=MAD2L2 |
| SOD1 | Superoxide Dismutase 1 | Protein Coding | 51 | GC21P031659 | 4.98 | https://www.genecards.org/cgi-bin/carddisp.pl?gene=SOD1 |
| CKS1B | CDC28 Protein Kinase Regulatory Subunit 1B | Protein Coding | 41 | GC01P154974 | 4.98 | https://www.genecards.org/cgi-bin/carddisp.pl?gene=CKS1B |
| CXCL9 | C-X-C Motif Chemokine Ligand 9 | Protein Coding | 39 | GC04M076001 | 4.97 | https://www.genecards.org/cgi-bin/carddisp.pl?gene=CXCL9 |
| NCOA1 | Nuclear Receptor Coactivator 1 | Protein Coding | 44 | GC02P024492 | 4.95 | https://www.genecards.org/cgi-bin/carddisp.pl?gene=NCOA1 |
| HLA-E | Major Histocompatibility Complex, Class I, E | Protein Coding | 41 | GC06P047281 | 4.95 | https://www.genecards.org/cgi-bin/carddisp.pl?gene=HLA-E |
| FOXA1 | Forkhead Box A1 | Protein Coding | 43 | GC14M037589 | 4.94 | https://www.genecards.org/cgi-bin/carddisp.pl?gene=FOXA1 |
| TPD52 | Tumor Protein D52 | Protein Coding | 40 | GC08M080034 | 4.94 | https://www.genecards.org/cgi-bin/carddisp.pl?gene=TPD52 |
| BRMS1 | BRMS1 Transcriptional Repressor And Anoikis Regulator | Protein Coding | 37 | GC11M066346 | 4.94 | https://www.genecards.org/cgi-bin/carddisp.pl?gene=BRMS1 |
| TCF7 | Transcription Factor 7 | Protein Coding | 44 | GC05P134114 | 4.92 | https://www.genecards.org/cgi-bin/carddisp.pl?gene=TCF7 |
| YY1 | YY1 Transcription Factor | Protein Coding | 48 | GC14P100238 | 4.92 | https://www.genecards.org/cgi-bin/carddisp.pl?gene=YY1 |
| BNIP3 | BCL2 Interacting Protein 3 | Protein Coding | 42 | GC10M131966 | 4.91 | https://www.genecards.org/cgi-bin/carddisp.pl?gene=BNIP3 |
| VTN | Vitronectin | Protein Coding | 44 | GC17M029955 | 4.91 | https://www.genecards.org/cgi-bin/carddisp.pl?gene=VTN |
| CCNG1 | Cyclin G1 | Protein Coding | 42 | GC05P163438 | 4.91 | https://www.genecards.org/cgi-bin/carddisp.pl?gene=CCNG1 |
| SATB1 | SATB Homeobox 1 | Protein Coding | 42 | GC03M018364 | 4.9 | https://www.genecards.org/cgi-bin/carddisp.pl?gene=SATB1 |
| EDN1 | Endothelin 1 | Protein Coding | 47 | GC06P012290 | 4.9 | https://www.genecards.org/cgi-bin/carddisp.pl?gene=EDN1 |
| ADAM10 | ADAM Metallopeptidase Domain 10 | Protein Coding | 52 | GC15M058588 | 4.89 | https://www.genecards.org/cgi-bin/carddisp.pl?gene=ADAM10 |
| ABCC3 | ATP Binding Cassette Subfamily C Member 3 | Protein Coding | 45 | GC17P050634 | 4.87 | https://www.genecards.org/cgi-bin/carddisp.pl?gene=ABCC3 |
| PLG | Plasminogen | Protein Coding | 48 | GC06P160702 | 4.86 | https://www.genecards.org/cgi-bin/carddisp.pl?gene=PLG |
| KRT10 | Keratin 10 | Protein Coding | 42 | GC17M040818 | 4.86 | https://www.genecards.org/cgi-bin/carddisp.pl?gene=KRT10 |
| MGAT5 | Alpha-1,6-Mannosylglycoprotein 6-Beta-N-Acetylglucosaminyltransferase | Protein Coding | 39 | GC02P134119 | 4.84 | https://www.genecards.org/cgi-bin/carddisp.pl?gene=MGAT5 |
| MAP3K8 | Mitogen-Activated Protein Kinase Kinase Kinase 8 | Protein Coding | 47 | GC10P030458 | 4.83 | https://www.genecards.org/cgi-bin/carddisp.pl?gene=MAP3K8 |
| CCNH | Cyclin H | Protein Coding | 45 | GC05M087311 | 4.82 | https://www.genecards.org/cgi-bin/carddisp.pl?gene=CCNH |
| LDLR | Low Density Lipoprotein Receptor | Protein Coding | 49 | GC19P011061 | 4.81 | https://www.genecards.org/cgi-bin/carddisp.pl?gene=LDLR |
| CDC25C | Cell Division Cycle 25C | Protein Coding | 48 | GC05M138285 | 4.81 | https://www.genecards.org/cgi-bin/carddisp.pl?gene=CDC25C |
| URGCP | Upregulator Of Cell Proliferation | Protein Coding | 34 | GC07M043876 | 4.78 | https://www.genecards.org/cgi-bin/carddisp.pl?gene=URGCP |
| MICB | MHC Class I Polypeptide-Related Sequence B | Protein Coding | 41 | GC06P047300 | 4.77 | https://www.genecards.org/cgi-bin/carddisp.pl?gene=MICB |
| DPP8 | Dipeptidyl Peptidase 8 | Protein Coding | 37 | GC15M065442 | 4.77 | https://www.genecards.org/cgi-bin/carddisp.pl?gene=DPP8 |
| PINX1 | PIN2 (TERF1) Interacting Telomerase Inhibitor 1 | Protein Coding | 39 | GC08M010726 | 4.75 | https://www.genecards.org/cgi-bin/carddisp.pl?gene=PINX1 |
| CD86 | CD86 Molecule | Protein Coding | 43 | GC03P122055 | 4.75 | https://www.genecards.org/cgi-bin/carddisp.pl?gene=CD86 |
| HDGF | Heparin Binding Growth Factor | Protein Coding | 40 | GC01M156754 | 4.75 | https://www.genecards.org/cgi-bin/carddisp.pl?gene=HDGF |
| MIR196B | MicroRNA 196b | RNA Gene | 20 | GC07M027260 | 4.73 | https://www.genecards.org/cgi-bin/carddisp.pl?gene=MIR196B |
| OPCML | Opioid Binding Protein/Cell Adhesion Molecule Like | Protein Coding | 41 | GC11M132414 | 4.71 | https://www.genecards.org/cgi-bin/carddisp.pl?gene=OPCML |
| HNRNPK | Heterogeneous Nuclear Ribonucleoprotein K | Protein Coding | 45 | GC09M084056 | 4.71 | https://www.genecards.org/cgi-bin/carddisp.pl?gene=HNRNPK |
| WEE1 | WEE1 G2 Checkpoint Kinase | Protein Coding | 46 | GC11P009573 | 4.7 | https://www.genecards.org/cgi-bin/carddisp.pl?gene=WEE1 |
| MIR449A | MicroRNA 449a | RNA Gene | 20 | GC05M055171 | 4.69 | https://www.genecards.org/cgi-bin/carddisp.pl?gene=MIR449A |
| DROSHA | Drosha Ribonuclease III | Protein Coding | 41 | GC05M031401 | 4.68 | https://www.genecards.org/cgi-bin/carddisp.pl?gene=DROSHA |
| KLF5 | Kruppel Like Factor 5 | Protein Coding | 44 | GC13P073054 | 4.66 | https://www.genecards.org/cgi-bin/carddisp.pl?gene=KLF5 |
| MIR7-1 | MicroRNA 7-1 | RNA Gene | 18 | GC09M084057 | 4.63 | https://www.genecards.org/cgi-bin/carddisp.pl?gene=MIR7-1 |
| KRT4 | Keratin 4 | Protein Coding | 43 | GC12M052806 | 4.62 | https://www.genecards.org/cgi-bin/carddisp.pl?gene=KRT4 |
| ERG | ETS Transcription Factor ERG | Protein Coding | 45 | GC21M038367 | 4.62 | https://www.genecards.org/cgi-bin/carddisp.pl?gene=ERG |
| TJP2 | Tight Junction Protein 2 | Protein Coding | 45 | GC09P069121 | 4.62 | https://www.genecards.org/cgi-bin/carddisp.pl?gene=TJP2 |
| CLDN5 | Claudin 5 | Protein Coding | 41 | GC22M019523 | 4.62 | https://www.genecards.org/cgi-bin/carddisp.pl?gene=CLDN5 |
| ALOX5 | Arachidonate 5-Lipoxygenase | Protein Coding | 48 | GC10P045374 | 4.61 | https://www.genecards.org/cgi-bin/carddisp.pl?gene=ALOX5 |
| IL15 | Interleukin 15 | Protein Coding | 40 | GC04P141636 | 4.6 | https://www.genecards.org/cgi-bin/carddisp.pl?gene=IL15 |
| ITGA2 | Integrin Subunit Alpha 2 | Protein Coding | 45 | GC05P052989 | 4.59 | https://www.genecards.org/cgi-bin/carddisp.pl?gene=ITGA2 |
| APAF1 | Apoptotic Peptidase Activating Factor 1 | Protein Coding | 47 | GC12P098645 | 4.59 | https://www.genecards.org/cgi-bin/carddisp.pl?gene=APAF1 |
| HLA-DPB1 | Major Histocompatibility Complex, Class II, DP Beta 1 | Protein Coding | 43 | GC06P047346 | 4.57 | https://www.genecards.org/cgi-bin/carddisp.pl?gene=HLA-DPB1 |
| SEMA3B | Semaphorin 3B | Protein Coding | 39 | GC03P050267 | 4.56 | https://www.genecards.org/cgi-bin/carddisp.pl?gene=SEMA3B |
| SERPINB2 | Serpin Family B Member 2 | Protein Coding | 44 | GC18P063871 | 4.56 | https://www.genecards.org/cgi-bin/carddisp.pl?gene=SERPINB2 |
| LTF | Lactotransferrin | Protein Coding | 43 | GC03M046435 | 4.55 | https://www.genecards.org/cgi-bin/carddisp.pl?gene=LTF |
| GJB1 | Gap Junction Protein Beta 1 | Protein Coding | 47 | GC0XP071215 | 4.54 | https://www.genecards.org/cgi-bin/carddisp.pl?gene=GJB1 |
| SNHG12 | Small Nucleolar RNA Host Gene 12 | RNA Gene | 22 | GC01M028578 | 4.54 | https://www.genecards.org/cgi-bin/carddisp.pl?gene=SNHG12 |
| PPIG | Peptidylprolyl Isomerase G | Protein Coding | 41 | GC02P169584 | 4.54 | https://www.genecards.org/cgi-bin/carddisp.pl?gene=PPIG |
| ROBO1 | Roundabout Guidance Receptor 1 | Protein Coding | 44 | GC03M078597 | 4.54 | https://www.genecards.org/cgi-bin/carddisp.pl?gene=ROBO1 |
| CFL1 | Cofilin 1 | Protein Coding | 45 | GC11M065823 | 4.53 | https://www.genecards.org/cgi-bin/carddisp.pl?gene=CFL1 |
| DAB2 | DAB Adaptor Protein 2 | Protein Coding | 43 | GC05M039371 | 4.51 | https://www.genecards.org/cgi-bin/carddisp.pl?gene=DAB2 |
| LASP1 | LIM And SH3 Protein 1 | Protein Coding | 42 | GC17P038869 | 4.5 | https://www.genecards.org/cgi-bin/carddisp.pl?gene=LASP1 |
| CDC25A | Cell Division Cycle 25A | Protein Coding | 47 | GC03M048173 | 4.5 | https://www.genecards.org/cgi-bin/carddisp.pl?gene=CDC25A |
| CYSLTR1 | Cysteinyl Leukotriene Receptor 1 | Protein Coding | 43 | GC0XM078271 | 4.48 | https://www.genecards.org/cgi-bin/carddisp.pl?gene=CYSLTR1 |
| MMP12 | Matrix Metallopeptidase 12 | Protein Coding | 44 | GC11M102862 | 4.48 | https://www.genecards.org/cgi-bin/carddisp.pl?gene=MMP12 |
| SREBF1 | Sterol Regulatory Element Binding Transcription Factor 1 | Protein Coding | 44 | GC17M017810 | 4.48 | https://www.genecards.org/cgi-bin/carddisp.pl?gene=SREBF1 |
| NGFR | Nerve Growth Factor Receptor | Protein Coding | 45 | GC17P049495 | 4.47 | https://www.genecards.org/cgi-bin/carddisp.pl?gene=NGFR |
| TACSTD2 | Tumor Associated Calcium Signal Transducer 2 | Protein Coding | 43 | GC01M058575 | 4.47 | https://www.genecards.org/cgi-bin/carddisp.pl?gene=TACSTD2 |
| AQP1 | Aquaporin 1 (Colton Blood Group) | Protein Coding | 45 | GC07P030911 | 4.46 | https://www.genecards.org/cgi-bin/carddisp.pl?gene=AQP1 |
| AGR2 | Anterior Gradient 2, Protein Disulphide Isomerase Family Member | Protein Coding | 40 | GC07M016832 | 4.46 | https://www.genecards.org/cgi-bin/carddisp.pl?gene=AGR2 |
| MIR346 | MicroRNA 346 | RNA Gene | 18 | GC10M086264 | 4.45 | https://www.genecards.org/cgi-bin/carddisp.pl?gene=MIR346 |
| MIR135B | MicroRNA 135b | RNA Gene | 19 | GC01M205448 | 4.45 | https://www.genecards.org/cgi-bin/carddisp.pl?gene=MIR135B |
| MBD4 | Methyl-CpG Binding Domain 4, DNA Glycosylase | Protein Coding | 42 | GC03M129430 | 4.44 | https://www.genecards.org/cgi-bin/carddisp.pl?gene=MBD4 |
| CCNE2 | Cyclin E2 | Protein Coding | 41 | GC08M094879 | 4.43 | https://www.genecards.org/cgi-bin/carddisp.pl?gene=CCNE2 |
| CXCL10 | C-X-C Motif Chemokine Ligand 10 | Protein Coding | 44 | GC04M076021 | 4.43 | https://www.genecards.org/cgi-bin/carddisp.pl?gene=CXCL10 |
| ATF1 | Activating Transcription Factor 1 | Protein Coding | 46 | GC12P050763 | 4.43 | https://www.genecards.org/cgi-bin/carddisp.pl?gene=ATF1 |
| AGER | Advanced Glycosylation End-Product Specific Receptor | Protein Coding | 44 | GC06M032180 | 4.42 | https://www.genecards.org/cgi-bin/carddisp.pl?gene=AGER |
| PDGFA | Platelet Derived Growth Factor Subunit A | Protein Coding | 44 | GC07M000497 | 4.41 | https://www.genecards.org/cgi-bin/carddisp.pl?gene=PDGFA |
| HOPX | HOP Homeobox | Protein Coding | 37 | GC04M056647 | 4.41 | https://www.genecards.org/cgi-bin/carddisp.pl?gene=HOPX |
| SEPTIN3 | Septin 3 | Protein Coding | 30 | GC22P042121 | 4.41 | https://www.genecards.org/cgi-bin/carddisp.pl?gene=SEPTIN3 |
| RRM1 | Ribonucleotide Reductase Catalytic Subunit M1 | Protein Coding | 47 | GC11P004115 | 4.4 | https://www.genecards.org/cgi-bin/carddisp.pl?gene=RRM1 |
| F2R | Coagulation Factor II Thrombin Receptor | Protein Coding | 46 | GC05P076716 | 4.38 | https://www.genecards.org/cgi-bin/carddisp.pl?gene=F2R |
| PTK6 | Protein Tyrosine Kinase 6 | Protein Coding | 47 | GC20M063528 | 4.38 | https://www.genecards.org/cgi-bin/carddisp.pl?gene=PTK6 |
| KDM1A | Lysine Demethylase 1A | Protein Coding | 47 | GC01P023019 | 4.37 | https://www.genecards.org/cgi-bin/carddisp.pl?gene=KDM1A |
| NOS3 | Nitric Oxide Synthase 3 | Protein Coding | 51 | GC07P150990 | 4.36 | https://www.genecards.org/cgi-bin/carddisp.pl?gene=NOS3 |
| MIR184 | MicroRNA 184 | RNA Gene | 23 | GC15P079209 | 4.36 | https://www.genecards.org/cgi-bin/carddisp.pl?gene=MIR184 |
| STAT5B | Signal Transducer And Activator Of Transcription 5B | Protein Coding | 49 | GC17M042199 | 4.34 | https://www.genecards.org/cgi-bin/carddisp.pl?gene=STAT5B |
| SPHK1 | Sphingosine Kinase 1 | Protein Coding | 47 | GC17P076376 | 4.34 | https://www.genecards.org/cgi-bin/carddisp.pl?gene=SPHK1 |
| S100A8 | S100 Calcium Binding Protein A8 | Protein Coding | 42 | GC01M153391 | 4.33 | https://www.genecards.org/cgi-bin/carddisp.pl?gene=S100A8 |
| FOXC1 | Forkhead Box C1 | Protein Coding | 41 | GC06P001610 | 4.32 | https://www.genecards.org/cgi-bin/carddisp.pl?gene=FOXC1 |
| CD1A | CD1a Molecule | Protein Coding | 41 | GC01P158255 | 4.32 | https://www.genecards.org/cgi-bin/carddisp.pl?gene=CD1A |
| TFRC | Transferrin Receptor | Protein Coding | 48 | GC03M196027 | 4.29 | https://www.genecards.org/cgi-bin/carddisp.pl?gene=TFRC |
| MIR218-1 | MicroRNA 218-1 | RNA Gene | 17 | GC04P020677 | 4.28 | https://www.genecards.org/cgi-bin/carddisp.pl?gene=MIR218-1 |
| WNT2B | Wnt Family Member 2B | Protein Coding | 44 | GC01P112466 | 4.28 | https://www.genecards.org/cgi-bin/carddisp.pl?gene=WNT2B |
| EDNRB | Endothelin Receptor Type B | Protein Coding | 49 | GC13M077895 | 4.28 | https://www.genecards.org/cgi-bin/carddisp.pl?gene=EDNRB |
| HAVCR2 | Hepatitis A Virus Cellular Receptor 2 | Protein Coding | 43 | GC05M157063 | 4.28 | https://www.genecards.org/cgi-bin/carddisp.pl?gene=HAVCR2 |
| LAPTM4B | Lysosomal Protein Transmembrane 4 Beta | Protein Coding | 40 | GC08P097775 | 4.28 | https://www.genecards.org/cgi-bin/carddisp.pl?gene=LAPTM4B |
| MACC1 | MET Transcriptional Regulator MACC1 | Protein Coding | 36 | GC07M020140 | 4.27 | https://www.genecards.org/cgi-bin/carddisp.pl?gene=MACC1 |
| KMT2C | Lysine Methyltransferase 2C | Protein Coding | 41 | GC07M152134 | 4.26 | https://www.genecards.org/cgi-bin/carddisp.pl?gene=KMT2C |
| HP | Haptoglobin | Protein Coding | 44 | GC16P072089 | 4.26 | https://www.genecards.org/cgi-bin/carddisp.pl?gene=HP |
| ANG | Angiogenin | Protein Coding | 45 | GC14P020830 | 4.25 | https://www.genecards.org/cgi-bin/carddisp.pl?gene=ANG |
| EIF2AK3 | Eukaryotic Translation Initiation Factor 2 Alpha Kinase 3 | Protein Coding | 48 | GC02M088637 | 4.25 | https://www.genecards.org/cgi-bin/carddisp.pl?gene=EIF2AK3 |
| CCL20 | C-C Motif Chemokine Ligand 20 | Protein Coding | 43 | GC02P227813 | 4.25 | https://www.genecards.org/cgi-bin/carddisp.pl?gene=CCL20 |
| BRD2 | Bromodomain Containing 2 | Protein Coding | 44 | GC06P047345 | 4.24 | https://www.genecards.org/cgi-bin/carddisp.pl?gene=BRD2 |
| TGFBR3 | Transforming Growth Factor Beta Receptor 3 | Protein Coding | 45 | GC01M091680 | 4.23 | https://www.genecards.org/cgi-bin/carddisp.pl?gene=TGFBR3 |
| BDNF | Brain Derived Neurotrophic Factor | Protein Coding | 47 | GC11M027654 | 4.22 | https://www.genecards.org/cgi-bin/carddisp.pl?gene=BDNF |
| EGLN1 | Egl-9 Family Hypoxia Inducible Factor 1 | Protein Coding | 48 | GC01M231363 | 4.2 | https://www.genecards.org/cgi-bin/carddisp.pl?gene=EGLN1 |
| CUL3 | Cullin 3 | Protein Coding | 46 | GC02M224470 | 4.2 | https://www.genecards.org/cgi-bin/carddisp.pl?gene=CUL3 |
| RAP1A | RAP1A, Member Of RAS Oncogene Family | Protein Coding | 47 | GC01P111542 | 4.19 | https://www.genecards.org/cgi-bin/carddisp.pl?gene=RAP1A |
| USP7 | Ubiquitin Specific Peptidase 7 | Protein Coding | 47 | GC16M008892 | 4.18 | https://www.genecards.org/cgi-bin/carddisp.pl?gene=USP7 |
| DDIT3 | DNA Damage Inducible Transcript 3 | Protein Coding | 45 | GC12M057516 | 4.18 | https://www.genecards.org/cgi-bin/carddisp.pl?gene=DDIT3 |
| CTSL | Cathepsin L | Protein Coding | 46 | GC09P087725 | 4.17 | https://www.genecards.org/cgi-bin/carddisp.pl?gene=CTSL |
| SYK | Spleen Associated Tyrosine Kinase | Protein Coding | 50 | GC09P091171 | 4.16 | https://www.genecards.org/cgi-bin/carddisp.pl?gene=SYK |
| KRT1 | Keratin 1 | Protein Coding | 44 | GC12M052674 | 4.15 | https://www.genecards.org/cgi-bin/carddisp.pl?gene=KRT1 |
| TIMELESS | Timeless Circadian Regulator | Protein Coding | 39 | GC12M056416 | 4.13 | https://www.genecards.org/cgi-bin/carddisp.pl?gene=TIMELESS |
| AMFR | Autocrine Motility Factor Receptor | Protein Coding | 43 | GC16M056361 | 4.12 | https://www.genecards.org/cgi-bin/carddisp.pl?gene=AMFR |
| MIR125B2 | MicroRNA 125b-2 | RNA Gene | 21 | GC21P016590 | 4.12 | https://www.genecards.org/cgi-bin/carddisp.pl?gene=MIR125B2 |
| DRAIC | Downregulated RNA In Cancer, Inhibitor Of Cell Invasion And Migration | RNA Gene | 14 | GC15P072894 | 4.12 | https://www.genecards.org/cgi-bin/carddisp.pl?gene=DRAIC |
| LIF | LIF Interleukin 6 Family Cytokine | Protein Coding | 43 | GC22M030240 | 4.11 | https://www.genecards.org/cgi-bin/carddisp.pl?gene=LIF |
| LDHB | Lactate Dehydrogenase B | Protein Coding | 46 | GC12M021635 | 4.11 | https://www.genecards.org/cgi-bin/carddisp.pl?gene=LDHB |
| IGFBP1 | Insulin Like Growth Factor Binding Protein 1 | Protein Coding | 43 | GC07P046552 | 4.1 | https://www.genecards.org/cgi-bin/carddisp.pl?gene=IGFBP1 |
| CIP2A | Cellular Inhibitor Of PP2A | Protein Coding | 28 | GC03M108545 | 4.08 | https://www.genecards.org/cgi-bin/carddisp.pl?gene=CIP2A |
| S100A9 | S100 Calcium Binding Protein A9 | Protein Coding | 43 | GC01P153357 | 4.07 | https://www.genecards.org/cgi-bin/carddisp.pl?gene=S100A9 |
| CDK5 | Cyclin Dependent Kinase 5 | Protein Coding | 52 | GC07M151053 | 4.07 | https://www.genecards.org/cgi-bin/carddisp.pl?gene=CDK5 |
| ZNRF3 | Zinc And Ring Finger 3 | Protein Coding | 35 | GC22P028803 | 4.04 | https://www.genecards.org/cgi-bin/carddisp.pl?gene=ZNRF3 |
| RUNX2 | RUNX Family Transcription Factor 2 | Protein Coding | 47 | GC06P047549 | 4.04 | https://www.genecards.org/cgi-bin/carddisp.pl?gene=RUNX2 |
| AGO2 | Argonaute RISC Catalytic Component 2 | Protein Coding | 39 | GC08M140522 | 4.03 | https://www.genecards.org/cgi-bin/carddisp.pl?gene=AGO2 |
| XBP1 | X-Box Binding Protein 1 | Protein Coding | 45 | GC22M028794 | 4.03 | https://www.genecards.org/cgi-bin/carddisp.pl?gene=XBP1 |
| BMX | BMX Non-Receptor Tyrosine Kinase | Protein Coding | 44 | GC0XP015392 | 4.02 | https://www.genecards.org/cgi-bin/carddisp.pl?gene=BMX |
| SERPIND1 | Serpin Family D Member 1 | Protein Coding | 45 | GC22P020836 | 4.02 | https://www.genecards.org/cgi-bin/carddisp.pl?gene=SERPIND1 |
| NLRP3 | NLR Family Pyrin Domain Containing 3 | Protein Coding | 47 | GC01P247415 | 4.01 | https://www.genecards.org/cgi-bin/carddisp.pl?gene=NLRP3 |
| FGF8 | Fibroblast Growth Factor 8 | Protein Coding | 47 | GC10M101770 | 4 | https://www.genecards.org/cgi-bin/carddisp.pl?gene=FGF8 |
| MIR299 | MicroRNA 299 | RNA Gene | 16 | GC14P104780 | 3.98 | https://www.genecards.org/cgi-bin/carddisp.pl?gene=MIR299 |
| VCAM1 | Vascular Cell Adhesion Molecule 1 | Protein Coding | 45 | GC01P100719 | 3.98 | https://www.genecards.org/cgi-bin/carddisp.pl?gene=VCAM1 |
| DSG3 | Desmoglein 3 | Protein Coding | 38 | GC18P031447 | 3.98 | https://www.genecards.org/cgi-bin/carddisp.pl?gene=DSG3 |
| CCNA1 | Cyclin A1 | Protein Coding | 42 | GC13P036431 | 3.98 | https://www.genecards.org/cgi-bin/carddisp.pl?gene=CCNA1 |
| MPO | Myeloperoxidase | Protein Coding | 50 | GC17M058269 | 3.98 | https://www.genecards.org/cgi-bin/carddisp.pl?gene=MPO |
| MIR124-2 | MicroRNA 124-2 | RNA Gene | 18 | GC08P064379 | 3.97 | https://www.genecards.org/cgi-bin/carddisp.pl?gene=MIR124-2 |
| POLB | DNA Polymerase Beta | Protein Coding | 47 | GC08P042338 | 3.97 | https://www.genecards.org/cgi-bin/carddisp.pl?gene=POLB |
| HDAC2 | Histone Deacetylase 2 | Protein Coding | 51 | GC06M113933 | 3.97 | https://www.genecards.org/cgi-bin/carddisp.pl?gene=HDAC2 |
| INTS6 | Integrator Complex Subunit 6 | Protein Coding | 37 | GC13M051354 | 3.95 | https://www.genecards.org/cgi-bin/carddisp.pl?gene=INTS6 |
| CHRNA5 | Cholinergic Receptor Nicotinic Alpha 5 Subunit | Protein Coding | 44 | GC15P078565 | 3.94 | https://www.genecards.org/cgi-bin/carddisp.pl?gene=CHRNA5 |
| ACTA2 | Actin Alpha 2, Smooth Muscle | Protein Coding | 48 | GC10M088935 | 3.93 | https://www.genecards.org/cgi-bin/carddisp.pl?gene=ACTA2 |
| RBBP8 | RB Binding Protein 8, Endonuclease | Protein Coding | 44 | GC18P022798 | 3.93 | https://www.genecards.org/cgi-bin/carddisp.pl?gene=RBBP8 |
| HSPD1 | Heat Shock Protein Family D (Hsp60) Member 1 | Protein Coding | 47 | GC02M197486 | 3.91 | https://www.genecards.org/cgi-bin/carddisp.pl?gene=HSPD1 |
| CASP2 | Caspase 2 | Protein Coding | 49 | GC07P144979 | 3.91 | https://www.genecards.org/cgi-bin/carddisp.pl?gene=CASP2 |
| ADAM12 | ADAM Metallopeptidase Domain 12 | Protein Coding | 45 | GC10M126012 | 3.9 | https://www.genecards.org/cgi-bin/carddisp.pl?gene=ADAM12 |
| STC1 | Stanniocalcin 1 | Protein Coding | 40 | GC08M023841 | 3.89 | https://www.genecards.org/cgi-bin/carddisp.pl?gene=STC1 |
| APOA1 | Apolipoprotein A1 | Protein Coding | 48 | GC11M116835 | 3.89 | https://www.genecards.org/cgi-bin/carddisp.pl?gene=APOA1 |
| ASS1 | Argininosuccinate Synthase 1 | Protein Coding | 48 | GC09P130444 | 3.88 | https://www.genecards.org/cgi-bin/carddisp.pl?gene=ASS1 |
| F2RL1 | F2R Like Trypsin Receptor 1 | Protein Coding | 45 | GC05P076818 | 3.87 | https://www.genecards.org/cgi-bin/carddisp.pl?gene=F2RL1 |
| HES1 | Hes Family BHLH Transcription Factor 1 | Protein Coding | 43 | GC03P194136 | 3.87 | https://www.genecards.org/cgi-bin/carddisp.pl?gene=HES1 |
| PHF20 | PHD Finger Protein 20 | Protein Coding | 39 | GC20P035771 | 3.86 | https://www.genecards.org/cgi-bin/carddisp.pl?gene=PHF20 |
| SELE | Selectin E | Protein Coding | 44 | GC01M169722 | 3.85 | https://www.genecards.org/cgi-bin/carddisp.pl?gene=SELE |
| CFAP45 | Cilia And Flagella Associated Protein 45 | Protein Coding | 29 | GC01M159873 | 3.85 | https://www.genecards.org/cgi-bin/carddisp.pl?gene=CFAP45 |
| ECRG4 | ECRG4 Augurin Precursor | Protein Coding | 27 | GC02P106063 | 3.84 | https://www.genecards.org/cgi-bin/carddisp.pl?gene=ECRG4 |
| MMP19 | Matrix Metallopeptidase 19 | Protein Coding | 47 | GC12M055835 | 3.84 | https://www.genecards.org/cgi-bin/carddisp.pl?gene=MMP19 |
| IL12A | Interleukin 12A | Protein Coding | 44 | GC03P159988 | 3.83 | https://www.genecards.org/cgi-bin/carddisp.pl?gene=IL12A |
| CCL3 | C-C Motif Chemokine Ligand 3 | Protein Coding | 39 | GC17M036088 | 3.83 | https://www.genecards.org/cgi-bin/carddisp.pl?gene=CCL3 |
| TP53COR1 | Tumor Protein P53 Pathway Corepressor 1 | RNA Gene | 8 | GC06U903133 | 3.83 | https://www.genecards.org/cgi-bin/carddisp.pl?gene=TP53COR1 |
| CHFR | Checkpoint With Forkhead And Ring Finger Domains | Protein Coding | 40 | GC12M132822 | 3.82 | https://www.genecards.org/cgi-bin/carddisp.pl?gene=CHFR |
| SLCO1B3 | Solute Carrier Organic Anion Transporter Family Member 1B3 | Protein Coding | 44 | GC12P020810 | 3.82 | https://www.genecards.org/cgi-bin/carddisp.pl?gene=SLCO1B3 |
| RRM2B | Ribonucleotide Reductase Regulatory TP53 Inducible Subunit M2B | Protein Coding | 49 | GC08M102204 | 3.81 | https://www.genecards.org/cgi-bin/carddisp.pl?gene=RRM2B |
| ACE | Angiotensin I Converting Enzyme | Protein Coding | 49 | GC17P063477 | 3.81 | https://www.genecards.org/cgi-bin/carddisp.pl?gene=ACE |
| MLANA | Melan-A | Protein Coding | 39 | GC09P005846 | 3.81 | https://www.genecards.org/cgi-bin/carddisp.pl?gene=MLANA |
| CRYAB | Crystallin Alpha B | Protein Coding | 45 | GC11M111908 | 3.79 | https://www.genecards.org/cgi-bin/carddisp.pl?gene=CRYAB |
| DLL4 | Delta Like Canonical Notch Ligand 4 | Protein Coding | 45 | GC15P040929 | 3.79 | https://www.genecards.org/cgi-bin/carddisp.pl?gene=DLL4 |
| AFAP1 | Actin Filament Associated Protein 1 | Protein Coding | 39 | GC04M007758 | 3.78 | https://www.genecards.org/cgi-bin/carddisp.pl?gene=AFAP1 |
| SFRP2 | Secreted Frizzled Related Protein 2 | Protein Coding | 42 | GC04M153780 | 3.78 | https://www.genecards.org/cgi-bin/carddisp.pl?gene=SFRP2 |
| LGALS3BP | Galectin 3 Binding Protein | Protein Coding | 41 | GC17M078971 | 3.77 | https://www.genecards.org/cgi-bin/carddisp.pl?gene=LGALS3BP |
| CDC6 | Cell Division Cycle 6 | Protein Coding | 45 | GC17P040287 | 3.74 | https://www.genecards.org/cgi-bin/carddisp.pl?gene=CDC6 |
| CXCL1 | C-X-C Motif Chemokine Ligand 1 | Protein Coding | 43 | GC04P073869 | 3.74 | https://www.genecards.org/cgi-bin/carddisp.pl?gene=CXCL1 |
| GADD45G | Growth Arrest And DNA Damage Inducible Gamma | Protein Coding | 40 | GC09P089605 | 3.73 | https://www.genecards.org/cgi-bin/carddisp.pl?gene=GADD45G |
| CCL4 | C-C Motif Chemokine Ligand 4 | Protein Coding | 40 | GC17P036103 | 3.73 | https://www.genecards.org/cgi-bin/carddisp.pl?gene=CCL4 |
| EDN2 | Endothelin 2 | Protein Coding | 40 | GC01M041478 | 3.73 | https://www.genecards.org/cgi-bin/carddisp.pl?gene=EDN2 |
| RXRB | Retinoid X Receptor Beta | Protein Coding | 47 | GC06M033193 | 3.73 | https://www.genecards.org/cgi-bin/carddisp.pl?gene=RXRB |
| KAT2B | Lysine Acetyltransferase 2B | Protein Coding | 48 | GC03P020081 | 3.72 | https://www.genecards.org/cgi-bin/carddisp.pl?gene=KAT2B |
| CREB5 | CAMP Responsive Element Binding Protein 5 | Protein Coding | 41 | GC07P028305 | 3.72 | https://www.genecards.org/cgi-bin/carddisp.pl?gene=CREB5 |
| FBP1 | Fructose-Bisphosphatase 1 | Protein Coding | 50 | GC09M094603 | 3.71 | https://www.genecards.org/cgi-bin/carddisp.pl?gene=FBP1 |
| SND1-IT1 | SND1 Intronic Transcript 1 | RNA Gene | 16 | GC07P127997 | 3.71 | https://www.genecards.org/cgi-bin/carddisp.pl?gene=SND1-IT1 |
| NR4A3 | Nuclear Receptor Subfamily 4 Group A Member 3 | Protein Coding | 45 | GC09P099821 | 3.69 | https://www.genecards.org/cgi-bin/carddisp.pl?gene=NR4A3 |
| LGALS9 | Galectin 9 | Protein Coding | 39 | GC17P027629 | 3.69 | https://www.genecards.org/cgi-bin/carddisp.pl?gene=LGALS9 |
| FLOT2 | Flotillin 2 | Protein Coding | 41 | GC17M029963 | 3.68 | https://www.genecards.org/cgi-bin/carddisp.pl?gene=FLOT2 |
| MIR572 | MicroRNA 572 | RNA Gene | 14 | GC04P013477 | 3.66 | https://www.genecards.org/cgi-bin/carddisp.pl?gene=MIR572 |
| ATF6 | Activating Transcription Factor 6 | Protein Coding | 47 | GC01P161766 | 3.66 | https://www.genecards.org/cgi-bin/carddisp.pl?gene=ATF6 |
| MEFV | MEFV Innate Immuity Regulator, Pyrin | Protein Coding | 43 | GC16M003281 | 3.66 | https://www.genecards.org/cgi-bin/carddisp.pl?gene=MEFV |
| TFPI2 | Tissue Factor Pathway Inhibitor 2 | Protein Coding | 41 | GC07M093885 | 3.66 | https://www.genecards.org/cgi-bin/carddisp.pl?gene=TFPI2 |
| SLPI | Secretory Leukocyte Peptidase Inhibitor | Protein Coding | 39 | GC20M045252 | 3.65 | https://www.genecards.org/cgi-bin/carddisp.pl?gene=SLPI |
| F3 | Coagulation Factor III, Tissue Factor | Protein Coding | 45 | GC01M094530 | 3.65 | https://www.genecards.org/cgi-bin/carddisp.pl?gene=F3 |
| MIR1-2 | MicroRNA 1-2 | RNA Gene | 17 | GC18M021828 | 3.65 | https://www.genecards.org/cgi-bin/carddisp.pl?gene=MIR1-2 |
| MUC5B | Mucin 5B, Oligomeric Mucus/Gel-Forming | Protein Coding | 41 | GC11P001244 | 3.65 | https://www.genecards.org/cgi-bin/carddisp.pl?gene=MUC5B |
| MIR495 | MicroRNA 495 | RNA Gene | 16 | GC14P104815 | 3.64 | https://www.genecards.org/cgi-bin/carddisp.pl?gene=MIR495 |
| NFKB2 | Nuclear Factor Kappa B Subunit 2 | Protein Coding | 52 | GC10P102394 | 3.62 | https://www.genecards.org/cgi-bin/carddisp.pl?gene=NFKB2 |
| RAD51B | RAD51 Paralog B | Protein Coding | 37 | GC14P067819 | 3.61 | https://www.genecards.org/cgi-bin/carddisp.pl?gene=RAD51B |
| HSP90AB1 | Heat Shock Protein 90 Alpha Family Class B Member 1 | Protein Coding | 45 | GC06P044246 | 3.61 | https://www.genecards.org/cgi-bin/carddisp.pl?gene=HSP90AB1 |
| BBC3 | BCL2 Binding Component 3 | Protein Coding | 41 | GC19M047220 | 3.61 | https://www.genecards.org/cgi-bin/carddisp.pl?gene=BBC3 |
| SPAG9 | Sperm Associated Antigen 9 | Protein Coding | 40 | GC17M050962 | 3.6 | https://www.genecards.org/cgi-bin/carddisp.pl?gene=SPAG9 |
| BRD3 | Bromodomain Containing 3 | Protein Coding | 41 | GC09M134030 | 3.59 | https://www.genecards.org/cgi-bin/carddisp.pl?gene=BRD3 |
| RPA1 | Replication Protein A1 | Protein Coding | 45 | GC17P001829 | 3.59 | https://www.genecards.org/cgi-bin/carddisp.pl?gene=RPA1 |
| CLCN3 | Chloride Voltage-Gated Channel 3 | Protein Coding | 41 | GC04P169612 | 3.58 | https://www.genecards.org/cgi-bin/carddisp.pl?gene=CLCN3 |
| SAA1 | Serum Amyloid A1 | Protein Coding | 42 | GC11P018267 | 3.58 | https://www.genecards.org/cgi-bin/carddisp.pl?gene=SAA1 |
| CD68 | CD68 Molecule | Protein Coding | 40 | GC17P007579 | 3.57 | https://www.genecards.org/cgi-bin/carddisp.pl?gene=CD68 |
| DAB2IP | DAB2 Interacting Protein | Protein Coding | 39 | GC09P121566 | 3.57 | https://www.genecards.org/cgi-bin/carddisp.pl?gene=DAB2IP |
| AQP3 | Aquaporin 3 (Gill Blood Group) | Protein Coding | 47 | GC09M033431 | 3.57 | https://www.genecards.org/cgi-bin/carddisp.pl?gene=AQP3 |
| EMP2 | Epithelial Membrane Protein 2 | Protein Coding | 42 | GC16M010541 | 3.56 | https://www.genecards.org/cgi-bin/carddisp.pl?gene=EMP2 |
| REG1A | Regenerating Family Member 1 Alpha | Protein Coding | 41 | GC02P079120 | 3.56 | https://www.genecards.org/cgi-bin/carddisp.pl?gene=REG1A |
| RBL2 | RB Transcriptional Corepressor Like 2 | Protein Coding | 43 | GC16P053433 | 3.54 | https://www.genecards.org/cgi-bin/carddisp.pl?gene=RBL2 |
| CXADR | CXADR Ig-Like Cell Adhesion Molecule | Protein Coding | 43 | GC21P017512 | 3.54 | https://www.genecards.org/cgi-bin/carddisp.pl?gene=CXADR |
| PIM1 | Pim-1 Proto-Oncogene, Serine/Threonine Kinase | Protein Coding | 50 | GC06P047469 | 3.53 | https://www.genecards.org/cgi-bin/carddisp.pl?gene=PIM1 |
| PTP4A1 | Protein Tyrosine Phosphatase 4A1 | Protein Coding | 41 | GC06P063521 | 3.53 | https://www.genecards.org/cgi-bin/carddisp.pl?gene=PTP4A1 |
| PMEL | Premelanosome Protein | Protein Coding | 37 | GC12M055954 | 3.53 | https://www.genecards.org/cgi-bin/carddisp.pl?gene=PMEL |
| PDK1 | Pyruvate Dehydrogenase Kinase 1 | Protein Coding | 47 | GC02P172555 | 3.52 | https://www.genecards.org/cgi-bin/carddisp.pl?gene=PDK1 |
| PTH | Parathyroid Hormone | Protein Coding | 47 | GC11M013492 | 3.52 | https://www.genecards.org/cgi-bin/carddisp.pl?gene=PTH |
| CD70 | CD70 Molecule | Protein Coding | 43 | GC19M006583 | 3.5 | https://www.genecards.org/cgi-bin/carddisp.pl?gene=CD70 |
| TNFRSF19 | TNF Receptor Superfamily Member 19 | Protein Coding | 39 | GC13P023570 | 3.5 | https://www.genecards.org/cgi-bin/carddisp.pl?gene=TNFRSF19 |
| TGIF1 | TGFB Induced Factor Homeobox 1 | Protein Coding | 45 | GC18P003411 | 3.49 | https://www.genecards.org/cgi-bin/carddisp.pl?gene=TGIF1 |
| IL3 | Interleukin 3 | Protein Coding | 44 | GC05P132060 | 3.49 | https://www.genecards.org/cgi-bin/carddisp.pl?gene=IL3 |
| EIF2AK2 | Eukaryotic Translation Initiation Factor 2 Alpha Kinase 2 | Protein Coding | 45 | GC02M037099 | 3.49 | https://www.genecards.org/cgi-bin/carddisp.pl?gene=EIF2AK2 |
| MIR218-2 | MicroRNA 218-2 | RNA Gene | 18 | GC05M168768 | 3.49 | https://www.genecards.org/cgi-bin/carddisp.pl?gene=MIR218-2 |
| LIFR | LIF Receptor Subunit Alpha | Protein Coding | 47 | GC05M038475 | 3.48 | https://www.genecards.org/cgi-bin/carddisp.pl?gene=LIFR |
| SLIT2 | Slit Guidance Ligand 2 | Protein Coding | 45 | GC04P020287 | 3.47 | https://www.genecards.org/cgi-bin/carddisp.pl?gene=SLIT2 |
| MCAM | Melanoma Cell Adhesion Molecule | Protein Coding | 40 | GC11M119308 | 3.47 | https://www.genecards.org/cgi-bin/carddisp.pl?gene=MCAM |
| CCL27 | C-C Motif Chemokine Ligand 27 | Protein Coding | 37 | GC09M034662 | 3.46 | https://www.genecards.org/cgi-bin/carddisp.pl?gene=CCL27 |
| PAWR | Pro-Apoptotic WT1 Regulator | Protein Coding | 40 | GC12M079574 | 3.46 | https://www.genecards.org/cgi-bin/carddisp.pl?gene=PAWR |
| AGK | Acylglycerol Kinase | Protein Coding | 41 | GC07P141551 | 3.46 | https://www.genecards.org/cgi-bin/carddisp.pl?gene=AGK |
| PARK7 | Parkinsonism Associated Deglycase | Protein Coding | 45 | GC01P007983 | 3.45 | https://www.genecards.org/cgi-bin/carddisp.pl?gene=PARK7 |
| TRPM7 | Transient Receptor Potential Cation Channel Subfamily M Member 7 | Protein Coding | 45 | GC15M050552 | 3.45 | https://www.genecards.org/cgi-bin/carddisp.pl?gene=TRPM7 |
| CYP3A5 | Cytochrome P450 Family 3 Subfamily A Member 5 | Protein Coding | 47 | GC07M099648 | 3.44 | https://www.genecards.org/cgi-bin/carddisp.pl?gene=CYP3A5 |
| ACKR3 | Atypical Chemokine Receptor 3 | Protein Coding | 40 | GC02P236537 | 3.44 | https://www.genecards.org/cgi-bin/carddisp.pl?gene=ACKR3 |
| CP | Ceruloplasmin | Protein Coding | 47 | GC03M149162 | 3.42 | https://www.genecards.org/cgi-bin/carddisp.pl?gene=CP |
| NGF | Nerve Growth Factor | Protein Coding | 50 | GC01M115285 | 3.42 | https://www.genecards.org/cgi-bin/carddisp.pl?gene=NGF |
| NEDD4 | NEDD4 E3 Ubiquitin Protein Ligase | Protein Coding | 45 | GC15M055826 | 3.42 | https://www.genecards.org/cgi-bin/carddisp.pl?gene=NEDD4 |
| HIF1AN | Hypoxia Inducible Factor 1 Subunit Alpha Inhibitor | Protein Coding | 44 | GC10P100529 | 3.41 | https://www.genecards.org/cgi-bin/carddisp.pl?gene=HIF1AN |
| LINC00210 | Long Intergenic Non-Protein Coding RNA 210 | RNA Gene | 13 | GC01P217892 | 3.41 | https://www.genecards.org/cgi-bin/carddisp.pl?gene=LINC00210 |
| IQGAP1 | IQ Motif Containing GTPase Activating Protein 1 | Protein Coding | 43 | GC15P090388 | 3.4 | https://www.genecards.org/cgi-bin/carddisp.pl?gene=IQGAP1 |
| ZBTB7A | Zinc Finger And BTB Domain Containing 7A | Protein Coding | 36 | GC19M004045 | 3.4 | https://www.genecards.org/cgi-bin/carddisp.pl?gene=ZBTB7A |
| LIG3 | DNA Ligase 3 | Protein Coding | 44 | GC17P034980 | 3.39 | https://www.genecards.org/cgi-bin/carddisp.pl?gene=LIG3 |
| MYH9 | Myosin Heavy Chain 9 | Protein Coding | 49 | GC22M036281 | 3.39 | https://www.genecards.org/cgi-bin/carddisp.pl?gene=MYH9 |
| SCGB1A1 | Secretoglobin Family 1A Member 1 | Protein Coding | 40 | GC11P062405 | 3.39 | https://www.genecards.org/cgi-bin/carddisp.pl?gene=SCGB1A1 |
| GPX2 | Glutathione Peroxidase 2 | Protein Coding | 44 | GC14M064939 | 3.39 | https://www.genecards.org/cgi-bin/carddisp.pl?gene=GPX2 |
| CD19 | CD19 Molecule | Protein Coding | 49 | GC16P029083 | 3.38 | https://www.genecards.org/cgi-bin/carddisp.pl?gene=CD19 |
| TNFRSF11B | TNF Receptor Superfamily Member 11b | Protein Coding | 47 | GC08M118923 | 3.38 | https://www.genecards.org/cgi-bin/carddisp.pl?gene=TNFRSF11B |
| AIFM1 | Apoptosis Inducing Factor Mitochondria Associated 1 | Protein Coding | 49 | GC0XM130129 | 3.38 | https://www.genecards.org/cgi-bin/carddisp.pl?gene=AIFM1 |
| EIF3A | Eukaryotic Translation Initiation Factor 3 Subunit A | Protein Coding | 41 | GC10M119034 | 3.37 | https://www.genecards.org/cgi-bin/carddisp.pl?gene=EIF3A |
| RARRES1 | Retinoic Acid Receptor Responder 1 | Protein Coding | 40 | GC03M158696 | 3.37 | https://www.genecards.org/cgi-bin/carddisp.pl?gene=RARRES1 |
| HCP5 | HLA Complex P5 | RNA Gene | 29 | GC06P031400 | 3.37 | https://www.genecards.org/cgi-bin/carddisp.pl?gene=HCP5 |
| ABCC4 | ATP Binding Cassette Subfamily C Member 4 | Protein Coding | 45 | GC13M095019 | 3.37 | https://www.genecards.org/cgi-bin/carddisp.pl?gene=ABCC4 |
| ESS2 | Ess-2 Splicing Factor Homolog | Protein Coding | 28 | GC22M019130 | 3.36 | https://www.genecards.org/cgi-bin/carddisp.pl?gene=ESS2 |
| ANXA3 | Annexin A3 | Protein Coding | 42 | GC04P078551 | 3.35 | https://www.genecards.org/cgi-bin/carddisp.pl?gene=ANXA3 |
| ZFP57 | ZFP57 Zinc Finger Protein | Protein Coding | 39 | GC06M029672 | 3.35 | https://www.genecards.org/cgi-bin/carddisp.pl?gene=ZFP57 |
| ACP1 | Acid Phosphatase 1 | Protein Coding | 44 | GC02P000254 | 3.33 | https://www.genecards.org/cgi-bin/carddisp.pl?gene=ACP1 |
| CD9 | CD9 Molecule | Protein Coding | 44 | GC12P008124 | 3.33 | https://www.genecards.org/cgi-bin/carddisp.pl?gene=CD9 |
| MIR506 | MicroRNA 506 | RNA Gene | 15 | GC0XM147230 | 3.33 | https://www.genecards.org/cgi-bin/carddisp.pl?gene=MIR506 |
| ADM | Adrenomedullin | Protein Coding | 44 | GC11P010304 | 3.32 | https://www.genecards.org/cgi-bin/carddisp.pl?gene=ADM |
| MIR103A2 | MicroRNA 103a-2 | RNA Gene | 18 | GC20P003917 | 3.32 | https://www.genecards.org/cgi-bin/carddisp.pl?gene=MIR103A2 |
| SLC5A5 | Solute Carrier Family 5 Member 5 | Protein Coding | 45 | GC19P023315 | 3.31 | https://www.genecards.org/cgi-bin/carddisp.pl?gene=SLC5A5 |
| ARHGAP5 | Rho GTPase Activating Protein 5 | Protein Coding | 43 | GC14P032076 | 3.3 | https://www.genecards.org/cgi-bin/carddisp.pl?gene=ARHGAP5 |
| ROR2 | Receptor Tyrosine Kinase Like Orphan Receptor 2 | Protein Coding | 48 | GC09M091564 | 3.3 | https://www.genecards.org/cgi-bin/carddisp.pl?gene=ROR2 |
| DUSP6 | Dual Specificity Phosphatase 6 | Protein Coding | 48 | GC12M089347 | 3.29 | https://www.genecards.org/cgi-bin/carddisp.pl?gene=DUSP6 |
| IL6ST | Interleukin 6 Signal Transducer | Protein Coding | 45 | GC05M055935 | 3.29 | https://www.genecards.org/cgi-bin/carddisp.pl?gene=IL6ST |
| ADAM17 | ADAM Metallopeptidase Domain 17 | Protein Coding | 51 | GC02M009488 | 3.28 | https://www.genecards.org/cgi-bin/carddisp.pl?gene=ADAM17 |
| HLA-DPA1 | Major Histocompatibility Complex, Class II, DP Alpha 1 | Protein Coding | 40 | GC06M033064 | 3.28 | https://www.genecards.org/cgi-bin/carddisp.pl?gene=HLA-DPA1 |
| RBP1 | Retinol Binding Protein 1 | Protein Coding | 39 | GC03M139517 | 3.28 | https://www.genecards.org/cgi-bin/carddisp.pl?gene=RBP1 |
| RELB | RELB Proto-Oncogene, NF-KB Subunit | Protein Coding | 45 | GC19P045002 | 3.28 | https://www.genecards.org/cgi-bin/carddisp.pl?gene=RELB |
| LIG4 | DNA Ligase 4 | Protein Coding | 48 | GC13M108207 | 3.26 | https://www.genecards.org/cgi-bin/carddisp.pl?gene=LIG4 |
| PTPN6 | Protein Tyrosine Phosphatase Non-Receptor Type 6 | Protein Coding | 50 | GC12P008223 | 3.25 | https://www.genecards.org/cgi-bin/carddisp.pl?gene=PTPN6 |
| HLA-F | Major Histocompatibility Complex, Class I, F | Protein Coding | 40 | GC06P047251 | 3.24 | https://www.genecards.org/cgi-bin/carddisp.pl?gene=HLA-F |
| MIR328 | MicroRNA 328 | RNA Gene | 18 | GC16M067203 | 3.23 | https://www.genecards.org/cgi-bin/carddisp.pl?gene=MIR328 |
| HSPA1B | Heat Shock Protein Family A (Hsp70) Member 1B | Protein Coding | 40 | GC06P047325 | 3.22 | https://www.genecards.org/cgi-bin/carddisp.pl?gene=HSPA1B |
| CXCL5 | C-X-C Motif Chemokine Ligand 5 | Protein Coding | 40 | GC04M073995 | 3.22 | https://www.genecards.org/cgi-bin/carddisp.pl?gene=CXCL5 |
| TRAF2 | TNF Receptor Associated Factor 2 | Protein Coding | 44 | GC09P136881 | 3.21 | https://www.genecards.org/cgi-bin/carddisp.pl?gene=TRAF2 |
| CEBPB | CCAAT Enhancer Binding Protein Beta | Protein Coding | 43 | GC20P050190 | 3.21 | https://www.genecards.org/cgi-bin/carddisp.pl?gene=CEBPB |
| COL1A1 | Collagen Type I Alpha 1 Chain | Protein Coding | 50 | GC17M050183 | 3.2 | https://www.genecards.org/cgi-bin/carddisp.pl?gene=COL1A1 |
| CDC27 | Cell Division Cycle 27 | Protein Coding | 41 | GC17M047117 | 3.19 | https://www.genecards.org/cgi-bin/carddisp.pl?gene=CDC27 |
| PANTR1 | POU3F3 Adjacent Non-Coding Transcript 1 | RNA Gene | 13 | GC02M104806 | 3.18 | https://www.genecards.org/cgi-bin/carddisp.pl?gene=PANTR1 |
| CALM1 | Calmodulin 1 | Protein Coding | 45 | GC14P090396 | 3.18 | https://www.genecards.org/cgi-bin/carddisp.pl?gene=CALM1 |
| LACTB | Lactamase Beta | Protein Coding | 36 | GC15P073008 | 3.17 | https://www.genecards.org/cgi-bin/carddisp.pl?gene=LACTB |
| TKTL1 | Transketolase Like 1 | Protein Coding | 41 | GC0XP154295 | 3.17 | https://www.genecards.org/cgi-bin/carddisp.pl?gene=TKTL1 |
| SLC7A4 | Solute Carrier Family 7 Member 4 | Protein Coding | 37 | GC22M021028 | 3.17 | https://www.genecards.org/cgi-bin/carddisp.pl?gene=SLC7A4 |
| MIR630 | MicroRNA 630 | RNA Gene | 13 | GC15P072587 | 3.17 | https://www.genecards.org/cgi-bin/carddisp.pl?gene=MIR630 |
| MAP1LC3A | Microtubule Associated Protein 1 Light Chain 3 Alpha | Protein Coding | 43 | GC20P034546 | 3.16 | https://www.genecards.org/cgi-bin/carddisp.pl?gene=MAP1LC3A |
| CD63 | CD63 Molecule | Protein Coding | 41 | GC12M055725 | 3.16 | https://www.genecards.org/cgi-bin/carddisp.pl?gene=CD63 |
| FOXO4 | Forkhead Box O4 | Protein Coding | 43 | GC0XP071095 | 3.15 | https://www.genecards.org/cgi-bin/carddisp.pl?gene=FOXO4 |
| PRDX2 | Peroxiredoxin 2 | Protein Coding | 46 | GC19M012796 | 3.15 | https://www.genecards.org/cgi-bin/carddisp.pl?gene=PRDX2 |
| HBB | Hemoglobin Subunit Beta | Protein Coding | 45 | GC11M005352 | 3.15 | https://www.genecards.org/cgi-bin/carddisp.pl?gene=HBB |
| NCOA2 | Nuclear Receptor Coactivator 2 | Protein Coding | 44 | GC08M070109 | 3.15 | https://www.genecards.org/cgi-bin/carddisp.pl?gene=NCOA2 |
| PLAT | Plasminogen Activator, Tissue Type | Protein Coding | 49 | GC08M042174 | 3.14 | https://www.genecards.org/cgi-bin/carddisp.pl?gene=PLAT |
| GSTA1 | Glutathione S-Transferase Alpha 1 | Protein Coding | 41 | GC06M052791 | 3.13 | https://www.genecards.org/cgi-bin/carddisp.pl?gene=GSTA1 |
| TOX3 | TOX High Mobility Group Box Family Member 3 | Protein Coding | 37 | GC16M052471 | 3.13 | https://www.genecards.org/cgi-bin/carddisp.pl?gene=TOX3 |
| HAGLROS | HAGLR Opposite Strand LncRNA | RNA Gene | 14 | GC02P176177 | 3.13 | https://www.genecards.org/cgi-bin/carddisp.pl?gene=HAGLROS |
| NME2 | NME/NM23 Nucleoside Diphosphate Kinase 2 | Protein Coding | 46 | GC17P051165 | 3.12 | https://www.genecards.org/cgi-bin/carddisp.pl?gene=NME2 |
| TNFAIP3 | TNF Alpha Induced Protein 3 | Protein Coding | 48 | GC06P137866 | 3.12 | https://www.genecards.org/cgi-bin/carddisp.pl?gene=TNFAIP3 |
| AIMP2 | Aminoacyl TRNA Synthetase Complex Interacting Multifunctional Protein 2 | Protein Coding | 41 | GC07P006016 | 3.11 | https://www.genecards.org/cgi-bin/carddisp.pl?gene=AIMP2 |
| TAPBP | TAP Binding Protein | Protein Coding | 43 | GC06M033299 | 3.11 | https://www.genecards.org/cgi-bin/carddisp.pl?gene=TAPBP |
| APP | Amyloid Beta Precursor Protein | Protein Coding | 51 | GC21M025880 | 3.1 | https://www.genecards.org/cgi-bin/carddisp.pl?gene=APP |
| TNFRSF9 | TNF Receptor Superfamily Member 9 | Protein Coding | 44 | GC01M007915 | 3.09 | https://www.genecards.org/cgi-bin/carddisp.pl?gene=TNFRSF9 |
| ID2 | Inhibitor Of DNA Binding 2 | Protein Coding | 44 | GC02P008772 | 3.09 | https://www.genecards.org/cgi-bin/carddisp.pl?gene=ID2 |
| CDH4 | Cadherin 4 | Protein Coding | 41 | GC20P061252 | 3.08 | https://www.genecards.org/cgi-bin/carddisp.pl?gene=CDH4 |
| TPI1 | Triosephosphate Isomerase 1 | Protein Coding | 48 | GC12P008208 | 3.08 | https://www.genecards.org/cgi-bin/carddisp.pl?gene=TPI1 |
| TRAF1 | TNF Receptor Associated Factor 1 | Protein Coding | 43 | GC09M120902 | 3.05 | https://www.genecards.org/cgi-bin/carddisp.pl?gene=TRAF1 |
| KANK1 | KN Motif And Ankyrin Repeat Domains 1 | Protein Coding | 41 | GC09P000474 | 3.04 | https://www.genecards.org/cgi-bin/carddisp.pl?gene=KANK1 |
| FAM215A | Family With Sequence Similarity 215 Member A | RNA Gene | 22 | GC17P043917 | 3.04 | https://www.genecards.org/cgi-bin/carddisp.pl?gene=FAM215A |
| IL16 | Interleukin 16 | Protein Coding | 42 | GC15P081159 | 3.02 | https://www.genecards.org/cgi-bin/carddisp.pl?gene=IL16 |
| ESM1 | Endothelial Cell Specific Molecule 1 | Protein Coding | 40 | GC05M054977 | 3.02 | https://www.genecards.org/cgi-bin/carddisp.pl?gene=ESM1 |
| EMP1 | Epithelial Membrane Protein 1 | Protein Coding | 35 | GC12P013196 | 3.01 | https://www.genecards.org/cgi-bin/carddisp.pl?gene=EMP1 |
| TBX3 | T-Box Transcription Factor 3 | Protein Coding | 45 | GC12M114670 | 3 | https://www.genecards.org/cgi-bin/carddisp.pl?gene=TBX3 |
| FOXQ1 | Forkhead Box Q1 | Protein Coding | 33 | GC06P001312 | 3 | https://www.genecards.org/cgi-bin/carddisp.pl?gene=FOXQ1 |
| ITGA9 | Integrin Subunit Alpha 9 | Protein Coding | 43 | GC03P037468 | 3 | https://www.genecards.org/cgi-bin/carddisp.pl?gene=ITGA9 |
| MAP3K5 | Mitogen-Activated Protein Kinase Kinase Kinase 5 | Protein Coding | 47 | GC06M136557 | 3 | https://www.genecards.org/cgi-bin/carddisp.pl?gene=MAP3K5 |
| CCL11 | C-C Motif Chemokine Ligand 11 | Protein Coding | 43 | GC17P034285 | 3 | https://www.genecards.org/cgi-bin/carddisp.pl?gene=CCL11 |
| MIR663A | MicroRNA 663a | RNA Gene | 15 | GC20M026189 | 2.99 | https://www.genecards.org/cgi-bin/carddisp.pl?gene=MIR663A |
| TNFRSF1B | TNF Receptor Superfamily Member 1B | Protein Coding | 47 | GC01P012167 | 2.98 | https://www.genecards.org/cgi-bin/carddisp.pl?gene=TNFRSF1B |
| TACC3 | Transforming Acidic Coiled-Coil Containing Protein 3 | Protein Coding | 43 | GC04P001723 | 2.98 | https://www.genecards.org/cgi-bin/carddisp.pl?gene=TACC3 |
| TSPAN8 | Tetraspanin 8 | Protein Coding | 39 | GC12M071125 | 2.96 | https://www.genecards.org/cgi-bin/carddisp.pl?gene=TSPAN8 |
| LATS2 | Large Tumor Suppressor Kinase 2 | Protein Coding | 44 | GC13M020973 | 2.96 | https://www.genecards.org/cgi-bin/carddisp.pl?gene=LATS2 |
| VCP | Valosin Containing Protein | Protein Coding | 48 | GC09M035056 | 2.96 | https://www.genecards.org/cgi-bin/carddisp.pl?gene=VCP |
| FLG | Filaggrin | Protein Coding | 40 | GC01M152274 | 2.95 | https://www.genecards.org/cgi-bin/carddisp.pl?gene=FLG |
| STYK1 | Serine/Threonine/Tyrosine Kinase 1 | Protein Coding | 40 | GC12M013873 | 2.95 | https://www.genecards.org/cgi-bin/carddisp.pl?gene=STYK1 |
| RERG | RAS Like Estrogen Regulated Growth Inhibitor | Protein Coding | 38 | GC12M015151 | 2.94 | https://www.genecards.org/cgi-bin/carddisp.pl?gene=RERG |
| NRIP1 | Nuclear Receptor Interacting Protein 1 | Protein Coding | 43 | GC21M014961 | 2.94 | https://www.genecards.org/cgi-bin/carddisp.pl?gene=NRIP1 |
| MIR1-1 | MicroRNA 1-1 | RNA Gene | 19 | GC20P062893 | 2.93 | https://www.genecards.org/cgi-bin/carddisp.pl?gene=MIR1-1 |
| FZR1 | Fizzy And Cell Division Cycle 20 Related 1 | Protein Coding | 42 | GC19P003506 | 2.93 | https://www.genecards.org/cgi-bin/carddisp.pl?gene=FZR1 |
| TBL1XR1 | TBL1X Receptor 1 | Protein Coding | 44 | GC03M177019 | 2.93 | https://www.genecards.org/cgi-bin/carddisp.pl?gene=TBL1XR1 |
| UGT1A1 | UDP Glucuronosyltransferase Family 1 Member A1 | Protein Coding | 48 | GC02P233760 | 2.92 | https://www.genecards.org/cgi-bin/carddisp.pl?gene=UGT1A1 |
| TET2 | Tet Methylcytosine Dioxygenase 2 | Protein Coding | 44 | GC04P105145 | 2.92 | https://www.genecards.org/cgi-bin/carddisp.pl?gene=TET2 |
| TEP1 | Telomerase Associated Protein 1 | Protein Coding | 40 | GC14M020365 | 2.91 | https://www.genecards.org/cgi-bin/carddisp.pl?gene=TEP1 |
| SOX10 | SRY-Box Transcription Factor 10 | Protein Coding | 45 | GC22M046233 | 2.9 | https://www.genecards.org/cgi-bin/carddisp.pl?gene=SOX10 |
| DBT | Dihydrolipoamide Branched Chain Transacylase E2 | Protein Coding | 43 | GC01M100186 | 2.88 | https://www.genecards.org/cgi-bin/carddisp.pl?gene=DBT |
| CSNK2B | Casein Kinase 2 Beta | Protein Coding | 47 | GC06P047308 | 2.88 | https://www.genecards.org/cgi-bin/carddisp.pl?gene=CSNK2B |
| RASSF2 | Ras Association Domain Family Member 2 | Protein Coding | 41 | GC20M004780 | 2.88 | https://www.genecards.org/cgi-bin/carddisp.pl?gene=RASSF2 |
| EIF4G1 | Eukaryotic Translation Initiation Factor 4 Gamma 1 | Protein Coding | 45 | GC03P184314 | 2.88 | https://www.genecards.org/cgi-bin/carddisp.pl?gene=EIF4G1 |
| CCL7 | C-C Motif Chemokine Ligand 7 | Protein Coding | 41 | GC17P034270 | 2.87 | https://www.genecards.org/cgi-bin/carddisp.pl?gene=CCL7 |
| CYP2C9 | Cytochrome P450 Family 2 Subfamily C Member 9 | Protein Coding | 48 | GC10P094938 | 2.87 | https://www.genecards.org/cgi-bin/carddisp.pl?gene=CYP2C9 |
| PKP3 | Plakophilin 3 | Protein Coding | 37 | GC11P000394 | 2.87 | https://www.genecards.org/cgi-bin/carddisp.pl?gene=PKP3 |
| VWA5A | Von Willebrand Factor A Domain Containing 5A | Protein Coding | 34 | GC11P124115 | 2.86 | https://www.genecards.org/cgi-bin/carddisp.pl?gene=VWA5A |
| CACNA2D2 | Calcium Voltage-Gated Channel Auxiliary Subunit Alpha2delta 2 | Protein Coding | 41 | GC03M050385 | 2.86 | https://www.genecards.org/cgi-bin/carddisp.pl?gene=CACNA2D2 |
| G6PD | Glucose-6-Phosphate Dehydrogenase | Protein Coding | 50 | GC0XM154531 | 2.86 | https://www.genecards.org/cgi-bin/carddisp.pl?gene=G6PD |
| HELLS | Helicase, Lymphoid Specific | Protein Coding | 45 | GC10P094501 | 2.85 | https://www.genecards.org/cgi-bin/carddisp.pl?gene=HELLS |
| MIR383 | MicroRNA 383 | RNA Gene | 15 | GC08M014853 | 2.85 | https://www.genecards.org/cgi-bin/carddisp.pl?gene=MIR383 |
| DUSP1 | Dual Specificity Phosphatase 1 | Protein Coding | 47 | GC05M172768 | 2.84 | https://www.genecards.org/cgi-bin/carddisp.pl?gene=DUSP1 |
| SEMA3F | Semaphorin 3F | Protein Coding | 41 | GC03P050167 | 2.84 | https://www.genecards.org/cgi-bin/carddisp.pl?gene=SEMA3F |
| GNB1L | G Protein Subunit Beta 1 Like | Protein Coding | 36 | GC22M019770 | 2.84 | https://www.genecards.org/cgi-bin/carddisp.pl?gene=GNB1L |
| CSTA | Cystatin A | Protein Coding | 44 | GC03P122325 | 2.84 | https://www.genecards.org/cgi-bin/carddisp.pl?gene=CSTA |
| LAMB1 | Laminin Subunit Beta 1 | Protein Coding | 47 | GC07M107923 | 2.83 | https://www.genecards.org/cgi-bin/carddisp.pl?gene=LAMB1 |
| MIR134 | MicroRNA 134 | RNA Gene | 18 | GC14P104777 | 2.82 | https://www.genecards.org/cgi-bin/carddisp.pl?gene=MIR134 |
| CR1 | Complement C3b/C4b Receptor 1 (Knops Blood Group) | Protein Coding | 44 | GC01P207496 | 2.82 | https://www.genecards.org/cgi-bin/carddisp.pl?gene=CR1 |
| ANLN | Anillin Actin Binding Protein | Protein Coding | 41 | GC07P036389 | 2.81 | https://www.genecards.org/cgi-bin/carddisp.pl?gene=ANLN |
| CLIC1 | Chloride Intracellular Channel 1 | Protein Coding | 41 | GC06M032620 | 2.81 | https://www.genecards.org/cgi-bin/carddisp.pl?gene=CLIC1 |
| MIR19B1 | MicroRNA 19b-1 | RNA Gene | 17 | GC13P091435 | 2.79 | https://www.genecards.org/cgi-bin/carddisp.pl?gene=MIR19B1 |
| KLK1 | Kallikrein 1 | Protein Coding | 45 | GC19M050819 | 2.79 | https://www.genecards.org/cgi-bin/carddisp.pl?gene=KLK1 |
| ITGAL | Integrin Subunit Alpha L | Protein Coding | 45 | GC16P030472 | 2.79 | https://www.genecards.org/cgi-bin/carddisp.pl?gene=ITGAL |
| IL11 | Interleukin 11 | Protein Coding | 41 | GC19M055364 | 2.79 | https://www.genecards.org/cgi-bin/carddisp.pl?gene=IL11 |
| SETDB1 | SET Domain Bifurcated Histone Lysine Methyltransferase 1 | Protein Coding | 41 | GC01P150926 | 2.79 | https://www.genecards.org/cgi-bin/carddisp.pl?gene=SETDB1 |
| ULK1 | Unc-51 Like Autophagy Activating Kinase 1 | Protein Coding | 45 | GC12P131894 | 2.79 | https://www.genecards.org/cgi-bin/carddisp.pl?gene=ULK1 |
| ZNF365 | Zinc Finger Protein 365 | Protein Coding | 37 | GC10P062374 | 2.78 | https://www.genecards.org/cgi-bin/carddisp.pl?gene=ZNF365 |
| PPIA | Peptidylprolyl Isomerase A | Protein Coding | 47 | GC07P044807 | 2.78 | https://www.genecards.org/cgi-bin/carddisp.pl?gene=PPIA |
| LBR | Lamin B Receptor | Protein Coding | 47 | GC01M225401 | 2.78 | https://www.genecards.org/cgi-bin/carddisp.pl?gene=LBR |
| VCL | Vinculin | Protein Coding | 47 | GC10P073995 | 2.77 | https://www.genecards.org/cgi-bin/carddisp.pl?gene=VCL |
| MED15 | Mediator Complex Subunit 15 | Protein Coding | 39 | GC22P020496 | 2.77 | https://www.genecards.org/cgi-bin/carddisp.pl?gene=MED15 |
| ZNF671 | Zinc Finger Protein 671 | Protein Coding | 33 | GC19M057719 | 2.77 | https://www.genecards.org/cgi-bin/carddisp.pl?gene=ZNF671 |
| RBMS3 | RNA Binding Motif Single Stranded Interacting Protein 3 | Protein Coding | 35 | GC03P028575 | 2.77 | https://www.genecards.org/cgi-bin/carddisp.pl?gene=RBMS3 |
| IGFBP6 | Insulin Like Growth Factor Binding Protein 6 | Protein Coding | 42 | GC12P053097 | 2.76 | https://www.genecards.org/cgi-bin/carddisp.pl?gene=IGFBP6 |
| FLNA | Filamin A | Protein Coding | 49 | GC0XM154348 | 2.76 | https://www.genecards.org/cgi-bin/carddisp.pl?gene=FLNA |
| LINC00520 | Long Intergenic Non-Protein Coding RNA 520 | RNA Gene | 17 | GC14M055781 | 2.76 | https://www.genecards.org/cgi-bin/carddisp.pl?gene=LINC00520 |
| TAP2 | Transporter 2, ATP Binding Cassette Subfamily B Member | Protein Coding | 44 | GC06M032821 | 2.76 | https://www.genecards.org/cgi-bin/carddisp.pl?gene=TAP2 |
| STARD3 | StAR Related Lipid Transfer Domain Containing 3 | Protein Coding | 39 | GC17P039637 | 2.75 | https://www.genecards.org/cgi-bin/carddisp.pl?gene=STARD3 |
| NUAK1 | NUAK Family Kinase 1 | Protein Coding | 43 | GC12M106063 | 2.74 | https://www.genecards.org/cgi-bin/carddisp.pl?gene=NUAK1 |
| APOE | Apolipoprotein E | Protein Coding | 50 | GC19P044906 | 2.74 | https://www.genecards.org/cgi-bin/carddisp.pl?gene=APOE |
| TCF3 | Transcription Factor 3 | Protein Coding | 45 | GC19M001609 | 2.73 | https://www.genecards.org/cgi-bin/carddisp.pl?gene=TCF3 |
| SCARB1 | Scavenger Receptor Class B Member 1 | Protein Coding | 45 | GC12M124776 | 2.71 | https://www.genecards.org/cgi-bin/carddisp.pl?gene=SCARB1 |
| DDX58 | DExD/H-Box Helicase 58 | Protein Coding | 47 | GC09M032455 | 2.71 | https://www.genecards.org/cgi-bin/carddisp.pl?gene=DDX58 |
| GALC | Galactosylceramidase | Protein Coding | 43 | GC14M087837 | 2.7 | https://www.genecards.org/cgi-bin/carddisp.pl?gene=GALC |
| ARSB | Arylsulfatase B | Protein Coding | 44 | GC05M078777 | 2.7 | https://www.genecards.org/cgi-bin/carddisp.pl?gene=ARSB |
| RCVRN | Recoverin | Protein Coding | 38 | GC17M009896 | 2.69 | https://www.genecards.org/cgi-bin/carddisp.pl?gene=RCVRN |
| TLR10 | Toll Like Receptor 10 | Protein Coding | 40 | GC04M038773 | 2.69 | https://www.genecards.org/cgi-bin/carddisp.pl?gene=TLR10 |
| ASNS | Asparagine Synthetase (Glutamine-Hydrolyzing) | Protein Coding | 46 | GC07M097854 | 2.68 | https://www.genecards.org/cgi-bin/carddisp.pl?gene=ASNS |
| USP22 | Ubiquitin Specific Peptidase 22 | Protein Coding | 41 | GC17M020999 | 2.68 | https://www.genecards.org/cgi-bin/carddisp.pl?gene=USP22 |
| CYP4B1 | Cytochrome P450 Family 4 Subfamily B Member 1 | Protein Coding | 43 | GC01P046757 | 2.68 | https://www.genecards.org/cgi-bin/carddisp.pl?gene=CYP4B1 |
| NEU1 | Neuraminidase 1 | Protein Coding | 44 | GC06M031857 | 2.68 | https://www.genecards.org/cgi-bin/carddisp.pl?gene=NEU1 |
| UBE2T | Ubiquitin Conjugating Enzyme E2 T | Protein Coding | 43 | GC01M202300 | 2.67 | https://www.genecards.org/cgi-bin/carddisp.pl?gene=UBE2T |
| LTA | Lymphotoxin Alpha | Protein Coding | 42 | GC06P047303 | 2.66 | https://www.genecards.org/cgi-bin/carddisp.pl?gene=LTA |
| RCN1 | Reticulocalbin 1 | Protein Coding | 39 | GC11P032090 | 2.65 | https://www.genecards.org/cgi-bin/carddisp.pl?gene=RCN1 |
| GABBR1 | Gamma-Aminobutyric Acid Type B Receptor Subunit 1 | Protein Coding | 47 | GC06M029555 | 2.64 | https://www.genecards.org/cgi-bin/carddisp.pl?gene=GABBR1 |
| PDE5A | Phosphodiesterase 5A | Protein Coding | 44 | GC04M119494 | 2.63 | https://www.genecards.org/cgi-bin/carddisp.pl?gene=PDE5A |
| CAPNS1 | Calpain Small Subunit 1 | Protein Coding | 41 | GC19P038254 | 2.63 | https://www.genecards.org/cgi-bin/carddisp.pl?gene=CAPNS1 |
| CLCA2 | Chloride Channel Accessory 2 | Protein Coding | 41 | GC01P086424 | 2.63 | https://www.genecards.org/cgi-bin/carddisp.pl?gene=CLCA2 |
| SCGB3A1 | Secretoglobin Family 3A Member 1 | Protein Coding | 35 | GC05M180590 | 2.63 | https://www.genecards.org/cgi-bin/carddisp.pl?gene=SCGB3A1 |
| PRMT5 | Protein Arginine Methyltransferase 5 | Protein Coding | 42 | GC14M022920 | 2.62 | https://www.genecards.org/cgi-bin/carddisp.pl?gene=PRMT5 |
| TBXT | T-Box Transcription Factor T | Protein Coding | 32 | GC06M166158 | 2.62 | https://www.genecards.org/cgi-bin/carddisp.pl?gene=TBXT |
| PRDX5 | Peroxiredoxin 5 | Protein Coding | 46 | GC11P064317 | 2.61 | https://www.genecards.org/cgi-bin/carddisp.pl?gene=PRDX5 |
| SERPINC1 | Serpin Family C Member 1 | Protein Coding | 48 | GC01M174153 | 2.6 | https://www.genecards.org/cgi-bin/carddisp.pl?gene=SERPINC1 |
| THORLNC | Testis Associated Oncogenic LncRNA | RNA Gene | 10 | GC02M118133 | 2.6 | https://www.genecards.org/cgi-bin/carddisp.pl?gene=THORLNC |
| RIPK4 | Receptor Interacting Serine/Threonine Kinase 4 | Protein Coding | 43 | GC21M041739 | 2.6 | https://www.genecards.org/cgi-bin/carddisp.pl?gene=RIPK4 |
| CCDC136 | Coiled-Coil Domain Containing 136 | Protein Coding | 35 | GC07P129455 | 2.59 | https://www.genecards.org/cgi-bin/carddisp.pl?gene=CCDC136 |
| REN | Renin | Protein Coding | 48 | GC01M204154 | 2.59 | https://www.genecards.org/cgi-bin/carddisp.pl?gene=REN |
| TMPRSS3 | Transmembrane Serine Protease 3 | Protein Coding | 41 | GC21M042371 | 2.59 | https://www.genecards.org/cgi-bin/carddisp.pl?gene=TMPRSS3 |
| C5 | Complement C5 | Protein Coding | 46 | GC09M120952 | 2.58 | https://www.genecards.org/cgi-bin/carddisp.pl?gene=C5 |
| SOX1 | SRY-Box Transcription Factor 1 | Protein Coding | 36 | GC13P112067 | 2.58 | https://www.genecards.org/cgi-bin/carddisp.pl?gene=SOX1 |
| PTP4A3 | Protein Tyrosine Phosphatase 4A3 | Protein Coding | 41 | GC08P141391 | 2.57 | https://www.genecards.org/cgi-bin/carddisp.pl?gene=PTP4A3 |
| PPARGC1A | PPARG Coactivator 1 Alpha | Protein Coding | 46 | GC04M023755 | 2.56 | https://www.genecards.org/cgi-bin/carddisp.pl?gene=PPARGC1A |
| CDX1 | Caudal Type Homeobox 1 | Protein Coding | 36 | GC05P150133 | 2.55 | https://www.genecards.org/cgi-bin/carddisp.pl?gene=CDX1 |
| SESN2 | Sestrin 2 | Protein Coding | 39 | GC01P028270 | 2.55 | https://www.genecards.org/cgi-bin/carddisp.pl?gene=SESN2 |
| MED1 | Mediator Complex Subunit 1 | Protein Coding | 41 | GC17M039404 | 2.55 | https://www.genecards.org/cgi-bin/carddisp.pl?gene=MED1 |
| TIA1 | TIA1 Cytotoxic Granule Associated RNA Binding Protein | Protein Coding | 43 | GC02M070209 | 2.55 | https://www.genecards.org/cgi-bin/carddisp.pl?gene=TIA1 |
| PAK2 | P21 (RAC1) Activated Kinase 2 | Protein Coding | 46 | GC03P196739 | 2.54 | https://www.genecards.org/cgi-bin/carddisp.pl?gene=PAK2 |
| TRIM29 | Tripartite Motif Containing 29 | Protein Coding | 39 | GC11M120111 | 2.54 | https://www.genecards.org/cgi-bin/carddisp.pl?gene=TRIM29 |
| CCR3 | C-C Motif Chemokine Receptor 3 | Protein Coding | 47 | GC03P046227 | 2.53 | https://www.genecards.org/cgi-bin/carddisp.pl?gene=CCR3 |
| SP3 | Sp3 Transcription Factor | Protein Coding | 42 | GC02M173882 | 2.53 | https://www.genecards.org/cgi-bin/carddisp.pl?gene=SP3 |
| FSTL1 | Follistatin Like 1 | Protein Coding | 41 | GC03M120392 | 2.53 | https://www.genecards.org/cgi-bin/carddisp.pl?gene=FSTL1 |
| THRA | Thyroid Hormone Receptor Alpha | Protein Coding | 48 | GC17P040058 | 2.52 | https://www.genecards.org/cgi-bin/carddisp.pl?gene=THRA |
| MBL2 | Mannose Binding Lectin 2 | Protein Coding | 47 | GC10M052760 | 2.52 | https://www.genecards.org/cgi-bin/carddisp.pl?gene=MBL2 |
| MAPK11 | Mitogen-Activated Protein Kinase 11 | Protein Coding | 48 | GC22M050263 | 2.52 | https://www.genecards.org/cgi-bin/carddisp.pl?gene=MAPK11 |
| HDAC4 | Histone Deacetylase 4 | Protein Coding | 51 | GC02M239048 | 2.51 | https://www.genecards.org/cgi-bin/carddisp.pl?gene=HDAC4 |
| CHI3L1 | Chitinase 3 Like 1 | Protein Coding | 43 | GC01M203148 | 2.51 | https://www.genecards.org/cgi-bin/carddisp.pl?gene=CHI3L1 |
| FOXC2 | Forkhead Box C2 | Protein Coding | 44 | GC16P086567 | 2.51 | https://www.genecards.org/cgi-bin/carddisp.pl?gene=FOXC2 |
| ETV7 | ETS Variant Transcription Factor 7 | Protein Coding | 36 | GC06M042229 | 2.49 | https://www.genecards.org/cgi-bin/carddisp.pl?gene=ETV7 |
| ERP29 | Endoplasmic Reticulum Protein 29 | Protein Coding | 35 | GC12P112013 | 2.49 | https://www.genecards.org/cgi-bin/carddisp.pl?gene=ERP29 |
| COL3A1 | Collagen Type III Alpha 1 Chain | Protein Coding | 47 | GC02P188974 | 2.49 | https://www.genecards.org/cgi-bin/carddisp.pl?gene=COL3A1 |
| TUSC1 | Tumor Suppressor Candidate 1 | Protein Coding | 28 | GC09M025668 | 2.48 | https://www.genecards.org/cgi-bin/carddisp.pl?gene=TUSC1 |
| KAT5 | Lysine Acetyltransferase 5 | Protein Coding | 46 | GC11P065711 | 2.48 | https://www.genecards.org/cgi-bin/carddisp.pl?gene=KAT5 |
| ELANE | Elastase, Neutrophil Expressed | Protein Coding | 46 | GC19P000854 | 2.48 | https://www.genecards.org/cgi-bin/carddisp.pl?gene=ELANE |
| CEBPD | CCAAT Enhancer Binding Protein Delta | Protein Coding | 38 | GC08M047759 | 2.47 | https://www.genecards.org/cgi-bin/carddisp.pl?gene=CEBPD |
| HDAC3 | Histone Deacetylase 3 | Protein Coding | 49 | GC05M141583 | 2.46 | https://www.genecards.org/cgi-bin/carddisp.pl?gene=HDAC3 |
| TRPS1 | Transcriptional Repressor GATA Binding 1 | Protein Coding | 45 | GC08M115408 | 2.46 | https://www.genecards.org/cgi-bin/carddisp.pl?gene=TRPS1 |
| PTMA | Prothymosin Alpha | Protein Coding | 39 | GC02P231707 | 2.45 | https://www.genecards.org/cgi-bin/carddisp.pl?gene=PTMA |
| SOCS6 | Suppressor Of Cytokine Signaling 6 | Protein Coding | 41 | GC18P070288 | 2.45 | https://www.genecards.org/cgi-bin/carddisp.pl?gene=SOCS6 |
| CEL | Carboxyl Ester Lipase | Protein Coding | 46 | GC09P133061 | 2.44 | https://www.genecards.org/cgi-bin/carddisp.pl?gene=CEL |
| CDK12 | Cyclin Dependent Kinase 12 | Protein Coding | 40 | GC17P039461 | 2.44 | https://www.genecards.org/cgi-bin/carddisp.pl?gene=CDK12 |
| CST3 | Cystatin C | Protein Coding | 44 | GC20M023608 | 2.43 | https://www.genecards.org/cgi-bin/carddisp.pl?gene=CST3 |
| ZNF750 | Zinc Finger Protein 750 | Protein Coding | 36 | GC17M082829 | 2.42 | https://www.genecards.org/cgi-bin/carddisp.pl?gene=ZNF750 |
| NUDT6 | Nudix Hydrolase 6 | Protein Coding | 37 | GC04M122888 | 2.41 | https://www.genecards.org/cgi-bin/carddisp.pl?gene=NUDT6 |
| AIP | Aryl Hydrocarbon Receptor Interacting Protein | Protein Coding | 44 | GC11P067468 | 2.41 | https://www.genecards.org/cgi-bin/carddisp.pl?gene=AIP |
| SRGN | Serglycin | Protein Coding | 38 | GC10P069088 | 2.4 | https://www.genecards.org/cgi-bin/carddisp.pl?gene=SRGN |
| SCGB2A1 | Secretoglobin Family 2A Member 1 | Protein Coding | 34 | GC11P062227 | 2.4 | https://www.genecards.org/cgi-bin/carddisp.pl?gene=SCGB2A1 |
| HDAC5 | Histone Deacetylase 5 | Protein Coding | 47 | GC17M044076 | 2.4 | https://www.genecards.org/cgi-bin/carddisp.pl?gene=HDAC5 |
| SLC16A1 | Solute Carrier Family 16 Member 1 | Protein Coding | 48 | GC01M112913 | 2.4 | https://www.genecards.org/cgi-bin/carddisp.pl?gene=SLC16A1 |
| CENPH | Centromere Protein H | Protein Coding | 34 | GC05P069189 | 2.4 | https://www.genecards.org/cgi-bin/carddisp.pl?gene=CENPH |
| CTNNBIP1 | Catenin Beta Interacting Protein 1 | Protein Coding | 41 | GC01M009848 | 2.4 | https://www.genecards.org/cgi-bin/carddisp.pl?gene=CTNNBIP1 |
| CBL | Cbl Proto-Oncogene | Protein Coding | 50 | GC11P119206 | 2.4 | https://www.genecards.org/cgi-bin/carddisp.pl?gene=CBL |
| TLR1 | Toll Like Receptor 1 | Protein Coding | 47 | GC04M038797 | 2.39 | https://www.genecards.org/cgi-bin/carddisp.pl?gene=TLR1 |
| CDK2AP1 | Cyclin Dependent Kinase 2 Associated Protein 1 | Protein Coding | 38 | GC12M123260 | 2.39 | https://www.genecards.org/cgi-bin/carddisp.pl?gene=CDK2AP1 |
| BCL2L12 | BCL2 Like 12 | Protein Coding | 37 | GC19P049665 | 2.38 | https://www.genecards.org/cgi-bin/carddisp.pl?gene=BCL2L12 |
| CEP55 | Centrosomal Protein 55 | Protein Coding | 40 | GC10P093496 | 2.38 | https://www.genecards.org/cgi-bin/carddisp.pl?gene=CEP55 |
| DACT2 | Dishevelled Binding Antagonist Of Beta Catenin 2 | Protein Coding | 35 | GC06M168292 | 2.37 | https://www.genecards.org/cgi-bin/carddisp.pl?gene=DACT2 |
| MRPL47 | Mitochondrial Ribosomal Protein L47 | Protein Coding | 33 | GC03M179588 | 2.37 | https://www.genecards.org/cgi-bin/carddisp.pl?gene=MRPL47 |
| MAGED2 | MAGE Family Member D2 | Protein Coding | 39 | GC0XP054807 | 2.37 | https://www.genecards.org/cgi-bin/carddisp.pl?gene=MAGED2 |
| SUMO2P1 | SUMO2 Pseudogene 1 | Pseudogene | 8 | GC06M029639 | 2.36 | https://www.genecards.org/cgi-bin/carddisp.pl?gene=SUMO2P1 |
| ABCC5 | ATP Binding Cassette Subfamily C Member 5 | Protein Coding | 43 | GC03M183919 | 2.36 | https://www.genecards.org/cgi-bin/carddisp.pl?gene=ABCC5 |
| SOX5 | SRY-Box Transcription Factor 5 | Protein Coding | 46 | GC12M023529 | 2.36 | https://www.genecards.org/cgi-bin/carddisp.pl?gene=SOX5 |
| MAPRE1 | Microtubule Associated Protein RP/EB Family Member 1 | Protein Coding | 44 | GC20P032819 | 2.35 | https://www.genecards.org/cgi-bin/carddisp.pl?gene=MAPRE1 |
| LINC00476 | Long Intergenic Non-Protein Coding RNA 476 | RNA Gene | 18 | GC09M095759 | 2.35 | https://www.genecards.org/cgi-bin/carddisp.pl?gene=LINC00476 |
| SIRT2 | Sirtuin 2 | Protein Coding | 48 | GC19M038878 | 2.35 | https://www.genecards.org/cgi-bin/carddisp.pl?gene=SIRT2 |
| FLOT1 | Flotillin 1 | Protein Coding | 41 | GC06M030904 | 2.33 | https://www.genecards.org/cgi-bin/carddisp.pl?gene=FLOT1 |
| PON1 | Paraoxonase 1 | Protein Coding | 45 | GC07M095297 | 2.33 | https://www.genecards.org/cgi-bin/carddisp.pl?gene=PON1 |
| LAMP2 | Lysosomal Associated Membrane Protein 2 | Protein Coding | 44 | GC0XM120426 | 2.33 | https://www.genecards.org/cgi-bin/carddisp.pl?gene=LAMP2 |
| TLR8 | Toll Like Receptor 8 | Protein Coding | 47 | GC0XP012924 | 2.32 | https://www.genecards.org/cgi-bin/carddisp.pl?gene=TLR8 |
| MSH5 | MutS Homolog 5 | Protein Coding | 41 | GC06P047322 | 2.31 | https://www.genecards.org/cgi-bin/carddisp.pl?gene=MSH5 |
| HLA-DRA | Major Histocompatibility Complex, Class II, DR Alpha | Protein Coding | 47 | GC06P032439 | 2.3 | https://www.genecards.org/cgi-bin/carddisp.pl?gene=HLA-DRA |
| EGLN2 | Egl-9 Family Hypoxia Inducible Factor 2 | Protein Coding | 44 | GC19P040799 | 2.3 | https://www.genecards.org/cgi-bin/carddisp.pl?gene=EGLN2 |
| CDK10 | Cyclin Dependent Kinase 10 | Protein Coding | 44 | GC16P089680 | 2.3 | https://www.genecards.org/cgi-bin/carddisp.pl?gene=CDK10 |
| XAF1 | XIAP Associated Factor 1 | Protein Coding | 39 | GC17P006757 | 2.3 | https://www.genecards.org/cgi-bin/carddisp.pl?gene=XAF1 |
| FOXP1 | Forkhead Box P1 | Protein Coding | 45 | GC03M070926 | 2.3 | https://www.genecards.org/cgi-bin/carddisp.pl?gene=FOXP1 |
| IL12B | Interleukin 12B | Protein Coding | 44 | GC05M159314 | 2.29 | https://www.genecards.org/cgi-bin/carddisp.pl?gene=IL12B |
| BCAT1 | Branched Chain Amino Acid Transaminase 1 | Protein Coding | 46 | GC12M024732 | 2.28 | https://www.genecards.org/cgi-bin/carddisp.pl?gene=BCAT1 |
| PCOTH | Pro-X-Gly Collagen Triple Helix Like Repeat Containing | Protein Coding | 22 | GC13P023889 | 2.28 | https://www.genecards.org/cgi-bin/carddisp.pl?gene=PCOTH |
| TLN1 | Talin 1 | Protein Coding | 41 | GC09M035687 | 2.27 | https://www.genecards.org/cgi-bin/carddisp.pl?gene=TLN1 |
| KCNA4 | Potassium Voltage-Gated Channel Subfamily A Member 4 | Protein Coding | 43 | GC11M030009 | 2.27 | https://www.genecards.org/cgi-bin/carddisp.pl?gene=KCNA4 |
| NCR1 | Natural Cytotoxicity Triggering Receptor 1 | Protein Coding | 37 | GC19P054906 | 2.27 | https://www.genecards.org/cgi-bin/carddisp.pl?gene=NCR1 |
| MIR101-2 | MicroRNA 101-2 | RNA Gene | 16 | GC09P004863 | 2.27 | https://www.genecards.org/cgi-bin/carddisp.pl?gene=MIR101-2 |
| CCL18 | C-C Motif Chemokine Ligand 18 | Protein Coding | 36 | GC17P036064 | 2.27 | https://www.genecards.org/cgi-bin/carddisp.pl?gene=CCL18 |
| UTP6 | UTP6 Small Subunit Processome Component | Protein Coding | 37 | GC17M031860 | 2.26 | https://www.genecards.org/cgi-bin/carddisp.pl?gene=UTP6 |
| RPA3 | Replication Protein A3 | Protein Coding | 41 | GC07M007637 | 2.25 | https://www.genecards.org/cgi-bin/carddisp.pl?gene=RPA3 |
| CD209 | CD209 Molecule | Protein Coding | 43 | GC19M007739 | 2.24 | https://www.genecards.org/cgi-bin/carddisp.pl?gene=CD209 |
| GLP1R | Glucagon Like Peptide 1 Receptor | Protein Coding | 46 | GC06P039048 | 2.24 | https://www.genecards.org/cgi-bin/carddisp.pl?gene=GLP1R |
| DEFB1 | Defensin Beta 1 | Protein Coding | 39 | GC08M006870 | 2.23 | https://www.genecards.org/cgi-bin/carddisp.pl?gene=DEFB1 |
| CDKN2D | Cyclin Dependent Kinase Inhibitor 2D | Protein Coding | 40 | GC19M010566 | 2.23 | https://www.genecards.org/cgi-bin/carddisp.pl?gene=CDKN2D |
| SCYL1 | SCY1 Like Pseudokinase 1 | Protein Coding | 41 | GC11P065525 | 2.23 | https://www.genecards.org/cgi-bin/carddisp.pl?gene=SCYL1 |
| CXCL16 | C-X-C Motif Chemokine Ligand 16 | Protein Coding | 40 | GC17M004733 | 2.23 | https://www.genecards.org/cgi-bin/carddisp.pl?gene=CXCL16 |
| EIF5A2 | Eukaryotic Translation Initiation Factor 5A2 | Protein Coding | 40 | GC03M170888 | 2.22 | https://www.genecards.org/cgi-bin/carddisp.pl?gene=EIF5A2 |
| TRPC1 | Transient Receptor Potential Cation Channel Subfamily C Member 1 | Protein Coding | 42 | GC03P142724 | 2.22 | https://www.genecards.org/cgi-bin/carddisp.pl?gene=TRPC1 |
| CD38 | CD38 Molecule | Protein Coding | 45 | GC04P015779 | 2.22 | https://www.genecards.org/cgi-bin/carddisp.pl?gene=CD38 |
| PLA2G10 | Phospholipase A2 Group X | Protein Coding | 44 | GC16M014672 | 2.21 | https://www.genecards.org/cgi-bin/carddisp.pl?gene=PLA2G10 |
| NEK2 | NIMA Related Kinase 2 | Protein Coding | 50 | GC01M211658 | 2.21 | https://www.genecards.org/cgi-bin/carddisp.pl?gene=NEK2 |
| SERPINF1 | Serpin Family F Member 1 | Protein Coding | 44 | GC17P001761 | 2.2 | https://www.genecards.org/cgi-bin/carddisp.pl?gene=SERPINF1 |
| HSPA9 | Heat Shock Protein Family A (Hsp70) Member 9 | Protein Coding | 47 | GC05M138554 | 2.2 | https://www.genecards.org/cgi-bin/carddisp.pl?gene=HSPA9 |
| SYCP1 | Synaptonemal Complex Protein 1 | Protein Coding | 36 | GC01P114854 | 2.19 | https://www.genecards.org/cgi-bin/carddisp.pl?gene=SYCP1 |
| ZFX | Zinc Finger Protein X-Linked | Protein Coding | 37 | GC0XP024148 | 2.19 | https://www.genecards.org/cgi-bin/carddisp.pl?gene=ZFX |
| HOXC13 | Homeobox C13 | Protein Coding | 40 | GC12P053938 | 2.18 | https://www.genecards.org/cgi-bin/carddisp.pl?gene=HOXC13 |
| TNFAIP2 | TNF Alpha Induced Protein 2 | Protein Coding | 39 | GC14P104642 | 2.18 | https://www.genecards.org/cgi-bin/carddisp.pl?gene=TNFAIP2 |
| ING4 | Inhibitor Of Growth Family Member 4 | Protein Coding | 36 | GC12M006650 | 2.17 | https://www.genecards.org/cgi-bin/carddisp.pl?gene=ING4 |
| MIR1307 | MicroRNA 1307 | RNA Gene | 17 | GC10M103394 | 2.17 | https://www.genecards.org/cgi-bin/carddisp.pl?gene=MIR1307 |
| MT2A | Metallothionein 2A | Protein Coding | 42 | GC16P056627 | 2.17 | https://www.genecards.org/cgi-bin/carddisp.pl?gene=MT2A |
| CENPF | Centromere Protein F | Protein Coding | 42 | GC01P214603 | 2.17 | https://www.genecards.org/cgi-bin/carddisp.pl?gene=CENPF |
| BTG1 | BTG Anti-Proliferation Factor 1 | Protein Coding | 41 | GC12M092140 | 2.17 | https://www.genecards.org/cgi-bin/carddisp.pl?gene=BTG1 |
| PFN1 | Profilin 1 | Protein Coding | 47 | GC17M004945 | 2.16 | https://www.genecards.org/cgi-bin/carddisp.pl?gene=PFN1 |
| FEZF1 | FEZ Family Zinc Finger 1 | Protein Coding | 39 | GC07M122301 | 2.16 | https://www.genecards.org/cgi-bin/carddisp.pl?gene=FEZF1 |
| CIITA | Class II Major Histocompatibility Complex Transactivator | Protein Coding | 45 | GC16P010879 | 2.16 | https://www.genecards.org/cgi-bin/carddisp.pl?gene=CIITA |
| PKD1 | Polycystin 1, Transient Receptor Potential Channel Interacting | Protein Coding | 45 | GC16M002348 | 2.16 | https://www.genecards.org/cgi-bin/carddisp.pl?gene=PKD1 |
| HEPH | Hephaestin | Protein Coding | 39 | GC0XP066162 | 2.15 | https://www.genecards.org/cgi-bin/carddisp.pl?gene=HEPH |
| CCNT1 | Cyclin T1 | Protein Coding | 41 | GC12M048688 | 2.14 | https://www.genecards.org/cgi-bin/carddisp.pl?gene=CCNT1 |
| MEF2D | Myocyte Enhancer Factor 2D | Protein Coding | 43 | GC01M156463 | 2.13 | https://www.genecards.org/cgi-bin/carddisp.pl?gene=MEF2D |
| BTRC | Beta-Transducin Repeat Containing E3 Ubiquitin Protein Ligase | Protein Coding | 46 | GC10P101354 | 2.13 | https://www.genecards.org/cgi-bin/carddisp.pl?gene=BTRC |
| IDUA | Alpha-L-Iduronidase | Protein Coding | 42 | GC04P000986 | 2.12 | https://www.genecards.org/cgi-bin/carddisp.pl?gene=IDUA |
| TKTL2 | Transketolase Like 2 | Protein Coding | 37 | GC04M163471 | 2.12 | https://www.genecards.org/cgi-bin/carddisp.pl?gene=TKTL2 |
| SIAH1 | Siah E3 Ubiquitin Protein Ligase 1 | Protein Coding | 45 | GC16M048357 | 2.11 | https://www.genecards.org/cgi-bin/carddisp.pl?gene=SIAH1 |
| RTN4 | Reticulon 4 | Protein Coding | 44 | GC02M054934 | 2.11 | https://www.genecards.org/cgi-bin/carddisp.pl?gene=RTN4 |
| RIPK1 | Receptor Interacting Serine/Threonine Kinase 1 | Protein Coding | 49 | GC06P003064 | 2.11 | https://www.genecards.org/cgi-bin/carddisp.pl?gene=RIPK1 |
| LTBP2 | Latent Transforming Growth Factor Beta Binding Protein 2 | Protein Coding | 43 | GC14M074498 | 2.1 | https://www.genecards.org/cgi-bin/carddisp.pl?gene=LTBP2 |
| NRF1 | Nuclear Respiratory Factor 1 | Protein Coding | 43 | GC07P129611 | 2.1 | https://www.genecards.org/cgi-bin/carddisp.pl?gene=NRF1 |
| C5AR1 | Complement C5a Receptor 1 | Protein Coding | 43 | GC19P047290 | 2.1 | https://www.genecards.org/cgi-bin/carddisp.pl?gene=C5AR1 |
| ACVRL1 | Activin A Receptor Like Type 1 | Protein Coding | 50 | GC12P051906 | 2.09 | https://www.genecards.org/cgi-bin/carddisp.pl?gene=ACVRL1 |
| MIR1204 | MicroRNA 1204 | RNA Gene | 13 | GC08P127795 | 2.09 | https://www.genecards.org/cgi-bin/carddisp.pl?gene=MIR1204 |
| AGPS | Alkylglycerone Phosphate Synthase | Protein Coding | 43 | GC02P177392 | 2.08 | https://www.genecards.org/cgi-bin/carddisp.pl?gene=AGPS |
| TCN1 | Transcobalamin 1 | Protein Coding | 40 | GC11M061283 | 2.08 | https://www.genecards.org/cgi-bin/carddisp.pl?gene=TCN1 |
| PLAGL1 | PLAG1 Like Zinc Finger 1 | Protein Coding | 43 | GC06M143940 | 2.08 | https://www.genecards.org/cgi-bin/carddisp.pl?gene=PLAGL1 |
| PRPF8 | Pre-MRNA Processing Factor 8 | Protein Coding | 41 | GC17M001650 | 2.08 | https://www.genecards.org/cgi-bin/carddisp.pl?gene=PRPF8 |
| LPO | Lactoperoxidase | Protein Coding | 39 | GC17P058218 | 2.08 | https://www.genecards.org/cgi-bin/carddisp.pl?gene=LPO |
| ZAP70 | Zeta Chain Of T Cell Receptor Associated Protein Kinase 70 | Protein Coding | 51 | GC02P097696 | 2.08 | https://www.genecards.org/cgi-bin/carddisp.pl?gene=ZAP70 |
| CCR9 | C-C Motif Chemokine Receptor 9 | Protein Coding | 40 | GC03P045903 | 2.07 | https://www.genecards.org/cgi-bin/carddisp.pl?gene=CCR9 |
| MIR539 | MicroRNA 539 | RNA Gene | 16 | GC14P104817 | 2.07 | https://www.genecards.org/cgi-bin/carddisp.pl?gene=MIR539 |
| CLDN2 | Claudin 2 | Protein Coding | 41 | GC0XP106900 | 2.07 | https://www.genecards.org/cgi-bin/carddisp.pl?gene=CLDN2 |
| TBP | TATA-Box Binding Protein | Protein Coding | 48 | GC06P170554 | 2.06 | https://www.genecards.org/cgi-bin/carddisp.pl?gene=TBP |
| HLA-DMA | Major Histocompatibility Complex, Class II, DM Alpha | Protein Coding | 40 | GC06M032950 | 2.06 | https://www.genecards.org/cgi-bin/carddisp.pl?gene=HLA-DMA |
| LAMP1 | Lysosomal Associated Membrane Protein 1 | Protein Coding | 43 | GC13P113297 | 2.06 | https://www.genecards.org/cgi-bin/carddisp.pl?gene=LAMP1 |
| HSBP1 | Heat Shock Factor Binding Protein 1 | Protein Coding | 34 | GC16P083807 | 2.06 | https://www.genecards.org/cgi-bin/carddisp.pl?gene=HSBP1 |
| RAC3 | Rac Family Small GTPase 3 | Protein Coding | 45 | GC17P082031 | 2.06 | https://www.genecards.org/cgi-bin/carddisp.pl?gene=RAC3 |
| ISG15 | ISG15 Ubiquitin Like Modifier | Protein Coding | 47 | GC01P001001 | 2.05 | https://www.genecards.org/cgi-bin/carddisp.pl?gene=ISG15 |
| ARNTL | Aryl Hydrocarbon Receptor Nuclear Translocator Like | Protein Coding | 42 | GC11P013276 | 2.05 | https://www.genecards.org/cgi-bin/carddisp.pl?gene=ARNTL |
| CCL8 | C-C Motif Chemokine Ligand 8 | Protein Coding | 39 | GC17P034319 | 2.05 | https://www.genecards.org/cgi-bin/carddisp.pl?gene=CCL8 |
| ADH5 | Alcohol Dehydrogenase 5 (Class III), Chi Polypeptide | Protein Coding | 45 | GC04M099070 | 2.05 | https://www.genecards.org/cgi-bin/carddisp.pl?gene=ADH5 |
| TET1 | Tet Methylcytosine Dioxygenase 1 | Protein Coding | 38 | GC10P068560 | 2.05 | https://www.genecards.org/cgi-bin/carddisp.pl?gene=TET1 |
| MIR940 | MicroRNA 940 | RNA Gene | 15 | GC16P002271 | 2.04 | https://www.genecards.org/cgi-bin/carddisp.pl?gene=MIR940 |
| VLDLR | Very Low Density Lipoprotein Receptor | Protein Coding | 50 | GC09P002611 | 2.04 | https://www.genecards.org/cgi-bin/carddisp.pl?gene=VLDLR |
| NFATC2 | Nuclear Factor Of Activated T Cells 2 | Protein Coding | 45 | GC20M051386 | 2.04 | https://www.genecards.org/cgi-bin/carddisp.pl?gene=NFATC2 |
| AGT | Angiotensinogen | Protein Coding | 49 | GC01M230702 | 2.04 | https://www.genecards.org/cgi-bin/carddisp.pl?gene=AGT |
| NTF3 | Neurotrophin 3 | Protein Coding | 43 | GC12P005432 | 2.03 | https://www.genecards.org/cgi-bin/carddisp.pl?gene=NTF3 |
| MAPKAPK2 | MAPK Activated Protein Kinase 2 | Protein Coding | 48 | GC01P206684 | 2.03 | https://www.genecards.org/cgi-bin/carddisp.pl?gene=MAPKAPK2 |
| DDX39B | DExD-Box Helicase 39B | Protein Coding | 39 | GC06M031530 | 2.03 | https://www.genecards.org/cgi-bin/carddisp.pl?gene=DDX39B |
| JUNB | JunB Proto-Oncogene, AP-1 Transcription Factor Subunit | Protein Coding | 41 | GC19P012791 | 2.03 | https://www.genecards.org/cgi-bin/carddisp.pl?gene=JUNB |
| MACROH2A1 | MacroH2A.1 Histone | Protein Coding | 35 | GC05M135334 | 2.03 | https://www.genecards.org/cgi-bin/carddisp.pl?gene=MACROH2A1 |
| CCNG2 | Cyclin G2 | Protein Coding | 36 | GC04P077158 | 2.03 | https://www.genecards.org/cgi-bin/carddisp.pl?gene=CCNG2 |
| XDH | Xanthine Dehydrogenase | Protein Coding | 47 | GC02M031294 | 2.03 | https://www.genecards.org/cgi-bin/carddisp.pl?gene=XDH |
| CTDSPL | CTD Small Phosphatase Like | Protein Coding | 40 | GC03P037861 | 2.03 | https://www.genecards.org/cgi-bin/carddisp.pl?gene=CTDSPL |
| SLC5A2 | Solute Carrier Family 5 Member 2 | Protein Coding | 47 | GC16P031580 | 2.02 | https://www.genecards.org/cgi-bin/carddisp.pl?gene=SLC5A2 |
| GLB1 | Galactosidase Beta 1 | Protein Coding | 48 | GC03M033013 | 2.02 | https://www.genecards.org/cgi-bin/carddisp.pl?gene=GLB1 |
| NID2 | Nidogen 2 | Protein Coding | 37 | GC14M052004 | 2.02 | https://www.genecards.org/cgi-bin/carddisp.pl?gene=NID2 |
| MAFB | MAF BZIP Transcription Factor B | Protein Coding | 43 | GC20M040685 | 2.01 | https://www.genecards.org/cgi-bin/carddisp.pl?gene=MAFB |
| TRIM21 | Tripartite Motif Containing 21 | Protein Coding | 42 | GC11M004384 | 2.01 | https://www.genecards.org/cgi-bin/carddisp.pl?gene=TRIM21 |
| BST2 | Bone Marrow Stromal Cell Antigen 2 | Protein Coding | 39 | GC19M017403 | 2.01 | https://www.genecards.org/cgi-bin/carddisp.pl?gene=BST2 |
| PCDH10 | Protocadherin 10 | Protein Coding | 39 | GC04P133149 | 2 | https://www.genecards.org/cgi-bin/carddisp.pl?gene=PCDH10 |
| CAPG | Capping Actin Protein, Gelsolin Like | Protein Coding | 40 | GC02M085394 | 2 | https://www.genecards.org/cgi-bin/carddisp.pl?gene=CAPG |
| CDKN2A-DT | CDKN2A Divergent Transcript | RNA Gene | 17 | GC09P021967 | 2 | https://www.genecards.org/cgi-bin/carddisp.pl?gene=CDKN2A-DT |
| CPT1A | Carnitine Palmitoyltransferase 1A | Protein Coding | 48 | GC11M068754 | 2 | https://www.genecards.org/cgi-bin/carddisp.pl?gene=CPT1A |
| KIDINS220 | Kinase D Interacting Substrate 220 | Protein Coding | 41 | GC02M008715 | 1.99 | https://www.genecards.org/cgi-bin/carddisp.pl?gene=KIDINS220 |
| OLFM4 | Olfactomedin 4 | Protein Coding | 37 | GC13P053028 | 1.99 | https://www.genecards.org/cgi-bin/carddisp.pl?gene=OLFM4 |
| ATF6B | Activating Transcription Factor 6 Beta | Protein Coding | 37 | GC06M032115 | 1.99 | https://www.genecards.org/cgi-bin/carddisp.pl?gene=ATF6B |
| SOX7 | SRY-Box Transcription Factor 7 | Protein Coding | 35 | GC08M010723 | 1.99 | https://www.genecards.org/cgi-bin/carddisp.pl?gene=SOX7 |
| C4A | Complement C4A (Rodgers Blood Group) | Protein Coding | 42 | GC06P047332 | 1.99 | https://www.genecards.org/cgi-bin/carddisp.pl?gene=C4A |
| CMA1 | Chymase 1 | Protein Coding | 43 | GC14M024506 | 1.99 | https://www.genecards.org/cgi-bin/carddisp.pl?gene=CMA1 |
| HLA-DRB5 | Major Histocompatibility Complex, Class II, DR Beta 5 | Protein Coding | 40 | GC06M032683 | 1.99 | https://www.genecards.org/cgi-bin/carddisp.pl?gene=HLA-DRB5 |
| PPP1R10 | Protein Phosphatase 1 Regulatory Subunit 10 | Protein Coding | 38 | GC06M030600 | 1.98 | https://www.genecards.org/cgi-bin/carddisp.pl?gene=PPP1R10 |
| TNFAIP8L3 | TNF Alpha Induced Protein 8 Like 3 | Protein Coding | 33 | GC15M051056 | 1.98 | https://www.genecards.org/cgi-bin/carddisp.pl?gene=TNFAIP8L3 |
| ILF3 | Interleukin Enhancer Binding Factor 3 | Protein Coding | 38 | GC19P010625 | 1.97 | https://www.genecards.org/cgi-bin/carddisp.pl?gene=ILF3 |
| TAC1 | Tachykinin Precursor 1 | Protein Coding | 43 | GC07P097731 | 1.97 | https://www.genecards.org/cgi-bin/carddisp.pl?gene=TAC1 |
| VPS52 | VPS52 Subunit Of GARP Complex | Protein Coding | 36 | GC06M033251 | 1.96 | https://www.genecards.org/cgi-bin/carddisp.pl?gene=VPS52 |
| KDM3A | Lysine Demethylase 3A | Protein Coding | 41 | GC02P086440 | 1.96 | https://www.genecards.org/cgi-bin/carddisp.pl?gene=KDM3A |
| KL | Klotho | Protein Coding | 44 | GC13P033016 | 1.96 | https://www.genecards.org/cgi-bin/carddisp.pl?gene=KL |
| CELF2 | CUGBP Elav-Like Family Member 2 | Protein Coding | 41 | GC10P010462 | 1.96 | https://www.genecards.org/cgi-bin/carddisp.pl?gene=CELF2 |
| MIR1207 | MicroRNA 1207 | RNA Gene | 14 | GC08P128049 | 1.96 | https://www.genecards.org/cgi-bin/carddisp.pl?gene=MIR1207 |
| HLA-DQB2 | Major Histocompatibility Complex, Class II, DQ Beta 2 | Protein Coding | 36 | GC06M032756 | 1.96 | https://www.genecards.org/cgi-bin/carddisp.pl?gene=HLA-DQB2 |
| GRIK1 | Glutamate Ionotropic Receptor Kainate Type Subunit 1 | Protein Coding | 45 | GC21M029536 | 1.95 | https://www.genecards.org/cgi-bin/carddisp.pl?gene=GRIK1 |
| SLC22A2 | Solute Carrier Family 22 Member 2 | Protein Coding | 44 | GC06M160173 | 1.95 | https://www.genecards.org/cgi-bin/carddisp.pl?gene=SLC22A2 |
| CRKL | CRK Like Proto-Oncogene, Adaptor Protein | Protein Coding | 46 | GC22P020917 | 1.95 | https://www.genecards.org/cgi-bin/carddisp.pl?gene=CRKL |
| MAP3K3 | Mitogen-Activated Protein Kinase Kinase Kinase 3 | Protein Coding | 47 | GC17P063622 | 1.95 | https://www.genecards.org/cgi-bin/carddisp.pl?gene=MAP3K3 |
| MGAM | Maltase-Glucoamylase | Protein Coding | 41 | GC07P145052 | 1.93 | https://www.genecards.org/cgi-bin/carddisp.pl?gene=MGAM |
| KRT6A | Keratin 6A | Protein Coding | 44 | GC12M052488 | 1.93 | https://www.genecards.org/cgi-bin/carddisp.pl?gene=KRT6A |
| TRIM26 | Tripartite Motif Containing 26 | Protein Coding | 37 | GC06M030184 | 1.93 | https://www.genecards.org/cgi-bin/carddisp.pl?gene=TRIM26 |
| N4BP2L1 | NEDD4 Binding Protein 2 Like 1 | Protein Coding | 32 | GC13M033515 | 1.93 | https://www.genecards.org/cgi-bin/carddisp.pl?gene=N4BP2L1 |
| FCGR1A | Fc Fragment Of IgG Receptor Ia | Protein Coding | 41 | GC01P149754 | 1.93 | https://www.genecards.org/cgi-bin/carddisp.pl?gene=FCGR1A |
| GPSM3 | G Protein Signaling Modulator 3 | Protein Coding | 33 | GC06M032647 | 1.93 | https://www.genecards.org/cgi-bin/carddisp.pl?gene=GPSM3 |
| HLA-DQA2 | Major Histocompatibility Complex, Class II, DQ Alpha 2 | Protein Coding | 37 | GC06P032741 | 1.93 | https://www.genecards.org/cgi-bin/carddisp.pl?gene=HLA-DQA2 |
| HLA-DRB6 | Major Histocompatibility Complex, Class II, DR Beta 6 (Pseudogene) | Pseudogene | 16 | GC06M032693 | 1.93 | https://www.genecards.org/cgi-bin/carddisp.pl?gene=HLA-DRB6 |
| CYP21A1P | Cytochrome P450 Family 21 Subfamily A Member 1, Pseudogene | Pseudogene | 15 | GC06P032005 | 1.93 | https://www.genecards.org/cgi-bin/carddisp.pl?gene=CYP21A1P |
| PBX2 | PBX Homeobox 2 | Protein Coding | 39 | GC06M032184 | 1.93 | https://www.genecards.org/cgi-bin/carddisp.pl?gene=PBX2 |
| YWHAG | Tyrosine 3-Monooxygenase/Tryptophan 5-Monooxygenase Activation Protein Gamma | Protein Coding | 49 | GC07M076327 | 1.93 | https://www.genecards.org/cgi-bin/carddisp.pl?gene=YWHAG |
| CXCR6 | C-X-C Motif Chemokine Receptor 6 | Protein Coding | 40 | GC03P045982 | 1.92 | https://www.genecards.org/cgi-bin/carddisp.pl?gene=CXCR6 |
| SLCO1B1 | Solute Carrier Organic Anion Transporter Family Member 1B1 | Protein Coding | 48 | GC12P021132 | 1.92 | https://www.genecards.org/cgi-bin/carddisp.pl?gene=SLCO1B1 |
| RBPJ | Recombination Signal Binding Protein For Immunoglobulin Kappa J Region | Protein Coding | 47 | GC04P026165 | 1.92 | https://www.genecards.org/cgi-bin/carddisp.pl?gene=RBPJ |
| P2RY2 | Purinergic Receptor P2Y2 | Protein Coding | 45 | GC11P073217 | 1.91 | https://www.genecards.org/cgi-bin/carddisp.pl?gene=P2RY2 |
| TCF19 | Transcription Factor 19 | Protein Coding | 36 | GC06P047294 | 1.91 | https://www.genecards.org/cgi-bin/carddisp.pl?gene=TCF19 |
| CDHR3 | Cadherin Related Family Member 3 | Protein Coding | 36 | GC07P105876 | 1.91 | https://www.genecards.org/cgi-bin/carddisp.pl?gene=CDHR3 |
| ABCF1 | ATP Binding Cassette Subfamily F Member 1 | Protein Coding | 39 | GC06P030571 | 1.91 | https://www.genecards.org/cgi-bin/carddisp.pl?gene=ABCF1 |
| PTGDR2 | Prostaglandin D2 Receptor 2 | Protein Coding | 41 | GC11M060850 | 1.91 | https://www.genecards.org/cgi-bin/carddisp.pl?gene=PTGDR2 |
| RIN1 | Ras And Rab Interactor 1 | Protein Coding | 39 | GC11M066335 | 1.9 | https://www.genecards.org/cgi-bin/carddisp.pl?gene=RIN1 |
| CCL25 | C-C Motif Chemokine Ligand 25 | Protein Coding | 39 | GC19P008117 | 1.9 | https://www.genecards.org/cgi-bin/carddisp.pl?gene=CCL25 |
| CFH | Complement Factor H | Protein Coding | 45 | GC01P196621 | 1.9 | https://www.genecards.org/cgi-bin/carddisp.pl?gene=CFH |
| TMBIM6 | Transmembrane BAX Inhibitor Motif Containing 6 | Protein Coding | 36 | GC12P049707 | 1.9 | https://www.genecards.org/cgi-bin/carddisp.pl?gene=TMBIM6 |
| RAB14 | RAB14, Member RAS Oncogene Family | Protein Coding | 40 | GC09M121178 | 1.9 | https://www.genecards.org/cgi-bin/carddisp.pl?gene=RAB14 |
| ADAMTS9 | ADAM Metallopeptidase With Thrombospondin Type 1 Motif 9 | Protein Coding | 37 | GC03M064501 | 1.89 | https://www.genecards.org/cgi-bin/carddisp.pl?gene=ADAMTS9 |
| FNDC3B | Fibronectin Type III Domain Containing 3B | Protein Coding | 36 | GC03P172039 | 1.89 | https://www.genecards.org/cgi-bin/carddisp.pl?gene=FNDC3B |
| MIR449B | MicroRNA 449b | RNA Gene | 16 | GC05M055172 | 1.89 | https://www.genecards.org/cgi-bin/carddisp.pl?gene=MIR449B |
| KDM4A | Lysine Demethylase 4A | Protein Coding | 41 | GC01P043650 | 1.89 | https://www.genecards.org/cgi-bin/carddisp.pl?gene=KDM4A |
| SDC2 | Syndecan 2 | Protein Coding | 45 | GC08P096494 | 1.88 | https://www.genecards.org/cgi-bin/carddisp.pl?gene=SDC2 |
| FDFT1 | Farnesyl-Diphosphate Farnesyltransferase 1 | Protein Coding | 44 | GC08P011795 | 1.88 | https://www.genecards.org/cgi-bin/carddisp.pl?gene=FDFT1 |
| RNF8 | Ring Finger Protein 8 | Protein Coding | 41 | GC06P047472 | 1.88 | https://www.genecards.org/cgi-bin/carddisp.pl?gene=RNF8 |
| CCHCR1 | Coiled-Coil Alpha-Helical Rod Protein 1 | Protein Coding | 38 | GC06M031145 | 1.88 | https://www.genecards.org/cgi-bin/carddisp.pl?gene=CCHCR1 |
| GALNT7 | Polypeptide N-Acetylgalactosaminyltransferase 7 | Protein Coding | 38 | GC04P173168 | 1.88 | https://www.genecards.org/cgi-bin/carddisp.pl?gene=GALNT7 |
| TIGAR | TP53 Induced Glycolysis Regulatory Phosphatase | Protein Coding | 38 | GC12P008104 | 1.88 | https://www.genecards.org/cgi-bin/carddisp.pl?gene=TIGAR |
| KLF12 | Kruppel Like Factor 12 | Protein Coding | 38 | GC13M073686 | 1.87 | https://www.genecards.org/cgi-bin/carddisp.pl?gene=KLF12 |
| GNLY | Granulysin | Protein Coding | 38 | GC02P085685 | 1.87 | https://www.genecards.org/cgi-bin/carddisp.pl?gene=GNLY |
| CACNA2D3 | Calcium Voltage-Gated Channel Auxiliary Subunit Alpha2delta 3 | Protein Coding | 40 | GC03P054156 | 1.87 | https://www.genecards.org/cgi-bin/carddisp.pl?gene=CACNA2D3 |
| IRF6 | Interferon Regulatory Factor 6 | Protein Coding | 43 | GC01M209785 | 1.87 | https://www.genecards.org/cgi-bin/carddisp.pl?gene=IRF6 |
| HLA-DMB | Major Histocompatibility Complex, Class II, DM Beta | Protein Coding | 41 | GC06M032934 | 1.86 | https://www.genecards.org/cgi-bin/carddisp.pl?gene=HLA-DMB |
| HLA-DOA | Major Histocompatibility Complex, Class II, DO Alpha | Protein Coding | 40 | GC06M033004 | 1.86 | https://www.genecards.org/cgi-bin/carddisp.pl?gene=HLA-DOA |
| HLA-DOB | Major Histocompatibility Complex, Class II, DO Beta | Protein Coding | 40 | GC06M032823 | 1.86 | https://www.genecards.org/cgi-bin/carddisp.pl?gene=HLA-DOB |
| HLA-DQB1-AS1 | HLA-DQB1 Antisense RNA 1 | RNA Gene | 12 | GC06P032659 | 1.86 | https://www.genecards.org/cgi-bin/carddisp.pl?gene=HLA-DQB1-AS1 |
| ENSG00000264545 |  | Protein Coding | 10 | GC09P021802 | 1.86 | https://www.genecards.org/cgi-bin/carddisp.pl?gene=ENSG00000264545 |
| PTOV1 | PTOV1 Extended AT-Hook Containing Adaptor Protein | Protein Coding | 36 | GC19P049850 | 1.86 | https://www.genecards.org/cgi-bin/carddisp.pl?gene=PTOV1 |
| NLK | Nemo Like Kinase | Protein Coding | 43 | GC17P028042 | 1.86 | https://www.genecards.org/cgi-bin/carddisp.pl?gene=NLK |
| MIR384 | MicroRNA 384 | RNA Gene | 9 | GC0XM076919 | 1.86 | https://www.genecards.org/cgi-bin/carddisp.pl?gene=MIR384 |
| HK1 | Hexokinase 1 | Protein Coding | 51 | GC10P069269 | 1.86 | https://www.genecards.org/cgi-bin/carddisp.pl?gene=HK1 |
| HAX1 | HCLS1 Associated Protein X-1 | Protein Coding | 43 | GC01P154273 | 1.85 | https://www.genecards.org/cgi-bin/carddisp.pl?gene=HAX1 |
| LCP1 | Lymphocyte Cytosolic Protein 1 | Protein Coding | 41 | GC13M046132 | 1.85 | https://www.genecards.org/cgi-bin/carddisp.pl?gene=LCP1 |
| TEX101 | Testis Expressed 101 | Protein Coding | 35 | GC19P043388 | 1.84 | https://www.genecards.org/cgi-bin/carddisp.pl?gene=TEX101 |
| APLNR | Apelin Receptor | Protein Coding | 43 | GC11M057233 | 1.84 | https://www.genecards.org/cgi-bin/carddisp.pl?gene=APLNR |
| MIR421 | MicroRNA 421 | RNA Gene | 13 | GC0XM074218 | 1.84 | https://www.genecards.org/cgi-bin/carddisp.pl?gene=MIR421 |
| PSORS1C1 | Psoriasis Susceptibility 1 Candidate 1 | Protein Coding | 28 | GC06P031114 | 1.84 | https://www.genecards.org/cgi-bin/carddisp.pl?gene=PSORS1C1 |
| EHMT2 | Euchromatic Histone Lysine Methyltransferase 2 | Protein Coding | 45 | GC06M031879 | 1.83 | https://www.genecards.org/cgi-bin/carddisp.pl?gene=EHMT2 |
| SLC5A4 | Solute Carrier Family 5 Member 4 | Protein Coding | 38 | GC22M032218 | 1.83 | https://www.genecards.org/cgi-bin/carddisp.pl?gene=SLC5A4 |
| CREM | CAMP Responsive Element Modulator | Protein Coding | 41 | GC10P035126 | 1.82 | https://www.genecards.org/cgi-bin/carddisp.pl?gene=CREM |
| MIR20B | MicroRNA 20b | RNA Gene | 15 | GC0XM134217 | 1.81 | https://www.genecards.org/cgi-bin/carddisp.pl?gene=MIR20B |
| TP53INP1 | Tumor Protein P53 Inducible Nuclear Protein 1 | Protein Coding | 38 | GC08M094925 | 1.81 | https://www.genecards.org/cgi-bin/carddisp.pl?gene=TP53INP1 |
| F11R | F11 Receptor | Protein Coding | 43 | GC01M160995 | 1.8 | https://www.genecards.org/cgi-bin/carddisp.pl?gene=F11R |
| PSORS1C2 | Psoriasis Susceptibility 1 Candidate 2 | Protein Coding | 31 | GC06M031137 | 1.8 | https://www.genecards.org/cgi-bin/carddisp.pl?gene=PSORS1C2 |
| HCG22 | HLA Complex Group 22 | Protein Coding | 20 | GC06P031053 | 1.8 | https://www.genecards.org/cgi-bin/carddisp.pl?gene=HCG22 |
| HLA-J | Major Histocompatibility Complex, Class I, J (Pseudogene) | Pseudogene | 17 | GC06P047267 | 1.8 | https://www.genecards.org/cgi-bin/carddisp.pl?gene=HLA-J |
| ENSG00000272221 |  | RNA Gene | 6 | GC06M032544 | 1.8 | https://www.genecards.org/cgi-bin/carddisp.pl?gene=ENSG00000272221 |
| MN298114-200 |  | RNA Gene | 4 | GC06M031041 | 1.8 | https://www.genecards.org/cgi-bin/carddisp.pl?gene=MN298114-200 |
| PCDH20 | Protocadherin 20 | Protein Coding | 33 | GC13M061409 | 1.8 | https://www.genecards.org/cgi-bin/carddisp.pl?gene=PCDH20 |
| FEZF2 | FEZ Family Zinc Finger 2 | Protein Coding | 36 | GC03M062355 | 1.8 | https://www.genecards.org/cgi-bin/carddisp.pl?gene=FEZF2 |
| MT1E | Metallothionein 1E | Protein Coding | 39 | GC16P056625 | 1.8 | https://www.genecards.org/cgi-bin/carddisp.pl?gene=MT1E |
| NEDD8 | NEDD8 Ubiquitin Like Modifier | Protein Coding | 41 | GC14M024216 | 1.79 | https://www.genecards.org/cgi-bin/carddisp.pl?gene=NEDD8 |
| TAS2R38 | Taste 2 Receptor Member 38 | Protein Coding | 36 | GC07M141972 | 1.79 | https://www.genecards.org/cgi-bin/carddisp.pl?gene=TAS2R38 |
| DAPK2 | Death Associated Protein Kinase 2 | Protein Coding | 41 | GC15M063907 | 1.79 | https://www.genecards.org/cgi-bin/carddisp.pl?gene=DAPK2 |
| ACKR2 | Atypical Chemokine Receptor 2 | Protein Coding | 37 | GC03P042804 | 1.79 | https://www.genecards.org/cgi-bin/carddisp.pl?gene=ACKR2 |
| EWSAT1 | Ewing Sarcoma Associated Transcript 1 | RNA Gene | 18 | GC15P072889 | 1.79 | https://www.genecards.org/cgi-bin/carddisp.pl?gene=EWSAT1 |
| NCAPG | Non-SMC Condensin I Complex Subunit G | Protein Coding | 39 | GC04P017812 | 1.79 | https://www.genecards.org/cgi-bin/carddisp.pl?gene=NCAPG |
| TES | Testin LIM Domain Protein | Protein Coding | 41 | GC07P116210 | 1.78 | https://www.genecards.org/cgi-bin/carddisp.pl?gene=TES |
| IGKC | Immunoglobulin Kappa Constant | Protein Coding | 32 | GC02M089142 | 1.78 | https://www.genecards.org/cgi-bin/carddisp.pl?gene=IGKC |
| GJC1 | Gap Junction Protein Gamma 1 | Protein Coding | 42 | GC17M044800 | 1.78 | https://www.genecards.org/cgi-bin/carddisp.pl?gene=GJC1 |
| PGAP3 | Post-GPI Attachment To Proteins Phospholipase 3 | Protein Coding | 38 | GC17M039676 | 1.78 | https://www.genecards.org/cgi-bin/carddisp.pl?gene=PGAP3 |
| AIM2 | Absent In Melanoma 2 | Protein Coding | 41 | GC01M159062 | 1.77 | https://www.genecards.org/cgi-bin/carddisp.pl?gene=AIM2 |
| HOXC6 | Homeobox C6 | Protein Coding | 37 | GC12P053990 | 1.77 | https://www.genecards.org/cgi-bin/carddisp.pl?gene=HOXC6 |
| ILF2 | Interleukin Enhancer Binding Factor 2 | Protein Coding | 38 | GC01M153661 | 1.77 | https://www.genecards.org/cgi-bin/carddisp.pl?gene=ILF2 |
| SIVA1 | SIVA1 Apoptosis Inducing Factor | Protein Coding | 36 | GC14P104853 | 1.77 | https://www.genecards.org/cgi-bin/carddisp.pl?gene=SIVA1 |
| INHBB | Inhibin Subunit Beta B | Protein Coding | 42 | GC02P120349 | 1.77 | https://www.genecards.org/cgi-bin/carddisp.pl?gene=INHBB |
| IL23R | Interleukin 23 Receptor | Protein Coding | 44 | GC01P067138 | 1.77 | https://www.genecards.org/cgi-bin/carddisp.pl?gene=IL23R |
| GAS7 | Growth Arrest Specific 7 | Protein Coding | 40 | GC17M009910 | 1.76 | https://www.genecards.org/cgi-bin/carddisp.pl?gene=GAS7 |
| CASC22 | Cancer Susceptibility 22 | RNA Gene | 13 | GC16P052258 | 1.76 | https://www.genecards.org/cgi-bin/carddisp.pl?gene=CASC22 |
| IL27 | Interleukin 27 | Protein Coding | 38 | GC16M028511 | 1.76 | https://www.genecards.org/cgi-bin/carddisp.pl?gene=IL27 |
| BLZF1 | Basic Leucine Zipper Nuclear Factor 1 | Protein Coding | 39 | GC01P169367 | 1.76 | https://www.genecards.org/cgi-bin/carddisp.pl?gene=BLZF1 |
| SUCLG2 | Succinate-CoA Ligase GDP-Forming Subunit Beta | Protein Coding | 41 | GC03M067358 | 1.75 | https://www.genecards.org/cgi-bin/carddisp.pl?gene=SUCLG2 |
| MIR504 | MicroRNA 504 | RNA Gene | 13 | GC0XM138667 | 1.75 | https://www.genecards.org/cgi-bin/carddisp.pl?gene=MIR504 |
| UBR5 | Ubiquitin Protein Ligase E3 Component N-Recognin 5 | Protein Coding | 42 | GC08M102252 | 1.75 | https://www.genecards.org/cgi-bin/carddisp.pl?gene=UBR5 |
| CAST | Calpastatin | Protein Coding | 45 | GC05P096525 | 1.75 | https://www.genecards.org/cgi-bin/carddisp.pl?gene=CAST |
| CYP2A13 | Cytochrome P450 Family 2 Subfamily A Member 13 | Protein Coding | 41 | GC19P041088 | 1.74 | https://www.genecards.org/cgi-bin/carddisp.pl?gene=CYP2A13 |
| PCDH8 | Protocadherin 8 | Protein Coding | 39 | GC13M052842 | 1.74 | https://www.genecards.org/cgi-bin/carddisp.pl?gene=PCDH8 |
| ANAPC1 | Anaphase Promoting Complex Subunit 1 | Protein Coding | 40 | GC02M111611 | 1.74 | https://www.genecards.org/cgi-bin/carddisp.pl?gene=ANAPC1 |
| PIM3 | Pim-3 Proto-Oncogene, Serine/Threonine Kinase | Protein Coding | 40 | GC22P049960 | 1.73 | https://www.genecards.org/cgi-bin/carddisp.pl?gene=PIM3 |
| DHX16 | DEAH-Box Helicase 16 | Protein Coding | 40 | GC06M030653 | 1.73 | https://www.genecards.org/cgi-bin/carddisp.pl?gene=DHX16 |
| NFKBIL1 | NFKB Inhibitor Like 1 | Protein Coding | 37 | GC06P047302 | 1.73 | https://www.genecards.org/cgi-bin/carddisp.pl?gene=NFKBIL1 |
| NRM | Nurim | Protein Coding | 32 | GC06M030895 | 1.73 | https://www.genecards.org/cgi-bin/carddisp.pl?gene=NRM |
| LY6G5B | Lymphocyte Antigen 6 Family Member G5B | Protein Coding | 29 | GC06P047314 | 1.73 | https://www.genecards.org/cgi-bin/carddisp.pl?gene=LY6G5B |
| HCG4 | HLA Complex Group 4 | RNA Gene | 17 | GC06M030855 | 1.73 | https://www.genecards.org/cgi-bin/carddisp.pl?gene=HCG4 |
| HCG23 | HLA Complex Group 23 | RNA Gene | 14 | GC06P047338 | 1.73 | https://www.genecards.org/cgi-bin/carddisp.pl?gene=HCG23 |
| RPL3P2 | Ribosomal Protein L3 Pseudogene 2 | Pseudogene | 8 | GC06P031280 | 1.73 | https://www.genecards.org/cgi-bin/carddisp.pl?gene=RPL3P2 |
| ENSG00000272501 |  | RNA Gene | 8 | GC06M031195 | 1.73 | https://www.genecards.org/cgi-bin/carddisp.pl?gene=ENSG00000272501 |
| ENSG00000271581 |  | Pseudogene | 5 | GC06P047295 | 1.73 | https://www.genecards.org/cgi-bin/carddisp.pl?gene=ENSG00000271581 |
| RASSF6 | Ras Association Domain Family Member 6 | Protein Coding | 38 | GC04M073571 | 1.73 | https://www.genecards.org/cgi-bin/carddisp.pl?gene=RASSF6 |
| PCDH17 | Protocadherin 17 | Protein Coding | 36 | GC13P057630 | 1.72 | https://www.genecards.org/cgi-bin/carddisp.pl?gene=PCDH17 |
| TUBB4B | Tubulin Beta 4B Class IVb | Protein Coding | 44 | GC09P137241 | 1.72 | https://www.genecards.org/cgi-bin/carddisp.pl?gene=TUBB4B |
| TPST1 | Tyrosylprotein Sulfotransferase 1 | Protein Coding | 40 | GC07P066206 | 1.72 | https://www.genecards.org/cgi-bin/carddisp.pl?gene=TPST1 |
| UGT2B17 | UDP Glucuronosyltransferase Family 2 Member B17 | Protein Coding | 40 | GC04M068537 | 1.71 | https://www.genecards.org/cgi-bin/carddisp.pl?gene=UGT2B17 |
| NR4A2 | Nuclear Receptor Subfamily 4 Group A Member 2 | Protein Coding | 46 | GC02M156324 | 1.71 | https://www.genecards.org/cgi-bin/carddisp.pl?gene=NR4A2 |
| IRF8 | Interferon Regulatory Factor 8 | Protein Coding | 45 | GC16P085898 | 1.71 | https://www.genecards.org/cgi-bin/carddisp.pl?gene=IRF8 |
| PLAC8 | Placenta Associated 8 | Protein Coding | 37 | GC04M083090 | 1.71 | https://www.genecards.org/cgi-bin/carddisp.pl?gene=PLAC8 |
| CD79A | CD79a Molecule | Protein Coding | 46 | GC19P041877 | 1.71 | https://www.genecards.org/cgi-bin/carddisp.pl?gene=CD79A |
| CDK3 | Cyclin Dependent Kinase 3 | Protein Coding | 40 | GC17P076007 | 1.7 | https://www.genecards.org/cgi-bin/carddisp.pl?gene=CDK3 |
| CYP2B6 | Cytochrome P450 Family 2 Subfamily B Member 6 | Protein Coding | 47 | GC19P040991 | 1.7 | https://www.genecards.org/cgi-bin/carddisp.pl?gene=CYP2B6 |
| LSM2 | LSM2 Homolog, U6 Small Nuclear RNA And MRNA Degradation Associated | Protein Coding | 40 | GC06M032632 | 1.7 | https://www.genecards.org/cgi-bin/carddisp.pl?gene=LSM2 |
| NCR3 | Natural Cytotoxicity Triggering Receptor 3 | Protein Coding | 39 | GC06M031588 | 1.7 | https://www.genecards.org/cgi-bin/carddisp.pl?gene=NCR3 |
| GNAT1 | G Protein Subunit Alpha Transducin 1 | Protein Coding | 46 | GC03P050194 | 1.69 | https://www.genecards.org/cgi-bin/carddisp.pl?gene=GNAT1 |
| BACH2 | BTB Domain And CNC Homolog 2 | Protein Coding | 40 | GC06M089926 | 1.69 | https://www.genecards.org/cgi-bin/carddisp.pl?gene=BACH2 |
| SSRP1 | Structure Specific Recognition Protein 1 | Protein Coding | 40 | GC11M061223 | 1.69 | https://www.genecards.org/cgi-bin/carddisp.pl?gene=SSRP1 |
| ABCC6 | ATP Binding Cassette Subfamily C Member 6 | Protein Coding | 45 | GC16M016148 | 1.69 | https://www.genecards.org/cgi-bin/carddisp.pl?gene=ABCC6 |
| PRR4 | Proline Rich 4 | Protein Coding | 33 | GC12M013881 | 1.69 | https://www.genecards.org/cgi-bin/carddisp.pl?gene=PRR4 |
| STIL | STIL Centriolar Assembly Protein | Protein Coding | 41 | GC01M047250 | 1.69 | https://www.genecards.org/cgi-bin/carddisp.pl?gene=STIL |
| CYBA | Cytochrome B-245 Alpha Chain | Protein Coding | 47 | GC16M088643 | 1.69 | https://www.genecards.org/cgi-bin/carddisp.pl?gene=CYBA |
| TRIM39 | Tripartite Motif Containing 39 | Protein Coding | 37 | GC06P047275 | 1.68 | https://www.genecards.org/cgi-bin/carddisp.pl?gene=TRIM39 |
| CD93 | CD93 Molecule | Protein Coding | 40 | GC20M023079 | 1.68 | https://www.genecards.org/cgi-bin/carddisp.pl?gene=CD93 |
| INPP4B | Inositol Polyphosphate-4-Phosphatase Type II B | Protein Coding | 39 | GC04M142023 | 1.67 | https://www.genecards.org/cgi-bin/carddisp.pl?gene=INPP4B |
| NFYA | Nuclear Transcription Factor Y Subunit Alpha | Protein Coding | 39 | GC06P047495 | 1.67 | https://www.genecards.org/cgi-bin/carddisp.pl?gene=NFYA |
| MGLL | Monoglyceride Lipase | Protein Coding | 45 | GC03M127689 | 1.67 | https://www.genecards.org/cgi-bin/carddisp.pl?gene=MGLL |
| LRRC4 | Leucine Rich Repeat Containing 4 | Protein Coding | 38 | GC07M128027 | 1.66 | https://www.genecards.org/cgi-bin/carddisp.pl?gene=LRRC4 |
| HSPA1L | Heat Shock Protein Family A (Hsp70) Member 1 Like | Protein Coding | 43 | GC06M031809 | 1.66 | https://www.genecards.org/cgi-bin/carddisp.pl?gene=HSPA1L |
| S1PR3 | Sphingosine-1-Phosphate Receptor 3 | Protein Coding | 44 | GC09P088991 | 1.66 | https://www.genecards.org/cgi-bin/carddisp.pl?gene=S1PR3 |
| MIR548C | MicroRNA 548c | RNA Gene | 13 | GC12P064622 | 1.65 | https://www.genecards.org/cgi-bin/carddisp.pl?gene=MIR548C |
| CYP3A7 | Cytochrome P450 Family 3 Subfamily A Member 7 | Protein Coding | 43 | GC07M099705 | 1.65 | https://www.genecards.org/cgi-bin/carddisp.pl?gene=CYP3A7 |
| AGPAT1 | 1-Acylglycerol-3-Phosphate O-Acyltransferase 1 | Protein Coding | 41 | GC06M032168 | 1.65 | https://www.genecards.org/cgi-bin/carddisp.pl?gene=AGPAT1 |
| C6orf47 | Chromosome 6 Open Reading Frame 47 | Protein Coding | 29 | GC06M032610 | 1.65 | https://www.genecards.org/cgi-bin/carddisp.pl?gene=C6orf47 |
| PSORS1C3 | Psoriasis Susceptibility 1 Candidate 3 | RNA Gene | 21 | GC06M031179 | 1.65 | https://www.genecards.org/cgi-bin/carddisp.pl?gene=PSORS1C3 |
| HLA-W | Major Histocompatibility Complex, Class I, W (Pseudogene) | Pseudogene | 9 | GC06P047261 | 1.65 | https://www.genecards.org/cgi-bin/carddisp.pl?gene=HLA-W |
| HLA-U | Major Histocompatibility Complex, Class I, U (Pseudogene) | Pseudogene | 8 | GC06P047264 | 1.65 | https://www.genecards.org/cgi-bin/carddisp.pl?gene=HLA-U |
| STK19B | Serine/Threonine Kinase 19B (Pseudogene) | Pseudogene | 8 | GC06P032013 | 1.65 | https://www.genecards.org/cgi-bin/carddisp.pl?gene=STK19B |
| ENSG00000272540 |  | RNA Gene | 6 | GC06M030903 | 1.65 | https://www.genecards.org/cgi-bin/carddisp.pl?gene=ENSG00000272540 |
| lnc-HLA-DRB1-8 |  | RNA Gene | 4 | GC06M032619 | 1.65 | https://www.genecards.org/cgi-bin/carddisp.pl?gene=lnc-HLA-DRB1-8 |
| piR-33614-217 |  | RNA Gene | 3 | GC05P001309 | 1.65 | https://www.genecards.org/cgi-bin/carddisp.pl?gene=piR-33614-217 |
| SPINK6 | Serine Peptidase Inhibitor Kazal Type 6 | Protein Coding | 31 | GC05P148202 | 1.65 | https://www.genecards.org/cgi-bin/carddisp.pl?gene=SPINK6 |
| RPS6KA5 | Ribosomal Protein S6 Kinase A5 | Protein Coding | 45 | GC14M090847 | 1.65 | https://www.genecards.org/cgi-bin/carddisp.pl?gene=RPS6KA5 |
| RAB37 | RAB37, Member RAS Oncogene Family | Protein Coding | 37 | GC17P074671 | 1.64 | https://www.genecards.org/cgi-bin/carddisp.pl?gene=RAB37 |
| MKNK1 | MAPK Interacting Serine/Threonine Kinase 1 | Protein Coding | 45 | GC01M046557 | 1.63 | https://www.genecards.org/cgi-bin/carddisp.pl?gene=MKNK1 |
| EIF3I | Eukaryotic Translation Initiation Factor 3 Subunit I | Protein Coding | 39 | GC01P032221 | 1.63 | https://www.genecards.org/cgi-bin/carddisp.pl?gene=EIF3I |
| PDHB | Pyruvate Dehydrogenase E1 Subunit Beta | Protein Coding | 45 | GC03M058428 | 1.63 | https://www.genecards.org/cgi-bin/carddisp.pl?gene=PDHB |
| CARD8 | Caspase Recruitment Domain Family Member 8 | Protein Coding | 39 | GC19M048183 | 1.62 | https://www.genecards.org/cgi-bin/carddisp.pl?gene=CARD8 |
| IRF7 | Interferon Regulatory Factor 7 | Protein Coding | 46 | GC11M000612 | 1.62 | https://www.genecards.org/cgi-bin/carddisp.pl?gene=IRF7 |
| MT-ND2 | Mitochondrially Encoded NADH:Ubiquinone Oxidoreductase Core Subunit 2 | Protein Coding | 32 | GCMTP004472 | 1.62 | https://www.genecards.org/cgi-bin/carddisp.pl?gene=MT-ND2 |
| MT-ND1 | Mitochondrially Encoded NADH:Ubiquinone Oxidoreductase Core Subunit 1 | Protein Coding | 32 | GCMTP003309 | 1.62 | https://www.genecards.org/cgi-bin/carddisp.pl?gene=MT-ND1 |
| CAMP | Cathelicidin Antimicrobial Peptide | Protein Coding | 41 | GC03P048266 | 1.62 | https://www.genecards.org/cgi-bin/carddisp.pl?gene=CAMP |
| SKIV2L | Ski2 Like RNA Helicase | Protein Coding | 43 | GC06P047331 | 1.61 | https://www.genecards.org/cgi-bin/carddisp.pl?gene=SKIV2L |
| MUCL3 | Mucin Like 3 | Protein Coding | 26 | GC06P047945 | 1.61 | https://www.genecards.org/cgi-bin/carddisp.pl?gene=MUCL3 |
| HOXC8 | Homeobox C8 | Protein Coding | 37 | GC12P054198 | 1.61 | https://www.genecards.org/cgi-bin/carddisp.pl?gene=HOXC8 |
| KMT2A | Lysine Methyltransferase 2A | Protein Coding | 44 | GC11P118436 | 1.61 | https://www.genecards.org/cgi-bin/carddisp.pl?gene=KMT2A |
| FBLN2 | Fibulin 2 | Protein Coding | 43 | GC03P013565 | 1.61 | https://www.genecards.org/cgi-bin/carddisp.pl?gene=FBLN2 |
| MYOM2 | Myomesin 2 | Protein Coding | 39 | GC08P002045 | 1.6 | https://www.genecards.org/cgi-bin/carddisp.pl?gene=MYOM2 |
| SLC19A1 | Solute Carrier Family 19 Member 1 | Protein Coding | 45 | GC21M045493 | 1.6 | https://www.genecards.org/cgi-bin/carddisp.pl?gene=SLC19A1 |
| CYC1 | Cytochrome C1 | Protein Coding | 45 | GC08P144095 | 1.59 | https://www.genecards.org/cgi-bin/carddisp.pl?gene=CYC1 |
| FLAD1 | Flavin Adenine Dinucleotide Synthetase 1 | Protein Coding | 41 | GC01P154983 | 1.59 | https://www.genecards.org/cgi-bin/carddisp.pl?gene=FLAD1 |
| LRRFIP2 | LRR Binding FLII Interacting Protein 2 | Protein Coding | 38 | GC03M037052 | 1.59 | https://www.genecards.org/cgi-bin/carddisp.pl?gene=LRRFIP2 |
| FAM225A | Family With Sequence Similarity 225 Member A | RNA Gene | 13 | GC09P113112 | 1.59 | https://www.genecards.org/cgi-bin/carddisp.pl?gene=FAM225A |
| POLR2L | RNA Polymerase II, I And III Subunit L | Protein Coding | 41 | GC11M000829 | 1.58 | https://www.genecards.org/cgi-bin/carddisp.pl?gene=POLR2L |
| DGCR8 | DGCR8 Microprocessor Complex Subunit | Protein Coding | 40 | GC22P020080 | 1.58 | https://www.genecards.org/cgi-bin/carddisp.pl?gene=DGCR8 |
| EGFL6 | EGF Like Domain Multiple 6 | Protein Coding | 38 | GC0XP013569 | 1.58 | https://www.genecards.org/cgi-bin/carddisp.pl?gene=EGFL6 |
| H3-4 | H3.4 Histone | Protein Coding | 32 | GC01M228427 | 1.58 | https://www.genecards.org/cgi-bin/carddisp.pl?gene=H3-4 |
| CFB | Complement Factor B | Protein Coding | 45 | GC06P031945 | 1.57 | https://www.genecards.org/cgi-bin/carddisp.pl?gene=CFB |
| SPAG7 | Sperm Associated Antigen 7 | Protein Coding | 34 | GC17M004959 | 1.57 | https://www.genecards.org/cgi-bin/carddisp.pl?gene=SPAG7 |
| HCG27 | HLA Complex Group 27 | RNA Gene | 21 | GC06P031197 | 1.57 | https://www.genecards.org/cgi-bin/carddisp.pl?gene=HCG27 |
| HCG18 | HLA Complex Group 18 | RNA Gene | 16 | GC06M030874 | 1.57 | https://www.genecards.org/cgi-bin/carddisp.pl?gene=HCG18 |
| SNORD117 | Small Nucleolar RNA, C/D Box 117 | RNA Gene | 16 | GC06M032581 | 1.57 | https://www.genecards.org/cgi-bin/carddisp.pl?gene=SNORD117 |
| lnc-TERT-2 |  | RNA Gene | 3 | GC05M001321 | 1.57 | https://www.genecards.org/cgi-bin/carddisp.pl?gene=lnc-TERT-2 |
| MIR634 | MicroRNA 634 | RNA Gene | 13 | GC17P066787 | 1.56 | https://www.genecards.org/cgi-bin/carddisp.pl?gene=MIR634 |
| C6orf15 | Chromosome 6 Open Reading Frame 15 | Protein Coding | 33 | GC06M031111 | 1.56 | https://www.genecards.org/cgi-bin/carddisp.pl?gene=C6orf15 |
| CBY1 | Chibby Family Member 1, Beta Catenin Antagonist | Protein Coding | 40 | GC22P038656 | 1.56 | https://www.genecards.org/cgi-bin/carddisp.pl?gene=CBY1 |
| USP39 | Ubiquitin Specific Peptidase 39 | Protein Coding | 38 | GC02P085637 | 1.55 | https://www.genecards.org/cgi-bin/carddisp.pl?gene=USP39 |
| ME1 | Malic Enzyme 1 | Protein Coding | 42 | GC06M083210 | 1.55 | https://www.genecards.org/cgi-bin/carddisp.pl?gene=ME1 |
| LTBR | Lymphotoxin Beta Receptor | Protein Coding | 41 | GC12P006375 | 1.55 | https://www.genecards.org/cgi-bin/carddisp.pl?gene=LTBR |
| WDR5 | WD Repeat Domain 5 | Protein Coding | 44 | GC09P134135 | 1.55 | https://www.genecards.org/cgi-bin/carddisp.pl?gene=WDR5 |
| MIR3147 | MicroRNA 3147 | RNA Gene | 11 | GC07P057908 | 1.55 | https://www.genecards.org/cgi-bin/carddisp.pl?gene=MIR3147 |
| TNFRSF18 | TNF Receptor Superfamily Member 18 | Protein Coding | 42 | GC01M001203 | 1.55 | https://www.genecards.org/cgi-bin/carddisp.pl?gene=TNFRSF18 |
| CYP2J2 | Cytochrome P450 Family 2 Subfamily J Member 2 | Protein Coding | 44 | GC01M059893 | 1.55 | https://www.genecards.org/cgi-bin/carddisp.pl?gene=CYP2J2 |
| RAET1E | Retinoic Acid Early Transcript 1E | Protein Coding | 38 | GC06M149883 | 1.55 | https://www.genecards.org/cgi-bin/carddisp.pl?gene=RAET1E |
| NOL3 | Nucleolar Protein 3 | Protein Coding | 43 | GC16P067174 | 1.54 | https://www.genecards.org/cgi-bin/carddisp.pl?gene=NOL3 |
| LZTS2 | Leucine Zipper Tumor Suppressor 2 | Protein Coding | 35 | GC10P100996 | 1.54 | https://www.genecards.org/cgi-bin/carddisp.pl?gene=LZTS2 |
| BRCC3 | BRCA1/BRCA2-Containing Complex Subunit 3 | Protein Coding | 43 | GC0XP155071 | 1.54 | https://www.genecards.org/cgi-bin/carddisp.pl?gene=BRCC3 |
| HPSE2 | Heparanase 2 (Inactive) | Protein Coding | 41 | GC10M098457 | 1.53 | https://www.genecards.org/cgi-bin/carddisp.pl?gene=HPSE2 |
| NFKBIB | NFKB Inhibitor Beta | Protein Coding | 41 | GC19P038899 | 1.53 | https://www.genecards.org/cgi-bin/carddisp.pl?gene=NFKBIB |
| GLRX3 | Glutaredoxin 3 | Protein Coding | 40 | GC10P130136 | 1.53 | https://www.genecards.org/cgi-bin/carddisp.pl?gene=GLRX3 |
| UBE2L6 | Ubiquitin Conjugating Enzyme E2 L6 | Protein Coding | 40 | GC11M061227 | 1.52 | https://www.genecards.org/cgi-bin/carddisp.pl?gene=UBE2L6 |
| LDHC | Lactate Dehydrogenase C | Protein Coding | 42 | GC11P018433 | 1.52 | https://www.genecards.org/cgi-bin/carddisp.pl?gene=LDHC |
| RBFOX1 | RNA Binding Fox-1 Homolog 1 | Protein Coding | 37 | GC16P005240 | 1.52 | https://www.genecards.org/cgi-bin/carddisp.pl?gene=RBFOX1 |
| ATOH8 | Atonal BHLH Transcription Factor 8 | Protein Coding | 33 | GC02P085751 | 1.52 | https://www.genecards.org/cgi-bin/carddisp.pl?gene=ATOH8 |
| MIR577 | MicroRNA 577 | RNA Gene | 16 | GC04P114656 | 1.52 | https://www.genecards.org/cgi-bin/carddisp.pl?gene=MIR577 |
| HBA1 | Hemoglobin Subunit Alpha 1 | Protein Coding | 42 | GC16P001370 | 1.52 | https://www.genecards.org/cgi-bin/carddisp.pl?gene=HBA1 |
| SERPINB9 | Serpin Family B Member 9 | Protein Coding | 39 | GC06M002887 | 1.51 | https://www.genecards.org/cgi-bin/carddisp.pl?gene=SERPINB9 |
| TRIP10 | Thyroid Hormone Receptor Interactor 10 | Protein Coding | 40 | GC19P006737 | 1.51 | https://www.genecards.org/cgi-bin/carddisp.pl?gene=TRIP10 |
| TRIP6 | Thyroid Hormone Receptor Interactor 6 | Protein Coding | 39 | GC07P100867 | 1.51 | https://www.genecards.org/cgi-bin/carddisp.pl?gene=TRIP6 |
| SRI | Sorcin | Protein Coding | 42 | GC07M088205 | 1.51 | https://www.genecards.org/cgi-bin/carddisp.pl?gene=SRI |
| HBA2 | Hemoglobin Subunit Alpha 2 | Protein Coding | 42 | GC16P001373 | 1.5 | https://www.genecards.org/cgi-bin/carddisp.pl?gene=HBA2 |
| CTNNBL1 | Catenin Beta Like 1 | Protein Coding | 39 | GC20P037693 | 1.5 | https://www.genecards.org/cgi-bin/carddisp.pl?gene=CTNNBL1 |
| CNTNAP2 | Contactin Associated Protein 2 | Protein Coding | 44 | GC07P146116 | 1.5 | https://www.genecards.org/cgi-bin/carddisp.pl?gene=CNTNAP2 |
| PRDM5 | PR/SET Domain 5 | Protein Coding | 39 | GC04M120686 | 1.5 | https://www.genecards.org/cgi-bin/carddisp.pl?gene=PRDM5 |
| CACYBP | Calcyclin Binding Protein | Protein Coding | 39 | GC01P174968 | 1.5 | https://www.genecards.org/cgi-bin/carddisp.pl?gene=CACYBP |
| IMPDH2 | Inosine Monophosphate Dehydrogenase 2 | Protein Coding | 47 | GC03M049253 | 1.5 | https://www.genecards.org/cgi-bin/carddisp.pl?gene=IMPDH2 |
| DHRS2 | Dehydrogenase/Reductase 2 | Protein Coding | 39 | GC14P025289 | 1.5 | https://www.genecards.org/cgi-bin/carddisp.pl?gene=DHRS2 |
| KIF20A | Kinesin Family Member 20A | Protein Coding | 39 | GC05P138189 | 1.5 | https://www.genecards.org/cgi-bin/carddisp.pl?gene=KIF20A |
| OSR1 | Odd-Skipped Related Transcription Factor 1 | Protein Coding | 36 | GC02M019351 | 1.5 | https://www.genecards.org/cgi-bin/carddisp.pl?gene=OSR1 |
| MUC13 | Mucin 13, Cell Surface Associated | Protein Coding | 36 | GC03M124905 | 1.49 | https://www.genecards.org/cgi-bin/carddisp.pl?gene=MUC13 |
| ZNF423 | Zinc Finger Protein 423 | Protein Coding | 40 | GC16M049487 | 1.49 | https://www.genecards.org/cgi-bin/carddisp.pl?gene=ZNF423 |
| CYP20A1 | Cytochrome P450 Family 20 Subfamily A Member 1 | Protein Coding | 36 | GC02P203238 | 1.49 | https://www.genecards.org/cgi-bin/carddisp.pl?gene=CYP20A1 |
| MIR1301 | MicroRNA 1301 | RNA Gene | 14 | GC02M025328 | 1.49 | https://www.genecards.org/cgi-bin/carddisp.pl?gene=MIR1301 |
| MIR18B | MicroRNA 18b | RNA Gene | 15 | GC0XM134218 | 1.48 | https://www.genecards.org/cgi-bin/carddisp.pl?gene=MIR18B |
| MIR365B | MicroRNA 365b | RNA Gene | 13 | GC17P031572 | 1.48 | https://www.genecards.org/cgi-bin/carddisp.pl?gene=MIR365B |
| ULBP1 | UL16 Binding Protein 1 | Protein Coding | 37 | GC06P149963 | 1.48 | https://www.genecards.org/cgi-bin/carddisp.pl?gene=ULBP1 |
| MUC22 | Mucin 22 | Protein Coding | 24 | GC06P031005 | 1.48 | https://www.genecards.org/cgi-bin/carddisp.pl?gene=MUC22 |
| IFITM4P | Interferon Induced Transmembrane Protein 4 Pseudogene | Pseudogene | 12 | GC06M030848 | 1.48 | https://www.genecards.org/cgi-bin/carddisp.pl?gene=IFITM4P |
| MIR4457 | MicroRNA 4457 | RNA Gene | 9 | GC05M001311 | 1.48 | https://www.genecards.org/cgi-bin/carddisp.pl?gene=MIR4457 |
| RF00017-5428 |  | RNA Gene | 3 | GC06M032849 | 1.48 | https://www.genecards.org/cgi-bin/carddisp.pl?gene=RF00017-5428 |
| CYFIP1 | Cytoplasmic FMR1 Interacting Protein 1 | Protein Coding | 41 | GC15M022867 | 1.48 | https://www.genecards.org/cgi-bin/carddisp.pl?gene=CYFIP1 |
| FMNL1 | Formin Like 1 | Protein Coding | 37 | GC17P045222 | 1.47 | https://www.genecards.org/cgi-bin/carddisp.pl?gene=FMNL1 |
| NETO2 | Neuropilin And Tolloid Like 2 | Protein Coding | 38 | GC16M047077 | 1.47 | https://www.genecards.org/cgi-bin/carddisp.pl?gene=NETO2 |
| ULBP2 | UL16 Binding Protein 2 | Protein Coding | 39 | GC06P149941 | 1.47 | https://www.genecards.org/cgi-bin/carddisp.pl?gene=ULBP2 |
| MIR543 | MicroRNA 543 | RNA Gene | 14 | GC14P104629 | 1.46 | https://www.genecards.org/cgi-bin/carddisp.pl?gene=MIR543 |
| ATG10 | Autophagy Related 10 | Protein Coding | 39 | GC05P081972 | 1.46 | https://www.genecards.org/cgi-bin/carddisp.pl?gene=ATG10 |
| DPP6 | Dipeptidyl Peptidase Like 6 | Protein Coding | 43 | GC07P153748 | 1.46 | https://www.genecards.org/cgi-bin/carddisp.pl?gene=DPP6 |
| FGL2 | Fibrinogen Like 2 | Protein Coding | 39 | GC07M077193 | 1.46 | https://www.genecards.org/cgi-bin/carddisp.pl?gene=FGL2 |
| KIR2DL3 | Killer Cell Immunoglobulin Like Receptor, Two Ig Domains And Long Cytoplasmic Tail 3 | Protein Coding | 36 | GC19P055469 | 1.45 | https://www.genecards.org/cgi-bin/carddisp.pl?gene=KIR2DL3 |
| HAT1 | Histone Acetyltransferase 1 | Protein Coding | 43 | GC02P171922 | 1.45 | https://www.genecards.org/cgi-bin/carddisp.pl?gene=HAT1 |
| CDK16 | Cyclin Dependent Kinase 16 | Protein Coding | 42 | GC0XP047217 | 1.45 | https://www.genecards.org/cgi-bin/carddisp.pl?gene=CDK16 |
| MIR320B2 | MicroRNA 320b-2 | RNA Gene | 14 | GC01M224257 | 1.45 | https://www.genecards.org/cgi-bin/carddisp.pl?gene=MIR320B2 |
| GPC4 | Glypican 4 | Protein Coding | 45 | GC0XM133300 | 1.45 | https://www.genecards.org/cgi-bin/carddisp.pl?gene=GPC4 |
| KIR3DL1 | Killer Cell Immunoglobulin Like Receptor, Three Ig Domains And Long Cytoplasmic Tail 1 | Protein Coding | 40 | GC19P055442 | 1.44 | https://www.genecards.org/cgi-bin/carddisp.pl?gene=KIR3DL1 |
| CD14 | CD14 Molecule | Protein Coding | 44 | GC05M140631 | 1.44 | https://www.genecards.org/cgi-bin/carddisp.pl?gene=CD14 |
| OVOL2 | Ovo Like Zinc Finger 2 | Protein Coding | 36 | GC20M017956 | 1.44 | https://www.genecards.org/cgi-bin/carddisp.pl?gene=OVOL2 |
| FUCA2 | Alpha-L-Fucosidase 2 | Protein Coding | 40 | GC06M143494 | 1.43 | https://www.genecards.org/cgi-bin/carddisp.pl?gene=FUCA2 |
| FCER2 | Fc Fragment Of IgE Receptor II | Protein Coding | 45 | GC19M007689 | 1.43 | https://www.genecards.org/cgi-bin/carddisp.pl?gene=FCER2 |
| IFI16 | Interferon Gamma Inducible Protein 16 | Protein Coding | 42 | GC01P158969 | 1.42 | https://www.genecards.org/cgi-bin/carddisp.pl?gene=IFI16 |
| NFIA | Nuclear Factor I A | Protein Coding | 44 | GC01P060865 | 1.42 | https://www.genecards.org/cgi-bin/carddisp.pl?gene=NFIA |
| ELF2 | E74 Like ETS Transcription Factor 2 | Protein Coding | 38 | GC04M139028 | 1.42 | https://www.genecards.org/cgi-bin/carddisp.pl?gene=ELF2 |
| GALNS | Galactosamine (N-Acetyl)-6-Sulfatase | Protein Coding | 44 | GC16M088813 | 1.41 | https://www.genecards.org/cgi-bin/carddisp.pl?gene=GALNS |
| MAP2K6 | Mitogen-Activated Protein Kinase Kinase 6 | Protein Coding | 47 | GC17P069414 | 1.4 | https://www.genecards.org/cgi-bin/carddisp.pl?gene=MAP2K6 |
| MIR378G | MicroRNA 378g | RNA Gene | 12 | GC01M094745 | 1.4 | https://www.genecards.org/cgi-bin/carddisp.pl?gene=MIR378G |
| SHROOM2 | Shroom Family Member 2 | Protein Coding | 35 | GC0XP009786 | 1.39 | https://www.genecards.org/cgi-bin/carddisp.pl?gene=SHROOM2 |
| LOC284454 | Uncharacterized LOC284454 | RNA Gene | 12 | GC19M014007 | 1.39 | https://www.genecards.org/cgi-bin/carddisp.pl?gene=LOC284454 |
| LINC02570 | Long Intergenic Non-Protein Coding RNA 2570 | RNA Gene | 10 | GC06M030909 | 1.39 | https://www.genecards.org/cgi-bin/carddisp.pl?gene=LINC02570 |
| NAGLU | N-Acetyl-Alpha-Glucosaminidase | Protein Coding | 43 | GC17P042535 | 1.39 | https://www.genecards.org/cgi-bin/carddisp.pl?gene=NAGLU |
| TSGA10 | Testis Specific 10 | Protein Coding | 35 | GC02M098997 | 1.39 | https://www.genecards.org/cgi-bin/carddisp.pl?gene=TSGA10 |
| LINC00319 | Long Intergenic Non-Protein Coding RNA 319 | RNA Gene | 21 | GC21P043446 | 1.39 | https://www.genecards.org/cgi-bin/carddisp.pl?gene=LINC00319 |
| RRAD | RRAD, Ras Related Glycolysis Inhibitor And Calcium Channel Regulator | Protein Coding | 39 | GC16M066925 | 1.39 | https://www.genecards.org/cgi-bin/carddisp.pl?gene=RRAD |
| IL27RA | Interleukin 27 Receptor Subunit Alpha | Protein Coding | 40 | GC19P014031 | 1.38 | https://www.genecards.org/cgi-bin/carddisp.pl?gene=IL27RA |
| LOC344967 | Acyl-CoA Thioesterase 7 Pseudogene | Pseudogene | 13 | GC04M040045 | 1.38 | https://www.genecards.org/cgi-bin/carddisp.pl?gene=LOC344967 |
| CABIN1 | Calcineurin Binding Protein 1 | Protein Coding | 41 | GC22P024011 | 1.38 | https://www.genecards.org/cgi-bin/carddisp.pl?gene=CABIN1 |
| IQCH | IQ Motif Containing H | Protein Coding | 33 | GC15P067254 | 1.38 | https://www.genecards.org/cgi-bin/carddisp.pl?gene=IQCH |
| SFTA2 | Surfactant Associated 2 | Protein Coding | 27 | GC06M030972 | 1.38 | https://www.genecards.org/cgi-bin/carddisp.pl?gene=SFTA2 |
| ENSG00000285551 |  | Protein Coding | 7 | GC10P062520 | 1.38 | https://www.genecards.org/cgi-bin/carddisp.pl?gene=ENSG00000285551 |
| lnc-HLA-C-2 |  | RNA Gene | 5 | GC06M031317 | 1.38 | https://www.genecards.org/cgi-bin/carddisp.pl?gene=lnc-HLA-C-2 |
| ENSG00000285837 |  | Uncategorized | 4 | GC10P062375 | 1.38 | https://www.genecards.org/cgi-bin/carddisp.pl?gene=ENSG00000285837 |
| piR-52740 |  | RNA Gene | 4 | GC06M032558 | 1.38 | https://www.genecards.org/cgi-bin/carddisp.pl?gene=piR-52740 |
| HBP1 | HMG-Box Transcription Factor 1 | Protein Coding | 38 | GC07P107168 | 1.37 | https://www.genecards.org/cgi-bin/carddisp.pl?gene=HBP1 |
| LOC107882129 | EDNRB Proximal Promoter Region | Biological Region | 1 | GC13P077921 | 1.37 | https://www.genecards.org/cgi-bin/carddisp.pl?gene=LOC107882129 |
| CFL2 | Cofilin 2 | Protein Coding | 44 | GC14M034706 | 1.37 | https://www.genecards.org/cgi-bin/carddisp.pl?gene=CFL2 |
| PPP2R2C | Protein Phosphatase 2 Regulatory Subunit Bgamma | Protein Coding | 43 | GC04M006322 | 1.37 | https://www.genecards.org/cgi-bin/carddisp.pl?gene=PPP2R2C |
| PLCD3 | Phospholipase C Delta 3 | Protein Coding | 41 | GC17M045108 | 1.37 | https://www.genecards.org/cgi-bin/carddisp.pl?gene=PLCD3 |
| RIPK3 | Receptor Interacting Serine/Threonine Kinase 3 | Protein Coding | 43 | GC14M024336 | 1.36 | https://www.genecards.org/cgi-bin/carddisp.pl?gene=RIPK3 |
| NOLC1 | Nucleolar And Coiled-Body Phosphoprotein 1 | Protein Coding | 38 | GC10P102152 | 1.36 | https://www.genecards.org/cgi-bin/carddisp.pl?gene=NOLC1 |
| ITGA4 | Integrin Subunit Alpha 4 | Protein Coding | 48 | GC02P181456 | 1.35 | https://www.genecards.org/cgi-bin/carddisp.pl?gene=ITGA4 |
| MIR629 | MicroRNA 629 | RNA Gene | 16 | GC15M070079 | 1.35 | https://www.genecards.org/cgi-bin/carddisp.pl?gene=MIR629 |
| SHISA3 | Shisa Family Member 3 | Protein Coding | 31 | GC04P042399 | 1.34 | https://www.genecards.org/cgi-bin/carddisp.pl?gene=SHISA3 |
| TCOF1 | Treacle Ribosome Biogenesis Factor 1 | Protein Coding | 42 | GC05P150358 | 1.34 | https://www.genecards.org/cgi-bin/carddisp.pl?gene=TCOF1 |
| SSX2IP | SSX Family Member 2 Interacting Protein | Protein Coding | 36 | GC01M084643 | 1.34 | https://www.genecards.org/cgi-bin/carddisp.pl?gene=SSX2IP |
| PPP1R15A | Protein Phosphatase 1 Regulatory Subunit 15A | Protein Coding | 40 | GC19P048872 | 1.33 | https://www.genecards.org/cgi-bin/carddisp.pl?gene=PPP1R15A |
| GALE | UDP-Galactose-4-Epimerase | Protein Coding | 45 | GC01M023795 | 1.33 | https://www.genecards.org/cgi-bin/carddisp.pl?gene=GALE |
| PRR13 | Proline Rich 13 | Protein Coding | 29 | GC12P053442 | 1.33 | https://www.genecards.org/cgi-bin/carddisp.pl?gene=PRR13 |
| ABO | ABO, Alpha 1-3-N-Acetylgalactosaminyltransferase And Alpha 1-3-Galactosyltransferase | Protein Coding | 37 | GC09M133250 | 1.32 | https://www.genecards.org/cgi-bin/carddisp.pl?gene=ABO |
| ADAMTS18 | ADAM Metallopeptidase With Thrombospondin Type 1 Motif 18 | Protein Coding | 43 | GC16M077316 | 1.32 | https://www.genecards.org/cgi-bin/carddisp.pl?gene=ADAMTS18 |
| LINC00290 | Long Intergenic Non-Protein Coding RNA 290 | RNA Gene | 13 | GC04M181064 | 1.32 | https://www.genecards.org/cgi-bin/carddisp.pl?gene=LINC00290 |
| MIR320B1 | MicroRNA 320b-1 | RNA Gene | 14 | GC01P116671 | 1.31 | https://www.genecards.org/cgi-bin/carddisp.pl?gene=MIR320B1 |
| RTN4RL1 | Reticulon 4 Receptor Like 1 | Protein Coding | 37 | GC17M001942 | 1.31 | https://www.genecards.org/cgi-bin/carddisp.pl?gene=RTN4RL1 |
| UFD1 | Ubiquitin Recognition Factor In ER Associated Degradation 1 | Protein Coding | 35 | GC22M019450 | 1.31 | https://www.genecards.org/cgi-bin/carddisp.pl?gene=UFD1 |
| LCN1 | Lipocalin 1 | Protein Coding | 39 | GC09P135521 | 1.31 | https://www.genecards.org/cgi-bin/carddisp.pl?gene=LCN1 |
| UGT1A | UDP Glucuronosyltransferase Family 1 Member A Complex Locus | Uncategorized | 9 | GC02P233586 | 1.31 | https://www.genecards.org/cgi-bin/carddisp.pl?gene=UGT1A |
| ULBP3 | UL16 Binding Protein 3 | Protein Coding | 39 | GC06M150062 | 1.31 | https://www.genecards.org/cgi-bin/carddisp.pl?gene=ULBP3 |
| PLA2G7 | Phospholipase A2 Group VII | Protein Coding | 50 | GC06M046704 | 1.31 | https://www.genecards.org/cgi-bin/carddisp.pl?gene=PLA2G7 |
| PSIP1 | PC4 And SFRS1 Interacting Protein 1 | Protein Coding | 39 | GC09M015456 | 1.3 | https://www.genecards.org/cgi-bin/carddisp.pl?gene=PSIP1 |
| KIR2DS5 | Killer Cell Immunoglobulin Like Receptor, Two Ig Domains And Short Cytoplasmic Tail 5 | Protein Coding | 23 | GC19Mr00079 | 1.3 | https://www.genecards.org/cgi-bin/carddisp.pl?gene=KIR2DS5 |
| MYOCD | Myocardin | Protein Coding | 41 | GC17P012665 | 1.3 | https://www.genecards.org/cgi-bin/carddisp.pl?gene=MYOCD |
| FUT2 | Fucosyltransferase 2 | Protein Coding | 44 | GC19P048695 | 1.3 | https://www.genecards.org/cgi-bin/carddisp.pl?gene=FUT2 |
| KIR2DL1 | Killer Cell Immunoglobulin Like Receptor, Two Ig Domains And Long Cytoplasmic Tail 1 | Protein Coding | 36 | GC19P055437 | 1.3 | https://www.genecards.org/cgi-bin/carddisp.pl?gene=KIR2DL1 |
| GP1BA | Glycoprotein Ib Platelet Subunit Alpha | Protein Coding | 45 | GC17P004932 | 1.29 | https://www.genecards.org/cgi-bin/carddisp.pl?gene=GP1BA |
| VASP | Vasodilator Stimulated Phosphoprotein | Protein Coding | 43 | GC19P045507 | 1.29 | https://www.genecards.org/cgi-bin/carddisp.pl?gene=VASP |
| NLRP12 | NLR Family Pyrin Domain Containing 12 | Protein Coding | 44 | GC19M053793 | 1.29 | https://www.genecards.org/cgi-bin/carddisp.pl?gene=NLRP12 |
| GLYATL1 | Glycine-N-Acyltransferase Like 1 | Protein Coding | 35 | GC11P058906 | 1.29 | https://www.genecards.org/cgi-bin/carddisp.pl?gene=GLYATL1 |
| ESRRB | Estrogen Related Receptor Beta | Protein Coding | 50 | GC14P076310 | 1.29 | https://www.genecards.org/cgi-bin/carddisp.pl?gene=ESRRB |
| SUV39H2 | Suppressor Of Variegation 3-9 Homolog 2 | Protein Coding | 43 | GC10P014878 | 1.29 | https://www.genecards.org/cgi-bin/carddisp.pl?gene=SUV39H2 |
| IL9 | Interleukin 9 | Protein Coding | 43 | GC05M135891 | 1.28 | https://www.genecards.org/cgi-bin/carddisp.pl?gene=IL9 |
| NRG3 | Neuregulin 3 | Protein Coding | 41 | GC10P083672 | 1.28 | https://www.genecards.org/cgi-bin/carddisp.pl?gene=NRG3 |
| KLHDC4 | Kelch Domain Containing 4 | Protein Coding | 37 | GC16M087696 | 1.28 | https://www.genecards.org/cgi-bin/carddisp.pl?gene=KLHDC4 |
| HSPA4L | Heat Shock Protein Family A (Hsp70) Member 4 Like | Protein Coding | 37 | GC04P127781 | 1.28 | https://www.genecards.org/cgi-bin/carddisp.pl?gene=HSPA4L |
| LBH | LBH Regulator Of WNT Signaling Pathway | Protein Coding | 35 | GC02P030231 | 1.28 | https://www.genecards.org/cgi-bin/carddisp.pl?gene=LBH |
| CFAP46 | Cilia And Flagella Associated Protein 46 | Protein Coding | 26 | GC10M132808 | 1.28 | https://www.genecards.org/cgi-bin/carddisp.pl?gene=CFAP46 |
| MIR633 | MicroRNA 633 | RNA Gene | 13 | GC17P062944 | 1.28 | https://www.genecards.org/cgi-bin/carddisp.pl?gene=MIR633 |
| NFE2 | Nuclear Factor, Erythroid 2 | Protein Coding | 40 | GC12M054292 | 1.28 | https://www.genecards.org/cgi-bin/carddisp.pl?gene=NFE2 |
| PRRT1 | Proline Rich Transmembrane Protein 1 | Protein Coding | 32 | GC06M032643 | 1.28 | https://www.genecards.org/cgi-bin/carddisp.pl?gene=PRRT1 |
| ATG14 | Autophagy Related 14 | Protein Coding | 36 | GC14M055366 | 1.27 | https://www.genecards.org/cgi-bin/carddisp.pl?gene=ATG14 |
| HLA-S | Major Histocompatibility Complex, Class I, S (Pseudogene) | Pseudogene | 7 | GC06M031381 | 1.27 | https://www.genecards.org/cgi-bin/carddisp.pl?gene=HLA-S |
| HSP90AA2P | Heat Shock Protein 90 Alpha Family Class A Member 2, Pseudogene | Pseudogene | 20 | GC11M027888 | 1.27 | https://www.genecards.org/cgi-bin/carddisp.pl?gene=HSP90AA2P |
| BOD1 | Biorientation Of Chromosomes In Cell Division 1 | Protein Coding | 36 | GC05M173607 | 1.27 | https://www.genecards.org/cgi-bin/carddisp.pl?gene=BOD1 |
| NUDC | Nuclear Distribution C, Dynein Complex Regulator | Protein Coding | 41 | GC01P026932 | 1.27 | https://www.genecards.org/cgi-bin/carddisp.pl?gene=NUDC |
| NRXN1 | Neurexin 1 | Protein Coding | 47 | GC02M049918 | 1.27 | https://www.genecards.org/cgi-bin/carddisp.pl?gene=NRXN1 |
| A2M | Alpha-2-Macroglobulin | Protein Coding | 45 | GC12M009067 | 1.27 | https://www.genecards.org/cgi-bin/carddisp.pl?gene=A2M |
| YPEL3 | Yippee Like 3 | Protein Coding | 35 | GC16M030093 | 1.26 | https://www.genecards.org/cgi-bin/carddisp.pl?gene=YPEL3 |
| CASP12 | Caspase 12 (Gene/Pseudogene) | Protein Coding | 32 | GC11M104885 | 1.26 | https://www.genecards.org/cgi-bin/carddisp.pl?gene=CASP12 |
| EYS | Eyes Shut Homolog | Protein Coding | 36 | GC06M063719 | 1.26 | https://www.genecards.org/cgi-bin/carddisp.pl?gene=EYS |
| LPAR5 | Lysophosphatidic Acid Receptor 5 | Protein Coding | 41 | GC12M006618 | 1.25 | https://www.genecards.org/cgi-bin/carddisp.pl?gene=LPAR5 |
| VPS33B | VPS33B Late Endosome And Lysosome Associated | Protein Coding | 41 | GC15M090998 | 1.25 | https://www.genecards.org/cgi-bin/carddisp.pl?gene=VPS33B |
| TRIAP1 | TP53 Regulated Inhibitor Of Apoptosis 1 | Protein Coding | 35 | GC12M120443 | 1.25 | https://www.genecards.org/cgi-bin/carddisp.pl?gene=TRIAP1 |
| RBM24 | RNA Binding Motif Protein 24 | Protein Coding | 35 | GC06P017281 | 1.25 | https://www.genecards.org/cgi-bin/carddisp.pl?gene=RBM24 |
| MOG | Myelin Oligodendrocyte Glycoprotein | Protein Coding | 45 | GC06P047247 | 1.25 | https://www.genecards.org/cgi-bin/carddisp.pl?gene=MOG |
| ADO | 2-Aminoethanethiol Dioxygenase | Protein Coding | 37 | GC10P062804 | 1.25 | https://www.genecards.org/cgi-bin/carddisp.pl?gene=ADO |
| TRIL | TLR4 Interactor With Leucine Rich Repeats | Protein Coding | 32 | GC07M028953 | 1.25 | https://www.genecards.org/cgi-bin/carddisp.pl?gene=TRIL |
| GALNT17 | Polypeptide N-Acetylgalactosaminyltransferase 17 | Protein Coding | 31 | GC07P071133 | 1.25 | https://www.genecards.org/cgi-bin/carddisp.pl?gene=GALNT17 |
| FAM155A | Family With Sequence Similarity 155 Member A | Protein Coding | 31 | GC13M107163 | 1.25 | https://www.genecards.org/cgi-bin/carddisp.pl?gene=FAM155A |
| UBA52P6 | Ubiquitin A-52 Residue Ribosomal Protein Fusion Product 1 Pseudogene 6 | Pseudogene | 9 | GC09P022011 | 1.25 | https://www.genecards.org/cgi-bin/carddisp.pl?gene=UBA52P6 |
| MIR6891 | MicroRNA 6891 | RNA Gene | 9 | GC06M031355 | 1.25 | https://www.genecards.org/cgi-bin/carddisp.pl?gene=MIR6891 |
| NONHSAG043472.2 |  | RNA Gene | 4 | GC06P047952 | 1.25 | https://www.genecards.org/cgi-bin/carddisp.pl?gene=NONHSAG043472.2 |
| piR-47864 |  | RNA Gene | 4 | GC06M032550 | 1.25 | https://www.genecards.org/cgi-bin/carddisp.pl?gene=piR-47864 |
| TBX21 | T-Box Transcription Factor 21 | Protein Coding | 45 | GC17P047733 | 1.25 | https://www.genecards.org/cgi-bin/carddisp.pl?gene=TBX21 |
| FGD4 | FYVE, RhoGEF And PH Domain Containing 4 | Protein Coding | 43 | GC12P032407 | 1.24 | https://www.genecards.org/cgi-bin/carddisp.pl?gene=FGD4 |
| ARHGEF3 | Rho Guanine Nucleotide Exchange Factor 3 | Protein Coding | 41 | GC03M056736 | 1.24 | https://www.genecards.org/cgi-bin/carddisp.pl?gene=ARHGEF3 |
| HOXA2 | Homeobox A2 | Protein Coding | 40 | GC07M027100 | 1.24 | https://www.genecards.org/cgi-bin/carddisp.pl?gene=HOXA2 |
| FGF11 | Fibroblast Growth Factor 11 | Protein Coding | 39 | GC17P008020 | 1.24 | https://www.genecards.org/cgi-bin/carddisp.pl?gene=FGF11 |
| KLRC2 | Killer Cell Lectin Like Receptor C2 | Protein Coding | 37 | GC12M013869 | 1.24 | https://www.genecards.org/cgi-bin/carddisp.pl?gene=KLRC2 |
| ATG16L2 | Autophagy Related 16 Like 2 | Protein Coding | 36 | GC11P072814 | 1.24 | https://www.genecards.org/cgi-bin/carddisp.pl?gene=ATG16L2 |
| BEX3 | Brain Expressed X-Linked 3 | Protein Coding | 31 | GC0XP103377 | 1.24 | https://www.genecards.org/cgi-bin/carddisp.pl?gene=BEX3 |
| MIR4649 | MicroRNA 4649 | RNA Gene | 10 | GC07P044112 | 1.24 | https://www.genecards.org/cgi-bin/carddisp.pl?gene=MIR4649 |
| LOC100129148 | Uncharacterized LOC100129148 | RNA Gene | 7 | GC07M139417 | 1.24 | https://www.genecards.org/cgi-bin/carddisp.pl?gene=LOC100129148 |
| RPLP0P11 | Ribosomal Protein Lateral Stalk Subunit P0 Pseudogene 11 | Pseudogene | 5 | GC18P050457 | 1.24 | https://www.genecards.org/cgi-bin/carddisp.pl?gene=RPLP0P11 |
| LOC117307477 | CD209 Promoter Region | Biological Region | 1 | GC19P007749 | 1.24 | https://www.genecards.org/cgi-bin/carddisp.pl?gene=LOC117307477 |
| MX1 | MX Dynamin Like GTPase 1 | Protein Coding | 41 | GC21P041420 | 1.23 | https://www.genecards.org/cgi-bin/carddisp.pl?gene=MX1 |
| NGRN | Neugrin, Neurite Outgrowth Associated | Protein Coding | 33 | GC15P090265 | 1.23 | https://www.genecards.org/cgi-bin/carddisp.pl?gene=NGRN |
| LOC111162621 | DeltaNp63 Promoter Of Tumor Protein P63 | Biological Region | 1 | GC03P189786 | 1.23 | https://www.genecards.org/cgi-bin/carddisp.pl?gene=LOC111162621 |
| ADAMTS8 | ADAM Metallopeptidase With Thrombospondin Type 1 Motif 8 | Protein Coding | 39 | GC11M130308 | 1.23 | https://www.genecards.org/cgi-bin/carddisp.pl?gene=ADAMTS8 |
| INHBE | Inhibin Subunit Beta E | Protein Coding | 37 | GC12P057452 | 1.22 | https://www.genecards.org/cgi-bin/carddisp.pl?gene=INHBE |
| SOX11 | SRY-Box Transcription Factor 11 | Protein Coding | 41 | GC02P005703 | 1.22 | https://www.genecards.org/cgi-bin/carddisp.pl?gene=SOX11 |
| MT-ND5 | Mitochondrially Encoded NADH:Ubiquinone Oxidoreductase Core Subunit 5 | Protein Coding | 31 | GCMTP012339 | 1.21 | https://www.genecards.org/cgi-bin/carddisp.pl?gene=MT-ND5 |
| IL17D | Interleukin 17D | Protein Coding | 38 | GC13P020702 | 1.2 | https://www.genecards.org/cgi-bin/carddisp.pl?gene=IL17D |
| ARVCF | ARVCF Delta Catenin Family Member | Protein Coding | 38 | GC22M019966 | 1.2 | https://www.genecards.org/cgi-bin/carddisp.pl?gene=ARVCF |
| FGF14 | Fibroblast Growth Factor 14 | Protein Coding | 44 | GC13M101710 | 1.2 | https://www.genecards.org/cgi-bin/carddisp.pl?gene=FGF14 |
| SYTL4 | Synaptotagmin Like 4 | Protein Coding | 39 | GC0XM100674 | 1.2 | https://www.genecards.org/cgi-bin/carddisp.pl?gene=SYTL4 |
| MIR1178 | MicroRNA 1178 | RNA Gene | 13 | GC12M119713 | 1.2 | https://www.genecards.org/cgi-bin/carddisp.pl?gene=MIR1178 |
| SLC25A1 | Solute Carrier Family 25 Member 1 | Protein Coding | 46 | GC22M019176 | 1.19 | https://www.genecards.org/cgi-bin/carddisp.pl?gene=SLC25A1 |
| NAA80 | N-Alpha-Acetyltransferase 80, NatH Catalytic Subunit | Protein Coding | 25 | GC03M050310 | 1.19 | https://www.genecards.org/cgi-bin/carddisp.pl?gene=NAA80 |
| RTRAF | RNA Transcription, Translation And Transport Factor | Protein Coding | 28 | GC14P051992 | 1.19 | https://www.genecards.org/cgi-bin/carddisp.pl?gene=RTRAF |
| N4BP2 | NEDD4 Binding Protein 2 | Protein Coding | 33 | GC04P040058 | 1.18 | https://www.genecards.org/cgi-bin/carddisp.pl?gene=N4BP2 |
| ZNF382 | Zinc Finger Protein 382 | Protein Coding | 33 | GC19P038258 | 1.18 | https://www.genecards.org/cgi-bin/carddisp.pl?gene=ZNF382 |
| RASSF4 | Ras Association Domain Family Member 4 | Protein Coding | 37 | GC10P044959 | 1.18 | https://www.genecards.org/cgi-bin/carddisp.pl?gene=RASSF4 |
| LHX2 | LIM Homeobox 2 | Protein Coding | 36 | GC09P124001 | 1.17 | https://www.genecards.org/cgi-bin/carddisp.pl?gene=LHX2 |
| IFITM3 | Interferon Induced Transmembrane Protein 3 | Protein Coding | 41 | GC11M000319 | 1.16 | https://www.genecards.org/cgi-bin/carddisp.pl?gene=IFITM3 |
| LIPE | Lipase E, Hormone Sensitive Type | Protein Coding | 47 | GC19M042401 | 1.16 | https://www.genecards.org/cgi-bin/carddisp.pl?gene=LIPE |
| MIR1306 | MicroRNA 1306 | RNA Gene | 17 | GC22P020086 | 1.16 | https://www.genecards.org/cgi-bin/carddisp.pl?gene=MIR1306 |
| MIR2110 | MicroRNA 2110 | RNA Gene | 13 | GC10M114174 | 1.15 | https://www.genecards.org/cgi-bin/carddisp.pl?gene=MIR2110 |
| LYZ | Lysozyme | Protein Coding | 47 | GC12P069348 | 1.13 | https://www.genecards.org/cgi-bin/carddisp.pl?gene=LYZ |
| PYM1 | PYM Homolog 1, Exon Junction Complex Associated Factor | Protein Coding | 28 | GC12M055902 | 1.13 | https://www.genecards.org/cgi-bin/carddisp.pl?gene=PYM1 |
| HMGCL | 3-Hydroxy-3-Methylglutaryl-CoA Lyase | Protein Coding | 46 | GC01M023801 | 1.13 | https://www.genecards.org/cgi-bin/carddisp.pl?gene=HMGCL |
| CASP4 | Caspase 4 | Protein Coding | 46 | GC11M104942 | 1.12 | https://www.genecards.org/cgi-bin/carddisp.pl?gene=CASP4 |
| LOC108942766 | NANOG 5' Regulatory Region | Biological Region | 1 | GC12P008419 | 1.12 | https://www.genecards.org/cgi-bin/carddisp.pl?gene=LOC108942766 |
| MT-RNR1 | Mitochondrially Encoded 12S RRNA | RNA Gene | 14 | GCMTP000642 | 1.12 | https://www.genecards.org/cgi-bin/carddisp.pl?gene=MT-RNR1 |
| TRD | T Cell Receptor Delta Locus | Protein Coding | 11 | GC14P022425 | 1.12 | https://www.genecards.org/cgi-bin/carddisp.pl?gene=TRD |
| CYP4F12 | Cytochrome P450 Family 4 Subfamily F Member 12 | Protein Coding | 41 | GC19P015672 | 1.11 | https://www.genecards.org/cgi-bin/carddisp.pl?gene=CYP4F12 |
| COX7B2 | Cytochrome C Oxidase Subunit 7B2 | Protein Coding | 32 | GC04M046734 | 1.11 | https://www.genecards.org/cgi-bin/carddisp.pl?gene=COX7B2 |
| FJX1 | Four-Jointed Box Kinase 1 | Protein Coding | 31 | GC11P035618 | 1.11 | https://www.genecards.org/cgi-bin/carddisp.pl?gene=FJX1 |
| MIR597 | MicroRNA 597 | RNA Gene | 14 | GC08P009741 | 1.11 | https://www.genecards.org/cgi-bin/carddisp.pl?gene=MIR597 |
| LRIG2 | Leucine Rich Repeats And Immunoglobulin Like Domains 2 | Protein Coding | 40 | GC01P113073 | 1.11 | https://www.genecards.org/cgi-bin/carddisp.pl?gene=LRIG2 |
| AQP2 | Aquaporin 2 | Protein Coding | 46 | GC12P049950 | 1.1 | https://www.genecards.org/cgi-bin/carddisp.pl?gene=AQP2 |
| IGK | Immunoglobulin Kappa Locus | Protein Coding | 14 | GC02P088857 | 1.1 | https://www.genecards.org/cgi-bin/carddisp.pl?gene=IGK |
| ADCY3 | Adenylate Cyclase 3 | Protein Coding | 47 | GC02M024819 | 1.09 | https://www.genecards.org/cgi-bin/carddisp.pl?gene=ADCY3 |
| CRTAC1 | Cartilage Acidic Protein 1 | Protein Coding | 37 | GC10M097865 | 1.09 | https://www.genecards.org/cgi-bin/carddisp.pl?gene=CRTAC1 |
| REEP3 | Receptor Accessory Protein 3 | Protein Coding | 37 | GC10P063521 | 1.09 | https://www.genecards.org/cgi-bin/carddisp.pl?gene=REEP3 |
| CEP128 | Centrosomal Protein 128 | Protein Coding | 32 | GC14M080476 | 1.09 | https://www.genecards.org/cgi-bin/carddisp.pl?gene=CEP128 |
| TAFA5 | TAFA Chemokine Like Family Member 5 | Protein Coding | 25 | GC22P048490 | 1.09 | https://www.genecards.org/cgi-bin/carddisp.pl?gene=TAFA5 |
| ZDHHC20P1 | Zinc Finger DHHC-Type Containing 20 Pseudogene 1 | Pseudogene | 7 | GC06M030838 | 1.09 | https://www.genecards.org/cgi-bin/carddisp.pl?gene=ZDHHC20P1 |
| ENSG00000271955 |  | RNA Gene | 7 | GC02M059218 | 1.09 | https://www.genecards.org/cgi-bin/carddisp.pl?gene=ENSG00000271955 |
| ENSG00000259202 |  | RNA Gene | 7 | GC15M067142 | 1.09 | https://www.genecards.org/cgi-bin/carddisp.pl?gene=ENSG00000259202 |
| RNU6-75P | RNA, U6 Small Nuclear 75, Pseudogene | Pseudogene | 6 | GC13M090781 | 1.09 | https://www.genecards.org/cgi-bin/carddisp.pl?gene=RNU6-75P |
| RNU6-1133P | RNA, U6 Small Nuclear 1133, Pseudogene | Pseudogene | 6 | GC06P047292 | 1.09 | https://www.genecards.org/cgi-bin/carddisp.pl?gene=RNU6-1133P |
| ENSG00000249883 |  | RNA Gene | 6 | GC04M180731 | 1.09 | https://www.genecards.org/cgi-bin/carddisp.pl?gene=ENSG00000249883 |
| ENSG00000260773 |  | RNA Gene | 6 | GC15M066314 | 1.09 | https://www.genecards.org/cgi-bin/carddisp.pl?gene=ENSG00000260773 |
| ENSG00000250969 |  | RNA Gene | 5 | GC04P143286 | 1.09 | https://www.genecards.org/cgi-bin/carddisp.pl?gene=ENSG00000250969 |
| piR-33458 |  | RNA Gene | 4 | GC15P073267 | 1.09 | https://www.genecards.org/cgi-bin/carddisp.pl?gene=piR-33458 |
| ENSG00000234597 |  | RNA Gene | 4 | GC02M019459 | 1.09 | https://www.genecards.org/cgi-bin/carddisp.pl?gene=ENSG00000234597 |
| RF00017-1368 |  | RNA Gene | 4 | GC13P023495 | 1.09 | https://www.genecards.org/cgi-bin/carddisp.pl?gene=RF00017-1368 |
| LOC105378327 | Uncharacterized LOC105378327 | RNA Gene | 2 | GC10M062624 | 1.09 | https://www.genecards.org/cgi-bin/carddisp.pl?gene=LOC105378327 |
| piR-46506-002 |  | RNA Gene | 2 | GC05M001299 | 1.09 | https://www.genecards.org/cgi-bin/carddisp.pl?gene=piR-46506-002 |
| piR-38580-233 |  | RNA Gene | 2 | GC05M001301 | 1.09 | https://www.genecards.org/cgi-bin/carddisp.pl?gene=piR-38580-233 |
| ENSG00000288218 |  | Pseudogene | 1 | GC10P085156 | 1.09 | https://www.genecards.org/cgi-bin/carddisp.pl?gene=ENSG00000288218 |
| S100A16 | S100 Calcium Binding Protein A16 | Protein Coding | 37 | GC01M153606 | 1.09 | https://www.genecards.org/cgi-bin/carddisp.pl?gene=S100A16 |
| PXN-AS1 | PXN Antisense RNA 1 | RNA Gene | 13 | GC12P120201 | 1.09 | https://www.genecards.org/cgi-bin/carddisp.pl?gene=PXN-AS1 |
| MIR3188 | MicroRNA 3188 | RNA Gene | 13 | GC19P018282 | 1.09 | https://www.genecards.org/cgi-bin/carddisp.pl?gene=MIR3188 |
| UTS2R | Urotensin 2 Receptor | Protein Coding | 41 | GC17P082374 | 1.08 | https://www.genecards.org/cgi-bin/carddisp.pl?gene=UTS2R |
| LARS2 | Leucyl-TRNA Synthetase 2, Mitochondrial | Protein Coding | 45 | GC03P045405 | 1.08 | https://www.genecards.org/cgi-bin/carddisp.pl?gene=LARS2 |
| SGSM1 | Small G Protein Signaling Modulator 1 | Protein Coding | 36 | GC22P024806 | 1.08 | https://www.genecards.org/cgi-bin/carddisp.pl?gene=SGSM1 |
| ETV3 | ETS Variant Transcription Factor 3 | Protein Coding | 36 | GC01M157121 | 1.07 | https://www.genecards.org/cgi-bin/carddisp.pl?gene=ETV3 |
| CYB5R2 | Cytochrome B5 Reductase 2 | Protein Coding | 41 | GC11M007665 | 1.06 | https://www.genecards.org/cgi-bin/carddisp.pl?gene=CYB5R2 |
| PAG1 | Phosphoprotein Membrane Anchor With Glycosphingolipid Microdomains 1 | Protein Coding | 38 | GC08M080967 | 1.06 | https://www.genecards.org/cgi-bin/carddisp.pl?gene=PAG1 |
| CTSS | Cathepsin S | Protein Coding | 45 | GC01M150730 | 1.05 | https://www.genecards.org/cgi-bin/carddisp.pl?gene=CTSS |
| CAMK2G | Calcium/Calmodulin Dependent Protein Kinase II Gamma | Protein Coding | 47 | GC10M073812 | 1.04 | https://www.genecards.org/cgi-bin/carddisp.pl?gene=CAMK2G |
| HIRA | Histone Cell Cycle Regulator | Protein Coding | 42 | GC22M019318 | 1.04 | https://www.genecards.org/cgi-bin/carddisp.pl?gene=HIRA |
| DGCR2 | DiGeorge Syndrome Critical Region Gene 2 | Protein Coding | 39 | GC22M019037 | 1.04 | https://www.genecards.org/cgi-bin/carddisp.pl?gene=DGCR2 |
| SACS | Sacsin Molecular Chaperone | Protein Coding | 36 | GC13M023328 | 1.04 | https://www.genecards.org/cgi-bin/carddisp.pl?gene=SACS |
| RBP7 | Retinol Binding Protein 7 | Protein Coding | 36 | GC01P009997 | 1.04 | https://www.genecards.org/cgi-bin/carddisp.pl?gene=RBP7 |
| MRPL40 | Mitochondrial Ribosomal Protein L40 | Protein Coding | 37 | GC22P019431 | 1.03 | https://www.genecards.org/cgi-bin/carddisp.pl?gene=MRPL40 |
| MIPOL1 | Mirror-Image Polydactyly 1 | Protein Coding | 35 | GC14P037197 | 1.02 | https://www.genecards.org/cgi-bin/carddisp.pl?gene=MIPOL1 |
| KIR2DL5B | Killer Cell Immunoglobulin Like Receptor, Two Ig Domains And Long Cytoplasmic Tail 5B | Protein Coding | 18 | GC19MP00133 | 1.02 | https://www.genecards.org/cgi-bin/carddisp.pl?gene=KIR2DL5B |
| WASF5P | WASP Family Member 5, Pseudogene | Pseudogene | 9 | GC06M031287 | 1.02 | https://www.genecards.org/cgi-bin/carddisp.pl?gene=WASF5P |
| HYAL4 | Hyaluronidase 4 | Protein Coding | 37 | GC07P123769 | 1.01 | https://www.genecards.org/cgi-bin/carddisp.pl?gene=HYAL4 |
| STING1 | Stimulator Of Interferon Response CGAMP Interactor 1 | Protein Coding | 34 | GC05M139476 | 1.01 | https://www.genecards.org/cgi-bin/carddisp.pl?gene=STING1 |
| DEFB103B | Defensin Beta 103B | Protein Coding | 30 | GC08M007430 | 1 | https://www.genecards.org/cgi-bin/carddisp.pl?gene=DEFB103B |
| IL1RL1 | Interleukin 1 Receptor Like 1 | Protein Coding | 41 | GC02P102294 | 1 | https://www.genecards.org/cgi-bin/carddisp.pl?gene=IL1RL1 |
| TAB2 | TGF-Beta Activated Kinase 1 (MAP3K7) Binding Protein 2 | Protein Coding | 47 | GC06P149218 | 0.99 | https://www.genecards.org/cgi-bin/carddisp.pl?gene=TAB2 |
| DLD | Dihydrolipoamide Dehydrogenase | Protein Coding | 50 | GC07P107890 | 0.98 | https://www.genecards.org/cgi-bin/carddisp.pl?gene=DLD |
| RAET1G | Retinoic Acid Early Transcript 1G | Protein Coding | 33 | GC06M149916 | 0.98 | https://www.genecards.org/cgi-bin/carddisp.pl?gene=RAET1G |
| IRF2 | Interferon Regulatory Factor 2 | Protein Coding | 43 | GC04M184387 | 0.97 | https://www.genecards.org/cgi-bin/carddisp.pl?gene=IRF2 |
| ARL6IP1 | ADP Ribosylation Factor Like GTPase 6 Interacting Protein 1 | Protein Coding | 40 | GC16M018792 | 0.96 | https://www.genecards.org/cgi-bin/carddisp.pl?gene=ARL6IP1 |
| IRAG1-AS1 | IRAG1 Antisense RNA 1 | RNA Gene | 12 | GC11P010541 | 0.96 | https://www.genecards.org/cgi-bin/carddisp.pl?gene=IRAG1-AS1 |
| HBB-LCR | Beta-Globin Locus Control Region | Biological Region | 4 | GC11P005270 | 0.95 | https://www.genecards.org/cgi-bin/carddisp.pl?gene=HBB-LCR |
| SIX3 | SIX Homeobox 3 | Protein Coding | 42 | GC02P044941 | 0.93 | https://www.genecards.org/cgi-bin/carddisp.pl?gene=SIX3 |
| CBX1 | Chromobox 1 | Protein Coding | 40 | GC17M048070 | 0.93 | https://www.genecards.org/cgi-bin/carddisp.pl?gene=CBX1 |
| CMTM3 | CKLF Like MARVEL Transmembrane Domain Containing 3 | Protein Coding | 34 | GC16P066609 | 0.92 | https://www.genecards.org/cgi-bin/carddisp.pl?gene=CMTM3 |
| VRK2 | VRK Serine/Threonine Kinase 2 | Protein Coding | 41 | GC02P057907 | 0.92 | https://www.genecards.org/cgi-bin/carddisp.pl?gene=VRK2 |
| KIF22 | Kinesin Family Member 22 | Protein Coding | 43 | GC16P029802 | 0.91 | https://www.genecards.org/cgi-bin/carddisp.pl?gene=KIF22 |
| EBI3 | Epstein-Barr Virus Induced 3 | Protein Coding | 39 | GC19P004232 | 0.9 | https://www.genecards.org/cgi-bin/carddisp.pl?gene=EBI3 |
| BTF3 | Basic Transcription Factor 3 | Protein Coding | 39 | GC05P073498 | 0.9 | https://www.genecards.org/cgi-bin/carddisp.pl?gene=BTF3 |
| FOLR2 | Folate Receptor Beta | Protein Coding | 41 | GC11P072216 | 0.89 | https://www.genecards.org/cgi-bin/carddisp.pl?gene=FOLR2 |
| TANK | TRAF Family Member Associated NFKB Activator | Protein Coding | 43 | GC02P161136 | 0.88 | https://www.genecards.org/cgi-bin/carddisp.pl?gene=TANK |
| C4BPA | Complement Component 4 Binding Protein Alpha | Protein Coding | 40 | GC01P207105 | 0.87 | https://www.genecards.org/cgi-bin/carddisp.pl?gene=C4BPA |
| POU2F2 | POU Class 2 Homeobox 2 | Protein Coding | 43 | GC19M042086 | 0.87 | https://www.genecards.org/cgi-bin/carddisp.pl?gene=POU2F2 |
| HACE1 | HECT Domain And Ankyrin Repeat Containing E3 Ubiquitin Protein Ligase 1 | Protein Coding | 41 | GC06M104728 | 0.87 | https://www.genecards.org/cgi-bin/carddisp.pl?gene=HACE1 |
| GRIN1 | Glutamate Ionotropic Receptor NMDA Type Subunit 1 | Protein Coding | 49 | GC09P137138 | 0.87 | https://www.genecards.org/cgi-bin/carddisp.pl?gene=GRIN1 |
| AP3D1 | Adaptor Related Protein Complex 3 Subunit Delta 1 | Protein Coding | 40 | GC19M002101 | 0.86 | https://www.genecards.org/cgi-bin/carddisp.pl?gene=AP3D1 |
| DUT | Deoxyuridine Triphosphatase | Protein Coding | 42 | GC15P048331 | 0.85 | https://www.genecards.org/cgi-bin/carddisp.pl?gene=DUT |
| TRIP12 | Thyroid Hormone Receptor Interactor 12 | Protein Coding | 43 | GC02M229763 | 0.84 | https://www.genecards.org/cgi-bin/carddisp.pl?gene=TRIP12 |
| HTR3A | 5-Hydroxytryptamine Receptor 3A | Protein Coding | 45 | GC11P113974 | 0.84 | https://www.genecards.org/cgi-bin/carddisp.pl?gene=HTR3A |
| SUZ12 | SUZ12 Polycomb Repressive Complex 2 Subunit | Protein Coding | 41 | GC17P031937 | 0.83 | https://www.genecards.org/cgi-bin/carddisp.pl?gene=SUZ12 |
| CMTM5 | CKLF Like MARVEL Transmembrane Domain Containing 5 | Protein Coding | 36 | GC14P025285 | 0.82 | https://www.genecards.org/cgi-bin/carddisp.pl?gene=CMTM5 |
| H3C14 | H3 Clustered Histone 14 | Protein Coding | 29 | GC01M149963 | 0.81 | https://www.genecards.org/cgi-bin/carddisp.pl?gene=H3C14 |
| ST8SIA4 | ST8 Alpha-N-Acetyl-Neuraminide Alpha-2,8-Sialyltransferase 4 | Protein Coding | 40 | GC05M100806 | 0.8 | https://www.genecards.org/cgi-bin/carddisp.pl?gene=ST8SIA4 |
| ERVW-1 | Endogenous Retrovirus Group W Member 1, Envelope | Protein Coding | 32 | GC07M092468 | 0.8 | https://www.genecards.org/cgi-bin/carddisp.pl?gene=ERVW-1 |
| NR2F2-AS1 | NR2F2 Antisense RNA 1 | RNA Gene | 14 | GC15M103900 | 0.8 | https://www.genecards.org/cgi-bin/carddisp.pl?gene=NR2F2-AS1 |
| SV2A | Synaptic Vesicle Glycoprotein 2A | Protein Coding | 43 | GC01M149903 | 0.79 | https://www.genecards.org/cgi-bin/carddisp.pl?gene=SV2A |
| CCL28 | C-C Motif Chemokine Ligand 28 | Protein Coding | 40 | GC05M043356 | 0.79 | https://www.genecards.org/cgi-bin/carddisp.pl?gene=CCL28 |
| NKX2-2 | NK2 Homeobox 2 | Protein Coding | 40 | GC20M021511 | 0.78 | https://www.genecards.org/cgi-bin/carddisp.pl?gene=NKX2-2 |
| CETP | Cholesteryl Ester Transfer Protein | Protein Coding | 46 | GC16P056961 | 0.77 | https://www.genecards.org/cgi-bin/carddisp.pl?gene=CETP |
| GNAO1 | G Protein Subunit Alpha O1 | Protein Coding | 47 | GC16P056231 | 0.75 | https://www.genecards.org/cgi-bin/carddisp.pl?gene=GNAO1 |
| SRRM4 | Serine/Arginine Repetitive Matrix 4 | Protein Coding | 34 | GC12P118981 | 0.74 | https://www.genecards.org/cgi-bin/carddisp.pl?gene=SRRM4 |
| IFIH1 | Interferon Induced With Helicase C Domain 1 | Protein Coding | 47 | GC02M162267 | 0.74 | https://www.genecards.org/cgi-bin/carddisp.pl?gene=IFIH1 |
| BTG3 | BTG Anti-Proliferation Factor 3 | Protein Coding | 37 | GC21M017594 | 0.72 | https://www.genecards.org/cgi-bin/carddisp.pl?gene=BTG3 |
| IFNL4 | Interferon Lambda 4 (Gene/Pseudogene) | Protein Coding | 19 | GC19M039246 | 0.72 | https://www.genecards.org/cgi-bin/carddisp.pl?gene=IFNL4 |
| TOMM22 | Translocase Of Outer Mitochondrial Membrane 22 | Protein Coding | 36 | GC22P038681 | 0.71 | https://www.genecards.org/cgi-bin/carddisp.pl?gene=TOMM22 |
| LBP | Lipopolysaccharide Binding Protein | Protein Coding | 44 | GC20P038346 | 0.71 | https://www.genecards.org/cgi-bin/carddisp.pl?gene=LBP |
| BPIFB4 | BPI Fold Containing Family B Member 4 | Protein Coding | 32 | GC20P033079 | 0.71 | https://www.genecards.org/cgi-bin/carddisp.pl?gene=BPIFB4 |
| SLC26A3 | Solute Carrier Family 26 Member 3 | Protein Coding | 45 | GC07M107765 | 0.7 | https://www.genecards.org/cgi-bin/carddisp.pl?gene=SLC26A3 |
| EEF1B2 | Eukaryotic Translation Elongation Factor 1 Beta 2 | Protein Coding | 42 | GC02P206159 | 0.69 | https://www.genecards.org/cgi-bin/carddisp.pl?gene=EEF1B2 |
| MYBBP1A | MYB Binding Protein 1a | Protein Coding | 39 | GC17M004538 | 0.69 | https://www.genecards.org/cgi-bin/carddisp.pl?gene=MYBBP1A |
| PRDM1 | PR/SET Domain 1 | Protein Coding | 45 | GC06P105993 | 0.69 | https://www.genecards.org/cgi-bin/carddisp.pl?gene=PRDM1 |
| FCGR2A | Fc Fragment Of IgG Receptor IIa | Protein Coding | 45 | GC01P161505 | 0.68 | https://www.genecards.org/cgi-bin/carddisp.pl?gene=FCGR2A |
| CRIP2 | Cysteine Rich Protein 2 | Protein Coding | 36 | GC14P105472 | 0.67 | https://www.genecards.org/cgi-bin/carddisp.pl?gene=CRIP2 |
| KLRD1 | Killer Cell Lectin Like Receptor D1 | Protein Coding | 41 | GC12P010226 | 0.66 | https://www.genecards.org/cgi-bin/carddisp.pl?gene=KLRD1 |
| FCN2 | Ficolin 2 | Protein Coding | 40 | GC09P134864 | 0.66 | https://www.genecards.org/cgi-bin/carddisp.pl?gene=FCN2 |
| NSRP1 | Nuclear Speckle Splicing Regulatory Protein 1 | Protein Coding | 33 | GC17P030115 | 0.66 | https://www.genecards.org/cgi-bin/carddisp.pl?gene=NSRP1 |
| CBLL1 | Cbl Proto-Oncogene Like 1 | Protein Coding | 37 | GC07P107743 | 0.65 | https://www.genecards.org/cgi-bin/carddisp.pl?gene=CBLL1 |
| RAB18 | RAB18, Member RAS Oncogene Family | Protein Coding | 40 | GC10P027504 | 0.65 | https://www.genecards.org/cgi-bin/carddisp.pl?gene=RAB18 |
| IL5RA | Interleukin 5 Receptor Subunit Alpha | Protein Coding | 46 | GC03M003066 | 0.64 | https://www.genecards.org/cgi-bin/carddisp.pl?gene=IL5RA |
| BPIFB2 | BPI Fold Containing Family B Member 2 | Protein Coding | 33 | GC20P033007 | 0.64 | https://www.genecards.org/cgi-bin/carddisp.pl?gene=BPIFB2 |
| BPIFA2 | BPI Fold Containing Family A Member 2 | Protein Coding | 32 | GC20P033161 | 0.64 | https://www.genecards.org/cgi-bin/carddisp.pl?gene=BPIFA2 |
| HIP1 | Huntingtin Interacting Protein 1 | Protein Coding | 41 | GC07M075533 | 0.63 | https://www.genecards.org/cgi-bin/carddisp.pl?gene=HIP1 |
| USP11 | Ubiquitin Specific Peptidase 11 | Protein Coding | 40 | GC0XP047232 | 0.63 | https://www.genecards.org/cgi-bin/carddisp.pl?gene=USP11 |
| CPEB2 | Cytoplasmic Polyadenylation Element Binding Protein 2 | Protein Coding | 35 | GC04P015005 | 0.63 | https://www.genecards.org/cgi-bin/carddisp.pl?gene=CPEB2 |
| PCLO | Piccolo Presynaptic Cytomatrix Protein | Protein Coding | 41 | GC07M082754 | 0.61 | https://www.genecards.org/cgi-bin/carddisp.pl?gene=PCLO |
| DDX24 | DEAD-Box Helicase 24 | Protein Coding | 38 | GC14M094048 | 0.61 | https://www.genecards.org/cgi-bin/carddisp.pl?gene=DDX24 |
| UGDH | UDP-Glucose 6-Dehydrogenase | Protein Coding | 45 | GC04M039502 | 0.6 | https://www.genecards.org/cgi-bin/carddisp.pl?gene=UGDH |
| FOXO6 | Forkhead Box O6 | Protein Coding | 33 | GC01P041361 | 0.6 | https://www.genecards.org/cgi-bin/carddisp.pl?gene=FOXO6 |
| KLRC1 | Killer Cell Lectin Like Receptor C1 | Protein Coding | 40 | GC12M013870 | 0.59 | https://www.genecards.org/cgi-bin/carddisp.pl?gene=KLRC1 |
| CD244 | CD244 Molecule | Protein Coding | 43 | GC01M160830 | 0.59 | https://www.genecards.org/cgi-bin/carddisp.pl?gene=CD244 |
| KLC1 | Kinesin Light Chain 1 | Protein Coding | 41 | GC14P104644 | 0.59 | https://www.genecards.org/cgi-bin/carddisp.pl?gene=KLC1 |
| CMTM4 | CKLF Like MARVEL Transmembrane Domain Containing 4 | Protein Coding | 34 | GC16M066598 | 0.58 | https://www.genecards.org/cgi-bin/carddisp.pl?gene=CMTM4 |
| BPI | Bactericidal Permeability Increasing Protein | Protein Coding | 41 | GC20P038304 | 0.58 | https://www.genecards.org/cgi-bin/carddisp.pl?gene=BPI |
| JKAMP | JNK1/MAPK8 Associated Membrane Protein | Protein Coding | 34 | GC14P059484 | 0.58 | https://www.genecards.org/cgi-bin/carddisp.pl?gene=JKAMP |
| OXA1L | OXA1L Mitochondrial Inner Membrane Protein | Protein Coding | 39 | GC14P022766 | 0.58 | https://www.genecards.org/cgi-bin/carddisp.pl?gene=OXA1L |
| RSAD2 | Radical S-Adenosyl Methionine Domain Containing 2 | Protein Coding | 40 | GC02P006865 | 0.57 | https://www.genecards.org/cgi-bin/carddisp.pl?gene=RSAD2 |
| CPA6 | Carboxypeptidase A6 | Protein Coding | 43 | GC08M067422 | 0.55 | https://www.genecards.org/cgi-bin/carddisp.pl?gene=CPA6 |
| SRPK2 | SRSF Protein Kinase 2 | Protein Coding | 44 | GC07M105110 | 0.55 | https://www.genecards.org/cgi-bin/carddisp.pl?gene=SRPK2 |
| DHX40 | DEAH-Box Helicase 40 | Protein Coding | 36 | GC17P059565 | 0.54 | https://www.genecards.org/cgi-bin/carddisp.pl?gene=DHX40 |
| PDS5A | PDS5 Cohesin Associated Factor A | Protein Coding | 37 | GC04M039824 | 0.53 | https://www.genecards.org/cgi-bin/carddisp.pl?gene=PDS5A |
| TM2D3 | TM2 Domain Containing 3 | Protein Coding | 33 | GC15M101621 | 0.53 | https://www.genecards.org/cgi-bin/carddisp.pl?gene=TM2D3 |
| IGHE | Immunoglobulin Heavy Constant Epsilon | Protein Coding | 26 | GC14M109515 | 0.53 | https://www.genecards.org/cgi-bin/carddisp.pl?gene=IGHE |
| FLT3LG | Fms Related Receptor Tyrosine Kinase 3 Ligand | Protein Coding | 40 | GC19P049475 | 0.52 | https://www.genecards.org/cgi-bin/carddisp.pl?gene=FLT3LG |
| CMTM7 | CKLF Like MARVEL Transmembrane Domain Containing 7 | Protein Coding | 37 | GC03P032409 | 0.52 | https://www.genecards.org/cgi-bin/carddisp.pl?gene=CMTM7 |
| RHOT1 | Ras Homolog Family Member T1 | Protein Coding | 43 | GC17P032142 | 0.52 | https://www.genecards.org/cgi-bin/carddisp.pl?gene=RHOT1 |
| MANEA | Mannosidase Endo-Alpha | Protein Coding | 36 | GC06P095577 | 0.52 | https://www.genecards.org/cgi-bin/carddisp.pl?gene=MANEA |
| TDO2 | Tryptophan 2,3-Dioxygenase | Protein Coding | 45 | GC04P155854 | 0.52 | https://www.genecards.org/cgi-bin/carddisp.pl?gene=TDO2 |
| BPIFA3 | BPI Fold Containing Family A Member 3 | Protein Coding | 31 | GC20P033217 | 0.51 | https://www.genecards.org/cgi-bin/carddisp.pl?gene=BPIFA3 |
| HCLS1 | Hematopoietic Cell-Specific Lyn Substrate 1 | Protein Coding | 43 | GC03M121631 | 0.51 | https://www.genecards.org/cgi-bin/carddisp.pl?gene=HCLS1 |
| PLSCR1 | Phospholipid Scramblase 1 | Protein Coding | 43 | GC03M146515 | 0.51 | https://www.genecards.org/cgi-bin/carddisp.pl?gene=PLSCR1 |
| MT-RNR2 | Mitochondrially Encoded 16S RRNA | RNA Gene | 18 | GCMTP001674 | 0.49 | https://www.genecards.org/cgi-bin/carddisp.pl?gene=MT-RNR2 |
| ADD2 | Adducin 2 | Protein Coding | 40 | GC02M070626 | 0.48 | https://www.genecards.org/cgi-bin/carddisp.pl?gene=ADD2 |
| LINC02882 | Long Intergenic Non-Protein Coding RNA 2882 | RNA Gene | 10 | GC12M073212 | 0.48 | https://www.genecards.org/cgi-bin/carddisp.pl?gene=LINC02882 |
| BPIFB3 | BPI Fold Containing Family B Member 3 | Protein Coding | 32 | GC20P033059 | 0.47 | https://www.genecards.org/cgi-bin/carddisp.pl?gene=BPIFB3 |
| FBXW11 | F-Box And WD Repeat Domain Containing 11 | Protein Coding | 42 | GC05M171861 | 0.47 | https://www.genecards.org/cgi-bin/carddisp.pl?gene=FBXW11 |
| MT-TS1 | Mitochondrially Encoded TRNA-Ser (UCN) 1 | RNA Gene | 14 | GCMTM007447 | 0.46 | https://www.genecards.org/cgi-bin/carddisp.pl?gene=MT-TS1 |
| MT-TL2 | Mitochondrially Encoded TRNA-Leu (CUN) 2 | RNA Gene | 13 | GCMTP012268 | 0.46 | https://www.genecards.org/cgi-bin/carddisp.pl?gene=MT-TL2 |
| RNR1 | RNA, Ribosomal 45S Cluster 1 | RNA Gene | 9 | GC13U990028 | 0.46 | https://www.genecards.org/cgi-bin/carddisp.pl?gene=RNR1 |
| RNR2 | RNA, Ribosomal 45S Cluster 2 | RNA Gene | 8 | GC14U990054 | 0.46 | https://www.genecards.org/cgi-bin/carddisp.pl?gene=RNR2 |
| CROCC | Ciliary Rootlet Coiled-Coil, Rootletin | Protein Coding | 35 | GC01P016798 | 0.46 | https://www.genecards.org/cgi-bin/carddisp.pl?gene=CROCC |
| OMP | Olfactory Marker Protein | Protein Coding | 37 | GC11P077102 | 0.45 | https://www.genecards.org/cgi-bin/carddisp.pl?gene=OMP |
| ZFAND5 | Zinc Finger AN1-Type Containing 5 | Protein Coding | 38 | GC09M072351 | 0.44 | https://www.genecards.org/cgi-bin/carddisp.pl?gene=ZFAND5 |
| EPC1 | Enhancer Of Polycomb Homolog 1 | Protein Coding | 39 | GC10M032268 | 0.44 | https://www.genecards.org/cgi-bin/carddisp.pl?gene=EPC1 |
| HELZ | Helicase With Zinc Finger | Protein Coding | 35 | GC17M067070 | 0.43 | https://www.genecards.org/cgi-bin/carddisp.pl?gene=HELZ |
| RHBDD1 | Rhomboid Domain Containing 1 | Protein Coding | 35 | GC02P226803 | 0.43 | https://www.genecards.org/cgi-bin/carddisp.pl?gene=RHBDD1 |
| NICN1 | Nicolin 1 | Protein Coding | 33 | GC03M049422 | 0.43 | https://www.genecards.org/cgi-bin/carddisp.pl?gene=NICN1 |
| GMPS | Guanine Monophosphate Synthase | Protein Coding | 44 | GC03P155870 | 0.43 | https://www.genecards.org/cgi-bin/carddisp.pl?gene=GMPS |
| SUGT1 | SGT1 Homolog, MIS12 Kinetochore Complex Assembly Cochaperone | Protein Coding | 36 | GC13P052652 | 0.43 | https://www.genecards.org/cgi-bin/carddisp.pl?gene=SUGT1 |
| IPO13 | Importin 13 | Protein Coding | 37 | GC01P043947 | 0.42 | https://www.genecards.org/cgi-bin/carddisp.pl?gene=IPO13 |
| ZNF300 | Zinc Finger Protein 300 | Protein Coding | 36 | GC05M150894 | 0.42 | https://www.genecards.org/cgi-bin/carddisp.pl?gene=ZNF300 |
| RBM19 | RNA Binding Motif Protein 19 | Protein Coding | 35 | GC12M113816 | 0.42 | https://www.genecards.org/cgi-bin/carddisp.pl?gene=RBM19 |
| IFNL1 | Interferon Lambda 1 | Protein Coding | 35 | GC19P039296 | 0.42 | https://www.genecards.org/cgi-bin/carddisp.pl?gene=IFNL1 |
| TCEAL4 | Transcription Elongation Factor A Like 4 | Protein Coding | 33 | GC0XP103576 | 0.42 | https://www.genecards.org/cgi-bin/carddisp.pl?gene=TCEAL4 |
| EBF1 | EBF Transcription Factor 1 | Protein Coding | 41 | GC05M158695 | 0.4 | https://www.genecards.org/cgi-bin/carddisp.pl?gene=EBF1 |
| ARFGEF1 | ADP Ribosylation Factor Guanine Nucleotide Exchange Factor 1 | Protein Coding | 39 | GC08M067173 | 0.4 | https://www.genecards.org/cgi-bin/carddisp.pl?gene=ARFGEF1 |
| CHODL | Chondrolectin | Protein Coding | 36 | GC21P017819 | 0.4 | https://www.genecards.org/cgi-bin/carddisp.pl?gene=CHODL |
| FBXL7 | F-Box And Leucine Rich Repeat Protein 7 | Protein Coding | 35 | GC05P015553 | 0.4 | https://www.genecards.org/cgi-bin/carddisp.pl?gene=FBXL7 |
| PID1 | Phosphotyrosine Interaction Domain Containing 1 | Protein Coding | 35 | GC02M228850 | 0.4 | https://www.genecards.org/cgi-bin/carddisp.pl?gene=PID1 |
| COMMD10 | COMM Domain Containing 10 | Protein Coding | 33 | GC05P116084 | 0.4 | https://www.genecards.org/cgi-bin/carddisp.pl?gene=COMMD10 |
| FBXO38 | F-Box Protein 38 | Protein Coding | 37 | GC05P148383 | 0.39 | https://www.genecards.org/cgi-bin/carddisp.pl?gene=FBXO38 |
| PPIL4 | Peptidylprolyl Isomerase Like 4 | Protein Coding | 36 | GC06M149504 | 0.39 | https://www.genecards.org/cgi-bin/carddisp.pl?gene=PPIL4 |
| RABAC1 | Rab Acceptor 1 | Protein Coding | 36 | GC19M041956 | 0.39 | https://www.genecards.org/cgi-bin/carddisp.pl?gene=RABAC1 |
| ZFP82 | ZFP82 Zinc Finger Protein | Protein Coding | 29 | GC19M042422 | 0.38 | https://www.genecards.org/cgi-bin/carddisp.pl?gene=ZFP82 |
| ERGIC1 | Endoplasmic Reticulum-Golgi Intermediate Compartment 1 | Protein Coding | 40 | GC05P172834 | 0.38 | https://www.genecards.org/cgi-bin/carddisp.pl?gene=ERGIC1 |
| NUBP1 | Nucleotide Binding Protein 1 | Protein Coding | 40 | GC16P010743 | 0.38 | https://www.genecards.org/cgi-bin/carddisp.pl?gene=NUBP1 |
| IRAG2 | Inositol 1,4,5-Triphosphate Receptor Associated 2 | Protein Coding | 27 | GC12P025004 | 0.38 | https://www.genecards.org/cgi-bin/carddisp.pl?gene=IRAG2 |
| MIR1973 | MicroRNA 1973 | RNA Gene | 10 | GC04P116299 | 0.38 | https://www.genecards.org/cgi-bin/carddisp.pl?gene=MIR1973 |
| CYP2F1 | Cytochrome P450 Family 2 Subfamily F Member 1 | Protein Coding | 41 | GC19P041114 | 0.37 | https://www.genecards.org/cgi-bin/carddisp.pl?gene=CYP2F1 |
| TRG | T Cell Receptor Gamma Locus | Protein Coding | 11 | GC07M038240 | 0.37 | https://www.genecards.org/cgi-bin/carddisp.pl?gene=TRG |
| HOXC13-AS | HOXC13 Antisense RNA | RNA Gene | 15 | GC12M053935 | 0.37 | https://www.genecards.org/cgi-bin/carddisp.pl?gene=HOXC13-AS |
| PPM1G | Protein Phosphatase, Mg2+/Mn2+ Dependent 1G | Protein Coding | 43 | GC02M027382 | 0.35 | https://www.genecards.org/cgi-bin/carddisp.pl?gene=PPM1G |
| PLTP | Phospholipid Transfer Protein | Protein Coding | 43 | GC20M045898 | 0.34 | https://www.genecards.org/cgi-bin/carddisp.pl?gene=PLTP |
| MEF2B | Myocyte Enhancer Factor 2B | Protein Coding | 37 | GC19M019150 | 0.34 | https://www.genecards.org/cgi-bin/carddisp.pl?gene=MEF2B |
| POLR3H | RNA Polymerase III Subunit H | Protein Coding | 37 | GC22M041525 | 0.34 | https://www.genecards.org/cgi-bin/carddisp.pl?gene=POLR3H |
| NPTX1 | Neuronal Pentraxin 1 | Protein Coding | 37 | GC17M080466 | 0.32 | https://www.genecards.org/cgi-bin/carddisp.pl?gene=NPTX1 |
| NTF4 | Neurotrophin 4 | Protein Coding | 44 | GC19M049073 | 0.28 | https://www.genecards.org/cgi-bin/carddisp.pl?gene=NTF4 |
| GZMH | Granzyme H | Protein Coding | 39 | GC14M024606 | 0.28 | https://www.genecards.org/cgi-bin/carddisp.pl?gene=GZMH |
| IFNLR1 | Interferon Lambda Receptor 1 | Protein Coding | 35 | GC01M024155 | 0.24 | https://www.genecards.org/cgi-bin/carddisp.pl?gene=IFNLR1 |
| TRMT1L | TRNA Methyltransferase 1 Like | Protein Coding | 31 | GC01M185088 | 0.24 | https://www.genecards.org/cgi-bin/carddisp.pl?gene=TRMT1L |
| CNTRL | Centriolin | Protein Coding | 36 | GC09P121074 | 0.19 | https://www.genecards.org/cgi-bin/carddisp.pl?gene=CNTRL |
| MPP2 | Membrane Palmitoylated Protein 2 | Protein Coding | 37 | GC17M043875 | 0.19 | https://www.genecards.org/cgi-bin/carddisp.pl?gene=MPP2 |
| KLRC4 | Killer Cell Lectin Like Receptor C4 | Protein Coding | 36 | GC12M013866 | 0.19 | https://www.genecards.org/cgi-bin/carddisp.pl?gene=KLRC4 |
| SNTN | Sentan, Cilia Apical Structure Protein | Protein Coding | 30 | GC03P063652 | 0.19 | https://www.genecards.org/cgi-bin/carddisp.pl?gene=SNTN |
| HTR4 | 5-Hydroxytryptamine Receptor 4 | Protein Coding | 45 | GC05M148451 | 0.19 | https://www.genecards.org/cgi-bin/carddisp.pl?gene=HTR4 |
| RAB11FIP3 | RAB11 Family Interacting Protein 3 | Protein Coding | 37 | GC16P000425 | 0.17 | https://www.genecards.org/cgi-bin/carddisp.pl?gene=RAB11FIP3 |
| ANKRD12 | Ankyrin Repeat Domain 12 | Protein Coding | 35 | GC18P009136 | 0.15 | https://www.genecards.org/cgi-bin/carddisp.pl?gene=ANKRD12 |
| FAM72A | Family With Sequence Similarity 72 Member A | Protein Coding | 33 | GC01M206186 | 0.15 | https://www.genecards.org/cgi-bin/carddisp.pl?gene=FAM72A |
