# Supplementary material for: Luteolin Isolated from Polygonum cuspidatum Is a Potential Compound against Nasopharyngeal Carcinoma
Source: Biomed Res Int. 2022 Dec 23;2022:9740066. doi: 10.1155/2022/9740066 (PMC9803567; doi:10.1155/2022/9740066)
Supplement: Supplementary Materials — Table S1: Basic information of the bioactive compounds of P. cuspidatum. Table S2: The targets for the bioactive compounds of P. cuspidatum in the TCMSP database. Table S3: The standard names of targets for the bioactive compounds of P. cuspidatum. Table S4: Basic information of the disease related targets for NPC. Table S5: The common targets of disease targets for NPC and bioactive compounds from P. cuspidatum. Table S6: GO analysis of common targets of drug compounds and diseases through the DAVID website. Table S7: KEGG pathway analysis of common targets of drug compounds and diseases through the DAVID website. Figure S1: Effect of different bioactive compounds on the survival rate of CNE2 cells in NPC. [file 9740066.f1.zip › Table 5S The common targets of disease targets for NPC and bioactive compounds from P. cuspidatum.docx]

| **drug targets** | **disease targets** | **common targets** |
| --- | --- | --- |
| PGR | TP53 | PGR |
| NCOA2 | PIK3CA | PTGS2 |
| PTGS1 | CDKN2A | BCL2 |
| PTGS2 | EGFR | BAX |
| KCNH2 | PTEN | CASP9 |
| CHRM3 | HRAS | JUN |
| CHRM1 | CDH1 | CASP3 |
| SCN5A | CTNNB1 | CASP8 |
| CHRM4 | ERBB2 | EGFR |
| ADRA1A | MET | AKT1 |
| CHRM2 | AKT1 | VEGFA |
| ADRA1B | CCND1 | CCND1 |
| ADRB2 | TERT | BCL2L1 |
| CHRNA2 | MST1R | CDKN1A |
| SLC6A4 | NRAS | MMP2 |
| OPRM1 | CDKN1A | MMP9 |
| GABRA1 | MTOR | MAPK1 |
| BCL2 | SMAD4 | IL10 |
| BAX | TGFBR2 | RB1 |
| CASP9 | EGF | IL6 |
| JUN | BAX | TP63 |
| CASP3 | MIR21 | NFKBIA |
| CASP8 | MDM2 | MDM2 |
| PRKCA | H19 | MMP1 |
| PON1 | KIT | PCNA |
| MAP2 | MIR31 | ERBB2 |
| PTGS1 | ESR1 | PPARG |
| AR | MMP1 | ICAM1 |
| PTGS2 | MIR143 | BIRC5 |
| PRSS1 | MIR17 | IL2 |
| NCOA2 | VEGFA | CCNB1 |
| RELA | FGFR1 | IFNG |
| EGFR | MIR141 | IL4 |
| AKT1 | RAF1 | GSTP1 |
| VEGFA | MIR125A | MET |
| CCND1 | MIR29C | MMP3 |
| BCL2L1 | MYC | PLAU |
| CDKN1A | MIR205 | EGF |
| CASP9 | MIR185 | RAF1 |
| MMP2 | ERCC1 | HIF1A |
| MMP9 | TWIST1 | STAT1 |
| MAPK1 | MIR30E | CYP3A4 |
| IL10 | MIR93 | CAV1 |
| RB1 | KRT5 | MYC |
| TNFSF15 | MIR10B | CYP1A1 |
| JUN | MMP9 | IL1B |
| IL6 | MIR15A | CXCL8 |
| CASP3 | HIF1A | HSPB1 |
| TP63 | PTGS2 | PARP1 |
| NFKBIA | STAT3 | CHEK2 |
| TOP1 | BCL2 | CRP |
| MDM2 | NFKBIA | SPP1 |
| APP | MIR335 | RASSF1 |
| MMP1 | MIR34C | IGF2 |
| PCNA | IL1B | RASA1 |
| ERBB2 | KRAS | GSTM1 |
| PPARG | MMP2 |  |
| HMOX1 | KRT7 |  |
| CASP7 | CD44 |  |
| ICAM1 | APC |  |
| MCL1 | MUC1 |  |
| BIRC5 | BRAF |  |
| IL2 | MIR100 |  |
| CCNB1 | CD274 |  |
| TYR | IL6 |  |
| IFNG | MIR192 |  |
| IL4 | MIR324 |  |
| TOP2A | MIR27A |  |
| GSTP1 | CXCL8 |  |
| SLC2A4 | CASP3 |  |
| INSR | SMARCB1 |  |
| CD40LG | TNF |  |
| PTGES | NME1 |  |
| NUF2 | RASSF1 |  |
| ADCY2 | NOTCH1 |  |
| MET | MIR34B |  |
| PTGS1 | MIR342 |  |
| AR | MIR140 |  |
| PPARG | MIR193A |  |
| PTGS2 | STK11 |  |
| NCOA2 | MIR224 |  |
| AKR1B1 | KRT19 |  |
| PRSS1 | VEGFC |  |
| KCNH2 | FGFR2 |  |
| SCN5A | MLH1 |  |
| ADRB2 | CDK4 |  |
| MMP3 | GSTM1 |  |
| F7 | JUN |  |
| RXRA | CA9 |  |
| ACHE | MIR148A |  |
| GABRA1 | XRCC1 |  |
| MAOB | MIR483 |  |
| RELA | BCL2L1 |  |
| EGFR | EZH2 |  |
| AKT1 | CXCR4 |  |
| VEGFA | PGR |  |
| CCND1 | BRCA1 |  |
| BCL2 | NPCA2 |  |
| BCL2L1 | VHL |  |
| FOS | IL10 |  |
| CDKN1A | CDKN1B |  |
| EIF6 | FGFR3 |  |
| BAX | MIR486-1 |  |
| CASP9 | CASP9 |  |
| PLAU | KRT20 |  |
| MMP2 | VIM |  |
| MMP9 | MALAT1 |  |
| MAPK1 | FHIT |  |
| IL10 | HOTAIR |  |
| EGF | FGF2 |  |
| RB1 | CCNB1 |  |
| TNFSF15 | NKX2-1 |  |
| JUN | PARP1 |  |
| IL6 | CDH2 |  |
| AHSA1 | DNMT1 |  |
| CASP3 | RB1 |  |
| TP63 | MAPK8 |  |
| ELK1 | SOX2 |  |
| NFKBIA | PCNA |  |
| POR | MIR15B |  |
| ODC1 | CXCL12 |  |
| CASP8 | MIR30B |  |
| TOP1 | CDKN2B-AS1 |  |
| RAF1 | EPCAM |  |
| SOD1 | FAS |  |
| PRKCA | MIR30A |  |
| MMP1 | KRT18 |  |
| HIF1A | BMI1 |  |
| STAT1 | ABCC1 |  |
| RUNX1T1 | SNAI1 |  |
| ERBB2 | CYCS |  |
| PPARG | DNMT3B |  |
| ACACA | PTCH1 |  |
| HMOX1 | HSPB1 |  |
| CYP3A4 | KRT8 |  |
| CYP1A2 | SNAI2 |  |
| CAV1 | MIR9-1 |  |
| MYC | PVT1 |  |
| F3 | ANXA5 |  |
| GJA1 | MEG3 |  |
| CYP1A1 | MMP3 |  |
| ICAM1 | MIR139 |  |
| IL1B | TMEM8B |  |
| CCL2 | MIR144 |  |
| SELE | CASP8 |  |
| VCAM1 | DAPK1 |  |
| PTGER3 | ATM |  |
| CXCL8 | BPIFA1 |  |
| PRKCB | OGG1 |  |
| BIRC5 | ZEB1 |  |
| DUOX2 | EZR |  |
| NOS3 | GAS5 |  |
| HSPB1 | MIR142 |  |
| SULT1E1 | CHEK2 |  |
| MGAM | ENO2 |  |
| IL2 | TLR2 |  |
| NR1I2 | CDKN2B |  |
| CYP1B1 | CYP2E1 |  |
| CCNB1 | HLA-A |  |
| PLAT | MIR423 |  |
| THBD | NEAT1 |  |
| SERPINE1 | LINC00312 |  |
| COL1A1 | XIST |  |
| IFNG | MIR296 |  |
| ALOX5 | CCAT1 |  |
| IL1A | HULC |  |
| MPO | FASLG |  |
| TOP2A | BIRC5 |  |
| NCF1 | LINC-ROR |  |
| ABCG2 | ETS1 |  |
| HAS2 | MIR338 |  |
| GSTP1 | PDCD1 |  |
| NFE2L2 | ALB |  |
| NQO1 | MIR29B1 |  |
| PARP1 | PSMB9 |  |
| AHR | LRRC56 |  |
| PSMD3 | SETD2 |  |
| SLC2A4 | NPC1 |  |
| COL3A1 | MUC5AC |  |
| CXCL11 | HSPA4 |  |
| CXCL2 | CDH13 |  |
| DCAF5 | NTRK1 |  |
| NR1I3 | MIR193B |  |
| CHEK2 | CCR6 |  |
| INSR | TP63 |  |
| CLDN4 | IFNG |  |
| PPARA | AFAP1-AS1 |  |
| PPARD | ACTC1 |  |
| HSF1 | MKI67 |  |
| CRP | KRT13 |  |
| CXCL10 | MIR199A1 |  |
| CHUK | IGF2 |  |
| SPP1 | MIR24-1 |  |
| RUNX2 | HLA-B |  |
| RASSF1 | ENG |  |
| E2F1 | TGFB1 |  |
| E2F2 | CD80 |  |
| ACPP | ABCC2 |  |
| CTSD | CSF2 |  |
| IGFBP3 | MIR145 |  |
| IGF2 | SERPINA3 |  |
| CD40LG | LGALS3 |  |
| IRF1 | MIR212 |  |
| ERBB3 | CYP19A1 |  |
| PON1 | TKT |  |
| DIO1 | THY1 |  |
| PCOLCE | BMP6 |  |
| NPEPPS | UBAP1 |  |
| HK2 | IL4 |  |
| RASA1 | PDGFRA |  |
| GSTM1 | PECAM1 |  |
| GSTM2 | SRC |  |
|  | KDR |  |
|  | PPARG |  |
|  | CRP |  |
|  | ANKRD11 |  |
|  | IL17A |  |
|  | PMS2 |  |
|  | ICAM1 |  |
|  | STAT1 |  |
|  | CASC9 |  |
|  | MIR26B |  |
|  | S100A1 |  |
|  | MIR129-1 |  |
|  | ERCC2 |  |
|  | MIR452 |  |
|  | NPTN-IT1 |  |
|  | TNFRSF10B |  |
|  | CCR7 |  |
|  | TP73 |  |
|  | TG |  |
|  | GSTP1 |  |
|  | MIR34A |  |
|  | TGFB3 |  |
|  | H2AC18 |  |
|  | EP300 |  |
|  | SDHB |  |
|  | TLR4 |  |
|  | CDKN3 |  |
|  | MIR155 |  |
|  | MAPK1 |  |
|  | MIR363 |  |
|  | MAD2L1 |  |
|  | PIGR |  |
|  | NKILA |  |
|  | MIR214 |  |
|  | MIR151A |  |
|  | MIR200A |  |
|  | HNF1A-AS1 |  |
|  | MPP1 |  |
|  | CEACAM3 |  |
|  | POSTN |  |
|  | IL1RN |  |
|  | CADM1 |  |
|  | TLR3 |  |
|  | PCAT7 |  |
|  | FLT4 |  |
|  | MIR744 |  |
|  | MIR146A |  |
|  | IFNA2 |  |
|  | BRD7 |  |
|  | ZMYND10 |  |
|  | CSF3 |  |
|  | MIR200C |  |
|  | PDGFRB |  |
|  | NPCA1 |  |
|  | CEACAM5 |  |
|  | FN1 |  |
|  | KLRK1 |  |
|  | FOXD2-AS1 |  |
|  | CTNNA1 |  |
|  | TERC |  |
|  | IGF1R |  |
|  | AURKA |  |
|  | MIR150 |  |
|  | CYP3A4 |  |
|  | MIR196A1 |  |
|  | IL2 |  |
|  | TGFBR1 |  |
|  | FBXW7 |  |
|  | LINC00460 |  |
|  | MIR99A |  |
|  | FOXCUT |  |
|  | FH |  |
|  | SYP |  |
|  | RAD51 |  |
|  | ABCB1 |  |
|  | CHGA |  |
|  | MIR204 |  |
|  | ICOSLG |  |
|  | HGF |  |
|  | FOLR1 |  |
|  | MIR378A |  |
|  | INS |  |
|  | NCAM1 |  |
|  | BAP1 |  |
|  | MIR203A |  |
|  | JUP |  |
|  | GPT |  |
|  | FBXO30 |  |
|  | SMO |  |
|  | NFKB1 |  |
|  | JAK1 |  |
|  | AFP |  |
|  | TNFSF10 |  |
|  | FLT1 |  |
|  | MIRLET7A1 |  |
|  | SLC2A1 |  |
|  | CYP1A1 |  |
|  | CD99 |  |
|  | MIR200B |  |
|  | MAPK3 |  |
|  | XRCC3 |  |
|  | MVK |  |
|  | JAK2 |  |
|  | IL13 |  |
|  | MIR222 |  |
|  | BPIFB1 |  |
|  | TYMP |  |
|  | CCL5 |  |
|  | BCL3 |  |
|  | MSH3 |  |
|  | MIR23A |  |
|  | SPP1 |  |
|  | HLA-DRB1 |  |
|  | MIR216A |  |
|  | MIR210 |  |
|  | MIR122 |  |
|  | WWOX |  |
|  | CD4 |  |
|  | MIR490 |  |
|  | NBN |  |
|  | TLR9 |  |
|  | DICER1 |  |
|  | IFNB1 |  |
|  | MIR183 |  |
|  | MIR223 |  |
|  | MIR18A |  |
|  | PSMB8 |  |
|  | OSCP1 |  |
|  | CAV1 |  |
|  | MIR106B |  |
|  | BMP2 |  |
|  | MIR29A |  |
|  | NUTM1 |  |
|  | COLQ |  |
|  | IFI27 |  |
|  | TYMS |  |
|  | CR2 |  |
|  | MIR28 |  |
|  | CHEK1 |  |
|  | CD8A |  |
|  | PDLIM7 |  |
|  | KLF6 |  |
|  | RASA1 |  |
|  | TIMP2 |  |
|  | TBX1 |  |
|  | ITGB4 |  |
|  | MMP7 |  |
|  | S100B |  |
|  | NTRK3 |  |
|  | PLAU |  |
|  | RARB |  |
|  | RAC1 |  |
|  | MIR23B |  |
