# Supplementary material for: Luteolin Isolated from Polygonum cuspidatum Is a Potential Compound against Nasopharyngeal Carcinoma
Source: Biomed Res Int. 2022 Dec 23;2022:9740066. doi: 10.1155/2022/9740066 (PMC9803567; doi:10.1155/2022/9740066)
Supplement: Supplementary Materials — Table S1: Basic information of the bioactive compounds of P. cuspidatum. Table S2: The targets for the bioactive compounds of P. cuspidatum in the TCMSP database. Table S3: The standard names of targets for the bioactive compounds of P. cuspidatum. Table S4: Basic information of the disease related targets for NPC. Table S5: The common targets of disease targets for NPC and bioactive compounds from P. cuspidatum. Table S6: GO analysis of common targets of drug compounds and diseases through the DAVID website. Table S7: KEGG pathway analysis of common targets of drug compounds and diseases through the DAVID website. Figure S1: Effect of different bioactive compounds on the survival rate of CNE2 cells in NPC. [file 9740066.f1.zip › Table 6S GO analysis of common targets of drug compounds and diseases through the DAVID website.docx]

| ID | Description | GeneRatio | BgRatio | pvalue | p.adjust | qvalue | geneID | Count |
| --- | --- | --- | --- | --- | --- | --- | --- | --- |
| GO:0044389 | ubiquitin-like protein ligase binding | 12/56 | 316/18352 | 1.56E-10 | 4.18E-08 | 2.09E-08 | BCL2/JUN/CASP8/EGFR/CDKN1A/RB1/NFKBIA/MDM2/CCNB1/HIF1A/STAT1/CHEK2 | 12 |
| GO:0005126 | cytokine receptor binding | 11/56 | 271/18352 | 4.92E-10 | 6.56E-08 | 3.29E-08 | CASP3/CASP8/VEGFA/IL10/IL6/IL2/IFNG/IL4/STAT1/IL1B/CXCL8 | 11 |
| GO:0140297 | DNA-binding transcription factor binding | 11/56 | 347/18352 | 6.57E-09 | 5.84E-07 | 2.93E-07 | BCL2/JUN/RB1/NFKBIA/PCNA/PPARG/HIF1A/STAT1/MYC/HSPB1/PARP1 | 11 |
| GO:0019207 | kinase regulator activity | 9/56 | 216/18352 | 1.72E-08 | 1.04E-06 | 5.23E-07 | CASP3/CCND1/CDKN1A/IL2/CCNB1/GSTP1/EGF/HSPB1/IGF2 | 9 |
| GO:0031625 | ubiquitin protein ligase binding | 10/56 | 297/18352 | 1.95E-08 | 1.04E-06 | 5.23E-07 | BCL2/JUN/CASP8/EGFR/CDKN1A/RB1/NFKBIA/MDM2/HIF1A/CHEK2 | 10 |
| GO:0005125 | cytokine activity | 9/56 | 235/18352 | 3.57E-08 | 1.59E-06 | 7.94E-07 | VEGFA/IL10/IL6/IL2/IFNG/IL4/IL1B/CXCL8/SPP1 | 9 |
| GO:0061629 | RNA polymerase II-specific DNA-binding transcription factor binding | 9/56 | 267/18352 | 1.06E-07 | 4.06E-06 | 2.03E-06 | JUN/RB1/NFKBIA/PCNA/PPARG/HIF1A/STAT1/HSPB1/PARP1 | 9 |
| GO:0019902 | phosphatase binding | 8/56 | 194/18352 | 1.24E-07 | 4.15E-06 | 2.08E-06 | BCL2/EGFR/AKT1/MAPK1/ERBB2/PPARG/MET/STAT1 | 8 |
| GO:0048018 | receptor ligand activity | 11/56 | 487/18352 | 2.08E-07 | 5.59E-06 | 2.80E-06 | VEGFA/IL10/IL6/IL2/IFNG/IL4/EGF/IL1B/CXCL8/SPP1/IGF2 | 11 |
| GO:0030546 | signaling receptor activator activity | 11/56 | 492/18352 | 2.30E-07 | 5.59E-06 | 2.80E-06 | VEGFA/IL10/IL6/IL2/IFNG/IL4/EGF/IL1B/CXCL8/SPP1/IGF2 | 11 |
| GO:0070851 | growth factor receptor binding | 7/56 | 141/18352 | 2.30E-07 | 5.59E-06 | 2.80E-06 | VEGFA/IL10/IL6/IL2/IL4/EGF/IL1B | 7 |
| GO:0019903 | protein phosphatase binding | 7/56 | 149/18352 | 3.36E-07 | 7.47E-06 | 3.74E-06 | BCL2/EGFR/AKT1/ERBB2/PPARG/MET/STAT1 | 7 |
| GO:0008083 | growth factor activity | 7/56 | 162/18352 | 5.91E-07 | 1.21E-05 | 6.08E-06 | VEGFA/IL10/IL6/IL2/IL4/EGF/IGF2 | 7 |
| GO:0019887 | protein kinase regulator activity | 7/56 | 185/18352 | 1.44E-06 | 2.75E-05 | 1.38E-05 | CASP3/CCND1/CDKN1A/CCNB1/EGF/HSPB1/IGF2 | 7 |
| GO:0097199 | cysteine-type endopeptidase activity involved in apoptotic signaling pathway | 3/56 | 10/18352 | 3.18E-06 | 5.66E-05 | 2.83E-05 | CASP9/CASP3/CASP8 | 3 |
| GO:0051400 | BH domain binding | 3/56 | 11/18352 | 4.36E-06 | 6.85E-05 | 3.43E-05 | BCL2/BAX/BCL2L1 | 3 |
| GO:0070513 | death domain binding | 3/56 | 11/18352 | 4.36E-06 | 6.85E-05 | 3.43E-05 | BCL2/BAX/BCL2L1 | 3 |
| GO:0019209 | kinase activator activity | 5/56 | 89/18352 | 7.53E-06 | 0.000111655 | 5.59E-05 | CDKN1A/IL2/CCNB1/EGF/IGF2 | 5 |
| GO:0097153 | cysteine-type endopeptidase activity involved in apoptotic process | 3/56 | 15/18352 | 1.19E-05 | 0.000167663 | 8.39E-05 | CASP9/CASP3/CASP8 | 3 |
| GO:0016538 | cyclin-dependent protein serine/threonine kinase regulator activity | 4/56 | 50/18352 | 1.61E-05 | 0.000215332 | 0.000107815 | CASP3/CCND1/CDKN1A/CCNB1 | 4 |
| GO:0004175 | endopeptidase activity | 8/56 | 440/18352 | 5.31E-05 | 0.000674828 | 0.00033788 | CASP9/CASP3/CASP8/MMP2/MMP9/MMP1/MMP3/PLAU | 8 |
| GO:0070491 | repressing transcription factor binding | 4/56 | 74/18352 | 7.63E-05 | 0.000854488 | 0.000427833 | BCL2/PPARG/STAT1/MYC | 4 |
| GO:0005178 | integrin binding | 5/56 | 144/18352 | 7.68E-05 | 0.000854488 | 0.000427833 | EGFR/ICAM1/IL1B/SPP1/IGF2 | 5 |
| GO:0035257 | nuclear hormone receptor binding | 5/56 | 144/18352 | 7.68E-05 | 0.000854488 | 0.000427833 | PCNA/PPARG/HIF1A/STAT1/PARP1 | 5 |
| GO:0035035 | histone acetyltransferase binding | 3/56 | 28/18352 | 8.35E-05 | 0.000891996 | 0.000446614 | PCNA/HIF1A/STAT1 | 3 |
| GO:0033613 | activating transcription factor binding | 4/56 | 80/18352 | 0.000103501 | 0.001062873 | 0.00053217 | JUN/RB1/PPARG/MYC | 4 |
| GO:0030291 | protein serine/threonine kinase inhibitor activity | 3/56 | 31/18352 | 0.000113857 | 0.001086542 | 0.000544021 | CASP3/CDKN1A/HSPB1 | 3 |
| GO:0030295 | protein kinase activator activity | 4/56 | 82/18352 | 0.000113944 | 0.001086542 | 0.000544021 | CDKN1A/CCNB1/EGF/IGF2 | 4 |
| GO:0051721 | protein phosphatase 2A binding | 3/56 | 32/18352 | 0.000125364 | 0.001154212 | 0.000577903 | BCL2/AKT1/STAT1 | 3 |
| GO:0004252 | serine-type endopeptidase activity | 5/56 | 169/18352 | 0.000163111 | 0.00145169 | 0.000726847 | MMP2/MMP9/MMP1/MMP3/PLAU | 5 |
| GO:0051427 | hormone receptor binding | 5/56 | 177/18352 | 0.000202336 | 0.001742699 | 0.000872552 | PCNA/PPARG/HIF1A/STAT1/PARP1 | 5 |
| GO:0016248 | channel inhibitor activity | 3/56 | 39/18352 | 0.000227519 | 0.001898364 | 0.000950492 | BCL2/CAV1/RASA1 | 3 |
| GO:0008236 | serine-type peptidase activity | 5/56 | 187/18352 | 0.000261047 | 0.002112107 | 0.001057511 | MMP2/MMP9/MMP1/MMP3/PLAU | 5 |
| GO:0030331 | estrogen receptor binding | 3/56 | 42/18352 | 0.000283953 | 0.002195855 | 0.001099442 | PCNA/PPARG/PARP1 | 3 |
| GO:0017171 | serine hydrolase activity | 5/56 | 191/18352 | 0.000287846 | 0.002195855 | 0.001099442 | MMP2/MMP9/MMP1/MMP3/PLAU | 5 |
| GO:0004222 | metalloendopeptidase activity | 4/56 | 108/18352 | 0.000329208 | 0.002441629 | 0.001222499 | MMP2/MMP9/MMP1/MMP3 | 4 |
| GO:0032813 | tumor necrosis factor receptor superfamily binding | 3/56 | 48/18352 | 0.000422302 | 0.002987634 | 0.001495878 | CASP3/CASP8/STAT1 | 3 |
| GO:0046982 | protein heterodimerization activity | 6/56 | 321/18352 | 0.000425206 | 0.002987634 | 0.001495878 | BCL2/BAX/BCL2L1/ERBB2/HIF1A/CAV1 | 6 |
| GO:0070888 | E-box binding | 3/56 | 50/18352 | 0.000476496 | 0.003262164 | 0.001633333 | PPARG/HIF1A/MYC | 3 |
| GO:0004861 | cyclin-dependent protein serine/threonine kinase inhibitor activity | 2/56 | 12/18352 | 0.000591876 | 0.003854413 | 0.001929866 | CASP3/CDKN1A | 2 |
| GO:0043295 | glutathione binding | 2/56 | 12/18352 | 0.000591876 | 0.003854413 | 0.001929866 | GSTP1/GSTM1 | 2 |
| GO:1900750 | oligopeptide binding | 2/56 | 13/18352 | 0.00069812 | 0.004438052 | 0.002222088 | GSTP1/GSTM1 | 2 |
| GO:0004714 | transmembrane receptor protein tyrosine kinase activity | 3/56 | 61/18352 | 0.000854438 | 0.005305464 | 0.002656392 | EGFR/ERBB2/MET | 3 |
| GO:0004860 | protein kinase inhibitor activity | 3/56 | 65/18352 | 0.001028105 | 0.006238726 | 0.003123667 | CASP3/CDKN1A/HSPB1 | 3 |
| GO:0002039 | p53 binding | 3/56 | 66/18352 | 0.001074744 | 0.006376813 | 0.003192806 | TP63/MDM2/HIF1A | 3 |
| GO:0019210 | kinase inhibitor activity | 3/56 | 69/18352 | 0.001222627 | 0.00709655 | 0.003553171 | CASP3/CDKN1A/HSPB1 | 3 |
| GO:0005123 | death receptor binding | 2/56 | 20/18352 | 0.0016774 | 0.009529058 | 0.004771104 | CASP3/CASP8 | 2 |
| GO:0019199 | transmembrane receptor protein kinase activity | 3/56 | 80/18352 | 0.00187236 | 0.010415004 | 0.005214688 | EGFR/ERBB2/MET | 3 |
| GO:0035258 | steroid hormone receptor binding | 3/56 | 81/18352 | 0.001940196 | 0.010572088 | 0.005293338 | PCNA/PPARG/PARP1 | 3 |
| GO:0070412 | R-SMAD binding | 2/56 | 23/18352 | 0.002220521 | 0.011658026 | 0.005837056 | JUN/PARP1 | 2 |
| GO:0051219 | phosphoprotein binding | 3/56 | 85/18352 | 0.002226814 | 0.011658026 | 0.005837056 | MAPK1/RB1/RASA1 | 3 |
| GO:0004709 | MAP kinase kinase kinase activity | 2/56 | 24/18352 | 0.002417655 | 0.012383475 | 0.006200281 | EGFR/RAF1 | 2 |
| GO:0051117 | ATPase binding | 3/56 | 88/18352 | 0.002458143 | 0.012383475 | 0.006200281 | PGR/EGFR/CAV1 | 3 |
| GO:0004364 | glutathione transferase activity | 2/56 | 25/18352 | 0.002622754 | 0.012567245 | 0.006292293 | GSTP1/GSTM1 | 2 |
| GO:0070330 | aromatase activity | 2/56 | 25/18352 | 0.002622754 | 0.012567245 | 0.006292293 | CYP3A4/CYP1A1 | 2 |
| GO:0008237 | metallopeptidase activity | 4/56 | 189/18352 | 0.002635827 | 0.012567245 | 0.006292293 | MMP2/MMP9/MMP1/MMP3 | 4 |
| GO:0016922 | nuclear receptor binding | 3/56 | 101/18352 | 0.003630255 | 0.017004878 | 0.008514171 | PCNA/PPARG/PARP1 | 3 |
| GO:0005164 | tumor necrosis factor receptor binding | 2/56 | 31/18352 | 0.0040179 | 0.018496193 | 0.009260858 | CASP8/STAT1 | 2 |
| GO:0005496 | steroid binding | 3/56 | 106/18352 | 0.004157668 | 0.01881521 | 0.009420586 | PGR/CYP3A4/CAV1 | 3 |
| GO:0004197 | cysteine-type endopeptidase activity | 3/56 | 114/18352 | 0.005094169 | 0.022328248 | 0.011179529 | CASP9/CASP3/CASP8 | 3 |
| GO:0016712 | oxidoreductase activity, acting on paired donors, with incorporation or reduction of molecular oxygen, reduced flavin or flavoprotein as one donor, and incorporation of one atom of oxygen | 2/56 | 35/18352 | 0.00510121 | 0.022328248 | 0.011179529 | CYP3A4/CYP1A1 | 2 |
| GO:0042826 | histone deacetylase binding | 3/56 | 115/18352 | 0.005219422 | 0.02247719 | 0.011254102 | CCND1/HIF1A/PARP1 | 3 |
| GO:0019825 | oxygen binding | 2/56 | 36/18352 | 0.005390757 | 0.022489565 | 0.011260299 | CYP3A4/CYP1A1 | 2 |
| GO:0097718 | disordered domain specific binding | 2/56 | 36/18352 | 0.005390757 | 0.022489565 | 0.011260299 | RB1/MDM2 | 2 |
| GO:0008200 | ion channel inhibitor activity | 2/56 | 38/18352 | 0.005991989 | 0.023878525 | 0.011955737 | CAV1/RASA1 | 2 |
| GO:0008395 | steroid hydroxylase activity | 2/56 | 38/18352 | 0.005991989 | 0.023878525 | 0.011955737 | CYP3A4/CYP1A1 | 2 |
| GO:0043539 | protein serine/threonine kinase activator activity | 2/56 | 38/18352 | 0.005991989 | 0.023878525 | 0.011955737 | CCNB1/IGF2 | 2 |
| GO:0005504 | fatty acid binding | 2/56 | 39/18352 | 0.006303582 | 0.024750831 | 0.012392492 | PPARG/GSTP1 | 2 |
| GO:0031072 | heat shock protein binding | 3/56 | 127/18352 | 0.006868558 | 0.026578333 | 0.013307504 | BAX/HIF1A/CYP1A1 | 3 |
| GO:0001784 | phosphotyrosine residue binding | 2/56 | 42/18352 | 0.00728172 | 0.027003046 | 0.013520153 | MAPK1/RASA1 | 2 |
| GO:0030544 | Hsp70 protein binding | 2/56 | 42/18352 | 0.00728172 | 0.027003046 | 0.013520153 | BAX/CYP1A1 | 2 |
| GO:0051879 | Hsp90 protein binding | 2/56 | 42/18352 | 0.00728172 | 0.027003046 | 0.013520153 | HIF1A/CYP1A1 | 2 |
| GO:0004713 | protein tyrosine kinase activity | 3/56 | 135/18352 | 0.008121571 | 0.029633249 | 0.014837069 | EGFR/ERBB2/MET | 3 |
| GO:0004712 | protein serine/threonine/tyrosine kinase activity | 2/56 | 45/18352 | 0.008323946 | 0.029633249 | 0.014837069 | AKT1/MAPK1 | 2 |
| GO:0016709 | oxidoreductase activity, acting on paired donors, with incorporation or reduction of molecular oxygen, NAD(P)H as one donor, and incorporation of one atom of oxygen | 2/56 | 45/18352 | 0.008323946 | 0.029633249 | 0.014837069 | CYP3A4/CYP1A1 | 2 |
| GO:0020037 | heme binding | 3/56 | 138/18352 | 0.008623893 | 0.03010702 | 0.015074282 | PTGS2/CYP3A4/CYP1A1 | 3 |
| GO:0001046 | core promoter sequence-specific DNA binding | 2/56 | 46/18352 | 0.008685387 | 0.03010702 | 0.015074282 | STAT1/MYC | 2 |
| GO:0008234 | cysteine-type peptidase activity | 3/56 | 139/18352 | 0.008795309 | 0.03010702 | 0.015074282 | CASP9/CASP3/CASP8 | 3 |
| GO:0001102 | RNA polymerase II activating transcription factor binding | 2/56 | 47/18352 | 0.009053771 | 0.030599453 | 0.015320838 | JUN/RB1 | 2 |
| GO:0004674 | protein serine/threonine kinase activity | 5/56 | 435/18352 | 0.010366489 | 0.033956093 | 0.017001474 | EGFR/AKT1/MAPK1/RAF1/CHEK2 | 5 |
| GO:0016247 | channel regulator activity | 3/56 | 148/18352 | 0.010428463 | 0.033956093 | 0.017001474 | BCL2/CAV1/RASA1 | 3 |
| GO:0046906 | tetrapyrrole binding | 3/56 | 148/18352 | 0.010428463 | 0.033956093 | 0.017001474 | PTGS2/CYP3A4/CYP1A1 | 3 |
| GO:0004601 | peroxidase activity | 2/56 | 52/18352 | 0.01099828 | 0.034547539 | 0.017297605 | PTGS2/GSTP1 | 2 |
| GO:0004879 | nuclear receptor activity | 2/56 | 52/18352 | 0.01099828 | 0.034547539 | 0.017297605 | PGR/PPARG | 2 |
| GO:0098531 | ligand-activated transcription factor activity | 2/56 | 52/18352 | 0.01099828 | 0.034547539 | 0.017297605 | PGR/PPARG | 2 |
| GO:0015459 | potassium channel regulator activity | 2/56 | 53/18352 | 0.011407396 | 0.035008904 | 0.017528606 | CAV1/RASA1 | 2 |
| GO:0045309 | protein phosphorylated amino acid binding | 2/56 | 53/18352 | 0.011407396 | 0.035008904 | 0.017528606 | MAPK1/RASA1 | 2 |
| GO:0005080 | protein kinase C binding | 2/56 | 55/18352 | 0.012245495 | 0.037153945 | 0.018602606 | AKT1/HSPB1 | 2 |
| GO:0016684 | oxidoreductase activity, acting on peroxide as acceptor | 2/56 | 56/18352 | 0.012674394 | 0.037600701 | 0.018826292 | PTGS2/GSTP1 | 2 |
| GO:0016765 | transferase activity, transferring alkyl or aryl (other than methyl) groups | 2/56 | 56/18352 | 0.012674394 | 0.037600701 | 0.018826292 | GSTP1/GSTM1 | 2 |
| GO:0050840 | extracellular matrix binding | 2/56 | 57/18352 | 0.013109802 | 0.038465022 | 0.019259049 | VEGFA/SPP1 | 2 |
| GO:0016705 | oxidoreductase activity, acting on paired donors, with incorporation or reduction of molecular oxygen | 3/56 | 162/18352 | 0.013297826 | 0.038592603 | 0.019322928 | PTGS2/CYP3A4/CYP1A1 | 3 |
| GO:0042277 | peptide binding | 4/56 | 308/18352 | 0.014420989 | 0.041057383 | 0.020557018 | NFKBIA/PPARG/GSTP1/GSTM1 | 4 |
| GO:0097110 | scaffold protein binding | 2/56 | 60/18352 | 0.014454659 | 0.041057383 | 0.020557018 | CASP8/MDM2 | 2 |
| GO:0003684 | damaged DNA binding | 2/56 | 65/18352 | 0.016822369 | 0.047279712 | 0.023672475 | TP63/PCNA | 2 |
