# Supplementary material for: Luteolin Isolated from Polygonum cuspidatum Is a Potential Compound against Nasopharyngeal Carcinoma
Source: Biomed Res Int. 2022 Dec 23;2022:9740066. doi: 10.1155/2022/9740066 (PMC9803567; doi:10.1155/2022/9740066)
Supplement: Supplementary Materials — Table S1: Basic information of the bioactive compounds of P. cuspidatum. Table S2: The targets for the bioactive compounds of P. cuspidatum in the TCMSP database. Table S3: The standard names of targets for the bioactive compounds of P. cuspidatum. Table S4: Basic information of the disease related targets for NPC. Table S5: The common targets of disease targets for NPC and bioactive compounds from P. cuspidatum. Table S6: GO analysis of common targets of drug compounds and diseases through the DAVID website. Table S7: KEGG pathway analysis of common targets of drug compounds and diseases through the DAVID website. Figure S1: Effect of different bioactive compounds on the survival rate of CNE2 cells in NPC. [file 9740066.f1.zip › Table 7S KEGG pathway analysis of common targets of drug compounds and diseases through the DAVID website.docx]

| ID | Description | GeneRatio | BgRatio | pvalue | p.adjust | qvalue | geneID | Count |
| --- | --- | --- | --- | --- | --- | --- | --- | --- |
| hsa05167 | Kaposi sarcoma-associated herpesvirus infection | 20/55 | 193/8081 | 3.30E-19 | 1.65E-17 | 4.95E-18 | PTGS2/BAX/CASP9/JUN/CASP3/CASP8/AKT1/VEGFA/CCND1/CDKN1A/MAPK1/RB1/IL6/NFKBIA/ICAM1/RAF1/HIF1A/STAT1/MYC/CXCL8 | 20 |
| hsa04151 | PI3K-Akt signaling pathway | 20/55 | 354/8081 | 4.95E-14 | 6.60E-13 | 1.98E-13 | BCL2/CASP9/EGFR/AKT1/VEGFA/CCND1/BCL2L1/CDKN1A/MAPK1/IL6/MDM2/ERBB2/IL2/IL4/MET/EGF/RAF1/MYC/SPP1/IGF2 | 20 |
| hsa05161 | Hepatitis B | 19/55 | 162/8081 | 2.84E-19 | 1.65E-17 | 4.95E-18 | BCL2/BAX/CASP9/JUN/CASP3/CASP8/AKT1/CDKN1A/MMP9/MAPK1/RB1/IL6/NFKBIA/PCNA/BIRC5/RAF1/STAT1/MYC/CXCL8 | 19 |
| hsa05163 | Human cytomegalovirus infection | 19/55 | 225/8081 | 1.52E-16 | 4.35E-15 | 1.31E-15 | PTGS2/BAX/CASP9/CASP3/CASP8/EGFR/AKT1/VEGFA/CCND1/CDKN1A/MAPK1/RB1/IL6/NFKBIA/MDM2/RAF1/MYC/IL1B/CXCL8 | 19 |
| hsa05205 | Proteoglycans in cancer | 18/55 | 205/8081 | 5.66E-16 | 1.26E-14 | 3.77E-15 | CASP3/EGFR/AKT1/VEGFA/CCND1/CDKN1A/MMP2/MMP9/MAPK1/MDM2/ERBB2/MET/PLAU/RAF1/HIF1A/CAV1/MYC/IGF2 | 18 |
| hsa05215 | Prostate cancer | 17/55 | 97/8081 | 2.44E-20 | 2.44E-18 | 7.32E-19 | BCL2/CASP9/EGFR/AKT1/CCND1/CDKN1A/MMP9/MAPK1/RB1/NFKBIA/MDM2/ERBB2/GSTP1/MMP3/PLAU/EGF/RAF1 | 17 |
| hsa05206 | MicroRNAs in cancer | 17/55 | 310/8081 | 9.94E-12 | 6.63E-11 | 1.99E-11 | PTGS2/BCL2/CASP3/EGFR/VEGFA/CCND1/CDKN1A/MMP9/MAPK1/TP63/MDM2/ERBB2/MET/PLAU/RAF1/MYC/RASSF1 | 17 |
| hsa05219 | Bladder cancer | 16/55 | 41/8081 | 1.76E-25 | 3.51E-23 | 1.05E-23 | EGFR/VEGFA/CCND1/CDKN1A/MMP2/MMP9/MAPK1/RB1/MDM2/MMP1/ERBB2/EGF/RAF1/MYC/CXCL8/RASSF1 | 16 |
| hsa05160 | Hepatitis C | 16/55 | 157/8081 | 2.95E-15 | 4.22E-14 | 1.27E-14 | BAX/CASP9/CASP3/CASP8/EGFR/AKT1/CCND1/CDKN1A/MAPK1/RB1/NFKBIA/IFNG/EGF/RAF1/STAT1/MYC | 16 |
| hsa05169 | Epstein-Barr virus infection | 16/55 | 202/8081 | 1.62E-13 | 1.62E-12 | 4.86E-13 | BCL2/BAX/CASP9/JUN/CASP3/CASP8/AKT1/CCND1/CDKN1A/RB1/IL6/NFKBIA/MDM2/ICAM1/STAT1/MYC | 16 |
| hsa05165 | Human papillomavirus infection | 16/55 | 331/8081 | 3.05E-10 | 1.49E-09 | 4.47E-10 | PTGS2/BAX/CASP3/CASP8/EGFR/AKT1/VEGFA/CCND1/CDKN1A/MAPK1/RB1/MDM2/EGF/RAF1/STAT1/SPP1 | 16 |
| hsa04630 | JAK-STAT signaling pathway | 15/55 | 162/8081 | 1.05E-13 | 1.17E-12 | 3.51E-13 | BCL2/EGFR/AKT1/CCND1/BCL2L1/CDKN1A/IL10/IL6/IL2/IFNG/IL4/EGF/RAF1/STAT1/MYC | 15 |
| hsa04010 | MAPK signaling pathway | 15/55 | 294/8081 | 5.81E-10 | 2.64E-09 | 7.92E-10 | JUN/CASP3/EGFR/AKT1/VEGFA/MAPK1/ERBB2/MET/EGF/RAF1/MYC/IL1B/HSPB1/IGF2/RASA1 | 15 |
| hsa01524 | Platinum drug resistance | 14/55 | 73/8081 | 2.12E-17 | 8.46E-16 | 2.54E-16 | BCL2/BAX/CASP9/CASP3/CASP8/AKT1/BCL2L1/CDKN1A/MAPK1/MDM2/ERBB2/BIRC5/GSTP1/GSTM1 | 14 |
| hsa05212 | Pancreatic cancer | 14/55 | 76/8081 | 3.88E-17 | 1.29E-15 | 3.88E-16 | BAX/CASP9/EGFR/AKT1/VEGFA/CCND1/BCL2L1/CDKN1A/MAPK1/RB1/ERBB2/EGF/RAF1/STAT1 | 14 |
| hsa05210 | Colorectal cancer | 14/55 | 86/8081 | 2.44E-16 | 6.10E-15 | 1.83E-15 | BCL2/BAX/CASP9/JUN/CASP3/EGFR/AKT1/CCND1/CDKN1A/MAPK1/BIRC5/EGF/RAF1/MYC | 14 |
| hsa04657 | IL-17 signaling pathway | 14/55 | 94/8081 | 9.02E-16 | 1.64E-14 | 4.92E-15 | PTGS2/JUN/CASP3/CASP8/MMP9/MAPK1/IL6/NFKBIA/MMP1/IFNG/IL4/MMP3/IL1B/CXCL8 | 14 |
| hsa01522 | Endocrine resistance | 14/55 | 98/8081 | 1.66E-15 | 2.76E-14 | 8.28E-15 | BCL2/BAX/JUN/EGFR/AKT1/CCND1/CDKN1A/MMP2/MMP9/MAPK1/RB1/MDM2/ERBB2/RAF1 | 14 |
| hsa04933 | AGE-RAGE signaling pathway in diabetic complications | 14/55 | 100/8081 | 2.22E-15 | 3.42E-14 | 1.03E-14 | BCL2/BAX/JUN/CASP3/AKT1/VEGFA/CCND1/MMP2/MAPK1/IL6/ICAM1/STAT1/IL1B/CXCL8 | 14 |
| hsa05162 | Measles | 14/55 | 139/8081 | 2.44E-13 | 2.33E-12 | 6.98E-13 | BCL2/BAX/CASP9/JUN/CASP3/CASP8/AKT1/CCND1/BCL2L1/IL6/NFKBIA/IL2/STAT1/IL1B | 14 |
| hsa05225 | Hepatocellular carcinoma | 14/55 | 168/8081 | 3.40E-12 | 2.62E-11 | 7.85E-12 | BAX/EGFR/AKT1/CCND1/BCL2L1/CDKN1A/MAPK1/RB1/GSTP1/MET/RAF1/MYC/IGF2/GSTM1 | 14 |
| hsa05164 | Influenza A | 14/55 | 171/8081 | 4.34E-12 | 3.10E-11 | 9.29E-12 | BAX/CASP9/CASP3/CASP8/AKT1/MAPK1/IL6/NFKBIA/ICAM1/IFNG/RAF1/STAT1/IL1B/CXCL8 | 14 |
| hsa05166 | Human T-cell leukemia virus 1 infection | 14/55 | 219/8081 | 1.24E-10 | 6.71E-10 | 2.01E-10 | BAX/JUN/AKT1/CCND1/BCL2L1/CDKN1A/MAPK1/RB1/IL6/NFKBIA/ICAM1/IL2/MYC/CHEK2 | 14 |
| hsa05223 | Non-small cell lung cancer | 13/55 | 72/8081 | 7.75E-16 | 1.55E-14 | 4.65E-15 | BAX/CASP9/EGFR/AKT1/CCND1/CDKN1A/MAPK1/RB1/ERBB2/MET/EGF/RAF1/RASSF1 | 13 |
| hsa04625 | C-type lectin receptor signaling pathway | 13/55 | 104/8081 | 1.14E-13 | 1.20E-12 | 3.61E-13 | PTGS2/JUN/CASP8/AKT1/MAPK1/IL10/IL6/NFKBIA/MDM2/IL2/RAF1/STAT1/IL1B | 13 |
| hsa04210 | Apoptosis | 13/55 | 136/8081 | 3.87E-12 | 2.87E-11 | 8.60E-12 | BCL2/BAX/CASP9/JUN/CASP3/CASP8/AKT1/BCL2L1/MAPK1/NFKBIA/BIRC5/RAF1/PARP1 | 13 |
| hsa05224 | Breast cancer | 13/55 | 147/8081 | 1.05E-11 | 6.80E-11 | 2.04E-11 | PGR/BAX/JUN/EGFR/AKT1/CCND1/CDKN1A/MAPK1/RB1/ERBB2/EGF/RAF1/MYC | 13 |
| hsa05226 | Gastric cancer | 13/55 | 149/8081 | 1.25E-11 | 7.84E-11 | 2.35E-11 | BCL2/BAX/EGFR/AKT1/CCND1/CDKN1A/MAPK1/RB1/ERBB2/MET/EGF/RAF1/MYC | 13 |
| hsa05152 | Tuberculosis | 13/55 | 180/8081 | 1.38E-10 | 7.28E-10 | 2.18E-10 | BCL2/BAX/CASP9/CASP3/CASP8/AKT1/MAPK1/IL10/IL6/IFNG/RAF1/STAT1/IL1B | 13 |
| hsa04510 | Focal adhesion | 13/55 | 201/8081 | 5.49E-10 | 2.55E-09 | 7.66E-10 | BCL2/JUN/EGFR/AKT1/VEGFA/CCND1/MAPK1/ERBB2/MET/EGF/RAF1/CAV1/SPP1 | 13 |
| hsa05132 | Salmonella infection | 13/55 | 249/8081 | 7.57E-09 | 2.91E-08 | 8.74E-09 | BCL2/BAX/JUN/CASP3/CASP8/AKT1/MAPK1/IL6/NFKBIA/RAF1/MYC/IL1B/CXCL8 | 13 |
| hsa01521 | EGFR tyrosine kinase inhibitor resistance | 12/55 | 79/8081 | 1.00E-13 | 1.17E-12 | 3.51E-13 | BCL2/BAX/EGFR/AKT1/VEGFA/BCL2L1/MAPK1/IL6/ERBB2/MET/EGF/RAF1 | 12 |
| hsa05222 | Small cell lung cancer | 12/55 | 92/8081 | 6.67E-13 | 6.07E-12 | 1.82E-12 | PTGS2/BCL2/BAX/CASP9/CASP3/AKT1/CCND1/BCL2L1/CDKN1A/RB1/NFKBIA/MYC | 12 |
| hsa04668 | TNF signaling pathway | 12/55 | 112/8081 | 7.35E-12 | 5.07E-11 | 1.52E-11 | PTGS2/JUN/CASP3/CASP8/AKT1/MMP9/MAPK1/IL6/NFKBIA/ICAM1/MMP3/IL1B | 12 |
| hsa05418 | Fluid shear stress and atherosclerosis | 12/55 | 139/8081 | 9.70E-11 | 5.54E-10 | 1.66E-10 | BCL2/JUN/AKT1/VEGFA/MMP2/MMP9/ICAM1/IFNG/GSTP1/CAV1/IL1B/GSTM1 | 12 |
| hsa04218 | Cellular senescence | 12/55 | 156/8081 | 3.76E-10 | 1.79E-09 | 5.37E-10 | AKT1/CCND1/CDKN1A/MAPK1/RB1/IL6/MDM2/CCNB1/RAF1/MYC/CXCL8/CHEK2 | 12 |
| hsa05202 | Transcriptional misregulation in cancer | 12/55 | 192/8081 | 4.12E-09 | 1.65E-08 | 4.95E-09 | BAX/BCL2L1/CDKN1A/MMP9/IL6/MDM2/PPARG/MET/MMP3/PLAU/MYC/CXCL8 | 12 |
| hsa05170 | Human immunodeficiency virus 1 infection | 12/55 | 212/8081 | 1.27E-08 | 4.52E-08 | 1.36E-08 | BCL2/BAX/CASP9/JUN/CASP3/CASP8/AKT1/BCL2L1/MAPK1/NFKBIA/CCNB1/RAF1 | 12 |
| hsa05168 | Herpes simplex virus 1 infection | 12/55 | 498/8081 | 0.000100707 | 0.000228879 | 6.87E-05 | BCL2/BAX/CASP9/CASP3/CASP8/AKT1/BCL2L1/IL6/NFKBIA/IFNG/STAT1/IL1B | 12 |
| hsa05213 | Endometrial cancer | 11/55 | 58/8081 | 9.01E-14 | 1.13E-12 | 3.38E-13 | BAX/CASP9/EGFR/AKT1/CCND1/CDKN1A/MAPK1/ERBB2/EGF/RAF1/MYC | 11 |
| hsa05218 | Melanoma | 11/55 | 72/8081 | 1.11E-12 | 9.69E-12 | 2.91E-12 | BAX/EGFR/AKT1/CCND1/CDKN1A/MAPK1/RB1/MDM2/MET/EGF/RAF1 | 11 |
| hsa04115 | p53 signaling pathway | 11/55 | 73/8081 | 1.31E-12 | 1.09E-11 | 3.27E-12 | BCL2/BAX/CASP9/CASP3/CASP8/CCND1/BCL2L1/CDKN1A/MDM2/CCNB1/CHEK2 | 11 |
| hsa05220 | Chronic myeloid leukemia | 11/55 | 76/8081 | 2.07E-12 | 1.66E-11 | 4.97E-12 | BAX/AKT1/CCND1/BCL2L1/CDKN1A/MAPK1/RB1/NFKBIA/MDM2/RAF1/MYC | 11 |
| hsa05142 | Chagas disease | 11/55 | 102/8081 | 5.65E-11 | 3.32E-10 | 9.97E-11 | JUN/CASP8/AKT1/MAPK1/IL10/IL6/NFKBIA/IL2/IFNG/IL1B/CXCL8 | 11 |
| hsa04066 | HIF-1 signaling pathway | 11/55 | 109/8081 | 1.17E-10 | 6.53E-10 | 1.96E-10 | BCL2/EGFR/AKT1/VEGFA/CDKN1A/MAPK1/IL6/ERBB2/IFNG/EGF/HIF1A | 11 |
| hsa05145 | Toxoplasmosis | 11/55 | 112/8081 | 1.58E-10 | 8.11E-10 | 2.43E-10 | BCL2/CASP9/CASP3/CASP8/AKT1/BCL2L1/MAPK1/IL10/NFKBIA/IFNG/STAT1 | 11 |
| hsa04068 | FoxO signaling pathway | 11/55 | 131/8081 | 8.69E-10 | 3.86E-09 | 1.16E-09 | EGFR/AKT1/CCND1/CDKN1A/MAPK1/IL10/IL6/MDM2/CCNB1/EGF/RAF1 | 11 |
| hsa04014 | Ras signaling pathway | 11/55 | 232/8081 | 3.39E-07 | 9.67E-07 | 2.90E-07 | EGFR/AKT1/VEGFA/BCL2L1/MAPK1/MET/EGF/RAF1/RASSF1/IGF2/RASA1 | 11 |
| hsa05171 | Coronavirus disease - COVID-19 | 11/55 | 232/8081 | 3.39E-07 | 9.67E-07 | 2.90E-07 | JUN/EGFR/MAPK1/IL6/NFKBIA/MMP1/IL2/MMP3/STAT1/IL1B/CXCL8 | 11 |
| hsa05131 | Shigellosis | 11/55 | 246/8081 | 6.09E-07 | 1.72E-06 | 5.15E-07 | BCL2/BAX/JUN/EGFR/AKT1/BCL2L1/MAPK1/NFKBIA/MDM2/IL1B/CXCL8 | 11 |
| hsa05022 | Pathways of neurodegeneration - multiple diseases | 11/55 | 475/8081 | 0.000291362 | 0.000619919 | 0.000185976 | PTGS2/BCL2/BAX/CASP9/CASP3/CASP8/BCL2L1/MAPK1/IL6/RAF1/IL1B | 11 |
| hsa05214 | Glioma | 10/55 | 75/8081 | 5.35E-11 | 3.24E-10 | 9.73E-11 | BAX/EGFR/AKT1/CCND1/CDKN1A/MAPK1/RB1/MDM2/EGF/RAF1 | 10 |
| hsa05235 | PD-L1 expression and PD-1 checkpoint pathway in cancer | 10/55 | 89/8081 | 3.06E-10 | 1.49E-09 | 4.47E-10 | JUN/EGFR/AKT1/MAPK1/NFKBIA/IFNG/EGF/RAF1/HIF1A/STAT1 | 10 |
| hsa04620 | Toll-like receptor signaling pathway | 10/55 | 104/8081 | 1.45E-09 | 6.18E-09 | 1.85E-09 | JUN/CASP8/AKT1/MAPK1/IL6/NFKBIA/STAT1/IL1B/CXCL8/SPP1 | 10 |
| hsa04659 | Th17 cell differentiation | 10/55 | 107/8081 | 1.93E-09 | 7.86E-09 | 2.36E-09 | JUN/MAPK1/IL6/NFKBIA/IL2/IFNG/IL4/HIF1A/STAT1/IL1B | 10 |
| hsa04926 | Relaxin signaling pathway | 10/55 | 129/8081 | 1.20E-08 | 4.38E-08 | 1.31E-08 | JUN/EGFR/AKT1/VEGFA/MMP2/MMP9/MAPK1/NFKBIA/MMP1/RAF1 | 10 |
| hsa04621 | NOD-like receptor signaling pathway | 10/55 | 181/8081 | 3.03E-07 | 9.04E-07 | 2.71E-07 | BCL2/JUN/CASP8/BCL2L1/MAPK1/IL6/NFKBIA/STAT1/IL1B/CXCL8 | 10 |
| hsa05130 | Pathogenic Escherichia coli infection | 10/55 | 197/8081 | 6.64E-07 | 1.85E-06 | 5.54E-07 | BAX/CASP9/JUN/CASP3/CASP8/MAPK1/IL6/NFKBIA/IL1B/CXCL8 | 10 |
| hsa05203 | Viral carcinogenesis | 10/55 | 204/8081 | 9.16E-07 | 2.48E-06 | 7.43E-07 | BAX/JUN/CASP3/CASP8/CCND1/CDKN1A/MAPK1/RB1/NFKBIA/MDM2 | 10 |
| hsa05140 | Leishmaniasis | 9/55 | 77/8081 | 1.79E-09 | 7.47E-09 | 2.24E-09 | PTGS2/JUN/MAPK1/IL10/NFKBIA/IFNG/IL4/STAT1/IL1B | 9 |
| hsa04012 | ErbB signaling pathway | 9/55 | 85/8081 | 4.39E-09 | 1.72E-08 | 5.16E-09 | JUN/EGFR/AKT1/CDKN1A/MAPK1/ERBB2/EGF/RAF1/MYC | 9 |
| hsa05323 | Rheumatoid arthritis | 9/55 | 93/8081 | 9.84E-09 | 3.71E-08 | 1.11E-08 | JUN/VEGFA/IL6/MMP1/ICAM1/IFNG/MMP3/IL1B/CXCL8 | 9 |
| hsa04064 | NF-kappa B signaling pathway | 9/55 | 104/8081 | 2.65E-08 | 8.85E-08 | 2.65E-08 | PTGS2/BCL2/BCL2L1/NFKBIA/ICAM1/PLAU/IL1B/CXCL8/PARP1 | 9 |
| hsa04660 | T cell receptor signaling pathway | 9/55 | 104/8081 | 2.65E-08 | 8.85E-08 | 2.65E-08 | JUN/AKT1/MAPK1/IL10/NFKBIA/IL2/IFNG/IL4/RAF1 | 9 |
| hsa04919 | Thyroid hormone signaling pathway | 9/55 | 121/8081 | 1.00E-07 | 3.17E-07 | 9.52E-08 | CASP9/AKT1/CCND1/MAPK1/MDM2/RAF1/HIF1A/STAT1/MYC | 9 |
| hsa05135 | Yersinia infection | 9/55 | 137/8081 | 2.92E-07 | 8.84E-07 | 2.65E-07 | JUN/AKT1/MAPK1/IL10/IL6/NFKBIA/IL2/IL1B/CXCL8 | 9 |
| hsa04915 | Estrogen signaling pathway | 9/55 | 138/8081 | 3.11E-07 | 9.13E-07 | 2.74E-07 | PGR/BCL2/JUN/EGFR/AKT1/MMP2/MMP9/MAPK1/RAF1 | 9 |
| hsa05010 | Alzheimer disease | 9/55 | 369/8081 | 0.00077074 | 0.001511254 | 0.000453376 | PTGS2/CASP9/CASP3/CASP8/AKT1/MAPK1/IL6/RAF1/IL1B | 9 |
| hsa05321 | Inflammatory bowel disease | 8/55 | 65/8081 | 1.02E-08 | 3.77E-08 | 1.13E-08 | JUN/IL10/IL6/IL2/IFNG/IL4/STAT1/IL1B | 8 |
| hsa05211 | Renal cell carcinoma | 8/55 | 69/8081 | 1.65E-08 | 5.79E-08 | 1.74E-08 | JUN/AKT1/VEGFA/CDKN1A/MAPK1/MET/RAF1/HIF1A | 8 |
| hsa05230 | Central carbon metabolism in cancer | 8/55 | 70/8081 | 1.85E-08 | 6.39E-08 | 1.92E-08 | EGFR/AKT1/MAPK1/ERBB2/MET/RAF1/HIF1A/MYC | 8 |
| hsa04110 | Cell cycle | 8/55 | 124/8081 | 1.63E-06 | 4.35E-06 | 1.30E-06 | CCND1/CDKN1A/RB1/MDM2/PCNA/CCNB1/MYC/CHEK2 | 8 |
| hsa04380 | Osteoclast differentiation | 8/55 | 128/8081 | 2.07E-06 | 5.46E-06 | 1.64E-06 | JUN/AKT1/MAPK1/NFKBIA/PPARG/IFNG/STAT1/IL1B | 8 |
| hsa04932 | Non-alcoholic fatty liver disease | 8/55 | 150/8081 | 6.80E-06 | 1.66E-05 | 4.98E-06 | BAX/JUN/CASP3/CASP8/AKT1/IL6/IL1B/CXCL8 | 8 |
| hsa04215 | Apoptosis - multiple species | 7/55 | 32/8081 | 1.35E-09 | 5.85E-09 | 1.76E-09 | BCL2/BAX/CASP9/CASP3/CASP8/BCL2L1/BIRC5 | 7 |
| hsa05144 | Malaria | 7/55 | 50/8081 | 3.64E-08 | 1.19E-07 | 3.58E-08 | IL10/IL6/ICAM1/IFNG/MET/IL1B/CXCL8 | 7 |
| hsa05134 | Legionellosis | 7/55 | 57/8081 | 9.28E-08 | 2.99E-07 | 8.98E-08 | CASP9/CASP3/CASP8/IL6/NFKBIA/IL1B/CXCL8 | 7 |
| hsa04370 | VEGF signaling pathway | 7/55 | 59/8081 | 1.19E-07 | 3.70E-07 | 1.11E-07 | PTGS2/CASP9/AKT1/VEGFA/MAPK1/RAF1/HSPB1 | 7 |
| hsa05133 | Pertussis | 7/55 | 76/8081 | 6.95E-07 | 1.90E-06 | 5.71E-07 | JUN/CASP3/MAPK1/IL10/IL6/IL1B/CXCL8 | 7 |
| hsa04658 | Th1 and Th2 cell differentiation | 7/55 | 92/8081 | 2.56E-06 | 6.65E-06 | 2.00E-06 | JUN/MAPK1/NFKBIA/IL2/IFNG/IL4/STAT1 | 7 |
| hsa05231 | Choline metabolism in cancer | 7/55 | 98/8081 | 3.92E-06 | 9.92E-06 | 2.98E-06 | JUN/EGFR/AKT1/MAPK1/EGF/RAF1/HIF1A | 7 |
| hsa05146 | Amoebiasis | 7/55 | 102/8081 | 5.12E-06 | 1.27E-05 | 3.80E-06 | CASP3/IL10/IL6/IFNG/IL1B/CXCL8/HSPB1 | 7 |
| hsa04722 | Neurotrophin signaling pathway | 7/55 | 119/8081 | 1.42E-05 | 3.39E-05 | 1.02E-05 | BCL2/BAX/JUN/AKT1/MAPK1/NFKBIA/RAF1 | 7 |
| hsa04921 | Oxytocin signaling pathway | 7/55 | 154/8081 | 7.50E-05 | 0.000176564 | 5.30E-05 | PTGS2/JUN/EGFR/CCND1/CDKN1A/MAPK1/RAF1 | 7 |
| hsa04217 | Necroptosis | 7/55 | 159/8081 | 9.18E-05 | 0.000211103 | 6.33E-05 | BCL2/BAX/CASP8/IFNG/STAT1/IL1B/PARP1 | 7 |
| hsa04015 | Rap1 signaling pathway | 7/55 | 210/8081 | 0.000509496 | 0.001029285 | 0.000308785 | EGFR/AKT1/VEGFA/MAPK1/MET/EGF/RAF1 | 7 |
| hsa05020 | Prion disease | 7/55 | 273/8081 | 0.002353952 | 0.004241355 | 0.001272407 | BAX/CASP9/CASP3/MAPK1/IL6/CAV1/IL1B | 7 |
| hsa04060 | Cytokine-cytokine receptor interaction | 7/55 | 295/8081 | 0.003630455 | 0.006369218 | 0.001910766 | IL10/IL6/IL2/IFNG/IL4/IL1B/CXCL8 | 7 |
| hsa05216 | Thyroid cancer | 6/55 | 37/8081 | 1.49E-07 | 4.57E-07 | 1.37E-07 | BAX/CCND1/CDKN1A/MAPK1/PPARG/MYC | 6 |
| hsa05416 | Viral myocarditis | 6/55 | 60/8081 | 2.84E-06 | 7.27E-06 | 2.18E-06 | CASP9/CASP3/CASP8/CCND1/ICAM1/CAV1 | 6 |
| hsa05120 | Epithelial cell signaling in Helicobacter pylori infection | 6/55 | 70/8081 | 7.05E-06 | 1.70E-05 | 5.10E-06 | JUN/CASP3/EGFR/NFKBIA/MET/CXCL8 | 6 |
| hsa04140 | Autophagy - animal | 6/55 | 137/8081 | 0.000311773 | 0.000656363 | 0.000196909 | BCL2/AKT1/BCL2L1/MAPK1/RAF1/HIF1A | 6 |
| hsa04072 | Phospholipase D signaling pathway | 6/55 | 148/8081 | 0.000471887 | 0.000963035 | 0.000288911 | EGFR/AKT1/MAPK1/EGF/RAF1/CXCL8 | 6 |
| hsa04062 | Chemokine signaling pathway | 6/55 | 192/8081 | 0.001832152 | 0.003424582 | 0.001027375 | AKT1/MAPK1/NFKBIA/RAF1/STAT1/CXCL8 | 6 |
| hsa05143 | African trypanosomiasis | 5/55 | 37/8081 | 4.48E-06 | 1.12E-05 | 3.36E-06 | IL10/IL6/ICAM1/IFNG/IL1B | 5 |
| hsa05221 | Acute myeloid leukemia | 5/55 | 67/8081 | 8.50E-05 | 0.00019776 | 5.93E-05 | AKT1/CCND1/MAPK1/RAF1/MYC | 5 |
| hsa04917 | Prolactin signaling pathway | 5/55 | 70/8081 | 0.000104933 | 0.000235803 | 7.07E-05 | AKT1/CCND1/MAPK1/RAF1/STAT1 | 5 |
| hsa04662 | B cell receptor signaling pathway | 5/55 | 82/8081 | 0.000222374 | 0.000483421 | 0.000145026 | JUN/AKT1/MAPK1/NFKBIA/RAF1 | 5 |
| hsa05204 | Chemical carcinogenesis | 5/55 | 83/8081 | 0.000235412 | 0.000506263 | 0.000151879 | PTGS2/GSTP1/CYP3A4/CYP1A1/GSTM1 | 5 |
| hsa04912 | GnRH signaling pathway | 5/55 | 93/8081 | 0.00040023 | 0.000825217 | 0.000247565 | JUN/EGFR/MMP2/MAPK1/RAF1 | 5 |
| hsa04914 | Progesterone-mediated oocyte maturation | 5/55 | 100/8081 | 0.000559267 | 0.001118534 | 0.00033556 | PGR/AKT1/MAPK1/CCNB1/RAF1 | 5 |
| hsa04928 | Parathyroid hormone synthesis, secretion and action | 5/55 | 106/8081 | 0.000729914 | 0.001445375 | 0.000433613 | BCL2/EGFR/CDKN1A/MAPK1/RAF1 | 5 |
| hsa04071 | Sphingolipid signaling pathway | 5/55 | 119/8081 | 0.001230388 | 0.002343596 | 0.000703079 | BCL2/BAX/AKT1/MAPK1/RAF1 | 5 |
| hsa04650 | Natural killer cell mediated cytotoxicity | 5/55 | 131/8081 | 0.001885 | 0.003461716 | 0.001038515 | CASP3/MAPK1/ICAM1/IFNG/RAF1 | 5 |
| hsa04371 | Apelin signaling pathway | 5/55 | 137/8081 | 0.002294365 | 0.004171572 | 0.001251472 | AKT1/CCND1/MAPK1/RAF1/SPP1 | 5 |
| hsa04934 | Cushing syndrome | 5/55 | 155/8081 | 0.003911756 | 0.006803054 | 0.002040916 | EGFR/CCND1/CDKN1A/MAPK1/RB1 | 5 |
| hsa04024 | cAMP signaling pathway | 5/55 | 216/8081 | 0.015330559 | 0.024726708 | 0.007418012 | JUN/AKT1/MAPK1/NFKBIA/RAF1 | 5 |
| hsa04020 | Calcium signaling pathway | 5/55 | 240/8081 | 0.02308297 | 0.034974197 | 0.010492259 | EGFR/VEGFA/ERBB2/MET/EGF | 5 |
| hsa05330 | Allograft rejection | 4/55 | 38/8081 | 0.0001194 | 0.000265333 | 7.96E-05 | IL10/IL2/IFNG/IL4 | 4 |
| hsa05332 | Graft-versus-host disease | 4/55 | 42/8081 | 0.000177437 | 0.000389971 | 0.000116991 | IL6/IL2/IFNG/IL1B | 4 |
| hsa04672 | Intestinal immune network for IgA production | 4/55 | 49/8081 | 0.000324232 | 0.000675483 | 0.000202645 | IL10/IL6/IL2/IL4 | 4 |
| hsa04929 | GnRH secretion | 4/55 | 64/8081 | 0.000901604 | 0.001750687 | 0.000525206 | AKT1/MAPK1/RAF1/SPP1 | 4 |
| hsa04664 | Fc epsilon RI signaling pathway | 4/55 | 68/8081 | 0.001132613 | 0.002178102 | 0.000653431 | AKT1/MAPK1/IL4/RAF1 | 4 |
| hsa04520 | Adherens junction | 4/55 | 71/8081 | 0.001331092 | 0.002511495 | 0.000753448 | EGFR/MAPK1/ERBB2/MET | 4 |
| hsa00980 | Metabolism of xenobiotics by cytochrome P450 | 4/55 | 78/8081 | 0.001886635 | 0.003461716 | 0.001038515 | GSTP1/CYP3A4/CYP1A1/GSTM1 | 4 |
| hsa04540 | Gap junction | 4/55 | 88/8081 | 0.002933348 | 0.005238122 | 0.001571437 | EGFR/MAPK1/EGF/RAF1 | 4 |
| hsa04061 | Viral protein interaction with cytokine and cytokine receptor | 4/55 | 100/8081 | 0.004644906 | 0.008008459 | 0.002402538 | IL10/IL6/IL2/CXCL8 | 4 |
| hsa04726 | Serotonergic synapse | 4/55 | 115/8081 | 0.007597674 | 0.012877414 | 0.003863224 | PTGS2/CASP3/MAPK1/RAF1 | 4 |
| hsa04935 | Growth hormone synthesis, secretion and action | 4/55 | 119/8081 | 0.008554274 | 0.01437693 | 0.004313079 | AKT1/MAPK1/RAF1/STAT1 | 4 |
| hsa04550 | Signaling pathways regulating pluripotency of stem cells | 4/55 | 143/8081 | 0.015964888 | 0.025543821 | 0.007663146 | AKT1/MAPK1/RAF1/MYC | 4 |
| hsa04390 | Hippo signaling pathway | 4/55 | 157/8081 | 0.021720898 | 0.033416766 | 0.01002503 | CCND1/BIRC5/MYC/RASSF1 | 4 |
| hsa04530 | Tight junction | 4/55 | 169/8081 | 0.0275548 | 0.041126568 | 0.01233797 | JUN/CCND1/PCNA/ERBB2 | 4 |
| hsa04940 | Type I diabetes mellitus | 3/55 | 43/8081 | 0.003037071 | 0.005375348 | 0.001612604 | IL2/IFNG/IL1B | 3 |
| hsa05320 | Autoimmune thyroid disease | 3/55 | 53/8081 | 0.005495475 | 0.009393974 | 0.002818192 | IL10/IL2/IL4 | 3 |
| hsa04623 | Cytosolic DNA-sensing pathway | 3/55 | 63/8081 | 0.008881222 | 0.014802037 | 0.004440611 | IL6/NFKBIA/IL1B | 3 |
| hsa04137 | Mitophagy - animal | 3/55 | 68/8081 | 0.010944243 | 0.018089658 | 0.005426897 | JUN/BCL2L1/HIF1A | 3 |
| hsa04622 | RIG-I-like receptor signaling pathway | 3/55 | 70/8081 | 0.011840686 | 0.019410961 | 0.005823288 | CASP8/NFKBIA/CXCL8 | 3 |
| hsa00982 | Drug metabolism - cytochrome P450 | 3/55 | 72/8081 | 0.012778345 | 0.020777797 | 0.006233339 | GSTP1/CYP3A4/GSTM1 | 3 |
| hsa00983 | Drug metabolism - other enzymes | 3/55 | 80/8081 | 0.016946458 | 0.026899139 | 0.008069742 | GSTP1/CYP3A4/GSTM1 | 3 |
| hsa04211 | Longevity regulating pathway | 3/55 | 89/8081 | 0.022444929 | 0.034267067 | 0.01028012 | BAX/AKT1/PPARG | 3 |
| hsa04350 | TGF-beta signaling pathway | 3/55 | 94/8081 | 0.025872837 | 0.038906522 | 0.011671956 | MAPK1/IFNG/MYC | 3 |
| hsa04666 | Fc gamma R-mediated phagocytosis | 3/55 | 97/8081 | 0.028057585 | 0.041566792 | 0.012470038 | AKT1/MAPK1/RAF1 | 3 |
| hsa04640 | Hematopoietic cell lineage | 3/55 | 99/8081 | 0.029567306 | 0.043481333 | 0.0130444 | IL6/IL4/IL1B | 3 |
| hsa01523 | Antifolate resistance | 2/55 | 31/8081 | 0.018641833 | 0.029127864 | 0.008738359 | IL6/IL1B | 2 |
| hsa05310 | Asthma | 2/55 | 31/8081 | 0.018641833 | 0.029127864 | 0.008738359 | IL10/IL4 | 2 |
| hsa03410 | Base excision repair | 2/55 | 33/8081 | 0.020985291 | 0.032535335 | 0.0097606 | PCNA/PARP1 | 2 |
